# Catalytic asymmetric synthesis of CF_3_-substituted tertiary propargylic alcohols *via* direct aldol reaction of α-N_3_ amide†
†Electronic supplementary information (ESI) available. CCDC 1498994–1498996. For ESI and crystallographic data in CIF or other electronic format see DOI: 10.1039/c7sc00330g
Click here for additional data file.



**DOI:** 10.1039/c7sc00330g

**Published:** 2017-03-02

**Authors:** Hidetoshi Noda, Fuyuki Amemiya, Karin Weidner, Naoya Kumagai, Masakatsu Shibasaki

**Affiliations:** a Institute of Microbial Chemistry (BIKAKEN) , 3-14-23 Kamiosaki, Shinagawa-ku , Tokyo 141-0021 , Japan . Email: nkumagai@bikaken.or.jp ; Email: mshibasa@bikaken.or.jp

## Abstract

Organofluorine compounds are found in several important classes of chemicals, such as pharmaceuticals, agrochemicals, and functional materials. Chemists have been immensely interested in the development of methodologies for expeditious access to fluorine containing building blocks. In this study, we report a new method for the catalytic asymmetric synthesis of CF_3_-substituted tertiary propargylic alcohols with two contiguous stereogenic centers *via* the direct aldol reaction of an α-N_3_ amide to trifluoromethyl ketones. The key to the success of this method is the identification of a catalyst comprising Cu(ii)/chiral hydroxamic acid to promote the desired aldol reaction, constructing a tetrasubstituted carbon in a highly stereoselective fashion. Despite substantial prior advances in asymmetric catalysis, this class of catalysts has not been utilized for the formation of carbon–carbon bond-forming reactions. Our mechanistic study sheds light on the unique profile of this catalytic system, where the Cu(ii) complex plays a bifunctional role of serving as a Lewis acid and a Brønsted base. Furthermore, the densely functionalized aldol adducts undergo chemoselective transformations, affording a series of fluorine containing chiral building blocks with widespread application.

## Abstract 2

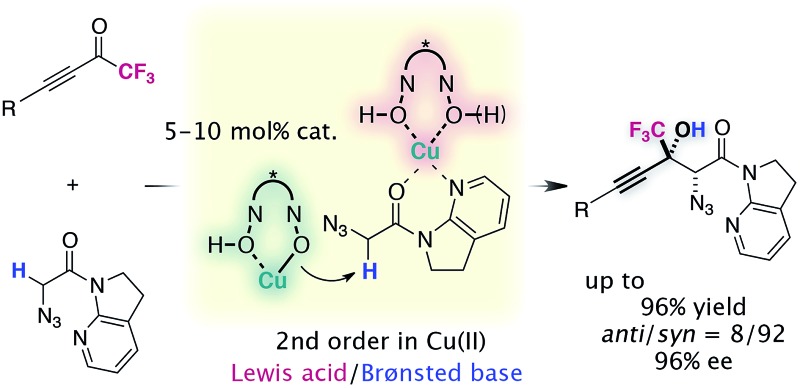
The catalyst comprising Cu(ii)/chiral hydroxamic acid was found to play a bifunctional role in the direct aldol reaction of α-N_3_ amide to alkynyl CF_3_ ketones.

## Introduction

**Fig. 1:**
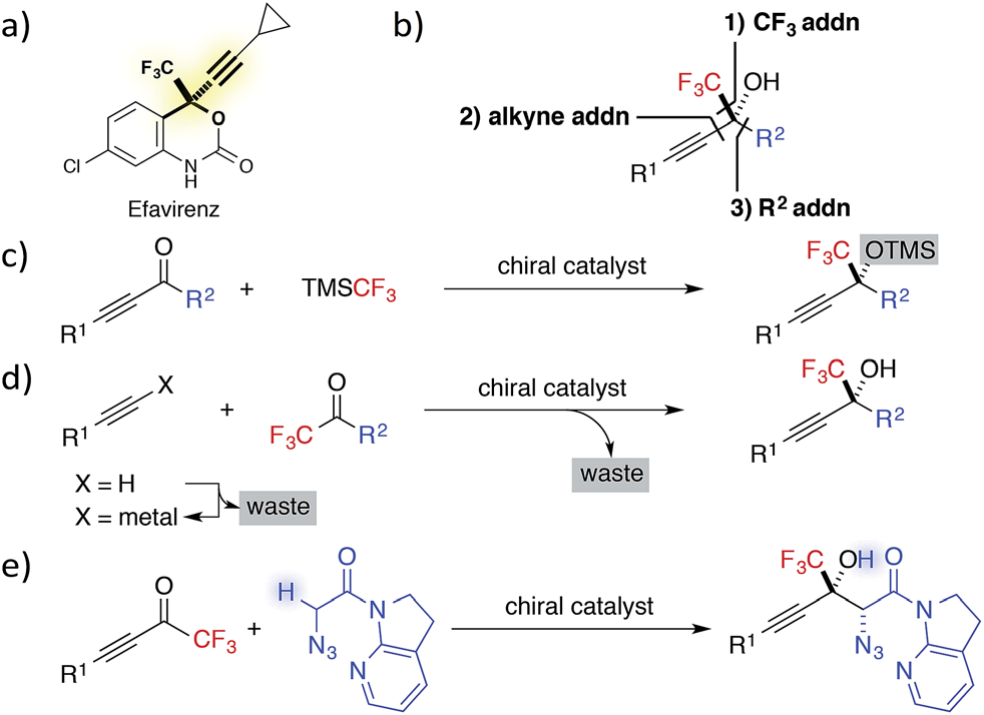
(a) Efavirenz. (b) Three strategies for the construction of CF_3_-substituted tertiary propargylic alcohols. (c) Addition of CF_3_ anion to alkynyl ketones. (d) Addition of alkynylide to CF_3_ ketones. (e) This work: Direct catalytic asymmetric aldol reaction of α-N_3_ amide to alkynyl CF_3_ ketones.

Fluorine containing compounds exhibit broad applications in pharmaceutical, agrochemical, polymer and other chemical industries.^1^ Particularly, enantioenriched trifluoromethyl-substituted tertiary propargylic alcohols are an important class of chiral building blocks, as exemplified in the structures of HIV reverse transcriptase inhibitors Efavirenz and related drugs (Fig. 1a).^2^ Despite their clear benefits, progress toward the catalytic asymmetric synthesis of CF_3_-containing alcohols has been slow,^3^ attributed to the difficulties associated with the construction of tetrasubstituted carbon centres.^4^

In contrast to the preparation of the corresponding secondary alcohols, the catalytic asymmetric synthesis of tertiary alcohols involves the formation of carbon–carbon bonds without resorting to the well-established catalytic enantioselective hydrogenation.^5^ Typically, the chiral building block can be synthesized by three approaches: (1) 1,2-addition of CF_3_ anions to ynones; (2) addition of a terminal alkyne to CF_3_ ketones; and (3) 1,2-addition of a carbon nucleophile to alkynyl CF_3_ ketones (Fig. 1b).

The first strategy commonly employs the Ruppert–Prakash reagent (TMSCF_3_)^6^ as a CF_3_ anion equivalent, due to the gaseous nature of fluoroform and the instability of metalated trifluoromethyl species.^7^ Although high enantioselectivity was recently achieved with the combination of a cinchona alkaloid-based catalyst and catalytic amount of a fluoride source,^8^ an additional step was required to remove a TMS group from the trifluoromethylated product.

The second strategy has been the most investigated, probably because the use of stoichiometric amount of chiral promoter in the original synthesis of Efavirenz^9^ has spurred scientists to develop new catalytic enantioselective alternatives.^10^ While various effective catalytic alkynylations to CF_3_ ketones were documented, most of these methods utilized preformed lithium, magnesium, or zinc alkynylide as the activated nucleophile in the presence of a catalytic amount of chiral promoter.^11^ Thus far, only a limited number of studies, including the first example reported by our group in 2007,^12^ have addressed the direct alkynylation to trifluoromethyl ketones,^13,14^ which avoids the preformation of metal alkynylide and generates the active nucleophile *in situ* with a catalytic Brønsted base.^15^

The third strategy has been the least examined. This fact is partly ascribed to the difficulty in managing the two distinct pathways of 1,2-addition and 1,4-addition,^16^ associated with alkynyl trifluoromethyl ketones, as well as a general problem of overcoming a high activation barrier for the construction of a tetrasubstituted stereogenic centre. The latter factor has been mainly addressed by the use of organometallic nucleophiles, *e.g.*, dialkylzinc,^17^ however the use of latent carbon nucleophiles, *e.g.*, carbonyl compounds, needs to be further developed.^18^ Since the stereoselective addition of enolates to alkynyl CF_3_ ketones appends a carbonyl group to the fluorine containing tertiary propargylic alcohol,^19^ the aldol reaction to the ketones is particularly advantageous for direct access to densely functionalized fluorinated chiral building blocks.^20^

The asymmetric aldol reaction, which affords an enantioenriched β-hydroxy carbonyl moiety with the concomitant formation of a carbon–carbon bond, is a fundamental transformation in organic chemistry.^21^ In the early stage for the development of catalytic asymmetric aldol reactions, chemists relied on the preformed enolate or its equivalent to suppress the retro-aldol reaction and to achieve high diastereo- and enantioselectivity.^22^ Nevertheless, more recently, parent carbonyl compounds have been used as pronucleophiles, avoiding the generation of reagent-derived waste.^23^ This atom-economical,^24^ direct aldol approach has attracted considerable attention from the chemistry community, and a significant number of studies realizing this difficult albeit desirable reaction have been reported in the last two decades.^25^ A majority of studies in this area, however, confines the scope of the aldol donors to aldehydes and ketones. Despite the synthetic utility of aldol adducts derived from carboxylate-type donors, the low acidity of their α-protons has limited their engagements in the direct aldol regime.^26,27^

In our continuous research program on direct enolization chemistry, our group has recently reported that a cooperative catalyst^28^ comprising a soft Lewis acid and a hard Brønsted base effectively promotes the direct aldol and Mannich-type reactions of 7-azaindoline amides, carboxylate-type donor substrates.^29^ The established chemistry involving the azide functionality^30^ has led us to develop a Cu(i)-catalyzed direct aldol reaction of α-N_3_ 7-azaindoline acetamide to aldehydes, affording β-hydroxy-α-azido amides with high diastereo- and enantioselectivity.^31^ Hence, we envisioned that an aldol addition of the amide to alkynyl trifluoromethyl ketones would be an attractive, viable route to furnish enantioenriched CF_3_-substituted tertiary propargylic alcohols.

In this Edge Article, we document our investigations over the course of the reaction development. The key features of this study include (1) identifying that the combination of Cu(ii)/bis-hydroxamic acid (BHA) and Barton's base acts as an effective catalyst to uniquely promote the diastereo- and enantioselective aldol reaction to α-fluorinated ketones; (2) mechanistic studies including NMR spectroscopy, X-ray crystallography, kinetic studies, and DFT calculations to shed light on the properties of this distinctive Lewis acid/Brønsted base catalytic system; (3) further derivatization of the densely functionalized aldol products by chemoselective transformations.

## Results and discussion

### Initial attempts

**Scheme 1:**
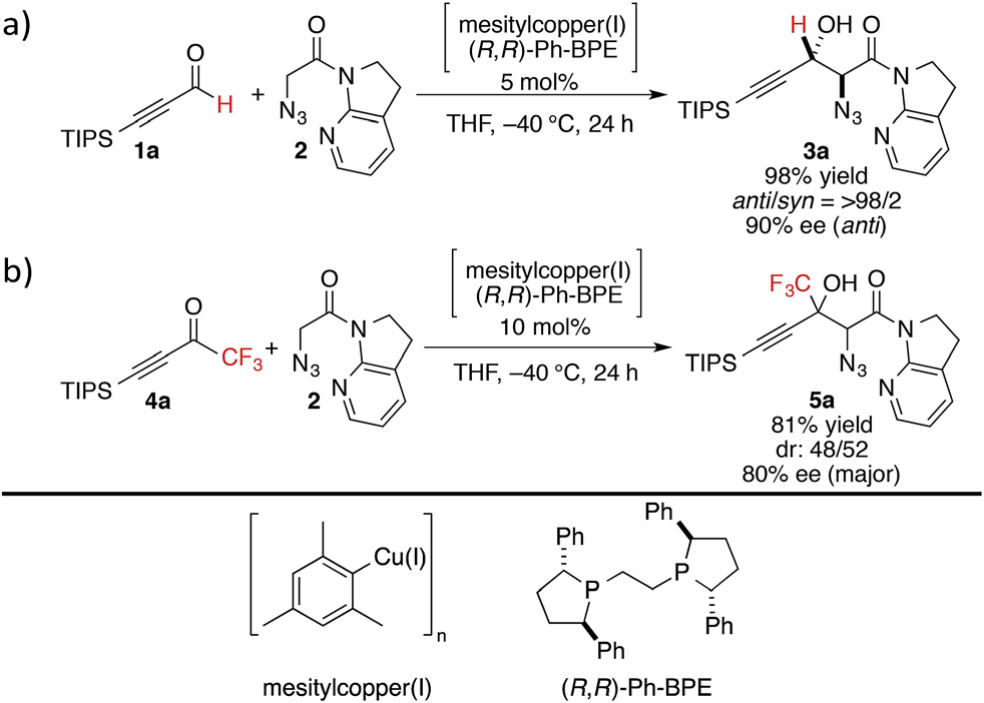
Head-to-head comparison of aldol additions to aldehyde and CF_3_ ketone under Cu(i) catalysis.

At the outset, we sought to extend the previous catalytic system optimized for the aldol reaction of α-N_3_ amide **2** to aldehydes. Given that the catalyst comprising Cu(i)/chiral phosphine ligand/hard Brønsted base has exhibited broad utility in a wide range of asymmetric transformations, the aldol addition to alkynyl trifluoromethyl ketones was expected to also proceed in a highly stereoselective manner. However, this was not the case, and almost no diastereoselectivity was observed for the addition to a structurally related trifluoromethyl ketone. Scheme 1 illustrates the head-to-head comparison of the aldol addition reactions to the ynal and CF_3_ ketone. The previously optimized Cu(i) catalyst afforded a good yield of aldol product **3a** derived from ynal **1a** with excellent anti- and enantioselectivity, while aldol product **5a** from trifluoromethyl ketone **4a** was obtained with almost 1 : 1 diastereoselectivity in lower yield and ee. The absence of a retro-aldol reaction under Cu(i)-based catalytic conditions^32^ suggested that the low diastereoselectivity arose from the low facial selectivity of the ketone under the catalysis. A wide range of different chiral ligands with Cu(i), Brønsted bases, and additives were extensively screened, but substantial improvements were not observed. Hence, we turned our attention to other metal sources with different ionic radii and/or coordination modes other than the tetrahedral geometry of the Cu(i) complex. These changes were expected to cause a slight change in the transition state in the aldol reaction, resulting in the improvement of diastereoselectivity.

### Conditions screening

Trifluoromethyl ketone **4a** was selected as a model substrate, and the reaction was screened with catalytic amounts of Barton's base in THF at –40 °C (Table 1). A quick examination of metal sources revealed that Cu(OTf)_2_ effectively catalyzed the reaction with marginal diastereoselectivity, whereas the other metal salts barely promoted the reaction (entries 1–4). Although numerous Cu(ii)-based Lewis acid catalysts have been established as privileged asymmetric catalysts,^33^ the activation of carbonyl compounds with Cu(ii) catalysts for their enolization has not been well documented.^34^ Neither bisoxazoline (BOX)^35^ **6** nor pyridine bisoxazoline (PyBOX)^36^ **7** was found to be suitable for this specific transformation, furnishing almost racemic products (entries 5 and 6). Extensive screening of the chiral ligands for Cu(ii) eventually led to the identification of BHA **8** (entry 7), which was originally developed by Yamamoto for the vanadium-catalyzed epoxidation of allylic alcohols.^37^ Using ligand **8**, aldol adduct **5a** was formed with preference for the *syn* diastereomer in 82% ee. Despite the large number of metal–hydroxamic acid complexes reported in the literature,^38^ chiral hydroxamic acids have not been employed for asymmetric carbon–carbon bond-forming reactions.^39^ Compared with **8**, BHA **9**, which has a shorter linker, afforded lower selectivities, albeit with higher yield (entry 8).

**Table 1:** Conditions screening for direct catalytic asymmetric aldol reaction of **2** to CF_3_ ketone **4a**

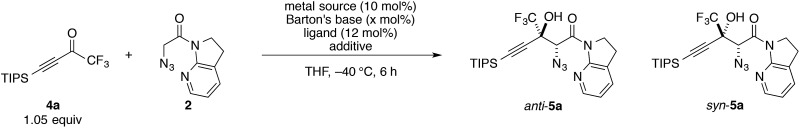							
Entry	Metal source	Ligand	Additive^*a*^	*x* (mol%)	Yield^*b*^ (%)	*anti*/*syn* ^*b*^	ee^*c*^ (%)
1^*d*^	LiOTf	—	—	10	5	45/55	—
2^*d*^	Fe(OTf)_3_	—	—	10	Trace	nd	—
3^*d*^	Zn(OTf)_2_	—	—	10	3	46/54	—
4^*d*^	Cu(OTf)_2_	—	—	10	91	63/37	—
5	Cu(OTf)_2_	BOX **6**	—	10	60	50/50	1
6	Cu(OTf)_2_	PyBOX **7**	—	10	9	65/35	1
7	Cu(OTf)_2_	BHA **8**	—	10	64	29/71	82
8	Cu(OTf)_2_	BHA **9**	—	10	91	53/47	25
9	Cu(OTf)_2_	BHA **8**	MS3A	10	17	41/59	90
10	Cu(OTf)_2_	BHA **8**	MS4A	10	Trace	nd	nd
11	Cu(OTf)_2_	BHA **8**	MS5A	10	72	25/75	72
12	Cu(OTf)_2_	BHA **8**	MS13X	10	78	22/78	93
13	Cu(OTf)_2_	BHA **8**	CaSO_4_	10	88	26/74	74
14	Cu(OTf)_2_	BHA **8**	MS13X	5	81	18/82	95
15	Cu(OTf)_2_	BHA **8**	MS13X	24	12	27/73	91
16^*e*^	Cu(OTf)_2_	BHA **8**	MS13X	5	93	17/83	96
17^*e*^	CuOTf·C_6_H_6_	BHA **8**	MS13X	5	16	18/82	88
18^*e*^	Cu(OTf)_2_	BHA **10**	MS13X	5	91	43/57	89
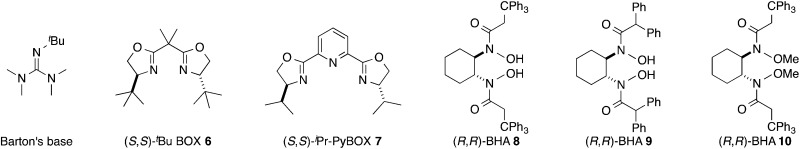							

^*a*^500% w/w additive was used.

^*b*^Yield and diastereomer ratio were determined by ^1^H NMR analysis of unpurified reaction mixture.

^*c*^Enantiomeric excess of the *syn* isomer was determined with normal phase HPLC on a chiral support.

^*d*^Reaction time was 24 h.

^*e*^1.2 equiv. of **4a** was employed. nd: not determined.

Further investigation of various additives^40^ showed the beneficial effect of MS13X on the reactivity and selectivities (entries 9–13). As other additives such as MS3A and 4A afforded lower yields, MS13X is less likely to serve as a simple desiccant in this case (*vide infra*). While the screening of solvents and counter anions did not reveal favourable effects, the loading of Brønsted bases was found to play an important role in the reaction outcomes; a lower loading of Barton's base (5 mol%) afforded the product with slightly higher selectivities without compromising reaction efficiency; more interestingly, a higher loading of Barton's base (24 mol%) significantly retarded the reaction (entries 14 and 15). Increasing the amount of ketone **4a** to 1.2 equiv. brought the reaction to its completion (entry 16). Both the Cu(ii) metal source and BHA **8** were crucial for high reactivity and selectivity, as the use of either the CuOTf·C_6_H_6_ complex or BHA **10**, which is a methyl ester variant of BHA **8**, under otherwise identical conditions afforded inferior results (entries 17 and 18).

### Mechanistic insights

**Scheme 2:**
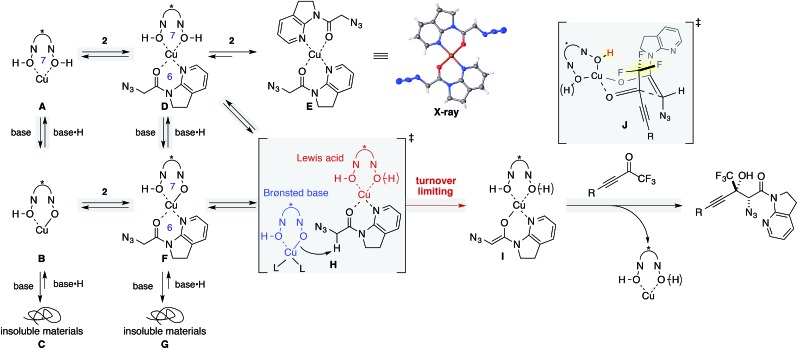
Proposed reaction pathway supported by mechanistic studies.

The dramatic decrease in reactivity with a higher loading of Brønsted base (Table 1, entry 15) led us to garner mechanistic insights, including the effects of the Brønsted base. Since Yamamoto and coworkers have used alkoxide metal sources such as VO(O^i^Pr)_3_, Zr(O^*t*^Bu)_4_, and Hf(O^*t*^Bu)_4_ for BHA ligands, their catalyst comprised the deprotonated hydroxamate ligand.^41^ On the other hand, the catalytic system used herein comprised Cu(OTf)_2_ as the metal source, and the ligand was assumed to retain its acidic protons as a form of hydroxamic acids. Barton's base was originally included for the enolization of amide **2**, but a higher loading of Barton's base can also deprotonate acidic protons on the ligand, leading to the irreversible formation of catalytically unreactive species. This hypothesis was evaluated by NMR studies (Scheme 2, left column **A–C**; see the ESI† for details). The ^1^H NMR spectrum of ligand **8** in THF-*d*_8_ displayed a sharp peak resulting from acidic protons at 8.5 ppm (Fig. S2†); this result is indicative of hydrogen bonding with carbonyl oxygen, as evidenced by its solid state structure (Fig. S4†). The sharp peak broadened at around 7.7 ppm upon the addition of Cu(OTf)_2_ (Fig. S2†). The symmetrical spectrum of the 1 : 1 mixture of Cu(OTf)_2_ and BHA **8** in THF-*d*_8_ suggested that the ligand accommodated a slightly larger Cu(ii) cation than Zr(iv) and Hf(iv) cations,^42^ and indicated that the addition of Cu(ii) salt disrupted the hydrogen bonding, affording a symmetric 1 : 1 Cu/ligand complex presumably *via* a 7-membered chelated structure (**A**).^43^ The addition of Barton's base to the complex solution led to a cloudy solution, which is proportional to the amount of base added (**B** and **C**), and the introduction of 2 equiv. of the base led to the immediate formation of a precipitate (Fig. S2c†), which was insoluble in any of the solvents including DMSO, MeOH, and CHCl_3_.

Similar NMR experiments were performed in the presence of 1 equiv. of amide **2** (**D–G**). The addition of the amide to a solution of 1 : 1 Cu/ligand (**A**) also resulted in a precipitate at ambient temperature, which consisted of a 1 : 2 Cu/amide complex (**E**) without ligand incorporation. The structure of **E** was unambiguously determined by X-ray crystallography. In solution, a trace amount of the free amide was detected in the ^1^H NMR spectrum, indicating that almost all amides bound to the Cu(ii) cation, replacing the BHA ligand on copper. The higher affinity of amide **2** as compared with ligand **8** for the metal is attributed to the weak binding nature of hydroxamic acids and to the thermodynamic preference of a 6-membered chelation for the square planar coordination geometry of Cu(ii) over a 7-membered chelation. Although **D** appears rather labile, it should be noted that the formation of precipitates of **E** in the stoichiometric experiment does not imply that a similar event will occur in the real catalytic system at –40 °C.^44^

In contrast, a slightly cloudy solution was obtained when 1 equiv. of amide **2** was introduced to a solution of 1 : 1 : 1 Cu(OTf)_2_/ligand/Barton's base (**B** to **F**). From the ^1^H NMR spectrum, all components were present in solution, indicating that the addition of Barton's base rendered the hydroxamic acids to be more strongly bound ligands by the deprotonation of the acidic hydrogen. The solution again formed insoluble materials after the addition of another equivalent of the Brønsted base (**G**). We conclude that higher loading of Barton's base disrupts the catalytic system by the formation of insoluble materials (**C**, **G**). MS13X possibly served as a reservoir to keep amide **2** and Barton's base inside the large pore, minimizing the unproductive pathways such as formations of **C**, **E**, and **G**.^45^

We then focused on an enolization event under the present catalysis. Our previous studies with 7-azaindoline amides have revealed that Cu(i)/phosphine complex triggers the transition of *E*-amide to *Z*-amide.^28^ The solid state structure of **E** in Scheme 2 demonstrates that amide **2** also binds to Cu(ii) in a bidentate manner by the flipping of the amide conformation. As shown in eqn (1), structurally similar amides failed to afford aldol products under otherwise identical conditions, suggesting that the bidentate coordination of **2** to the Lewis acid is also crucial to the facile formation of the amide enolate under the present Cu(ii) catalysis.
1

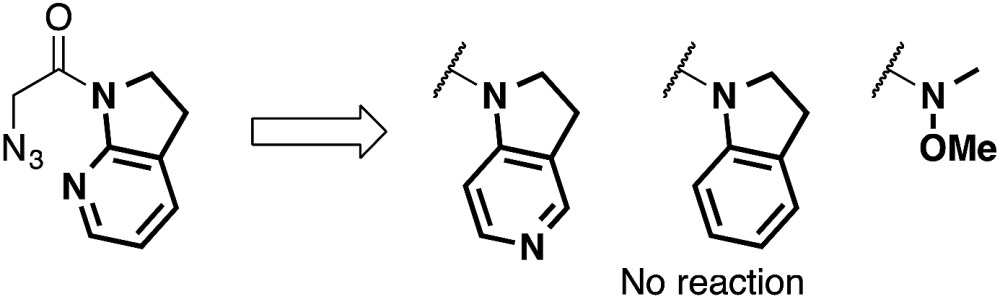




**Fig. 2:**
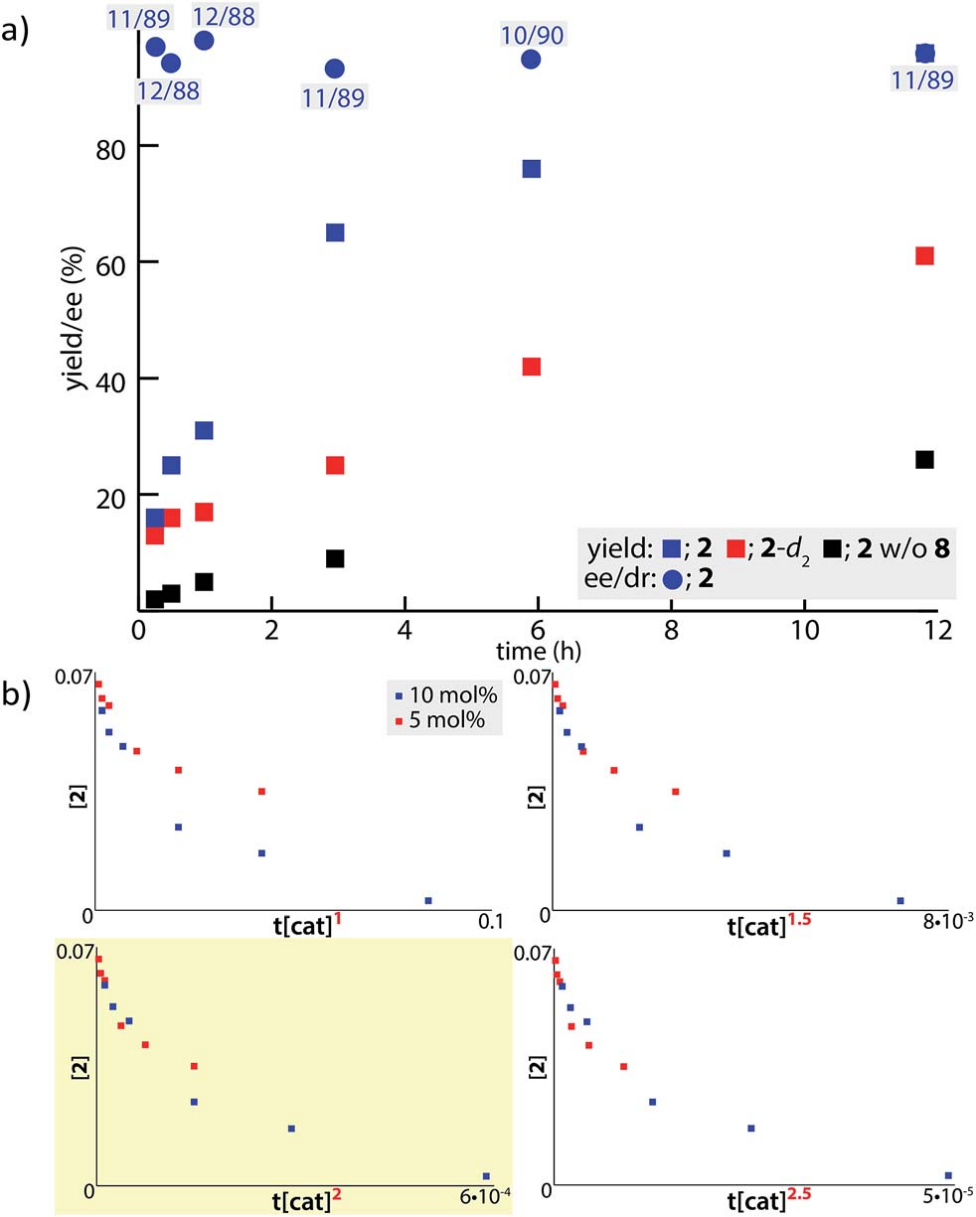
Kinetic profile of the Cu(ii)-catalyzed aldol reaction with **4a**. (a) Time course study with **2** (blue) and **2**-*d*_2_ (red) from the initial concentration of 70 mM at –60 °C. The numbers in grey boxes indicate the diastereomeric ratio of the product (*anti*/*syn*). The data with black square was obtained from control experiments without ligand **8**. (b) Normalized time scale plots with 5 and 10 mol% catalyst loading.

In order to examine the enolate formation further, the aldol reaction was monitored over 12 h with amide **2** or α,α-dideuterated amide **2**-*d*_2_. The rate of this asymmetric reaction under the standard conditions was fast (81% conversion after 30 min at –40 °C), and the time-course studies were conducted under more dilute conditions with a catalyst loading of 10 mol% at –60 °C (Fig. 2a, **2**: blue squares, **2**-*d*_2_: red squares). Comparison of the plots of **2** and **2**-*d*_2_ clearly illustrates that enolate formation is the turnover-limiting step in this aldol reaction. The observed consistency in the diastereoselectivity and enantioselectivity of product **5a** over the course of the reaction (ee: blue circles, *anti*/*syn*: grey boxes) indicates that the present catalytic system is free from retro-aldol pathways,^46^ as well as autocatalysis or related processes.^47^ The reaction was also monitored with 5 mol% catalyst at –60 °C. A graphical representation of these data sets (10 mol% and 5 mol% catalyst loading) by a normalized time scale method^48^ indicated that the order of the reaction in the catalyst is greater than 2 (Fig. 2b; see ESI† for details). The second order dependency on the catalyst corroborates the bifunctional role of the Cu(ii)/BHA **8** complex; one role for the activation of amide **2** as a Lewis acid and the other for the deprotonation of the α-proton as a Brønsted base (Scheme 2, **H**). The enhanced reaction kinetics by the addition of ligand **8** (Fig. 2a, blue squares *vs.* black squares) also supports the hypothesis that Cu-hydroxamate is responsible for the enolization of amide **2** rather than Barton's base.

The present Cu(ii) catalysis was found to promote aldol reactions specifically to α-fluorinated ketones (*vide infra*): aldol adduct **3a** derived from ynal **1a** was obtained in 21% yield with almost 1 : 1 diastereoselectivity (eqn (2)).^49^ Considering the low diastereoselectivity observed with ligand **10** (Table 1, entry 18), the high stereoselectivity possibly arises from non-bonding interactions involving fluorine atoms and acidic protons in the ligand,^50^ such as hydrogen bonding^51^ or ion–dipole interactions (Scheme 2, **J**).^52,53^

2

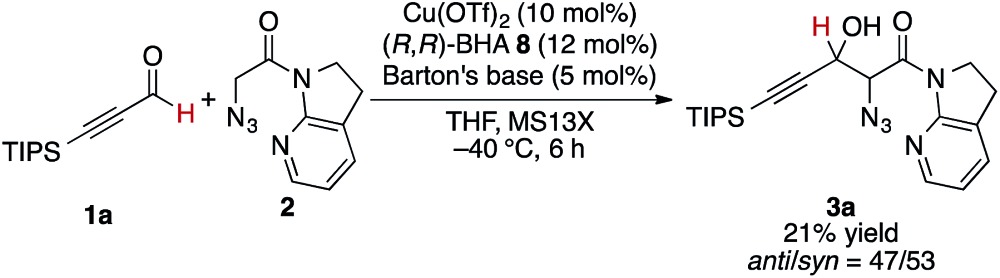




### Substrate scope and limitations

With this insight of reaction mechanism, the scope and limitations of the current catalytic system were examined. As the reaction outcome was determined by the complex equilibria between Cu(ii), ligand **8**, Barton's base, and amide **2**, the loading of the Brønsted base was adjusted according to the reactivity of the substrates employed. The reaction proved remarkably general with respect to the substituents on the alkynyl group of trifluoromethyl ketones, and a series of densely functionalized CF_3_-substituted tertiary propargylic alcohols were obtained in good yield with high *syn*- and enantioselectivity (Table 2). In addition to bulky TIPS group (**5a**), methylene-linked TBS ether was tolerated (**5b**). The present catalytic system also accommodated a long alkyl chain attached with the carbon–carbon triple bond (**5c**). Substituents such as TBDPS ether (**5d**) and primary alkyl chloride (**5e**) at the alkyl chain termini marginally affected the reaction outcome. The aryl substituents on the alkyne were also found to be general in scope. The electronic nature of the substituents (**5f** *vs.* **5g**) did not affect the formation of the corresponding aldol products. Aldol adduct **5f** was obtained with the same selectivity using 5 mol% of the Cu(ii) catalyst on a 2 mmol scale. In addition, reactions smoothly proceeded in the presence of acid-labile acetal (**5h**) or Lewis basic amide (**5i**). These functional groups provide a synthetic handle for further modifications to these high-value chiral building blocks. For **5i**, the hydrated form^54^ of the corresponding trifluoromethyl ketone **4i** was treated with CaSO_4_ in toluene at reflux temperature for 24 h before conducting the aldol reaction.^55^ Notably, potentially detrimental Lewis basic heteroaromatics barely retarded the catalysis, affording the coupled product in 83% yield with good stereoselectivity (**5j**). The cyclohexane ring was compatible (**5k**), whereas the cyclohexene ring comprising an enyne moiety was problematic; neither aldol (1,2-) nor conjugate (1,4- or 1,6-) addition was observed in the case of **5l**. The relative and absolute configurations of aldol product **5g** were determined by X-ray diffraction; the stereochemistry of other products was assigned by analogy. The robustness of this aldol protocol was further demonstrated by the synthesis of **5a** on a gram scale (eqn (3)).^56^

3

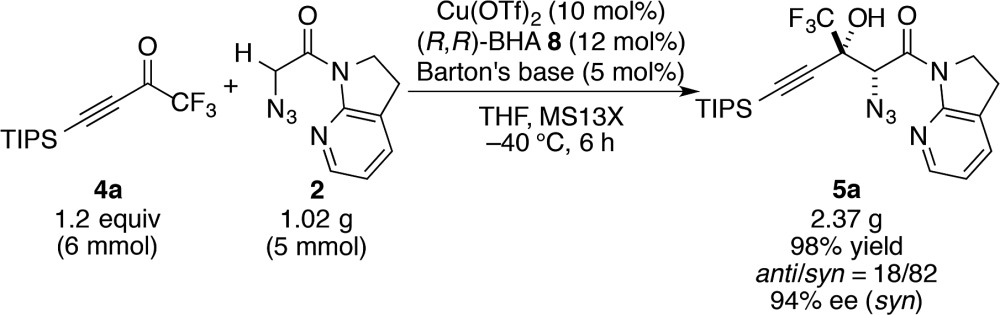




**Table 2:** Scope and limitations of direct catalytic asymmetric aldol reaction to alkynyl CF_3_ ketones^*a*^

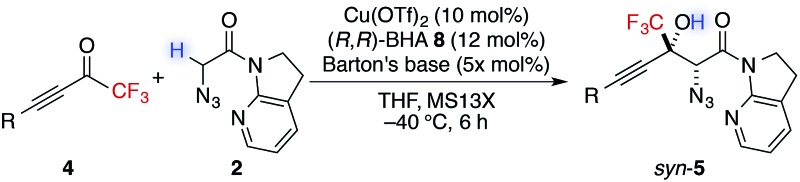
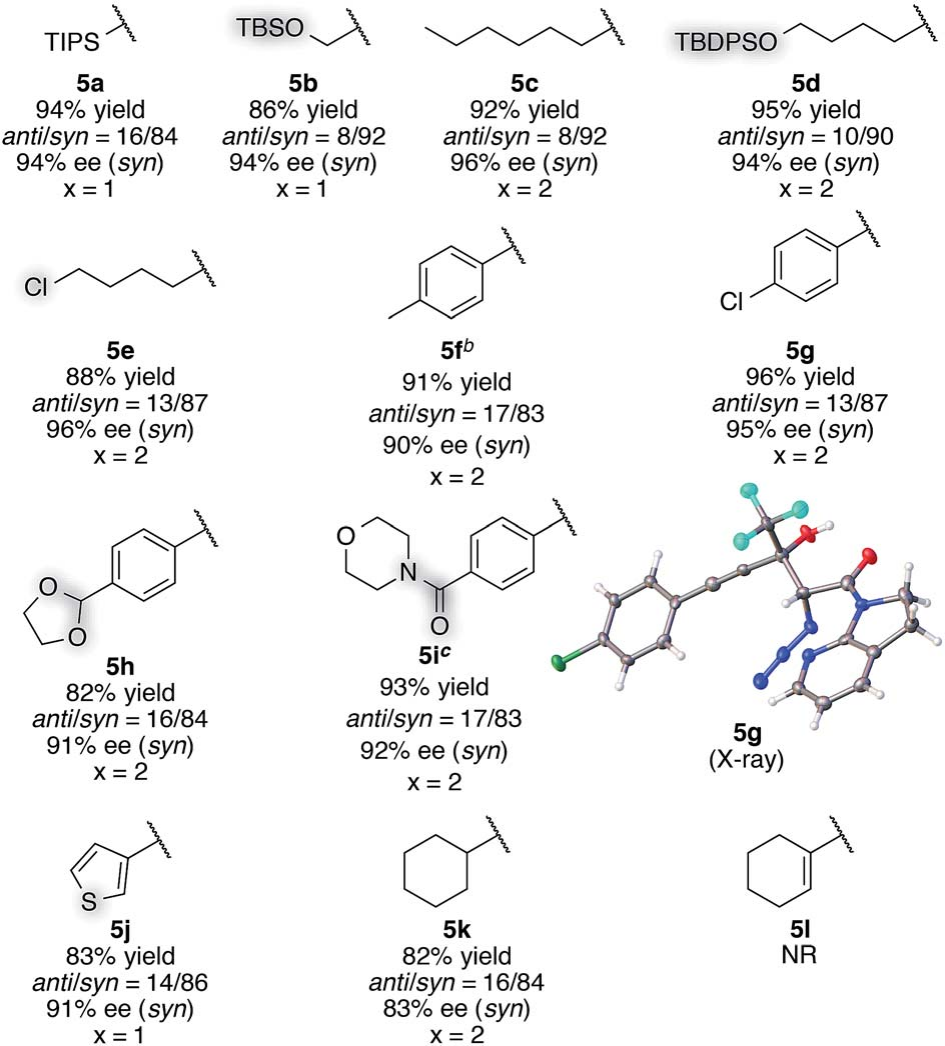

^*a*^Reaction conditions: amide **2** (0.2 mmol, 1.0 equiv.), ketone **4** (1.2 equiv.), Cu(OTf)_2_ (10 mol%), BHA **8** (12 mol%), Barton's base (5 or 10 mol%), THF (0.2 M), –40 °C, 6 h. Yield values refer to isolated yields after purification. Enantiomeric excess of the *syn* isomer was determined with normal phase HPLC on a chiral support.

^*b*^Amide **2** (2 mmol, 1.0 equiv.), Cu(OTf)_2_ (5 mol%), BHA **8** (6 mol%), Barton's base (5 mol%), –40 °C, 12 h.

^*c*^Hydrate form of **4i** was subjected to CaSO_4_ for 24 h in toluene before the aldol reaction.

In addition to trifluoromethyl ketones, other α-fluorinated ketones were briefly examined as electrophiles (Table 3). The substitution of one fluorine atom with a hydrogen atom afforded product **12a** with decreased diastereoselectivity, albeit with high enantioselectivity. On the other hand, the substitution of one fluorine atom with a chlorine or a bromine atom afforded products **12b** and **12c**, respectively, with high stereoselectivity but reduced reactivity, likely due to the increased steric hindrance around the carbonyl group. For product **12c**, higher catalyst loading was required for reasonable conversion within a practical time scale.^57^ These observations of diastereoselectivity are somewhat in agreement with the transition states controlled by non-bonding interactions (Scheme 2); they are possibly explained by the preferred ground-state conformation of CHF_2_, CF_3_, CF_2_Cl, and CF_2_Br ketones (Table S11†). DFT calculations revealed that difluoromethylketones favour the *syn*-coplanar conformation of C=O and C–H to minimize the dipole–dipole interaction between the carbonyl group and fluorine atoms. In contrast, *syn*-orientations of C=O and C–F were observed for the favoured conformations of other α-fluorinated ketones. The preferred conformation of the CHF_2_ ketone in the ground state possibly reflects the diminished energy difference between the transition states, leading to low diastereoselectivity.

**Table 3:** Direct catalytic aldol reactions to other α-fluorinated ketones^*a*^

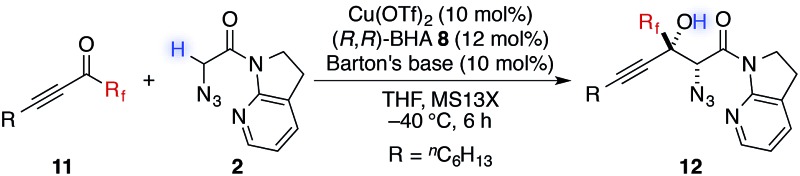
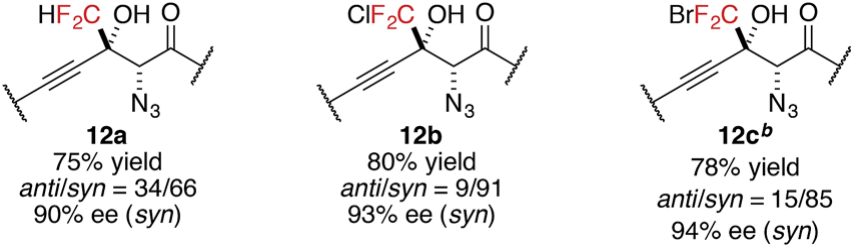

^*a*^Reaction conditions: amide **2** (0.1 mmol, 1.0 equiv.), ketone **11** (1.2 equiv.), Cu(OTf)_2_ (10 mol%), BHA **8** (12 mol%), Barton's base (5 mol%), THF (0.2 M), –40 °C, 6 h. Yield values refer to isolated yields after purification. Enantiomeric excess of the *syn* isomer was determined with normal phase HPLC on a chiral support.

^*b*^Cu(OTf)_2_ (20 mol%), BHA **8** (24 mol%) and Barton's base (20 mol%) were employed.

### Transformations of the aldol adduct

**Scheme 3:**
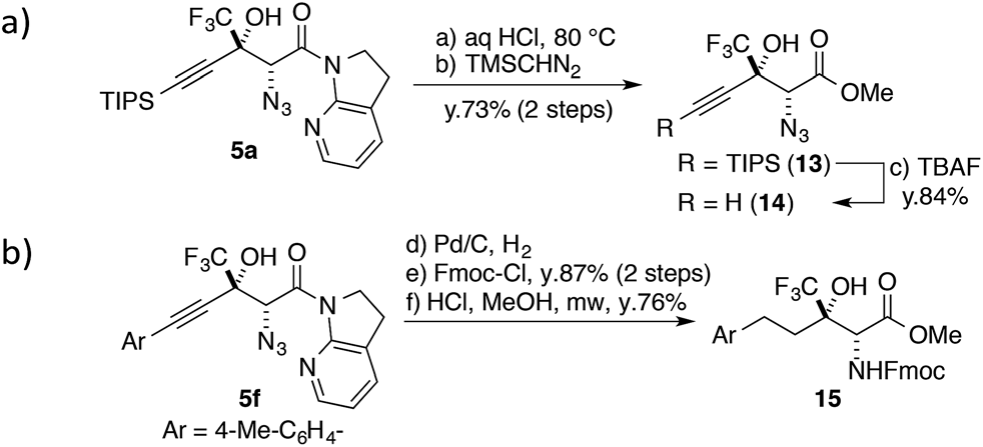
Transformations of aldol adducts.

One of the unique features of this study is the production of CF_3_-containing chiral building blocks decorated by three distinct functional groups, making it possible to further derivatize the aldol products. The 7-azaindoline amide motif was easily hydrolysed under acidic conditions (Scheme 3a); the CF_3_ substituent slowed down the formation of a tertiary cation at the propargylic position,^58^ and the stereochemical integrity was maintained over the course of the transformation. The following treatment with TBAF afforded highly functionalized terminal alkyne **14** in good yield. The alkyne moiety in the aldol product can also be considered as a masked alkyl group (Scheme 3b). The Pd catalyst allowed for the conversion of **5f** to the saturated product with the concomitant reduction of the azide group under a hydrogen atmosphere. After the introduction of the Fmoc group on the nitrogen, the direct conversion from the amide to the corresponding methyl ester was realized under microwave conditions, furnishing fluorinated α-amino acid ester **15**.

## Conclusions

We have identified a catalytic system comprising of Cu(ii)/chiral hydroxamic acid/Barton's base to promote aldol reactions of an α-N_3_ 7-azaindoline acetamide to trifluoromethyl ketones for the asymmetric synthesis of CF_3_-substituted tertiary propargylic alcohols. The catalytic system was studied using a combination of NMR spectroscopy, X-ray crystallography, and kinetic studies. Significant ligand acceleration and second order dependency on the Cu complex established the bifunctional role of the catalyst as a Lewis acid and a Brønsted base.

The scope of this aldol protocol is broad with respect to alkynyl trifluoromethyl ketones, furnishing enantioenriched fluorine containing building blocks bearing additional synthetic handles. In addition to the α-N_3_ carbonyl moiety, silyl ethers, a primary alkyl halide, an acetal, and a morpholine amide were incorporated into the aldol products. The synthetic utility of the aldol products was demonstrated by further chemoselective transformations, including the preparation of fluorinated α-amino acid derivatives.

This study also established chiral hydroxamic acids as a powerful and thus far underexplored class of chiral ligands ripe for further development and exploration in asymmetric catalysis. Current efforts in our group include the investigation of a metal/chiral hydroxamic acid complex in other asymmetric carbon–carbon bond-forming reactions with particular emphasis on gaining a deeper understanding of the nature of the catalyst.
