# Supplementary material for: Catalytic asymmetric synthesis of CF3-substituted tertiary propargylic alcohols via direct aldol reaction of α-N3 amide
Source: Chem Sci. 2017 Mar 2;8(4):3260–9. doi: 10.1039/c7sc00330g (PMC5424470; doi:10.1039/c7sc00330g)

Electronic Supplementary Material (ESI) for Chemical Science.
This journal is © The Royal Society of Chemistry 2017

Supplementary Information

Catalytic Asymmetric Synthesis of CF_3–Substituted Tertiary Propargylic Alcohols *via* Direct Aldol Reaction of α–N_3 Amide

Hidetoshi Noda, Fuyuki Amemiya, Karin Weidner, Naoya Kumagai,* Masakatsu Shibasaki*

Institute of Microbial Chemistry (BIKAKEN), Tokyo, Japan

nkumagai@bikaken.or.jp, mshibasa@bikaken.or.jp

Table of Contents

1. General methods S2
   1–1. Reactions and purifications S2
   1–2. Chracterizations S2
   1–3. Solvents and reagents S2
   1–4. Computational investigations S2
2. Preparation of substrates S3
3. Direct catalytic asymmetric aldol reaction of α–N_3 amide to CF_3 ketones S6
   3–1. Optimization study S6
   3–2. Synthesis of BHA **10** S7
   3–3. Control experiment S8
   3–4. Substrate scope and limitations S8
   3–5. Gram scale synthesis of **5a** and its SDE test on achiral silica gel chromatgraphy S16
4. Transformations of the aldol products S17
5. Mechanistic study S19
   5–1. NMR experiments S19
   5–2. Solid state structures of BHA **8** and 1:2 Cu/amide **2** complex (E) S20
   5–3. Time course study of the aldol reaction S21
   5–4. Computational analysis on the conformation of fluorinated ketones S24
6. References S25
7. Optimized coordinates S26
8. Spectra S28

1. General methods

1-1. Reactions and purifications

Unless otherwise noted, all reactions were carried out in an oven-dried glassware fitted with a 3-way glass stopcock under an argon atmosphere and were stirred with Teflon-coated magnetically stirred bars. All work-up and purification procedures were carried out with reagent-grade solvents under ambient atmosphere. Thin layer chromatography (TLC) was performed on Merck TLC plates (0.25 mm) pre-coated with silica gel 60 F254 and visualized by UV quenching and staining with ninhydrin, $KMnO_4$, anisaldehyde or ceric ammonium molybdate solution. Flash column chromatography was performed on a Teledyne CombiFlash Rf 200 or a Biotage Isolera Spektra One.

1-2. Chracterizations

Infrared (IR) spectra were recorded on a HORIBA FT210 Fourier transform infrared spectrophotometer. NMR spectra were recorded on a JEOL ECS-400, a Bruker AVANCE III HD400 or a Bruker AVANCE III 500. Chemical shifts (δ) are given in ppm relative to residual solvent peaks.[1] Data for ^{1}H NMR are reported as follows: chemical shift (multiplicity, coupling constants where applicable, number of hydrogens). Abbreviations are as follows: s (singlet), d (doublet), t (triplet), dd (doublet of doublet), dt (doublet of triplet), ddd (doublet of doublet of doublet), q (quartet), m (multiplet), br (broad). For ^{19}F NMR, chemical shifts were reported in the scale relative to $PhCF_3$ (δ –62.7680 ppm in $CDCl_3$) as an external reference. Single-crystal X-ray data were collected on a Rigaku R-AXIS RAPID II imaging plate area detector with graphite-monochromated Cu-Ka radiation. Optical rotation was measured using a 1 mL cell with a 1.0 dm path length on a JASCO polarimeter P-1030. High-resolution mass spectra (ESI TOF (+)) were measured on a Thermo Fisher Scientific LTQ Orbitrap XL.

1-3. Solvents and reagents

Powdered MS13X was activated by a heat gun under reduced pressure, and stored in a glove box. $CaSO_4$ was purchased from Sigma–Aldrich (Drierite™) and was ground into a powder before use. Anhydrous 1,4-dioxane and DME were purchased from commercial suppliers. THF, Et_2O, CPME, EtOAc, toluene, CH_2Cl_2, and CH_3CN were purified by passing through a solvent purification system (Glass Contour). All other starting materials were used as supplied by commercial vendors or prepared by the method described in the corresponding reference.

1-4. Computational investigations

All quantum chemical calculations were performed using the Gaussian 09 program.[2] Structural optimizations were conducted with very tight optimization parameters, and density functional theory (DFT) calculations employed an ultrafine integration grid (99 radial shells, 590 angular points). Frequency calculations confirmed the identity of geometry minima (no imaginary frequencies).

2. Preparation of substrates

All fluorinated ketones were prepared according to the known procedure[3] with a slight modification. CF_3 ketones **4a**,[4] **4c**,[5] **4f**,[6] **4g**,[6] and **11b**[7] were previously reported.

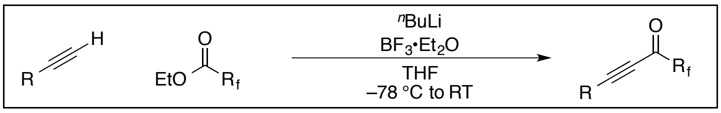

General procedure A: To a flame dried 500 mL flask equipped with a magnetically stirred chip, was added alkyne (1.0 equiv) and dry THF (0.1 M). The flask was cooled to –78 °C, and stirred for 30 min. *n*BuLi (hexane solution, 1.0 equiv) was added slowly down the side of the flask over 5 min. The solution was stirred for 30 min following the completion of *n*BuLi addition. To this solution, were slowly added fluorinated ethyl ester (1.1–1.2 equiv) and BF_3•Et_2O (1.2 equiv) directly into the solution. The solution was stirred for 12 h, during which time gradually warmed to RT. After the addition of sat aq NH_4Cl, aqueous phase was extracted with EtOAc (3x). The combined organic layers were washed with brine (2x), dried over Na_2SO_4, filtered, and removed under reduced pressure. The obtained material was purified by silica gel column chromatography (hexane/EtOAc).

5-((*tert*-Butyldimethylsilyl)oxy)-1,1,1-trifluoropent-3-yn-2-one (4b): Prepared by the general procedure A from

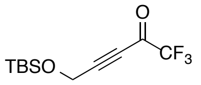

tert-butyldimethyl(2-propynyloxy)silane (3.0 mL, 14.8 mmol, 1.0 equiv), *n*BuLi (2.66 M in hexane, 5.56 mL, 14.8 mmol, 1.0 equiv), ethyl trifluoroacetate (1.94 mL, 16.3 mmol, 1.1 equiv), and BF_3•Et_2O (2.25 mL, 17.7 mmol, 1.2 equiv), purified by column chromatography (hexane/EtOAc) and isolated as a colorless oil (2.3 g, 58%). **IR** (thin film) 2957, 2933, 2860, 2208, 1716, 1218, 1132 cm^{-1}; **^{1}H NMR** (400 MHz, $CDCl_3$) δ 4.57 (s, 2H), 0.92 (s, 9H), 0.14 (s, 6H); **^{13}C NMR** (400 MHz, $CDCl_3$) δ 167.0 (q, *J* = 42.5 Hz), 114.7 (q, *J* = 288.3 Hz), 100.5, 79.0, 51.8, 25.7, 18.3, –5.2; **^{19}F NMR** (376 MHz, $CDCl_3$) δ –78.2; measurement of the exact mass has so far proven unsuccessful.

8-((*tert*-Butyldiphenylsilyl)oxy)-1,1,1-trifluorooct-3-yn-2-one (4d): Prepared by the general procedure A from

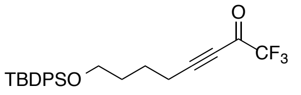

tert-butyl(hex-5-yn-1-yloxy)diphenylsilane[8] (7.24 g, 21.5 mmol, 1.0 equiv), *n*BuLi (2.66 M in hexane, 8.10 mL, 21.5 mmol, 1.0 equiv), ethyl trifluoroacetate (2.60 mL, 21.5 mmol, 1.0 equiv), and BF_3•Et_2O (3.20 mL, 25.8 mmol, 1.2 equiv), purified by column chromatography (hexane/EtOAc) and isolated as a colorless oil (5.4 g, 58%). **IR** (thin film) 2955, 2933, 2858, 2210, 1709, 1428, 1215, 1148, 1110 cm^{-1}; **^{1}H NMR** (400 MHz, $CDCl_3$) δ 7.72–7.70 (m, 4H), 7.52–7.38 (m, 6H), 3.74 (t, *J* = 5.8 Hz, 2H), 2.53 (t, *J* = 6.9 Hz, 2H), 1.89–1.77 (m, 2H), 1.75–1.69 (m, 2H), 1.11 (s, 9H); **^{13}C NMR** (100 MHz, $CDCl_3$) δ 167.3 (q, *J* = 41.9 Hz), 135.7, 133.9, 129.8, 127.8, 114.8 (q, *J* = 288.4 Hz), 105.2, 76.4, 63.1, 31.5, 27.0, 24.0, 19.3, 19.3; **^{19}F NMR** (376 MHz, $CDCl_3$) δ –78.2; the exact mass was measured after the treatment with $BnONH_2$•HCl in MeOH. **HRMS** (ESI): *m/z* calc'd for $C_{31}H_{34}O_2NF_3NaSi$ $[M + Na]^+$: 560.2203, found: 560.2203.

8-Chloro-1,1,1-trifluorooct-3-yn-2-one (4e): Prepared by the general procedure A from 6-chloro-1-hexyne (3.00 mL,

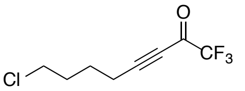

24.7 mmol, 1.0 equiv), *n*BuLi (2.66 M in hexane, 9.30 mL, 24.7 mmol, 1.0 equiv), ethyl trifluoroacetate (3.53 mL, 29.6 mmol, 1.2 equiv), and BF_3•Et_2O (3.89 mL, 29.6 mmol, 1.2 equiv), purified by column chromatography (hexane/EtOAc) and isolated as a colorless oil (3.7 g, 71%). **IR** (thin film) 2962, 2874, 2211, 1709, 1216, 1150 cm^{-1}; **^{1}H NMR** (400 MHz, $CDCl_3$) δ 3.58 (t, *J* = 6.1 Hz, 2H), 2.57 (t, *J* = 6.8 Hz, 2H), 1.99–1.88 (m, 2H), 1.88–1.77 (m, 2H); **^{13}C NMR** (100 MHz, $CDCl_3$) δ 167.2 (q, *J* = 42.1 Hz), 114.8 (q, *J* = 288.4 Hz), 103.9, 76.5, 44.1, 31.4, 24.6, 18.9; **^{19}F NMR** (376 MHz, $CDCl_3$) δ –78.3; measurement of the exact mass has so far proven unsuccessful.

4-(4-(1,3-Dioxolan-2-yl)phenyl)-1,1,1-trifluorobut-3-yn-2-one (4h): Prepared by the general procedure A from

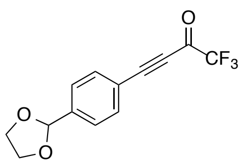

2-(4-ethynylphenyl)-1,3-dioxolane[9] (1.99 g, 11.4 mmol, 1.0 equiv), *n*BuLi (2.67 M in hexane, 4.27 mL, 11.4 mmol, 1.0 equiv), ethyl trifluoroacetate (1.63 mL, 13.7 mmol, 1.2 equiv), and BF_3•Et_2O (1.73 mL, 13.7 mmol, 1.2 equiv), purified by column chromatography (hexane/EtOAc) and isolated as a white solid (2.1 g, 70%). **m.p.** 37–38 °C; **IR** (thin film) 2950, 2854, 2201, 1703, 1199, 1086, 1045 cm^{-1}; **^{1}H NMR** (400 MHz, $CDCl_3$) δ 7.71–7.68 (m, 2H), 7.58–7.56 (m, 2H), 5.85 (s, 1H), 4.16–4.02 (m, 4H); **^{13}C NMR** (100 MHz, $CDCl_3$) δ 167.4 (q, *J* = 42.2 Hz), 142.9, 134.1, 127.2, 118.8, 115.0 (q, *J* = 286.6 Hz), 102.8, 100.1, 83.7, 65.6; **^{19}F NMR** (376 MHz, $CDCl_3$) δ –77.8; measurement of the exact mass has so far proven unsuccessful.

1,1,1-Trifluoro-4-(4-(morpholine-4-carbonyl)phenyl)but-3-yn-2-one hydrate (4i•H_2O): Prepared by the general

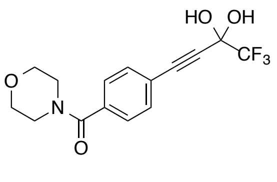

procedure A from (4-ethynylphenyl)(morpholino)methanone[10] (703 mg, 3.26 mmol, 1.0 equiv), *n*BuLi (2.67 M in hexane, 1.22 mL, 3.26 mmol, 1.0 equiv), ethyl trifluoroacetate (467 μL, 3.91 mmol, 1.2 equiv), and BF_3•Et_2O (496 μL, 3.91 mmol, 1.2 equiv), purified by column chromatography (hexane/EtOAc) to give a pale yellow oil of the corresponding trifluoromethyl ketone. The oil solidified while standing at RT, which was washed with $CHCl_3$ to afford the hydrate as a white solid (751 mg, 69%). **m.p.** 106–107 °C; **IR** (KBr) 3396, 2200, 1614, 1600, 1466, 1440, 1275, 1184, 1105, 994 cm^{-1}; **^{1}H NMR** (400 MHz, d_6-DMSO) δ 8.17 (s, 2H), 7.59–7.53 (m, 2H), 7.49–7.43 (m, 2H), 3.60 (brs, 8H); **^{13}C NMR** (100 MHz, d_6-DMSO) δ 168.1, 136.5, 131.7, 127.6, 122.1 (q, *J* = 285.6 Hz), 121.6, 86.6 (q, *J* = 34.8 Hz), 86.2, 82.7, 66.0, 47.5, 42.1; **^{19}F NMR** (376 MHz, d_6-DMSO) δ –84.4; the exact mass was measured after the treatment with $BnONH_2$•HCl in DMSO. **HRMS** (ESI): *m/z* calc'd for $C_{22}H_{20}O_3N_2F_3$ [M + H]$^+$: 417.1421, found: 417.1412.

1,1,1-Trifluoro-4-(thiophen-3-yl)but-3-yn-2-one (4j): Prepared by the general procedure A from 3-ethynylthiophene

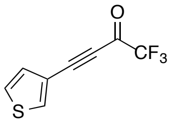

(2.00 g, 18.5 mmol, 1.0 equiv), *n*BuLi (2.66 M in hexane, 6.90 mL, 18.5 mmol, 1.0 equiv), ethyl trifluoroacetate (2.40 mL, 20.3 mmol, 1.1 equiv), and BF_3•Et_2O (2.80 mL, 22.2 mmol, 1.2 equiv), purified by column chromatography (hexane/EtOAc) and isolated as a colorless oil (2.4 g, 63%). **IR** (thin film) 3115, 2196, 1698, 1213, 1159, 1048 cm^{-1}; **^{1}H NMR** (400 MHz, $CDCl_3$) δ 7.97 (dd, *J* = 1.1, 2.9 Hz, 1H), 7.41 (dd, *J* = 2.9, 5.1 Hz, 1H), 7.31 (dd, *J* = 1.1, 5.1 Hz, 1H); **^{13}C NMR** (100 MHz, $CDCl_3$) δ 167.3 (q, *J* = 42.1 Hz), 137.4, 130.6, 127.1, 117.8, 115.0 (q, *J* = 286.6 Hz), 96.1, 84.2; **^{19}F NMR** (376 MHz, $CDCl_3$) δ –77.7; measurement of the exact mass has so far proven unsuccessful.

4-Cyclohexyl-1,1,1-trifluorobut-3-yn-2-one (4k): Prepared by the general procedure A from cyclohexylacetylene (2.16

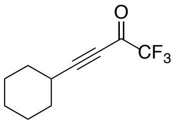

g, 19.9 mmol, 1.0 equiv), *n*BuLi (2.66 M in hexane, 7.52 mL, 19.9 mmol, 1.0 equiv), ethyl trifluoroacetate (2.86 mL, 23.9 mmol, 1.2 equiv), and BF_3•Et_2O (3.10 mL, 23.9 mmol, 1.2 equiv), purified by column chromatography (hexane/EtOAc) and isolated as a colorless oil (2.4 g, 59%). **IR** (thin film) 2938, 2860, 2207, 1710, 1214, 1153, 920 cm^{-1}; **^{1}H NMR** (400 MHz, $CDCl_3$) δ 2.69 (tt, *J* = 3.9, 8.5 Hz, 1H), 1.91–1.85 (m, 2H), 1.77–1.69 (m, 2H), 1.63–1.51 (m, 3H), 1.44–1.36 (m, 3H); **^{13}C NMR** (100 MHz, $CDCl_3$) δ 167.5 (q, *J* = 41.8 Hz), 114.9 (q, *J* = 288.5 Hz), 108.8, 76.3, 31.1, 29.6, 25.6, 24.5; **^{19}F NMR** (376 MHz, $CDCl_3$) δ –78.2; the exact mass was measured after the treatment with $BnONH_2$•HCl in MeOH. **HRMS** (ESI): *m/z* calc'd for $C_{17}H_{19}ONF_3$ [M + H]$^+$: 310.1413, found: 310.1415.

4-(Cyclohex-1-en-1-yl)-1,1,1-trifluorobut-3-yn-2-one (4l): Prepared by the general procedure A from

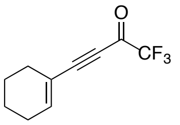

1-ethynylcyclohexene (3.00 g, 25.5 mmol, 1.0 equiv), *n*BuLi (2.66 M in hexane, 9.60 mL, 25.5 mmol, 1.0 equiv), ethyl trifluoroacetate (3.70 mL, 30.6 mmol, 1.2 equiv), and BF_3•Et_2O (3.98 mL, 30.6 mmol, 1.2 equiv), purified by column chromatography (hexane/EtOAc) and isolated as a colorless oil (3.8 g, 74%). **IR** (thin film) 2940, 2184, 1700, 1616, 1214, 1158, 1051, 1026 cm^{-1}; **^{1}H NMR** (400 MHz, $CDCl_3$) δ 6.73–6.71 (m, 1H), 2.25–2.21 (m, 4H), 1.76–1.57 (m, 4H); **^{13}C NMR** (100 MHz, $CDCl_3$) δ 167.4 (q, *J* = 41.7 Hz), 148.1, 118.5, 115.0 (q, *J* = 286.7 Hz), 103.4, 82.2, 27.8, 26.7, 21.8, 21.0; **^{19}F NMR** (376 MHz, $CDCl_3$) δ –77.9; measurement of the exact mass has so far proven unsuccessful.

1,1-Difluorodec-3-yn-2-one (11a): Prepared by the general procedure A from 1-octyne (3.5 mL, 23.7 mmol, 1.0 equiv),

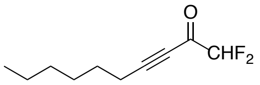

*n*BuLi (2.66 M in hexane, 8.9 mL, 23.7 mmol, 1.0 equiv), ethyl difluoroacetate (3.0 mL, 28.4 mmol, 1.2 equiv), and BF_3•Et_2O (3.6 mL, 28.4 mmol, 1.2 equiv), purified by column chromatography (hexane/EtOAc) and isolated as a colorless oil (3.1 g, 70%). **IR** (thin film) 2958, 2934, 2861, 2212, 1698, 1124, 1074 cm^{-1}; **^{1}H NMR** (400 MHz, $CDCl_3$) δ 5.73 (t, *J* = 54.3 Hz, 1H), 2.47 (t, *J* = 7.1 Hz, 2H), 1.68–1.56 (m, 2H), 1.50–1.38 (m, 2H), 1.34–1.27 (m, 4H), 0.90 (t, *J* = 6.9 Hz, 3H); **^{13}C NMR** (100 MHz, $CDCl_3$) δ 175.6 (t, *J* = 29.5 Hz), 108.9 (t, *J* = 252.7 Hz), 104.0, 77.1, 31.3, 28.6, 27.4, 22.6, 19.5, 14.1.; **^{19}F NMR** (376 MHz, $CDCl_3$) δ –125.8 (d, *J* = 54.3 Hz); the exact mass was measured after the treatment with $BnONH_2$•HCl in MeOH. **HRMS** (ESI): *m*/*z* calc'd for $C_{17}H_{22}ONF_2$ $[M + H]^+$: 294.1664, found: 294.1662.

1-Bromo-1,1-difluorodec-3-yn-2-one (11c): Prepared by the general procedure A from 1-octyne (3.5 mL, 23.7 mmol, 1.0

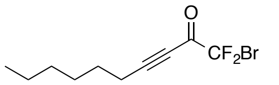

equiv), *n*BuLi (2.66 M in hexane, 8.9 mL, 23.7 mmol, 1.0 equiv), ethyl bromodifluoroacetate (3.7 mL, 28.4 mmol, 1.2 equiv), and BF_3•Et_2O (3.6 mL, 28.4 mmol, 1.2 equiv), purified by column chromatography (hexane/EtOAc) and isolated as a colorless oil (4.9 g, 77%). **IR** (thin film) 2958, 2933, 2861, 2212, 1709, 1160, 1129 cm^{-1}; **^{1}H NMR** (400 MHz, $CDCl_3$) δ 2.50 (t, *J* = 7.0 Hz, 2H), 1.71–1.60 (m, 2H), 1.47–1.41 (m, 2H), 1.39–1.21 (m, 4H), 0.90 (t, *J* = 6.9 Hz, 3H); **^{13}C NMR** (100 MHz, $CDCl_3$) δ 168.5 (t, *J* = 32.1 Hz), 113.0 (t, *J* = 316.9 Hz), 105.4, 75.0, 31.2, 28.6, 27.3, 22.6, 19.6, 14.1; **^{19}F NMR** (376 MHz, $CDCl_3$) δ –63.3; measurement of the exact mass has so far proven unsuccessful.

3. Direct catalytic asymmetric aldol reaction of α-N_3 amide to CF_3 ketones

3-1. Optimization study

General procedure B (Table 1): To a flame dried test tube equipped with a magnetically stirred chip was added amide **2** (20.3 mg, 0.1 mmol, 1.0 equiv) and ligand (0.012 mmol, 12 mol%). In a glove box, metal source (0.01 mmol, 10 mol%) and additive (500 % w/w) were added to the test tube. After it was taken out from the glove box, THF (0.35 mL, 0.28 M) and CF_3 ketone **4a** (29.8 μL, 0.105 mmol, 1.05 equiv) were added. The solution was stirred for 10 min at RT and 5 min at –40 °C before the addition of the solution of Barton's base (0.1 M in THF). The reaction was stirred for 6 h at the same temperature. After the addition of sat aq NH_4Cl at –40 °C, the solution was diluted with H_2O and EtOAc at RT and filtered through a pad of Celite. The aqueous phase was extracted with EtOAc (3x). The combined organic phases were dried over Na_2SO_4, filtered, and removed under reduced pressure.

General procedure C (Table S1): To a flame dried test tube equipped with a magnetically stirred chip was added amide **2** (20.3 mg, 0.1 mmol, 1.0 equiv) and BHA **8** (8.6 mg, 0.012 mmol, 12 mol%). In a glove box, $Cu(OTf)_2$ (3.7 mg, 0.01 mmol, 10 mol%) and MS13X (101 mg, 500 % w/w) were added to the test tube. After it was taken out from the glove box, solvent (0.35 mL, 0.28 M) and CF_3 ketone **4a** (29.8 μL, 0.105 mmol, 1.05 equiv) were added. The solution was stirred for 10 min at RT and 5 min at –40 °C before the addition of the solution of Barton's base (0.1 M, 100 μL, 0.01 mmol, 10 mol%). The reaction was stirred for 6 h at the same temperature. After the addition of sat aq NH_4Cl at –40 °C, the solution was diluted with H_2O and EtOAc at RT and filtered through a pad of Celite. The aqueous phase was extracted with EtOAc (3x). The combined organic phases were dried over Na_2SO_4, filtered, and removed under reduced pressure.

Table S1 Solvent Study

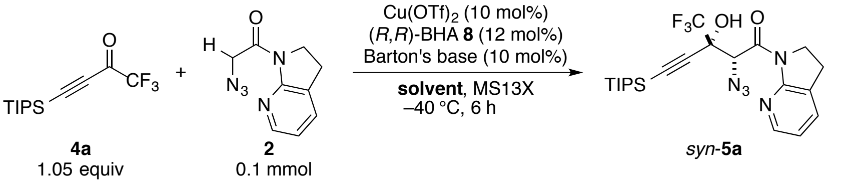

entry	**solvent**	yield[a] (%)	*anti/syn*[a]	ee[b] (%)
1	THF	78	22/78	93
2	Et_2O	3	42/58	71
3	1,4-dioxane	trace	nd	nd
4	CPME	trace	nd	nd
5	DME	40	24/76	95
6	toluene	trace	nd	nd
7	CH_2Cl_2	44	59/41	64
8	CH_3CN	9	57/43	76
9	EtOAc	38	31/69	91

[a]Yield and diastereomer ratio shown are from ^{1}H-NMR analysis on unpurified reaction mixture. [b]Enantiomeric excess was determined with normal phase HPLC on a chiral support. nd: not determined.

General procedure D (Table S2): To a flame dried test tube equipped with a magnetically stirred chip was added amide **2** (20.3 mg, 0.1 mmol, 1.0 equiv) and BHA **8** (8.6 mg, 0.012 mmol, 12 mol%). In a glove box, $Cu(OTf)_2$ (3.7 mg, 0.01 mmol, 10 mol%) and MS13X (101 mg, 500 % w/w) were added to the test tube. After it was taken out from the glove box, THF (0.35 mL, 0.28 M) and CF_3 ketone **4a** (29.8 μL, 0.105 mmol, 1.05 equiv) were added. The solution was stirred for 10 min at RT and 5 min at –40 °C before the addition of the solution of Brønsted base (0.1 M in THF, 100 μL, 0.01 mmol, 10 mol%). The reaction was stirred for 6 h at the same temperature. After the addition of sat aq NH_4Cl at –40 °C, the solution was diluted with H_2O and EtOAc at RT and filtered through a pad of Celite. The aqueous phase was extracted with EtOAc (3x). The combined organic phases were dried over Na_2SO_4, filtered, and removed under reduced pressure.

Table S2 Brønsted Base Effect

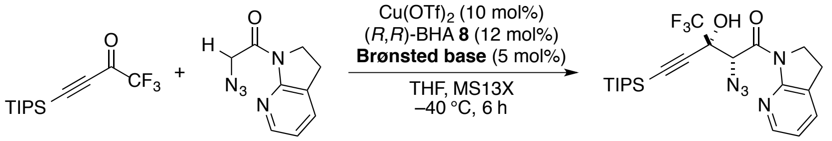

4a 1.2 equiv   **2** 0.1 mmol   *syn*-**5a**

entry	**Brønsted base**	yield[a] (%)	*anti*/*syn*[a]	ee[b] (*syn*)
1	Barton's base	93	17/83	96
2	$^{i}Pr_2NEt$	6	15/85	96
3	DBU	84	27/73	97
4	MTBD	85	22/78	97

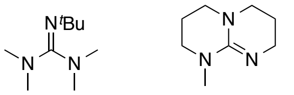

Barton's base   MTBD

[a]Yield and diastereomer ratio shown are from ^{1}H-NMR analysis on unpurified reaction mixture. [b]Enantiomeric excess was determined with normal phase HPLC on a chiral support.

3-2. Synthesis of BHA ***10***

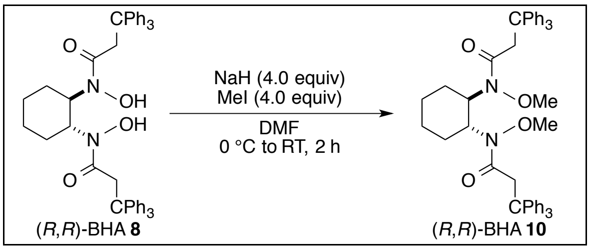

***N*,*N'*-((1*R*,2*R*)-cyclohexane-1,2-diyl)bis(*N*-methoxy-3,3,3-triphenylpropanamide) (10):** To a flame dried test tube equipped with a magnetically stirred chip was added BHA **8** (18 mg, 0.025 mmol, 1 equiv), NaH (60% in oil; 4 mg, 0.1 mmol, 4 equiv) and DMF (50 μL). The suspension was cooled to 0 °C and MeI (6 μL, 0.1 mmol, 4 equiv) was added. After 2 h, sat aq NH_4Cl and $CHCl_3$ were added. The aqueous phase was extracted with $CHCl_3$ (3x). The combined organic layers were washed with brine, dried over Na_2SO_4, filtered, and removed under reduced pressure. The obtained residue was purified by silica gel column chromatography eluting with hexane/EtOAc to give **10** (21 mg, 82%) as a colorless oil. **IR** (thin film) 3019, 1519, 1424, 1215, 757 cm^{-1}; **^{1}H NMR** (400 MHz, $CDCl_3$) δ 7.30–7.08 (m, 30H), 4.26 (brs, 2H), 4.01–3.97 (m, 2H), 3.73–3.68 (m, 2H), 3.29 (s, 6H), 1.57–1.45 (m, 6H), 1.17–0.99 (m, 2H); **^{13}C NMR** (100 MHz, $CDCl_3$) δ 174.5, 147.3, 129.5, 127.8, 126.0, 64.7, 56.6, 55.5, 43.8, 28.5, 24.8; **HRMS** (ESI): *m*/*z* calc'd for $C_{50}H_{51}O_4N_2$ [M + H]$^+$: 743.3843, found: 743.3841. $[\alpha]_D^{27}$ –6.0 (*c* 0.54, $CHCl_3$).

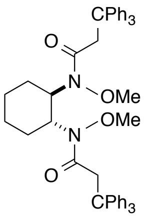

3-3. Control experiment

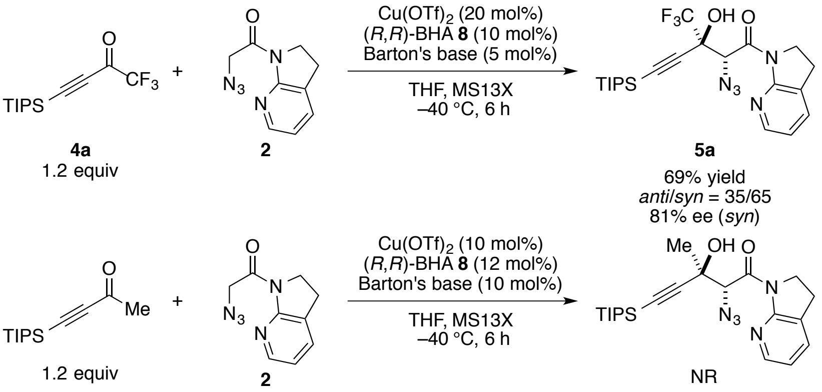

3-4. Substrate scope and limitations

General procedure E (Table 2 and 3): To a flame dried test tube equipped with a magnetically stirred chip was added amide **2** (1.0 equiv) and BHA **8** (12 mol%). In a glove box, $Cu(OTf)_2$ (10 mol%) and MS13X (500 % w/w) were added to the test tube. After it was taken out from the glove box, THF (0.28 M) and CF_3 ketone (1.2 equiv) were added. The solution was stirred for 10 min at RT and 5 min at –40 °C before the addition of the solution of Barton's base (0.1 M in THF, 5 or 10 mol%). After the addition of sat aq NH_4Cl at –40 °C, the solution was diluted with H_2O and EtOAc at RT and filtered through a pad of Celite. The aqueous phase was extracted with EtOAc (3x). The combined organic phases were dried over Na_2SO_4, filtered, and removed under reduced pressure.

(2*R*,3*S*)-2-Azido-1-(2,3-dihydro-1*H*-pyrrolo[2,3-*b*]pyridin-1-yl)-3-hydroxy-3-(trifluoromethyl)-5-(triisopropylsilyl)pent-4-yn-1-one (5a): Prepared by the general procedure E from amide **2** (40.6 mg, 0.20 mmol, 1.0 equiv), ketone **4a** (68.0 μL, 0.24 mmol, 1.2 equiv), Barton's base (0.1 M in THF, 100 μL, 10 μmol, 5 mol%), $Cu(OTf)_2$ (7.4 mg, 0.020 mmol, 10 mol%), and BHA **8** (17.2 mg, 0.024 mmol, 12 mol%), stirred for 6 h at –40 °C, purified by column chromatography (hexane/EtOAc), and isolated as a colorless oil (90.5 mg, 94%). **IR** (thin film) 3019, 2400, 2224, 1560, 1214, 771 cm^{-1}; **^{1}H NMR** (400 MHz, $CDCl_3$) δ 8.12–8.06 (m, 1H), 7.59 (dd, *J* = 1.4, 7.5 Hz, 1H), 7.07–6.98 (m, 2H), 5.80 (s, 1H), 4.30 (ddd, *J* = 5.7, 10.0, 12.3 Hz, 1H), 4.13 (ddd, *J* = 7.6, 10.0, 12.3 Hz, 1H), 3.24–3.06 (m, 2H), 1.14–1.11 (m, 21H); **^{13}C NMR** (100 MHz, $CDCl_3$) δ 166.5, 154.7, 145.7, 135.1, 127.1, 123.5 (q, *J* = 286.1 Hz), 119.9, 98.1, 93.0, 74.6 (q, *J* = 30.6 Hz), 57.8, 46.7, 24.5, 18.6, 11.2; **^{19}F NMR** (376 MHz, $CDCl_3$) δ –79.0; **HRMS** (ESI): *m*/*z* calc'd for $C_{22}H_{31}O_2N_5F_3Si$ $[M + H]^+$: 482.2194, found: 482.2187. $[\alpha]_D^{26}$ –49.6 (*c* 0.27, $CHCl_3$, 94% ee sample); HPLC analysis (CHIRALPAK AD-3 (ϕ = 0.46 cm x 25 cm), 2 propanol/*n*-hexane = 1/19, flow rate = 1.0 mL/min, detection at 254 nm, t_R = 6.4 min (minor), 8.0 min (major)):

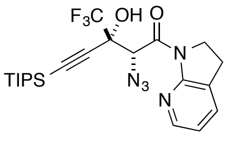

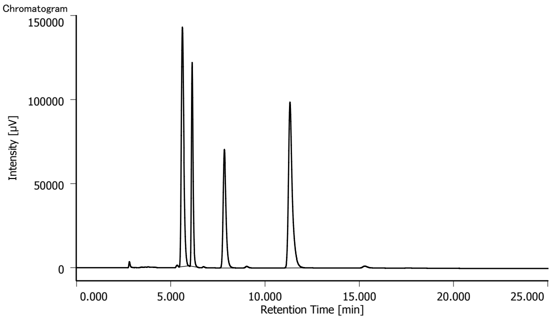

tR [min]	Area%
5.617	31.935
6.133	17.607
7.842	17.941
11.325	32.518

Racemic sample

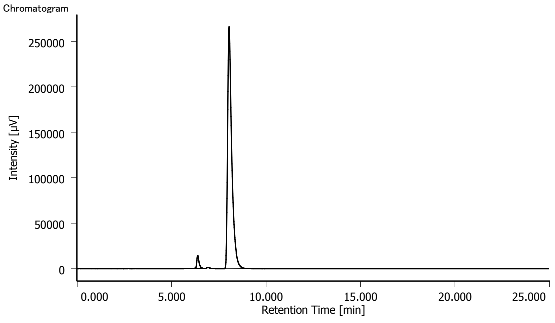

tR [min]	Area%
6.383	2.918
8.042	97.082

Reaction sample

(2*R*,3*S*)-2-Azido-6-((*tert*-butyldimethylsilyl)oxy)-1-(2,3-dihydro-1*H*-pyrrolo[2,3-*b*]pyridin-1-yl)-3-hydroxy-3-(trifluoromethyl)hex-4-yn-1-one (5b):

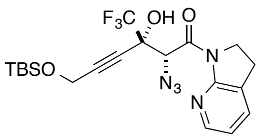

Prepared by the general procedure E from amide **2** (40.6 mg, 0.20 mmol, 1.0 equiv), ketone **4b** (62.8 μL, 0.24 mmol, 1.2 equiv), Barton's base (0.1 M in THF, 100 μL, 10 μmol, 5 mol%), Cu(OTf)$_2$ (7.4 mg, 0.020 mmol, 10 mol%), and BHA **8** (17.2 mg, 0.024 mmol, 12 mol%), stirred for 6 h at –40 °C, purified by column chromatography (hexane/EtOAc), and isolated as a colorless oil (80.7 mg, 86%). **IR** (thin film) 3019, 2399, 1429, 1215, 767 cm^{-1}; **^{1}H NMR** (400 MHz, CDCl$_3$) δ 8.14–8.07 (m, 1H), 7.60 (dd, *J* = 1.5, 7.4 Hz, 1H), 7.04 (dd, *J* = 5.2, 7.4 Hz, 1H), 6.96 (s, 1H), 5.85 (s, 1H), 4.46 (s, 2H), 4.29 (ddd, *J* = 5.8, 9.9, 12.4 Hz, 1H), 4.12 (ddd, *J* = 7.7, 9.9, 12.4 Hz, 1H), 3.24–3.07 (m, 2H), 0.92 (s, 9H), 0.15 (s, 6H); **^{13}C NMR** (100 MHz, CDCl$_3$) δ 166.5, 154.5, 145.8, 135.1, 127.0, 123.5 (q, *J* = 287.6 Hz), 120.0, 88.6, 76.4, 74.7 (q, *J* = 30.8 Hz), 57.8, 51.8, 46.6, 25.9, 24.5, 18.4, –5.2; **^{19}F NMR** (376 MHz, CDCl$_3$) δ –78.7; **HRMS** (ESI): *m/z* calc'd for $C_{20}H_{27}O_3N_5F_3Si$ [M + H]$^+$: 470.1830, found: 470.1830. $[\alpha]_D^{27}$ –90.3 (*c* 0.60, CHCl$_3$, 94% ee sample); HPLC analysis (CHIRALPAK IA (ϕ = 0.46 cm x 25 cm), 2 propanol/*n*-hexane = 1/19, flow rate = 1.0 mL/min, detection at 254 nm, t_R = 8.2 min (minor), 9.6 min (major)):

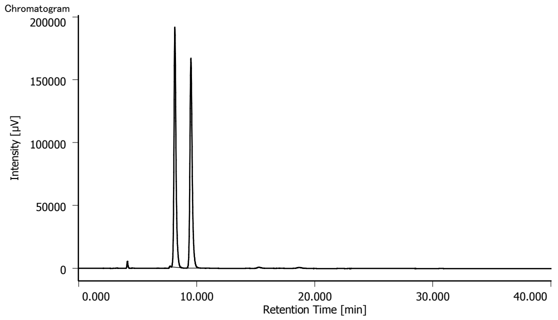

tR [min]	Area%
8.158	50.124
9.517	49.876

Racemic sample

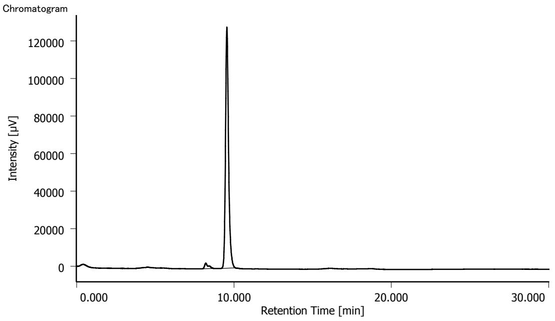

tR [min]	Area%
8.208	2.798
9.550	97.202

Reaction sample

(2*R*,3*S*)-2-Azido-1-(2,3-dihydro-1*H*-pyrrolo[2,3-*b*]pyridin-1-yl)-3-hydroxy-3-(trifluoromethyl)undec-4-yn-1-one (5c):

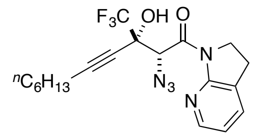

Prepared by the general procedure E from amide **2** (40.6 mg, 0.20 mmol, 1.0 equiv), ketone **4c** (49.0 μL, 0.24 mmol, 1.2 equiv), Barton's base (0.1 M in THF, 200 μL, 20 μmol, 10 mol%), Cu(OTf)$_2$ (7.4 mg, 0.020 mmol, 10 mol%), and BHA **8** (17.2 mg, 0.024 mmol, 12 mol%), stirred for 6 h at –40 °C, purified by column chromatography (hexane/EtOAc), and isolated as a colorless oil (75.3 mg, 92%). **IR** (thin film) 3019, 2399, 1523, 1425, 1215, 757 cm^{-1}; **^{1}H NMR** (400 MHz, CDCl$_3$) δ 8.13–8.04 (m, 1H), 7.63–7.56 (m, 1H), 7.08–6.96 (m, 2H), 5.67 (s, 1H), 4.32 (ddd, *J* = 5.3, 10.1, 12.3 Hz, 1H), 4.12 (ddd, *J* = 7.8, 10.1, 12.3 Hz, 1H), 3.24–3.05 (m, 2H), 2.33 (t, *J* = 7.2 Hz, 2H), 1.64–1.57 (m, 2H), 1.50–1.38 (m, 2H), 1.38–1.24 (m, 4H), 0.89 (t, *J* = 6.8 Hz, 3H); **^{13}C NMR** (100 MHz, CDCl$_3$) δ 166.6, 154.7, 145.6, 135.1, 127.2, 123.7 (q, *J* = 284.9 Hz), 119.9, 91.8, 74.5 (q, *J* = 30.6 Hz), 71.9, 57.9, 46.8, 31.4, 28.6, 27.9, 24.6, 22.7, 19.0, 14.2; **^{19}F NMR** (376 MHz, CDCl$_3$) δ –79.2; **HRMS** (ESI): *m/z* calc'd for $C_{19}H_{23}O_2N_5F_3$ [M + H]$^+$: 410.1798, found: 410.1797. $[\alpha]_D^{27}$ –56.2 (*c* 0.57, CHCl$_3$, 96% ee sample); HPLC analysis (CHIRALPAK AD-3 (ϕ = 0.46 cm x 25 cm), 2 propanol/*n*-hexane = 1/19, flow rate = 1.0 mL/min, detection at 254 nm, t_R = 18.8 min (minor), 20.8 min (major)):

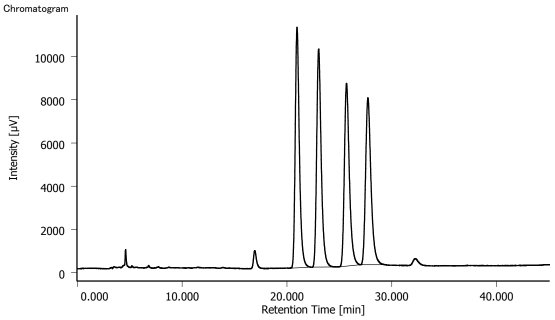

tR [min]	Area%
20.958	26.194
23.017	26.074
25.675	23.935
27.708	23.797

Racemic sample

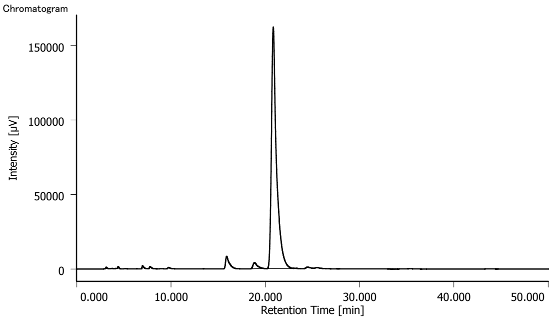

tR [min]	Area%
18.842	2.081
20.833	97.919

Reaction sample

(2*R*,3*S*)-2-Azido-9-((*tert*-butyldiphenylsilyl)oxy)-1-(2,3-dihydro-1*H*-pyrrolo[2,3-*b*]pyridin-1-yl)-3-hydroxy-3-(trifluoromethyl)non-4-yn-1-one (5d):

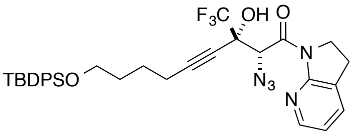

Prepared by the general procedure E from amide **2** (40.6 mg, 0.20 mmol, 1.0 equiv), ketone **4d** (96.2 μL, 0.24 mmol, 1.2 equiv), Barton's base (0.1 M in THF, 200 μL, 20 μmol, 10 mol%), $Cu(OTf)_2$ (7.4 mg, 0.020 mmol, 10 mol%), and BHA **8** (17.2 mg, 0.024 mmol, 12 mol%), stirred for 6 h at –40 °C, purified by column chromatography (hexane/EtOAc), and isolated as a colorless oil (120.8 mg, 95%). **IR** (thin film) 3019, 2399, 1421, 1215, 758 cm^{-1}; **^{1}H NMR** (400 MHz, $CDCl_3$) δ 8.17–8.10 (m, 1H), 7.67–7.59 (m, 4H), 7.56 (dd, *J* = 1.5, 7.4 Hz, 1H), 7.45–7.30 (m, 6H), 6.99 (dd, *J* = 5.1, 7.4 Hz, 1H), 5.98 (s, 2H), 4.11 (dd, *J* = 7.8, 9.2 Hz, 2H), 3.67 (t, *J* = 6.0 Hz, 2H), 3.15 (ddt, *J* = 1.2, 7.8, 9.7 Hz, 2H), 3.06–2.94 (m, 2H), 1.81–1.67 (m, 2H), 1.66–1.57 (m, 2H), 1.00 (s, 9H); **^{13}C NMR** (100 MHz, $CDCl_3$) δ 163.5, 155.3, 147.7, 146.5, 138.0, 135.7, 134.3, 133.9, 129.8, 127.8, 125.9, 119.3, 116.4 (q, *J* = 289.6 Hz), 76.3 (q, *J* = 30.0 Hz), 63.3, 52.3, 45.8, 32.3, 27.0, 24.7, 24.4, 23.7, 19.3; **^{19}F NMR** (376 MHz, $CDCl_3$) δ –74.1; **HRMS** (ESI): *m*/*z* calc'd for $C_{33}H_{37}O_3N_5F_3Si$ $[M + H]^+$: 636.2612, found: 636.2600. $[\alpha]_D^{25}$ 3.0 (*c* 0.19, $CHCl_3$, 94% ee sample); HPLC analysis (CHIRALCEL OD-3 (ϕ = 0.46 cm x 25 cm), 2 propanol/*n*-hexane = 1/19, flow rate = 1.0 mL/min, detection at 254 nm, t_R = 12.9 min (major), 14.3 min (minor)):

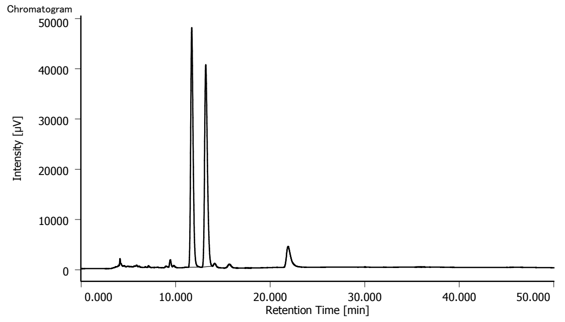

tR [min]	Area%
11.708	50.476
13.192	49.524

Racemic sample

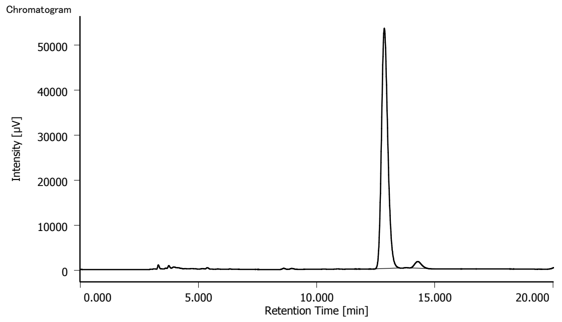

tR [min]	Area%
12.858	97.052
14.275	2.948

Reaction sample

(2*R*,3*S*)-2-Azido-9-chloro-1-(2,3-dihydro-1*H*-pyrrolo[2,3-*b*]pyridin-1-yl)-3-hydroxy-3-(trifluoromethyl)non-4-yn-1-one (5e):

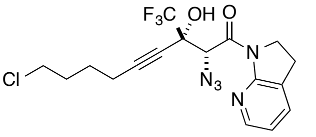

Prepared by the general procedure E from amide **2** (40.6 mg, 0.20 mmol, 1.0 equiv), ketone **4e** (42.2 μL, 0.24 mmol, 1.2 equiv), Barton's base (0.1 M in THF, 200 μL, 20 μmol, 10 mol%), $Cu(OTf)_2$ (7.4 mg, 0.020 mmol, 10 mol%), and BHA **8** (17.2 mg, 0.024 mmol, 12 mol%), stirred for 6 h at –40 °C, purified by column chromatography (hexane/EtOAc), and isolated as a pale yellow oil (73.1 mg, 88%). **IR** (thin film) 3019, 2399, 1521, 1427, 1215, 1046, 928, 756 cm^{-1}; **^{1}H NMR** (400 MHz, $CDCl_3$) δ 8.09 (dd, *J* = 1.8, 5.2 Hz, 1H), 7.60 (dd, *J* = 1.4, 7.4 Hz, 1H), 7.13 (s, 1H), 7.07–7.01 (m, 1H), 5.63 (s, 1H), 4.34 (ddd, *J* = 5.1, 10.1, 12.3 Hz, 1H), 4.13 (ddd, *J* = 7.9, 10.3, 12.3 Hz, 1H), 3.59 (t, *J* = 6.5 Hz, 2H), 3.28–3.05 (m, 2H), 2.40 (t, *J* = 6.9 Hz, 2H), 2.01–1.92 (m, 2H), 1.81–1.75 (m, 2H); **^{13}C NMR** (100 MHz, $CDCl_3$) δ 166.4, 154.7, 145.6, 135.2, 134.6, 127.2, 123.7 (q, *J* = 289.4 Hz), 120.0, 90.7, 74.4 (q, *J* = 30.4 Hz), 72.8, 57.8, 46.8, 44.7, 31.5, 25.0, 24.6, 18.3; **^{19}F NMR** (376 MHz, $CDCl_3$) δ –79.2; **HRMS** (ESI): *m*/*z* calc'd for $C_{17}H_{18}O_2N_5ClF_3$ $[M + H]^+$: 416.1096, found: 416.1097. $[\alpha]_D^{27}$ –111.6 (*c* 0.06, $CHCl_3$, 96% ee sample); HPLC analysis (CHIRALCEL OZ-H (ϕ = 0.46 cm x 25 cm), 2 propanol/*n*-hexane = 1/9, flow rate = 1.0 mL/min, detection at 254 nm, t_R = 11.4 min (minor), 15.1 min (major)):

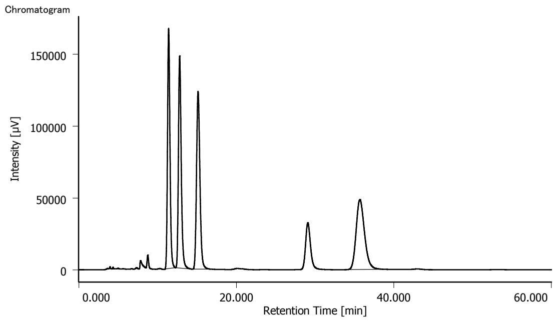

tR [min]	Area%
11.417	24.768
12.808	24.933
15.150	25.426
35.675	24.873

Racemic sample

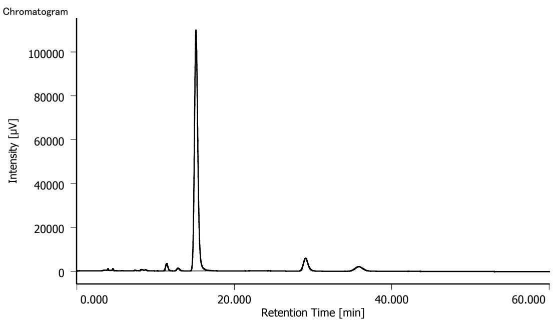

tR [min]	Area%
11.425	2.112
15.142	97.888

Reaction sample

(2*R*,3*S*)-2-Azido-1-(2,3-dihydro-1*H*-pyrrolo[2,3-*b*]pyridin-1-yl)-3-hydroxy-5-(*p*-tolyl)-3-(trifluoromethyl)pent-4-yn-1-one (5f): Prepared by the general procedure E from amide **2** (406 mg, 0.20 mmol, 1.0 equiv), ketone **4f** (342 μL, 0.24 mmol, 1.2 equiv), Barton's base (0.1 M in THF, 1.0 mL, 0.10 mmol, 5 mol%), Cu(OTf)$_2$ (36.1 mg, 0.10 mmol, 5 mol%), and BHA **8** (89.4 mg, 0.12 mmol, 6 mol%), stirred for 6 h at –40 °C, purified by column chromatography (hexane/EtOAc), and isolated as a pale brown oil (756 mg, 91%). **IR** (thin film) 3357, 2944, 2831, 2399, 1450, 1417, 1027 cm^{-1}; **^{1}H NMR** (400 MHz, CDCl$_3$) δ 8.20 (dd, *J* = 1.7, 5.1 Hz, 1H), 7.54 (dd, *J* = 1.3, 7.5 Hz, 1H), 7.10–6.97 (m, 5H), 6.52 (s, 1H), 5.46 (s, 1H), 4.21–4.17 (m, 2H), 3.22–2.94 (m, 2H), 2.31 (s, 3H); **^{13}C NMR** (100 MHz, CDCl$_3$) δ 167.3, 154.5, 146.7, 139.9, 134.5, 131.8, 129.2, 126.4, 122.8 (q, *J* = 284.0 Hz), 119.9, 117.8, 88.9, 81.8, 74.0 (q, *J* = 26.8 Hz), 59.3, 45.9, 24.2, 21.7; **^{19}F NMR** (376 MHz, CDCl$_3$) δ –77.6; **HRMS** (ESI): *m*/*z* calc'd for $C_{20}H_{16}O_2N_5F_3Na$ [M + Na]$^+$: 438.1148, found: 438.1139. $[\alpha]_D^{27}$ 3.2 (*c* 0.07, CHCl$_3$, 90% ee sample); HPLC analysis (CHIRALPAK AD-3 (ϕ = 0.46 cm x 25 cm), 2 propanol/*n*-hexane = 1/9, flow rate = 1.0 mL/min, detection at 254 nm, t_R = 17.6 min (major), 21.8 min (minor)):

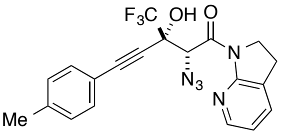

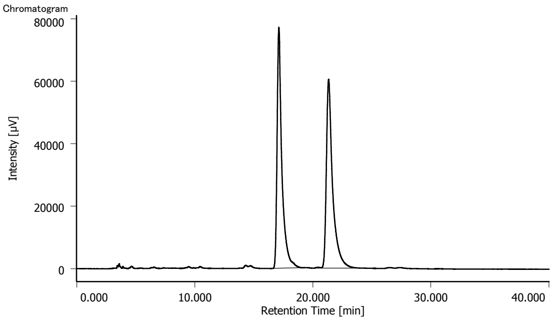

tR [min]	Area%
17.108	49.935
21.333	50.065

Racemic sample

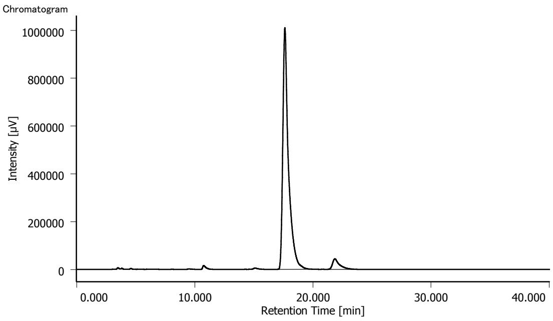

tR [min]	Area%
17.625	94.875
21.850	5.125

Reaction sample

(2*R*,3*S*)-2-Azido-5-(4-chlorophenyl)-1-(2,3-dihydro-1*H*-pyrrolo[2,3-*b*]pyridin-1-yl)-3-hydroxy-3-(trifluoromethyl)pent-4-yn-1-one (5g): Prepared by the general procedure E from amide **2** (40.6 mg, 0.20 mmol, 1.0 equiv), ketone **4g** (55.8 mg, 0.24 mmol, 1.2 equiv), Barton's base (0.1 M in THF, 200 μL, 20 μmol, 10 mol%), Cu(OTf)$_2$ (7.4 mg, 0.020 mmol, 10 mol%), and BHA **8** (17.2 mg, 0.024 mmol, 12 mol%), stirred for 6 h at –40 °C, purified by column chromatography (hexane/EtOAc), and isolated as a pale brown solid (83.6 mg, 96%). **m.p.** 154–155 °C; **IR** (thin film) 3019, 2399, 1214, 758 cm^{-1}; **^{1}H NMR** (400 MHz, CDCl$_3$) δ 8.11 (dd, *J* = 1.7, 5.2 Hz, 1H), 7.62 (dd, *J* = 1.4, 7.4 Hz, 1H), 7.56–7.50 (m, 2H), 7.38 (s, 1H), 7.35–7.29 (m, 2H), 7.06 (dd, *J* = 5.2, 7.4 Hz, 1H), 5.72 (s, 1H), 4.36 (ddd, *J* = 5.0, 10.2, 12.3 Hz, 1H), 4.15 (ddd, *J* = 8.0, 10.2, 12.3 Hz, 1H), 3.29–3.05 (m, 2H); **^{13}C NMR** (100 MHz, CDCl$_3$) δ 166.2, 154.5, 145.4, 135.6, 135.2, 133.5, 128.7, 127.2, 123.5 (q, *J* = 288.9 Hz), 120.0, 119.6, 88.4, 81.3, 74.8 (q, *J* = 31.1 Hz), 57.7, 46.8, 24.5; **^{19}F NMR** (376 MHz, CDCl$_3$) δ –78.8; **HRMS** (ESI): *m*/*z* calc'd for $C_{19}H_{14}O_2N_5ClF_3$ [M + H]$^+$: 436.0783, found: 436.0782. $[\alpha]_D^{26}$ –32.0 (*c* 0.23, CHCl$_3$, 95% ee sample); HPLC analysis (CHIRALCEL OZ-H (ϕ = 0.46 cm x 25 cm), 2 propanol/*n*-hexane = 1/19, flow rate = 1.0 mL/min, detection at 254 nm, t_R = 14.9 min (minor), 23.4 min (major)):

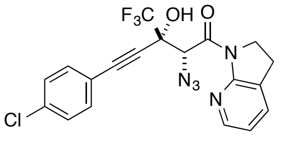

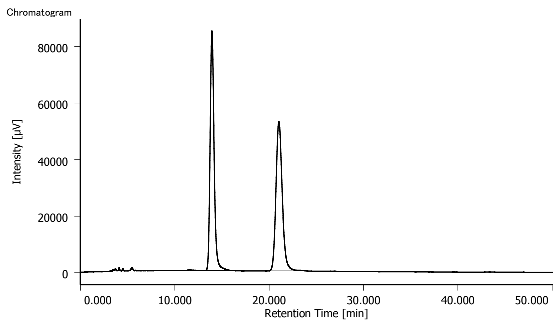

tR [min]	Area%
13.933	50.099
21.017	49.901

Racemic sample

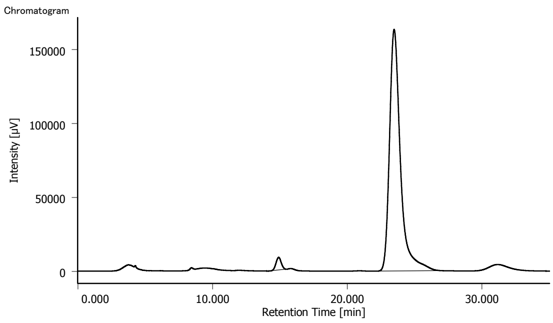

tR [min]	Area%
14.892	2.467
23.458	97.533

Reaction sample

Single crystals of **5g** were obtained by slow diffusion of hexanes to the solution of **5g** in $CHCl_3$ at RT. A suitable crystal was selected and the sample was measured on a Rigaku R-AXIS RAPID diffractometer using graphite monochromated Cu-Ka radiation. The data were collected at 93 K. Refined structure and crystallographic parameters are summarized in Fig. S1 and Table S3. CCDC 1498996 contains the supplementary crystallographic data for **5g**.

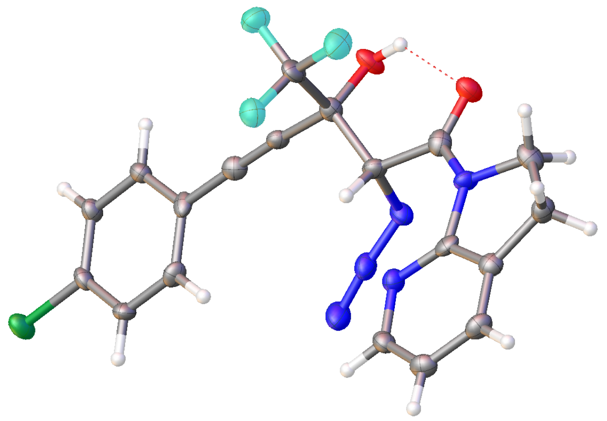

Fig. S1 ORTEP diagram of **5g**.

Table S3 Selected crystal data of **5g.**

Empirical Formula	$C_{19}H_{13}ClF_3N_5O_2$
Formula Weight	435.79
Crystal Color, Habit	colorless, platelet
Crystal Dimensions	0.200 x 0.200 x 0.100 mm
Crystal System	orthorhombic
Lattice Parameters	
a	10.7343(3) Å
b	12.0336(3) Å
c	28.9712(7) Å
V	3742.28(17) Å^3
Space Group	$P2_12_12_1$ (#19)
Z value	8
D_{calc}	1.547 g/cm^3
F_{000}	1776.00

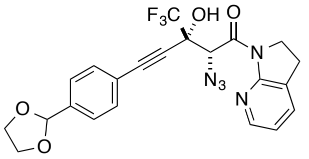

(2*R*,3*S*)-5-(4-(1,3-Dioxolan-2-yl)phenyl)-2-azido-1-(2,3-dihydro-1*H*-pyrrolo[2,3-*b*]pyridin-1-yl)-3-hydroxy-3-(trifluoromethyl)pent-4-yn-1-one (5h): Prepared by the general procedure E from amide **2** (40.6 mg, 0.20 mmol, 1.0 equiv), ketone **4h** (64.9 mg, 0.24 mmol, 1.2 equiv), Barton's base (0.1 M in THF, 200 μL, 20 μmol, 10 mol%), $Cu(OTf)_2$ (7.4 mg, 0.020 mmol, 10 mol%), and BHA **8** (17.2 mg, 0.024 mmol, 12 mol%), stirred for 6 h at –40 °C, purified by column chromatography (hexane/EtOAc), and isolated as a pale yellow oil (77.6 mg, 94%). **IR** (thin film) 3364, 2945, 2832, 1449, 1418, 1027 cm^{-1}; ^{1}**H NMR** (400 MHz, $CDCl_3$) δ 8.11 (dd, *J* = 1.8, 5.2 Hz, 1H), 7.65–7.58 (m, 3H), 7.50–7.43 (m, 2H), 7.32 (s, 1H), 7.05 (dd, *J* = 5.2, 7.4 Hz, 1H), 5.83 (s, 1H), 5.77 (s, 1H), 4.35 (ddd, *J* = 5.2, 10.1, 12.3 Hz, 1H), 4.21–3.99 (m, 5H), 3.29–3.06 (m, 2H); ^{13}**C NMR** (100 MHz, $CDCl_3$) δ 166.4, 154.6, 145.6, 139.4, 135.3, 132.5, 127.3, 123.6 (q, *J* = 289.2 Hz), 122.0, 120.0, 103.3, 89.4, 81.0, 75.1 (q, *J* = 30.9 Hz), 65.4, 58.0, 46.9, 24.6; ^{19}**F NMR** (376 MHz, $CDCl_3$) δ –78.7; **HRMS** (ESI): *m*/*z* calc'd for $C_{22}H_{19}O_4N_5F_3$ [M + H]$^+$: 474.1384, found: 474.1383. $[\alpha]_D^{27}$ –103.9 (*c* 0.16, $CHCl_3$, 91% ee sample); HPLC analysis (CHIRALCEL OZ–H (ϕ = 0.46 cm x 25 cm), 2 propanol/*n*-hexane = 1/9, flow rate = 1.0 mL/min, detection at 254 nm, t_R = 25.9 min (minor), 39.7 min (major)):

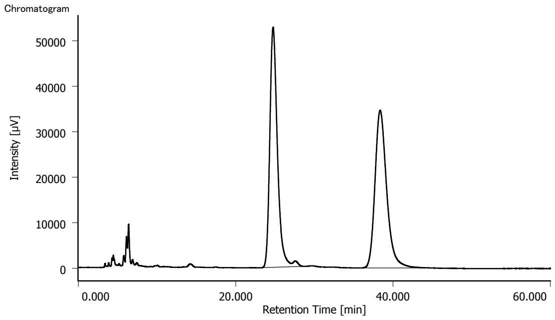

tR [min]	Area%
24.750	49.996
38.350	50.004

Racemic sample

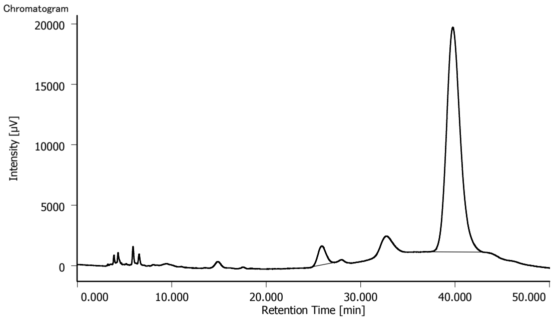

tR [min]	Area%
25.908	4.734
39.750	95.266

Reaction sample

(2*R*,3*S*)-2-Azido-1-(2,3-dihydro-1*H*-pyrrolo[2,3-*b*]pyridin-1-yl)-3-hydroxy-5-(4-(morpholine-4-carbonyl)phenyl)-3-(trifluoromethyl)pent-4-yn-1-one (5i):

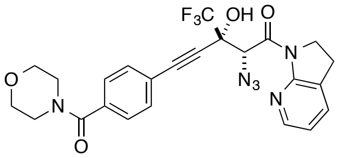

CF_3 ketone **4i** hydrate (79.0 mg, 0.24 mmol, 1.2 equiv) was treated with $CaSO_4$ (316 mg, 400% w/w) in dry toluene (4.0 mL) under reflux for 24 h. After cooled to RT, the suspension was filtered through celite, and the filtrate was removed under reduced pressure. The obtained ketone was subjected to the catalytic conditions described in General Procedure E, with amide **2** (40.6 mg, 0.20 mmol, 1.0 equiv), Barton's base (0.1 M in THF, 200 µL, 20 µmol, 10 mol%), $Cu(OTf)_2$ (7.4 mg, 0.020 mmol, 10 mol%), and BHA **8** (17.2 mg, 0.024 mmol, 12 mol%). The reaction was stirred for 6 h at –40 °C, and the crude material was purified by column chromatography (hexane/EtOAc) to give **5i** as a pale brown oil (95.6 mg, 93%). **IR** (thin film) 3347, 2944, 2831, 1449, 1029, 757 cm^{-1}; **^{1}H NMR** (400 MHz, $CDCl_3$) δ 8.10 (dd, *J* = 1.8, 5.3 Hz, 1H), 7.67–7.61 (m, 3H), 7.50–7.33 (m, 3H), 7.05 (dd, *J* = 5.3, 7.5 Hz, 1H), 5.71 (s, 1H), 4.36 (ddd, *J* = 4.9, 10.2, 12.3 Hz, 1H), 4.22–4.07 (m, 1H), 3.99–3.56 (m, 6H), 3.42 (brs, 2H), 3.27–3.03 (m, 2H); **^{13}C NMR** (100 MHz, $CDCl_3$) δ 169.7, 166.2, 154.6, 145.5, 136.2, 135.4, 132.6, 127.3, 127.3, 123.6 (q, *J* = 285.9 Hz), 122.9, 120.1, 88.7, 82.0, 75.0 (q, *J* = 31.1 Hz), 67.0, 57.9, 48.2, 46.9, 42.6, 24.6; **^{19}F NMR** (376 MHz, $CDCl_3$) δ –78.7; **HRMS** (ESI): *m/z* calc'd for $C_{24}H_{22}O_4N_6F_3$ $[M + H]^+$: 515.1649, found: 515.1645. $[\alpha]_D^{26}$ –46.4 (*c* 0.70, $CHCl_3$, 92% ee sample); HPLC analysis (CHIRALPAK IA (ϕ = 0.46 cm x 25 cm), 2 propanol/*n*-hexane = 1/4, flow rate = 1.0 mL/min, detection at 254 nm, t_R = 31.2 min (major), 46.1 min (minor)):

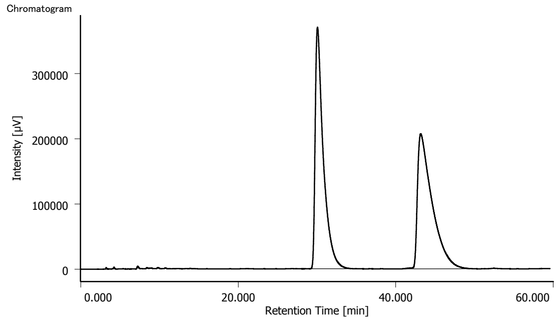

tR [min]	Area%
30.067	49.706
43.167	50.294

Racemic sample

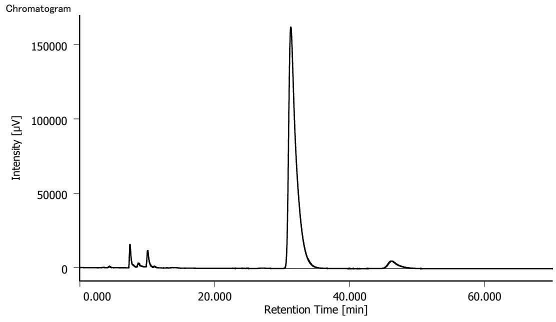

tR [min]	Area%
31.275	96.011
46.150	3.989

Reaction sample

(2*R*,3*S*)-2-Azido-1-(2,3-dihydro-1*H*-pyrrolo[2,3-*b*]pyridin-1-yl)-3-hydroxy-5-(thiophen-3-yl)-3-(trifluoromethyl)pent-4-yn-1-one (5j):

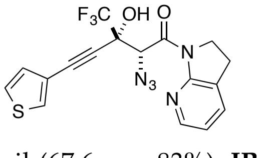

Prepared by the general procedure E from amide **2** (40.6 mg, 0.20 mmol, 1.0 equiv), ketone **4j** (34.0 µL, 0.24 mmol, 1.2 equiv), Barton's base (0.1 M in THF, 100 µL, 10 µmol, 5 mol%), $Cu(OTf)_2$ (7.4 mg, 0.020 mmol, 10 mol%), and BHA **8** (17.2 mg, 0.024 mmol, 12 mol%), stirred for 6 h at –40 °C, purified by column chromatography (hexane/EtOAc), and isolated as a pale brown oil (67.6 mg, 83%). **IR** (thin film) 3347, 2944, 2831, 2399, 1449, 1418, 1028, 757 cm^{-1}; **^{1}H NMR** (400 MHz, $CDCl_3$) δ 8.11 (dd, *J* = 1.5, 5.2 Hz, 1H), 7.66 (dd, *J* = 1.1, 3.0 Hz, 1H), 7.64–7.57 (m, 1H), 7.33–7.21 (m, 3H), 7.05 (dd, *J* = 5.2, 7.5 Hz, 1H), 5.76 (s, 1H), 4.35 (ddd, *J* = 5.1, 10.1, 12.3 Hz, 1H), 4.14 (ddd, *J* = 7.9, 10.1, 12.3 Hz, 1H), 3.29–3.05 (m, 2H); **^{13}C NMR** (100 MHz, $CDCl_3$) δ 166.4, 154.7, 145.6, 135.3, 131.3, 130.1, 127.3, 125.5, 123.6 (q, *J* = 285.8 Hz), 120.3, 120.1, 85.1, 80.3, 75.2 (q, *J* = 30.9 Hz), 57.9, 46.9, 24.6; **^{19}F NMR** (376 MHz, $CDCl_3$) δ –78.8; **HRMS** (ESI): *m/z* calc'd for $C_{17}H_{12}O_2N_5F_3NaS$ $[M + Na]^+$:

430.0556, found: 430.0555. $[\alpha]_D^{28}$ –46.2 (*c* 0.18, $CHCl_3$, 91% ee sample); HPLC analysis (CHIRALPAK IC (ϕ = 0.46 cm x 25 cm), 2 propanol/*n*-hexane = 1/9, flow rate = 1.0 mL/min, detection at 254 nm, t_R = 14.9 min (major), 16.9 min (minor)):

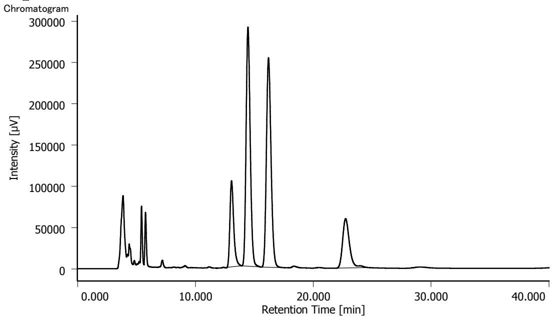

tR [min]	Area%
13.058	12.412
14.450	37.649
16.192	36.945
22.733	12.994

Racemic sample

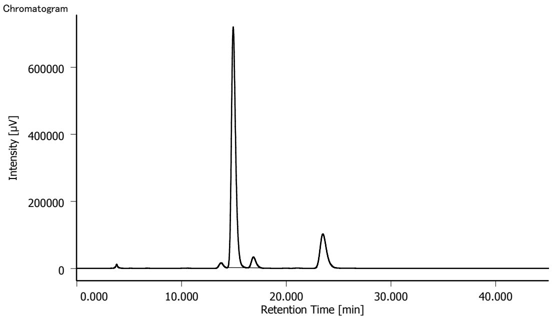

tR [min]	Area%
14.933	95.321
16.858	4.679

Reaction sample

(2*R*,3*S*)-2-Azido-5-cyclohexyl-1-(2,3-dihydro-1*H*-pyrrolo[2,3-*b*]pyridin-1-yl)-3-hydroxy-3-(trifluoromethyl)pent-4-yn-1-one (5k):

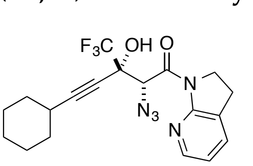

Prepared by the general procedure E from amide **2** (40.6 mg, 0.20 mmol, 1.0 equiv), ketone **4k** (44.2 μL, 0.24 mmol, 1.2 equiv), Barton's base (0.1 M in THF, 200 μL, 20 μmol, 10 mol%), $Cu(OTf)_2$ (7.4 mg, 0.020 mmol, 10 mol%), and BHA **8** (17.2 mg, 0.024 mmol, 12 mol%), stirred for 6 h at –40 °C, purified by column chromatography (hexane/EtOAc), and isolated as a pale yellow oil (66.8 mg, 82%). **IR** (thin film) 3348, 2944, 2832, 2520, 1655, 1449, 1417, 1028 cm^{-1}; ^{1}**H NMR** (400 MHz, $CDCl_3$) δ 8.1 (dd, *J* = 1.5, 5.3 Hz, 1H), 7.59 (dd, *J* = 1.5, 7.4 Hz, 1H), 7.12 (s, 1H), 7.02 (dd, *J* = 5.2, 7.4 Hz, 1H), 5.63 (s, 1H), 4.32 (ddd, *J* = 5.3, 10.1, 12.3 Hz, 1H), 4.13 (ddd, *J* = 7.8, 10.1, 12.3 Hz, 1H), 3.28–3.03 (m, 2H), 2.53 (tt, *J* = 3.8, 9.2 Hz, 1H), 1.88–1.85 (m, 2H), 1.77–1.70 (m, 2H), 1.65–1.46 (m, 3H), 1.40–1.19 (m, 3H); ^{13}**C NMR** (100 MHz, $CDCl_3$) δ 166.5, 154.7, 145.6, 135.2, 127.2, 123.7 (q, *J* = 281.6 Hz), 119.9, 95.3, 74.5 (q, *J* = 30.6 Hz), 71.9, 57.8, 46.8, 31.9, 29.2, 25.9, 24.9, 24.6; ^{19}**F NMR** (376 MHz, $CDCl_3$) δ –79.3; **HRMS** (ESI): *m*/*z* calc'd for $C_{19}H_{20}O_2N_5F_3Na$ [M + Na]$^+$: 430.1461, found: 430.1456. $[\alpha]_D^{28}$ –87.0 (*c* 0.16, $CHCl_3$, 83% ee sample); HPLC analysis (CHIRALPAK AD-3 (ϕ = 0.46 cm x 25 cm), 2 propanol/*n*-hexane = 1/19, flow rate = 1.0 mL/min, detection at 254 nm, t_R = 17.1 min (minor), 24.8 min (major)):

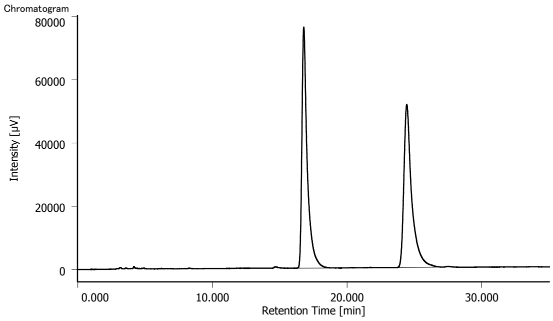

tR [min]	Area%
16.783	50.187
24.408	49.813

Racemic sample

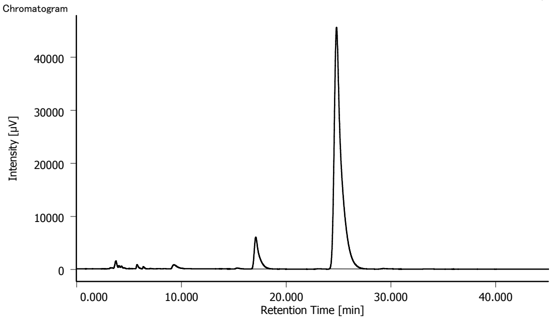

tR [min]	Area%
17.092	8.329
24.800	91.671

Reaction sample

(2*R*,3*S*)-2-Azido-3-(difluoromethyl)-1-(2,3-dihydro-1*H*-pyrrolo[2,3-*b*]pyridin-1-yl)-3-hydroxyundec-4-yn-1-one (12a):

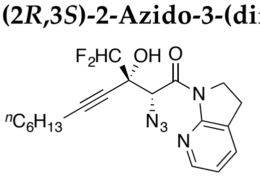

Prepared by the general procedure E from amide **2** (20.3 mg, 0.10 mmol, 1.0 equiv), ketone **11a** (44.8 μL, 0.12 mmol, 1.2 equiv), Barton's base (0.1 M in THF, 100 μL, 10 μmol, 10 mol%), $Cu(OTf)_2$ (3.7 mg, 0.010 mmol, 10 mol%), and BHA **8** (8.6 mg, 0.012 mmol, 12 mol%), stirred for 6 h at –40 °C, purified by column chromatography (hexane/EtOAc), and isolated as a pale brown oil (23.0 mg, 75%). **IR** (thin film) 3020, 2253, 2116, 1671, 1600, 1429, 1215, 908 cm^{-1}; ^{1}**H NMR** (400 MHz, $CDCl_3$) δ 8.13–8.06 (m, 1H), 7.58 (dd, *J* = 1.5, 7.4 Hz, 1H), 7.01 (dd, *J* = 5.2, 7.4 Hz, 1H), 6.36 (s, 1H), 5.96 (t, *J* = 56.0 Hz, 1H), 5.55 (s, 1H), 4.34 (ddd, *J* = 5.1, 10.2, 12.3 Hz, 1H), 4.12 (ddd, *J* = 7.9, 10.2, 12.3 Hz, 1H), 3.26–3.04 (m, 2H), 2.31 (t, *J* = 7.2 Hz, 2H), 1.67–1.48 (m, 2H), 1.46–1.38 (m, 2H), 1.36–1.21 (m, 4H), 0.89 (t, *J* = 6.8 Hz, 3H); ^{13}**C NMR** (100 MHz, $CDCl_3$) δ 166.8, 154.9, 145.6, 135.0, 127.1, 119.8, 114.7 (t, *J* = 250.0 Hz), 91.8, 74.3 (t, *J* = 23.3 Hz), 73.3, 58.3, 46.7, 31.4, 28.7, 28.1, 24.6, 22.7,

19.1, 14.2; **^{19}F NMR** (376 MHz, $CDCl_3$) δ –129.3 (dd, *J* = 56.0, 273.4 Hz), –132.9 (dd, *J* = 56.0, 273.4 Hz); **HRMS** (ESI): *m*/*z* calc'd for $C_{19}H_{23}O_2N_5F_2Na$ [M + Na]$^+$: 414.1712, found: 414.1706. $[\alpha]_D^{26}$ –27.7 (*c* 0.19, $CHCl_3$, 90% ee sample); HPLC analysis (CHIRALPAK AD-3 (ϕ = 0.46 cm x 25 cm), 2 propanol/*n*-hexane = 1/19, flow rate = 1.0 mL/min, detection at 254 nm, t_R = 22.4 min (minor), 25.9 min (major)):

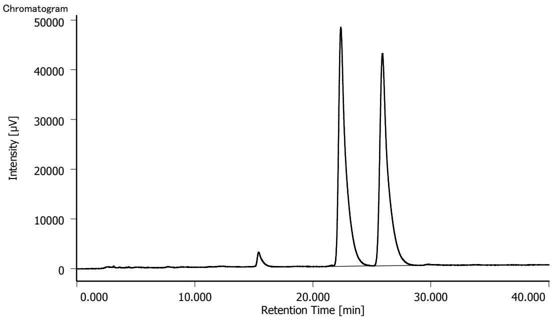

tR [min]	Area%
22.358	50.055
25.883	49.945

Racemic sample

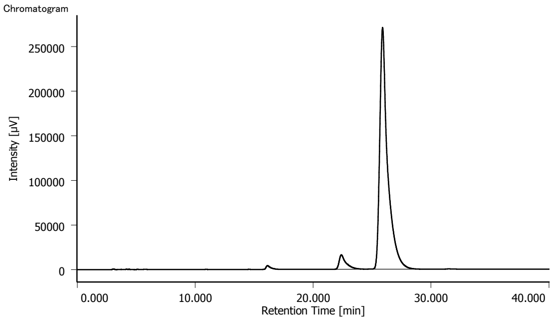

tR [min]	Area%
22.392	4.975
25.875	95.025

Reaction sample

(2*R*,3*S*)-2-Azido-3-(chlorodifluoromethyl)-1-(2,3-dihydro-1*H*-pyrrolo[2,3-*b*]pyridin-1-yl)-3-hydroxyundec-4-yn-1-one

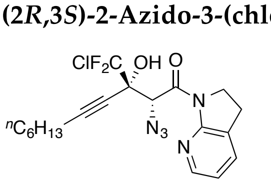

(12b): Prepared by the general procedure E from amide **2** (20.3 mg, 0.10 mmol, 1.0 equiv), ketone **11b** (50.8 μL, 0.12 mmol, 1.2 equiv), Barton's base (0.1 M in THF, 100 μL, 10 μmol, 10 mol%), $Cu(OTf)_2$ (3.7 mg, 0.010 mmol, 10 mol%), and BHA **8** (8.6 mg, 0.012 mmol, 12 mol%), stirred for 6 h at –40 °C, purified by column chromatography (hexane/EtOAc), and isolated as a colorless oil (27.3 mg, 80%). **IR** (thin film) 3019, 2399, 1524, 1426, 1215, 759 cm^{-1}; **^{1}H NMR** (400 MHz, $CDCl_3$) δ 8.12–8.07 (m, 1H), 7.59 (dd, *J* = 1.5, 7.5 Hz, 1H), 7.28 (s, 1H), 7.02 (dd, *J* = 5.2, 7.5 Hz, 1H), 5.77 (s, 1H), 4.32 (ddd, *J* = 5.2, 10.1, 12.3 Hz, 1H), 4.12 (ddd, *J* = 7.8, 10.1, 12.3 Hz, 1H), 3.25–3.05 (m, 2H), 2.33 (t, *J* = 7.1 Hz, 2H), 1.67–1.57 (m, 2H), 1.49–1.38 (m, 2H), 1.37–1.24 (m, 4H), 0.89 (t, *J* = 6.8 Hz, 3H); **^{13}C NMR** (100 MHz, $CDCl_3$) δ 166.8, 154.7, 145.6, 135.1, 129.7 (t, *J* = 302.5 Hz), 127.2, 119.9, 92.1, 78.4 (t, *J* = 27.2 Hz), 72.7, 58.2, 46.8, 31.4, 28.6, 27.9, 24.6, 22.7, 19.1, 14.2; **^{19}F NMR** (376 MHz, $CDCl_3$) δ –62.3 (d, *J* = 161.3 Hz), –64.4 (d, *J* = 161.3 Hz); **HRMS** (ESI): *m*/*z* calc'd for $C_{19}H_{22}O_2N_5ClF_2Na$ [M + Na]$^+$: 448.1322, found: 448.1317. $[\alpha]_D^{27}$ –24.5 (*c* 0.16, $CHCl_3$, 93% ee sample); HPLC analysis (CHIRALCEL OD–3 (ϕ = 0.46 cm x 25 cm), 2 propanol/*n*-hexane = 1/19, flow rate = 1.0 mL/min, detection at 254 nm, t_R = 13.1 min (major), 14.9 min (minor)):

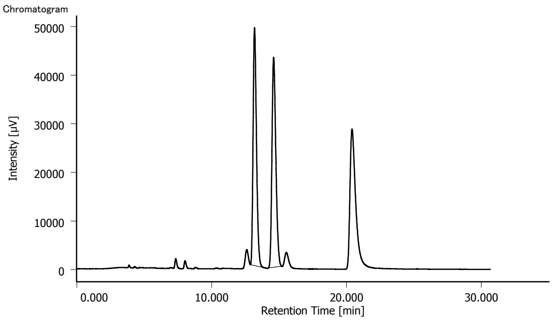

tR [min]	Area%
13.175	49.558
14.583	50.442

Racemic sample

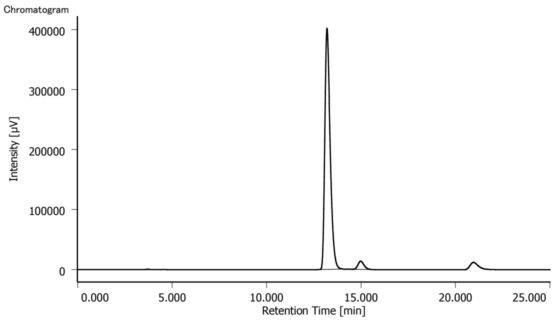

tR [min]	Area%
13.192	96.311
14.975	3.689

Reaction sample

(2*R*,3*S*)-2-Azido-3-(bromodifluoromethyl)-1-(2,3-dihydro-1*H*-pyrrolo[2,3-*b*]pyridin-1-yl)-3-hydroxyundec-4-yn-1-one

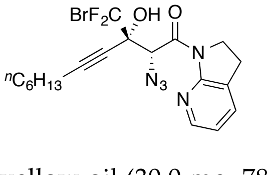

(12c): Prepared by the general procedure E from amide **2** (20.3 mg, 0.10 mmol, 1.0 equiv), ketone **11c** (51.4 μL, 0.12 mmol, 1.2 equiv), Barton's base (0.1 M in THF, 200 μL, 200 μmol, 20 mol%), $Cu(OTf)_2$ (7.4 mg, 0.020 mmol, 20 mol%), and BHA **8** (17.2 mg, 0.024 mmol, 24 mol%), stirred for 6 h at –40 °C, purified by column chromatography (hexane/EtOAc), and isolated as a pale yellow oil (30.0 mg, 78%). **IR** (thin film) 3019, 2399, 1680, 1525, 1429, 1215, 759 cm^{-1}; **^{1}H NMR** (400 MHz, $CDCl_3$) δ 8.10 (dd, *J* = 1.8, 5.1 Hz, 1H), 7.59 (dd, *J* = 1.4, 7.5 Hz, 1H), 7.35 (s, 1H), 7.03 (dd, *J* = 5.1, 7.5 Hz, 1H), 5.77 (s, 1H), 4.32 (ddd, *J* =

5.4, 10.1, 12.3 Hz, 1H), 4.12 (ddd, *J* = 7.8, 10.1, 12.3 Hz, 1H), 3.25–3.05 (m, 2H), 2.33 (t, *J* = 7.1 Hz, 2H), 1.64–1.57 (m, 2H), 1.51–1.40 (m, 2H), 1.37–1.22 (m, 4H), 0.89 (t, *J* = 6.8 Hz, 3H); ^{13}**C NMR** (125 MHz, $CDCl_3$) δ 166.7, 154.7, 145.6, 135.1, 127.2, 125.3 (t, *J* = 315.5 Hz), 119.9, 92.3, 79.2 (t, *J* = 24.8 Hz), 72.9, 58.2, 46.8, 31.4, 28.6, 27.9, 24.6, 22.7, 19.1, 14.2; ^{19}**F NMR** (376 MHz, $CDCl_3$) δ –55.4 (d, *J* = 160.1 Hz), –58.3 (d, *J* = 160.1 Hz); **HRMS** (ESI): *m/z* calc'd for $C_{19}H_{22}O_2N_5BrF_2Na$ [M + Na]$^+$: 492.0817, found: 492.0811. $[\alpha]_D^{26}$ 1.7 (*c* 0.14, $CHCl_3$, 94% ee sample); HPLC analysis (CHIRALCEL OD-3 (ϕ = 0.46 cm x 25 cm), 2 propanol/*n*-hexane = 1/19, flow rate = 0.5 mL/min, detection at 254 nm, t_R = 17.2 min (minor), 18.1 min (major)):

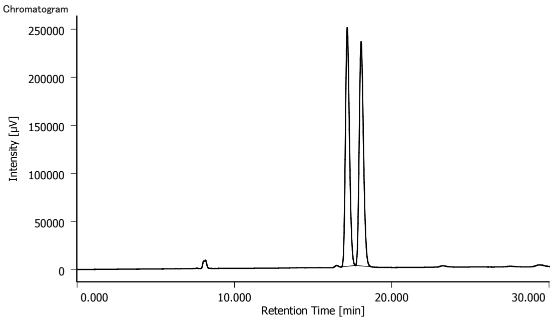

tR [min]	Area%
17.167	49.997
18.050	50.003

Racemic sample

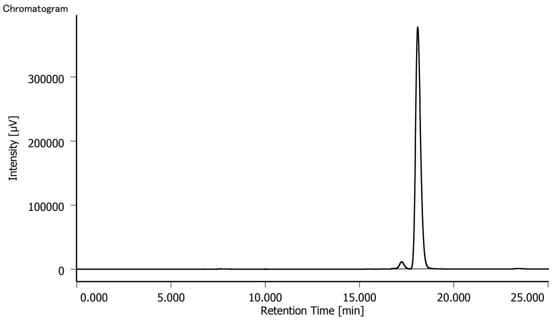

tR [min]	Area%
17.225	2.899
18.083	97.101

Reaction sample

*3-5. Gram scale synthesis of **5a** and its SDE test on achiral silica gel chromatgraphy*

To a flame dried 100 mL flask equipped with a magnetically stirred chip was added amide **2** (1.02 g, 5.0 mmol, 1.0 equiv) and BHA **8** (429 mg, 0.60 mmol, 12 mol%). In a glove box, $Cu(OTf)_2$ (181 mg, 10 mol%) and MS13X (5.10 g, 500 % w/w) were added to the flask. After it was taken out from the glove box, THF (15 mL) and CF_3 ketone (1.70 mL, 6.0 mmol, 1.2 equiv) were added. The solution was stirred for 10 min at RT and 5 min at –40 °C before the addition of the solution of Barton's base (5.0 mL, 0.1 M in THF, 0.50 mmol, 5 mol%). After the addition of sat aq NH_4Cl at –40 °C, the solution was diluted with H_2O and EtOAc at RT and filtered through a pad of Celite. The aqueous phase was extracted with EtOAc (3x). The combined organic phases were dried over Na_2SO_4, filtered, and removed under reduced pressure. The crude material was purified by silica gel chromatography (hexane/EtOAc), and nine fractions were collected. For each fraction, the ee of **5a** was determined by chiral HPLC. The obtained data was summarized below, confirming that no SDE was involved. All fractions were combined and evaporated to give **5a** (2.37 g, 98%) as a colorless oil.

fraction	1	2	3	4	5	6	7	8	9
ee (*syn*)	95	95	94	94	94	94	94	94	94

4. Transformations of the aldol products

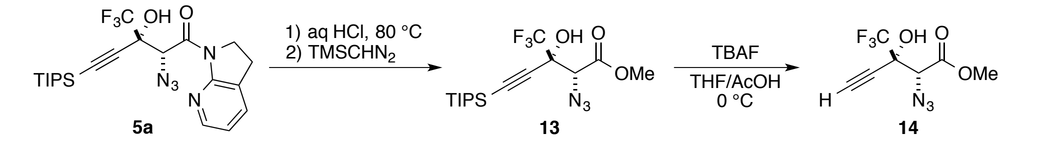

Methyl (2*R*,3*S*)-2-azido-3-hydroxy-3-(trifluoromethyl)-5-(triisopropylsilyl)pent-4-ynoate (13): To a solution of **5a**

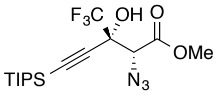

(31.4 mg, 65 μmol, 1.0 equiv) in CH_3CN (0.1 mL) was added conc. HCl (0.3 mL), and the solution was stirred at 80 °C for 15 h. After cooling to RT, sat aq $NaHCO_3$ was added and the resulting solution was evaporated. The obtained residue was suspended in 10:1 toluene/MeOH (0.5 mL) and cooled to 0 °C. To this, trimethylsilyldiazomethane (2.0 M in hexane, 65 μL, 0.13 mmol, 2.0 equiv) was added dropwise. The suspension was stirred for 5 min at the same temperature and for 30 min at RT before the addition of AcOH (a few drops), H_2O and EtOAc successively. The aqueous phase was extracted with EtOAc (3x). The combined organic layers were washed with brine, dried over Na_2SO_4, filtered, and removed under reduced pressure. The crude material was purified by flash column chromatography (hexane/EtOAc), affording the title compound as a colorless oil (18.7 mg, 73%). **IR** (thin film) 3019, 2946, 2399, 2121, 1438, 1215, 758 cm^{-1}; **^{1}H NMR** (500 MHz, C_6D_6) δ 4.33 (s, 1H), 3.18 (s, 3H), 1.09–0.98 (m, 21H); **^{13}C NMR** (125 MHz, C_6D_6) δ 168.4, 123.3 (q, *J* = 284.3 Hz), 98.8, 93.4, 72.8 (q, *J* = 31.3 Hz), 62.6 (q, *J* = 22.8 Hz), 52.9, 18.6, 11.3; **^{19}F NMR** (376 MHz, $CDCl_3$) δ –78.4; **HRMS** (ESI): *m/z* calc'd for $C_{16}H_{26}O_3N_3F_3NaSi$ [M + Na]$^+$: 416.1588, found: 416.1590. $[\alpha]_D^{25}$ –3.9 (*c* 0.72, $CHCl_3$, 94% ee sample)

Methyl (2*R*,3*S*)-2-azido-3-hydroxy-3-(trifluoromethyl)pent-4-ynoate (14): To a solution of **13** (5.1 mg, 13 μmol, 1.0

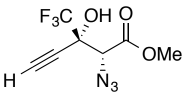

equiv) in THF (0.3 mL) was added AcOH (1.5 μL, 26 μmol, 2.0 equiv). The solution was cooled to 0 °C and TBAF (1.0 M in THF, 13 μL, 13 μmol, 1.0 equiv) was added. After 60 min, sat aq NH_4Cl was added and the aqueous phase was extracted with EtOAc (3x). The combined organic phase was washed with brine, dried over Na_2SO_4, filtered, and removed under reduced pressure. The obtained residue was purified by flash column chromatography, eluting with hexane/EtOAc to give **14** as a colorless oil (2.6 mg, 84%). **IR** (thin film) 3303, 3019, 2121, 1748, 1438, 1215, 758 cm^{-1}; **^{1}H NMR** (400 MHz, $CDCl_3$) δ 4.44 (s, 1H), 3.93 (s, 3H), 2.75 (s, 1H); **^{13}C NMR** (100 MHz, $CDCl_3$) δ 168.3, 122.4 (q, *J* = 284.4 Hz), 78.3, 75.3, 72.5 (q, *J* = 31.8 Hz), 62.1 (q, *J* = 38.4 Hz), 53.9; **^{19}F NMR** (376 MHz, $CDCl_3$) δ –78.5; measurement of the exact mass has so far proven unsuccessful. $[\alpha]_D^{26}$ 27.8 (*c* 1.0, $CHCl_3$, 94% ee sample).

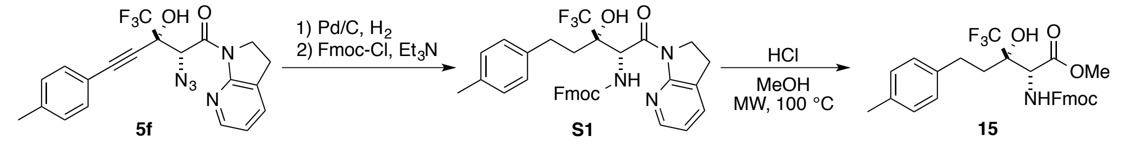

(9*H*-Fluoren-9-yl)methyl ((2*R*,3*S*)-1-(2,3-dihydro-1*H*-pyrrolo[2,3-*b*]pyridin-1-yl)-3-hydroxy-1-oxo-5-(*p*-tolyl)-3-(trifluoromethyl)pentan-2-yl)carbamate (S1): To a solution of **5f** (30.2 mg, 73 μmol, 1.0 equiv) in MeOH (1.00 mL) was added

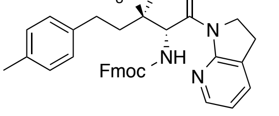

Pd/C (3.2 mg, 10% w/w). The mixture was stirred for 3 h at RT under an hydrogen atmosphere. After refilling Ar into the flask, the suspension was filtered through a pad of Celite, and the filtrate was evaporated. The obtained residue was dissolved in CH_2Cl_2 (1.00 mL), and Et_3N (30.4 μmol, 219 μmol, 3.0 equiv) and Fmoc-Cl (20.7 mmol, 80 μmol, 1.1 equiv) were added. The solution was stirred for 12 h at RT. After the addition of sat aq NH_4Cl, the aqueous phase was extracted with EtOAc (3x). The combined organic phase was washed with brine, dried over Na_2SO_4, filtered, and removed under reduced pressure. The crude material was purified by flash column chromatography (hexane/EtOAc) to give **S1** as a colorless oil (39 mg, 87%). **IR** (thin film) 3019, 2399, 2253, 1510, 1428, 1215, 909, 758 cm^{-1}; **^{1}H NMR** (400 MHz, $CDCl_3$) δ 8.20–8.11 (m, 1H), 7.75 (d, *J* = 7.6 Hz, 2H), 7.60–7.52 (m, 3H), 7.38 (dd, *J* = 7.5, 7.5 Hz, 2H), 7.31–7.24 (m, 2H), 7.15–7.09 (m, 4H), 7.07–6.96 (m, 1H), 6.50 (d, *J* = 10.1 Hz, 1H), 5.77 (d, *J* = 10.1 Hz, 1H), 4.49–4.31 (m, 2H), 4.31–4.14 (m, 2H), 4.13–4.04 (m, 1H), 3.24–3.15 (m, 1H), 3.14–2.97 (m, 1H), 2.82 (dt, *J* = 5.2, 12.0, 2H), 2.32 (s, 3H), 2.31–2.24 (m, 1H), 2.23–2.09 (m, 1H); **^{13}C NMR** (125 MHz, $CDCl_3$) δ 168.7, 155.6, 155.0, 145.4, 143.9, 143.8, 141.4, 139.0, 135.6, 135.3, 129.3, 128.6, 127.5, 127.2, 126.3 (q, *J* = 285.9 Hz),

125.3, 120.1, 120.0, 76.5 (q, *J* = 26.3 Hz), 67.7, 53.6, 47.2, 47.1, 32.7, 28.4, 24.7, 21.2.; **^{19}F NMR** (376 MHz, $CDCl_3$) δ –76.3; **HRMS** (ESI): *m*/*z* calc'd for $C_{35}H_{33}O_4N_3F_3$ [M + H]$^+$: 616.2418, found: 616.2410. $[\alpha]_D^{26}$ 3.9 (*c* 0.14, $CHCl_3$, 90% ee sample)

Methyl (2*R*,3*S*)-2-(((9*H*-fluoren-9-yl)methoxy)carbonyl)amino)-3-hydroxy-5-(*p*-tolyl)-3-(trifluoromethyl)pentanoate (15):

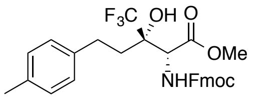

S1 (6.0 mg, 9.7 μmol) was taken in a vial for a microwave reactor. To this, HCl in MeOH (2 M, 0.5 mL) was added. The vial was tightly closed and placed on the reactor. The solution was stirred for 24 h at 100 °C. The volatile was removed to give the crude residue, which was purified by flash column chromatography (hexane/EtOAc), affording **15** as a pale brown oil (3.9 mg, 76%). **IR** (thin film) 3019, 2399, 1514, 1215, 909, 760 cm^{-1}; **^{1}H NMR** (500 MHz, $CDCl_3$, 273K) δ 7.83–7.70 (m, 2H), 7.64–7.52 (m, 2H), 7.46–7.38 (m, 2H), 7.34–7.30 (m, 2H), 7.14–7.06 (m, 4H), 5.62 (d, *J* = 9.8 Hz, 1H), 4.86 (d, *J* = 9.8 Hz, 1H), 4.53–4.41 (m, 2H), 4.23 (t, *J* = 6.9 Hz, 1H), 3.83 (s, 3H), 3.79 (s, 1H), 2.80 (t, *J* = 8.6 Hz, 2H), 2.31 (s, 3H), 2.10 (t, *J* = 8.6 Hz, 2H); **^{13}C NMR** (125 MHz, CD_3Cl, 273K) δ 170.1, 156.8, 143.4, 141.4, 137.8, 135.9, 129.4, 128.4, 128.0, 127.3, 125.2 (q, J = 286.3 Hz), 125.1, 120.2, 76.6 (q, J = 27.2 Hz), 68.0, 54.9, 53.6, 47.0, 34.5, 28.4, 21.2; **^{19}F NMR** (376 MHz, $CDCl_3$) δ –76.7; **HRMS** (ESI): *m*/*z* calc'd for $C_{29}H_{28}O_5NF_3Na$ [M + Na]$^+$: 550.1812, found: 550.1802. $[\alpha]_D^{26}$ 3.9 (*c* 0.14, $CHCl_3$, 90% ee sample)

5. Mechanistic study

5-1. NMR experiments

5-1-1. Spectra from BHA **8**, $Cu(OTf)_2$, and Bartons' base

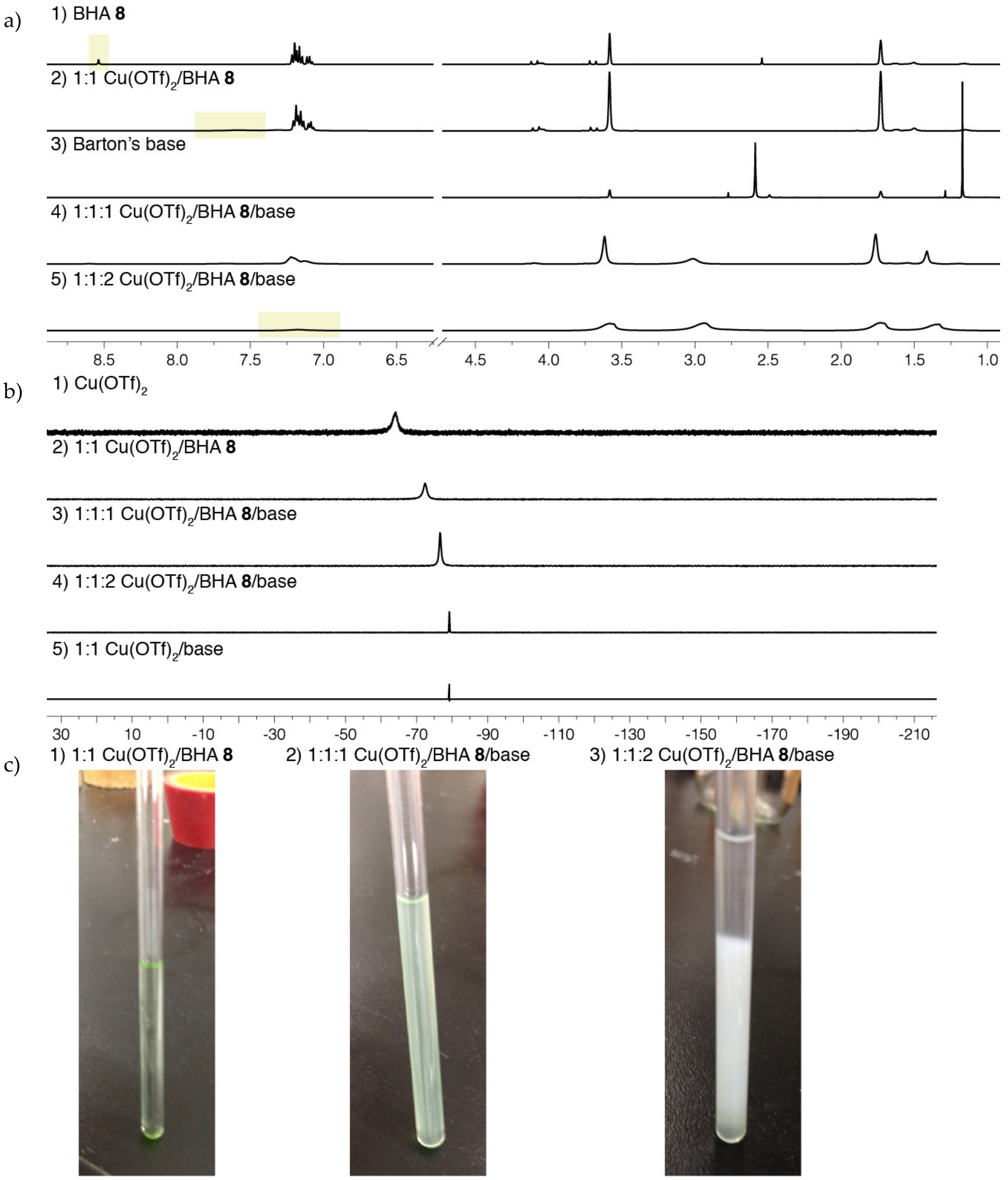

Fig. S2 NMR experiments in THF-*d*8 of BHA **8**, $Cu(OTf)_2$, and Barton's base. a) Stacked ^{1}H NMR spectra. b) Stacked ^{19}F NMR spectra. c) Appearances of NMR samples.

5-1-2. Spectra from BHA **8** and Barton's base

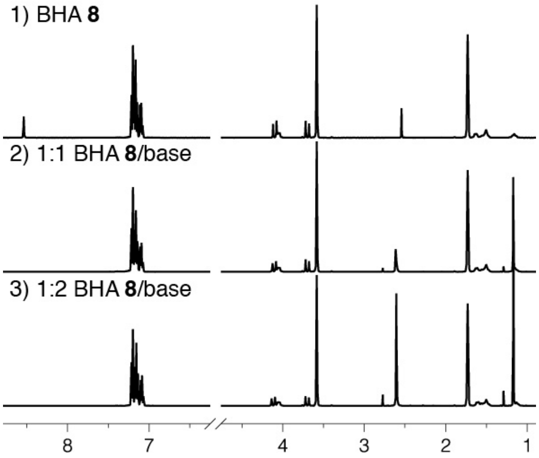

Fig. S3 Stacked ^1H NMR spectra in THF-d_8 from a mixture of BHA **8** and Barton's base.

*5-2. Solid state structures of BHA 8 and 1:2 Cu/amide **2** complex (**E**)*

5-2-1. Solid state structure of BHA **8**

Single crystals of ligand **8** were obtained by slow evaporation of the solution of **8** in THF at RT. A suitable crystal was selected and the sample was measured on a Rigaku R-AXIS RAPID diffractometer using graphite monochromated Cu-Ka radiation. The data were collected at 93 K. Refined structure and crystallographic parameters are summarized in Fig. S4 and Table S4. CCDC 1498994 contains the supplementary crystallographic data for **8**.

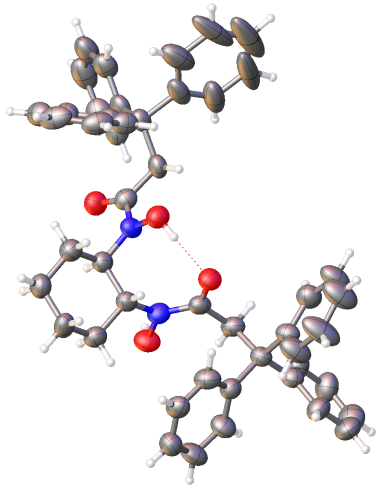

Fig. S4 ORTEP diagram of ligand **8**. Solvent molecules (THF) have been omitted for clarity.

Table S4 Selected crystal data of ligand **8**.

Empirical Formula	$C_{50}H_{50}N_2O_{4.5}$
Formula Weight	750.96
Crystal Color, Habit	colorless, needle
Crystal Dimensions	0.200 x 0.050 x 0.050 mm
Crystal System	orthorhombic
Lattice Parameters	
a	10.8070(2) Å
b	27.0000(5) Å
c	28.2406(7) Å
V	8240.3(3) $Å^3$
Space Group	$P2_12_12_1$ (#19)
Z value	8
D_{calc}	1.211 g/cm^3
F_{000}	3200.00

5-2-2. Solid state structure of **E**

Single crystals of **E** (1:2 Cu/amide **2** complex) were obtained from the solution of $Cu(OTf)_2$, ligand **8**, and amide **2** in THF-d_8 at RT. A suitable crystal was selected and the sample was measured on a Rigaku R-AXIS RAPID diffractometer using graphite monochromated Cu-Ka radiation. The data were collected at 93 K. Refined structure and crystallographic parameters are summarized in Fig. S5 and Table S5. CCDC 1498995 contains the supplementary crystallographic data for **E**.

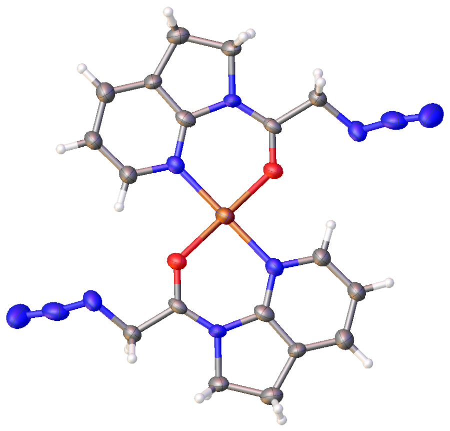

Fig. S5 ORTEP diagram of **E**. Triflate anions have been omitted for clarity.

Table S5 Selected crystal data of **E**.

Empirical Formula	$C_{20}H_{18}CuF_6N_{10}O_8S_2$
Formula Weight	768.08
Crystal Color, Habit	green, platelet
Crystal Dimensions	0.050 x 0.050 x 0.002 mm
Crystal System	triclinic
Lattice Parameters	
a	7.3568(6) Å
b	10.0126(8) Å
c	10.8607(9) Å
α	110.106(8) °
β	95.309(7) °
γ	106.922(8) °
V	702.02(11) Å^3
Space Group	*P-1* (#2)
Z value	1
D_{calc}	1.817 g/cm^3
F_{000}	387.00

5-3. Time course study of the aldol reaction

5-3-1. Preparation of amide **2**-*d*2

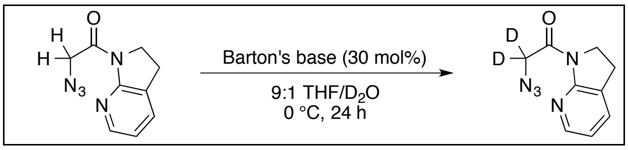

Procedure: To a solution of amide **2** (406 mg, 2.0 mmol, 1.0 equiv) in THF (24 mL) was added D_2O (1.2 mL), and the solution was cooled to 0 °C. Barton's base (0.1 M in THF, 6.0 mL, 0.60 mmol, 30 mol%) was added slowly, and the mixture was stirred for 24 h at the same temperature. After the addition of sat aq NH_4Cl at 0 °C, the solution was diluted with H_2O and EtOAc at RT. The aqueous phase was extracted with EtOAc (3x). The combined organic layers were washed with brine, dried over Na_2SO_4, filtered, and removed under reduced pressure. The obtained solid was purified by silica gel column chromatography to give amide **2**-*d*2 (372 mg, 91%, >97% D incorporation).

5-3-2. Time course study

5-3-2-a. Reaction progress under the standard conditions (Supplementary result)

Table S6 Reaction progress with amide **2** at –40 °C

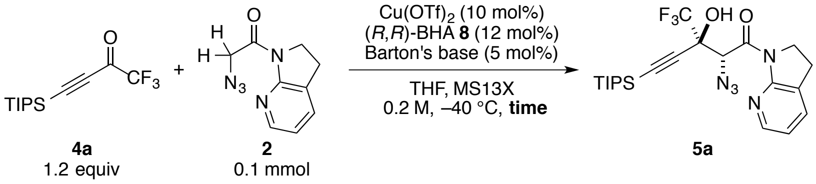

time (min)	yield	*anti*/*syn*	ee (*syn*)
30	81	17/83	99
60	95	17/83	99
120	98	18/82	99
180	97	17/83	94

5-3-2-b. Reaction progress under diluted conditions (Fig. 2a, blue and red squares)

General procedure F (Fig. 2): To a flame dried test tube equipped with a magnetically stirred chip was added amide **2** (20.3 mg, 0.1 mmol, 1.0 equiv) and BHA **8** (8.6 mg, 0.012 mmol, 12 mol%). In a glove box, $Cu(OTf)_2$ (3.6 mg, 0.010 mmol, 10 mol%) and MS13X (101 mg, 500 % w/w) were added to the test tube. After it was taken out from the glove box, THF (70 mM) and CF_3 ketone (34.0 μL, 0.12 mmol, 1.2 equiv) were added. The solution was stirred for 10 min at RT and 5 min at –40 °C before the addition of the solution of Barton's base (0.1 M in THF, 100 μL, 10 μmol, 5 mol%). The reaction was stirred for the indicated time. After the addition of sat aq NH_4Cl at –40 °C, the solution was diluted with H_2O and EtOAc at RT and filtered through a pad of Celite. The aqueous phase was extracted with EtOAc (3x). The combined organic phases were dried over Na_2SO_4, filtered, and removed under reduced pressure.

Table S7 Reaction progress with amide **2** at –60 °C

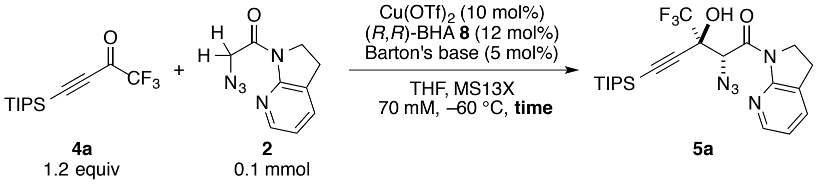

time (min)	yield	*anti*/*syn*	ee (*syn*)
15	16	11/89	97
30	25	12/88	94
60	31	12/88	98
180	65	11/89	93
360	76	10/90	95
720	96	11/89	96

Table S8 Reaction progress with amide **2**-d_2 at –60 °C

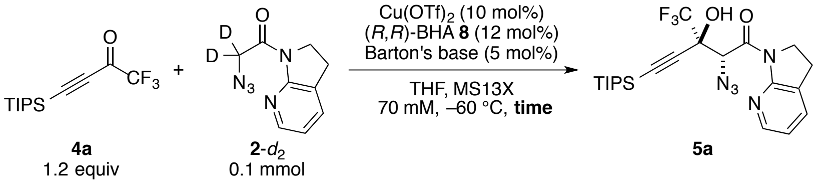

time (min)	yield	*anti*/*syn*	ee (*syn*)
15	13	18/82	99
30	16	16/84	99
60	17	17/83	99
180	25	14/86	99
360	42	12/88	99
720	61	11/89	99

5-3-2-c. Time course study under BHA **8** free conditions (Fig. 2a, black squares)

Table S9 Reaction progress with amide **2** without added BHA **8** at –60 °C

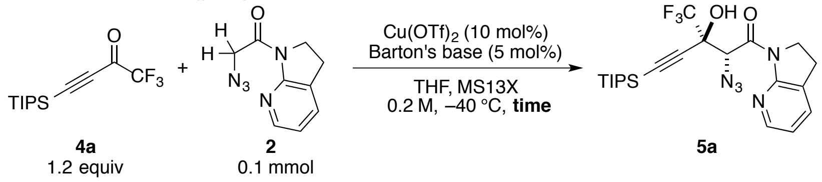

time (min)	yield	*anti*/*syn*
15	2	58/42
30	3	60/40
60	5	55/45
180	9	58/42
720	27	64/36

5-3-2-d. Time course study with amide **2** using 5 mol% catalyst

Table S10 Reaction progress with amide **2** using 5 mol% catalyst at –60 °C

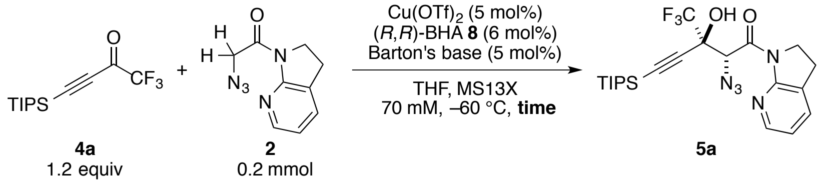

time (min)	yield	*anti*/*syn*	ee (*syn*)
15	5	13/87	97
30	11	11/89	96
60	14	12/88	97
180	33	14/86	94
360	41	11/89	93
720	50	11/89	96

5-3-3. Analysis by normalized time scale method

i) x = 1.0

time (h)	[amide **2**] (10 mol%)	t[cat]^x (10 mol%)	[amide **2**] (5 mol%)	t[cat]^x (5 mol%)
0.25	0.0587999970	0.0017499999	0.0665000007	0.0008750000
0.50	0.0524999984	0.0034999999	0.0622999966	0.0017499999
1.0	0.0482999980	0.0070000002	0.0601999983	0.0034999999
3.0	0.0244999994	0.0210000016	0.0469000004	0.0104999999
6.0	0.0167999994	0.0420000032	0.0412999988	0.0210000016
12.0	0.0027999999	0.0840000063	0.0350000001	0.0420000032

ii) x = 1.5

time (h)	[amide **2**] (10 mol%)	t[cat]^x (10 mol%)	[amide **2**] (5 mol%)	t[cat]^x (5 mol%)
0.25	0.0587999970	0.0001460000	0.0665000007	0.0000520000
0.50	0.0524999984	0.0002930000	0.0622999966	0.0001040000
1.0	0.0482999980	0.0005860000	0.0601999983	0.0002070000
3.0	0.0244999994	0.0017570000	0.0469000004	0.0006210000
6.0	0.0167999994	0.0035140000	0.0412999988	0.0012420000
12.0	0.0027999999	0.0070280000	0.0350000001	0.0024850001

iii) x = 2.0

time (h)	[amide **2**] (10 mol%)	t[cat]^x (10 mol%)	[amide **2**] (5 mol%)	t[cat]^x (5 mol%)
0.25	0.0587999970	0.0000120000	0.0665000007	0.0000030000

0.50	0.0524999984	0.0000240000	0.0622999966	0.0000060000
1.0	0.0482999980	0.0000490000	0.0601999983	0.0000120000
3.0	0.0244999994	0.0001470000	0.0469000004	0.0000370000
6.0	0.0167999994	0.0002940000	0.0412999988	0.0000730000
12.0	0.0027999999	0.0005880000	0.0350000001	0.0001470000

iv) x = 2.5

time (h)	[amide **2**] (10 mol%)	t[cat]^x (10 mol%)	[amide **2**] (5 mol%)	t[cat]^x (5 mol%)
0.25	0.0587999970	0.0000010000	0.0665000007	0.0000000000
0.50	0.0524999984	0.0000020000	0.0622999966	0.0000000000
1.0	0.0482999980	0.0000040000	0.0601999983	0.0000010000
3.0	0.0244999994	0.0000120000	0.0469000004	0.0000020000
6.0	0.0167999994	0.0000250000	0.0412999988	0.0000040000
12.0	0.0027999999	0.0000490000	0.0350000001	0.0000090000

5-4. Computational analysis on the conformation of fluorinated ketones

The ground state energies of model fluorinated ketones **S2–S5** were calculated by DFT method. All optimizations were conducted at the B3LYP/6-31+G(d) level of theory. Energies shown in Table S11 are after the zero-point corrections. Solvation by THF was taken into account by using the integral equation formalism polarizable continuum model (IEFPCM).

Ketones **S3–S5** have two stable conformers, while **S2** only has one (Table S11). Except **S3**, the stable conformers of **S2**, **S4** and **S5** take almost syn conformation between the carbonyl group and the fluorine atom, having larger dipole moment.

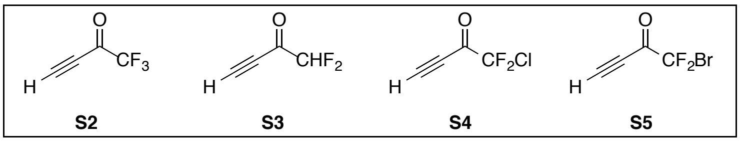

Table S11 Summary of calculated structures and energies of **S2–S5**.

	**S2a**	**S3a**	**S4a**	**S5a**
Sideview	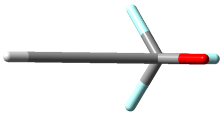	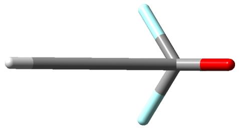	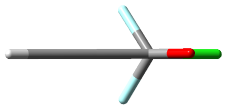	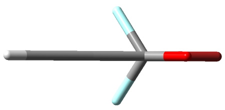
Dipole (D)	4.0800	2.0084	3.4293	3.1739
Energy (a.u.)	–527.673701	–428.415933	–888.014670	–2999.541754
ΔE (kcal/mol)	—	+0	+0.88	+1.12
		**S3b**	**S4b**	**S5b**
Sideview		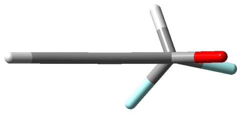	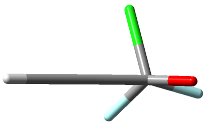	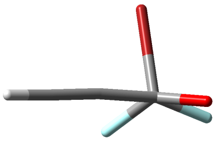
Dipole (D)		4.7189	4.1031	4.0364
Energy (a.u.)		–428.415257	–888.016080	–2999.543536
ΔE (kcal/mol)		+0.42	+0	+0

6. References

1. Fulmer, G. R. *et al.* NMR Chemical Shifts of Trace Impurities: Common Laboratory Solvents, Organics, and Gases in Deuterated Solvents Relevant to the Organometallic Chemist. *Organometallics* **29**, 2176–2179 (2010).
2. Frisch, M. J.; Trucks, G. W.; Schlegel, H. B.; Scuseria, G. E.; Robb, M. A.; Cheeseman, J. R.; Scalmani, G.; Barone, V.; Mennucci, B.; Petersson, G. A.; Nakatsuj, H.; Caricato, M.; Li, X.; Hratchian, H. P.; Izmaylov, A. F.; Bloino, J.; Zheng, G.; Sonnenberg, J. L.; Hada, M.; Ehara, M.; Toyota, K.; Fukuda, R.; Hasegawa, J.; Ishida, M.; Nakajima, T.; Honda, Y.; Kitao, O.; Nakai, H.; Vreven, T.; J. A. Montgomery, J.; Peralta, J. E.; Ogliaro, F.; Bearpark, M.; Heyd, J. J.; Brothers, E.; Kudin, K. N.; Staroverov, V. N.; Kobayashi, R.; Normand, J.; Raghavachari, K.; Rendell, A.; Burant, J. C.; Iyengar, S. S.; Tomasi, J.; Cossi, M.; Rega, N.; Millam, J. M.; Klene, M.; Knox, J. E.; Cross, J. B.; Bakken, V.; Adamo, C.; Jaramillo, J.; Gomperts, R.; Stratmann, R. E.; Yazyev, O.; Austin, A. J.; Cammi, R.; Pomelli, C.; Ochterski, J. W.; Martin, R. L.; Morokuma, K.; Zakrzewski, V. G.; Voth, G. A.; Salvador, P.; Dannenberg, J. J.; Dapprich, S.; Daniels, A. D.; Farkas, Ö.; Foresman, J. B.; Ortiz, J. V.; Cioslowski, J.; Fox, D. J. Gaussian 09, Revision D.01; Gaussian, Inc.: Wallingford, CT, 2013.
3. Linderman, R. J.; Lonikar, M. S. *J. Org. Chem.* **1988**, *53*, 6013–6022.
4. Maraval, V.; Leroyer, L.; Harano, A.; Barthes, C.; Saquet, A.; Duhayon, C.; Shinmyozu, T.; Chauvin, R. *Chem. Eur. J.* **2011**, *17*, 5086–5100.
5. Hanzawa, Y.; Kawagoe, K.; Ito, M.; Kobayashi, Y. *Chem. Pharm. Bull.* **1987**, *35*, 1633–1636.
6. Sasaki, S.; Ikekame, Y.; Tanayama, M.; Yamauchi, T.; Higashiyama, K. *Synlett* **2012**, 2699–2703.
7. Kuroboshi, M.; Ishihara, T. *Bull. Chem. Soc. Jpn.* **1990**, *63*, 428–437.
8. Armbrust, K. W.; Beaver, M. G.; Jamison, T. F. *J. Am. Chem. Soc.* **2015**, *137*, 6941–6946.
9. Komeyama, K.; Sasayama, D.; Kawabata, T.; Takehira, K.; Takaki, K. *Chem. Commun.* **2005**, 634–636.
10. Schlapbach, A.; Feifel, R.; Hawtin, S.; Heng, R.; Koch, G.; Moebitz, H.; Revesz, L.; Scheufler, C.; Velcicky, J.; Waelchli, R.; Huppertz, C. *Bioorg. Med. Chem. Lett.* **2008**, *18*, 6142–6146.

7. Optimized coordinates

S2a B3LYP/6-31+G(d)/IEFPCM(THF)

C	-0.47079657	0.69899523	0.00000032
O	-0.34455548	1.90621404	0.00000012
C	0.79261747	-0.21350064	0.00000010
C	-1.72580930	0.00221720	0.00000017
C	-2.78350663	-0.58744533	-0.00000000
F	0.79791739	-1.00898421	-1.09252098
F	0.79791349	-1.00899097	1.09251621
F	1.91560391	0.51271396	0.00000429
H	-3.72149912	-1.10396009	-0.00000028

S3a B3LYP/6-31+G(d)/IEFPCM(THF)

C	-0.31808137	0.71599795	0.00012772
O	-0.45622151	1.92769822	0.00033246
C	1.10244265	0.11689048	-0.00007209
C	-1.41282729	-0.21863655	0.00006131
C	-2.32831054	-1.01170399	0.00000282
F	1.25901738	-0.67891804	-1.10596029
F	1.25920559	-0.67919054	1.10559475
H	-3.14253979	-1.70676029	-0.00004741
H	1.86896450	0.89286443	-0.00004098

S3b B3LYP/6-31+G(d)/IEFPCM(THF)

C	0.27526662	0.60715868	0.01842656
O	0.05233129	1.77676486	-0.23236899
C	-0.87744616	-0.36102425	0.37541989
C	1.59498765	0.02939344	0.04419198
C	2.70395022	-0.45687012	0.07761710
F	-0.75156975	-1.50410904	-0.37884850
F	-2.07156924	0.21497872	0.07104569
H	3.68668667	-0.88069806	0.10309355
H	-0.87763606	-0.64319448	1.43215041

S4a B3LYP/6-31+G(d)/IEFPCM(THF)

C	-0.68676741	0.71358469	0.00000842
O	-0.42107515	1.89639907	0.00001879
C	0.43495890	-0.37975227	-0.00000281
C	-2.02030343	0.17923738	0.00000597
C	-3.14651496	-0.26578646	0.00000367
F	0.27191851	-1.17101917	-1.09201224
F	0.27193047	-1.17102761	1.09200246
H	-4.14414983	-0.65456182	0.00000171
Cl	2.06646303	0.29871119	-0.00000914

S4b B3LYP/6-31+G(d)/IEFPCM(THF)

C	-0.74896045	0.68441822	-0.24668774
O	-0.79901037	1.74932959	-0.82652334
C	0.61469908	0.17763638	0.31400350
C	-1.87248852	-0.18347107	-0.02991601
C	-2.82988024	-0.90245015	0.15062123
F	0.45434915	-0.29423697	1.57438226
F	1.49786235	1.18876519	0.36137052
H	-3.67707268	-1.53779281	0.30890624
Cl	1.26582547	-1.12731754	-0.72038944

S5a B3LYP/6-31+G(d)/IEFPCM(THF)

C	-1.13513832	0.69027587	-0.00001327
O	-0.79784603	1.85538482	-0.00004592
C	-0.08663437	-0.46592187	0.00001053
C	-2.50273986	0.25133885	0.00000636
C	-3.66654475	-0.08309958	0.00002381
F	-0.29765470	-1.24705471	-1.09248102
F	-0.29764718	-1.24700320	1.09254062
H	-4.69484130	-0.38139646	0.00003906
Br	1.73661915	0.16083656	-0.00001065

S5b B3LYP/6-31+G(d)/IEFPCM(THF)

C	-1.22456700	0.48276478	-0.43687999
O	-1.44743304	1.25043464	-1.35085270
C	0.07266434	0.64463994	0.39713404
C	-2.08326423	-0.60509151	-0.05605956
C	-2.88832479	-1.44732793	0.27410162
F	-0.16437579	0.44060812	1.71398158
F	0.57453824	1.88269520	0.25024364
H	-3.58417799	-2.20785820	0.56337069
Br	1.37751801	-0.66157887	-0.24298151

8. Spectra

5-((*Tert*-Butyldimethylsilyl)oxy)-1,1,1-trifluoropent-3-yn-2-one (4b):

^{1}H NMR (400MHz, $CDCl_3$)

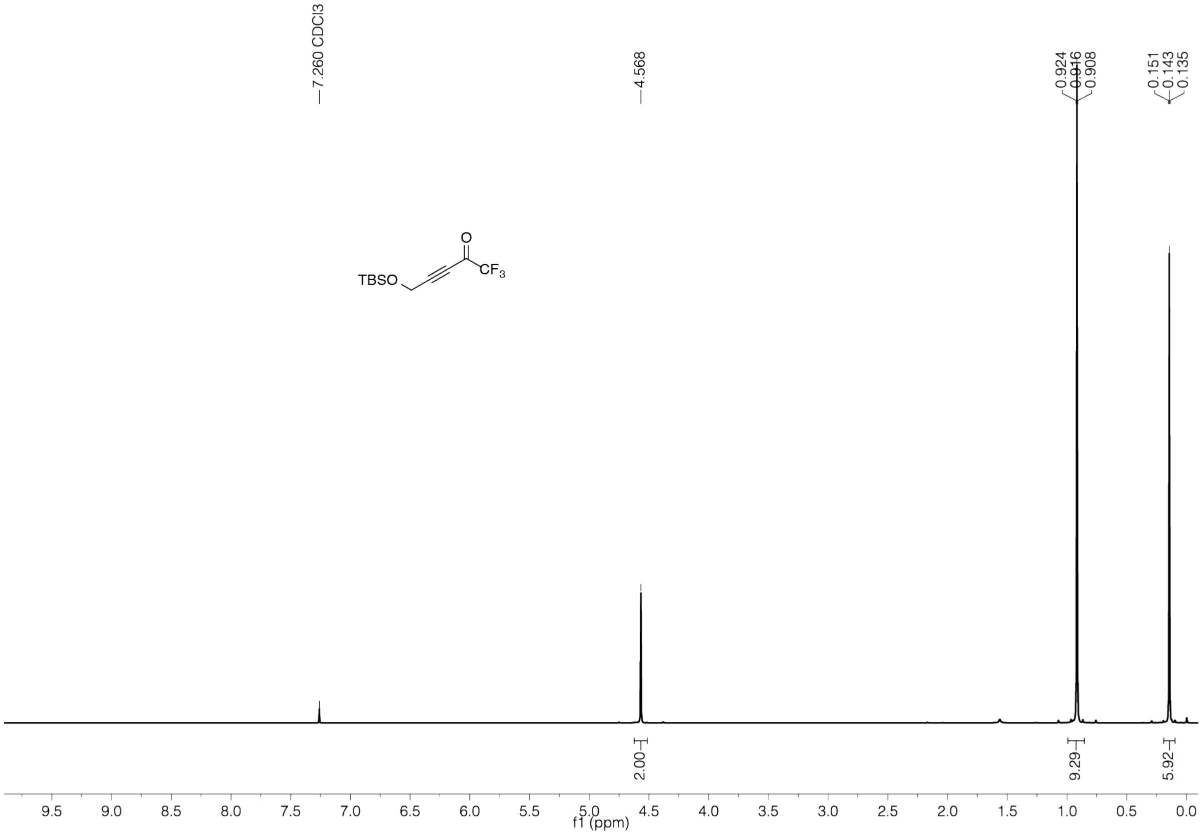

^{13}C NMR (100MHz, $CDCl_3$)

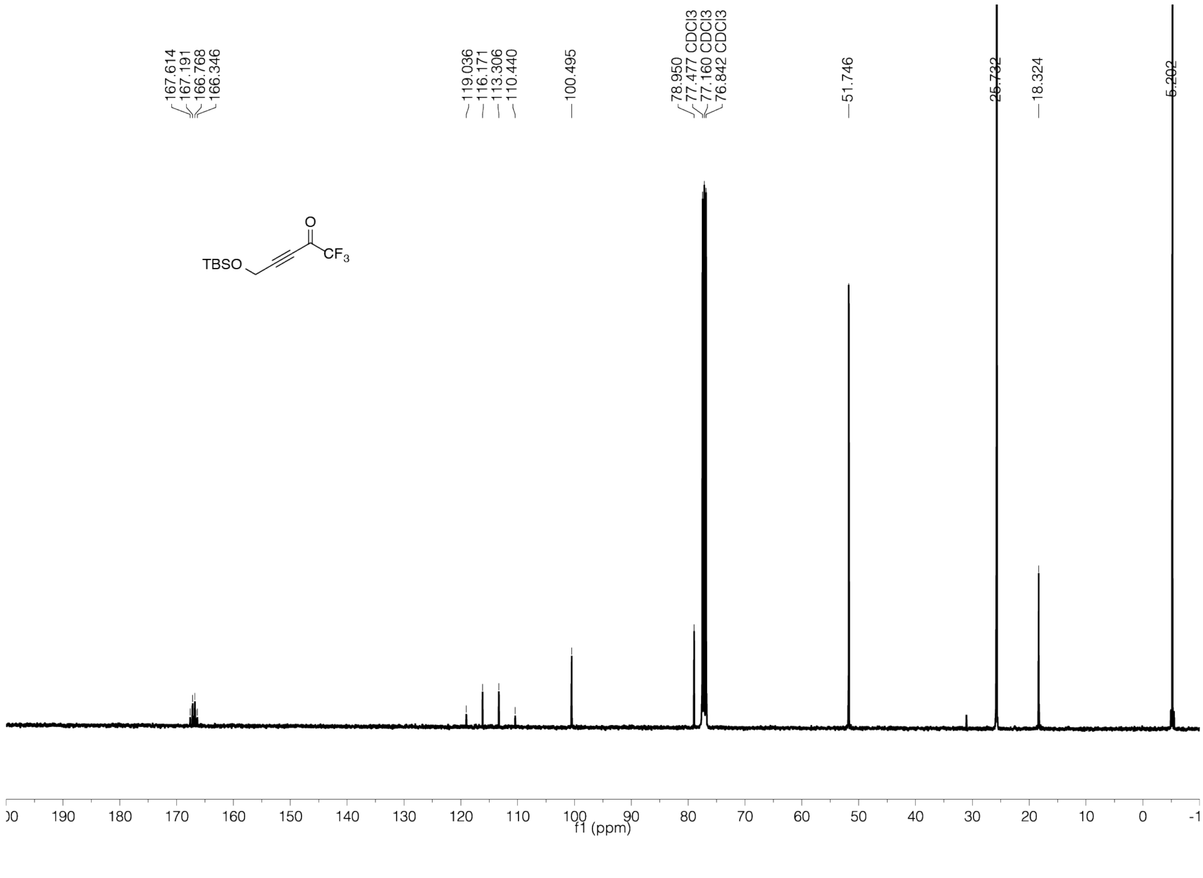

^{19}F NMR (376MHz, $CDCl_3$)

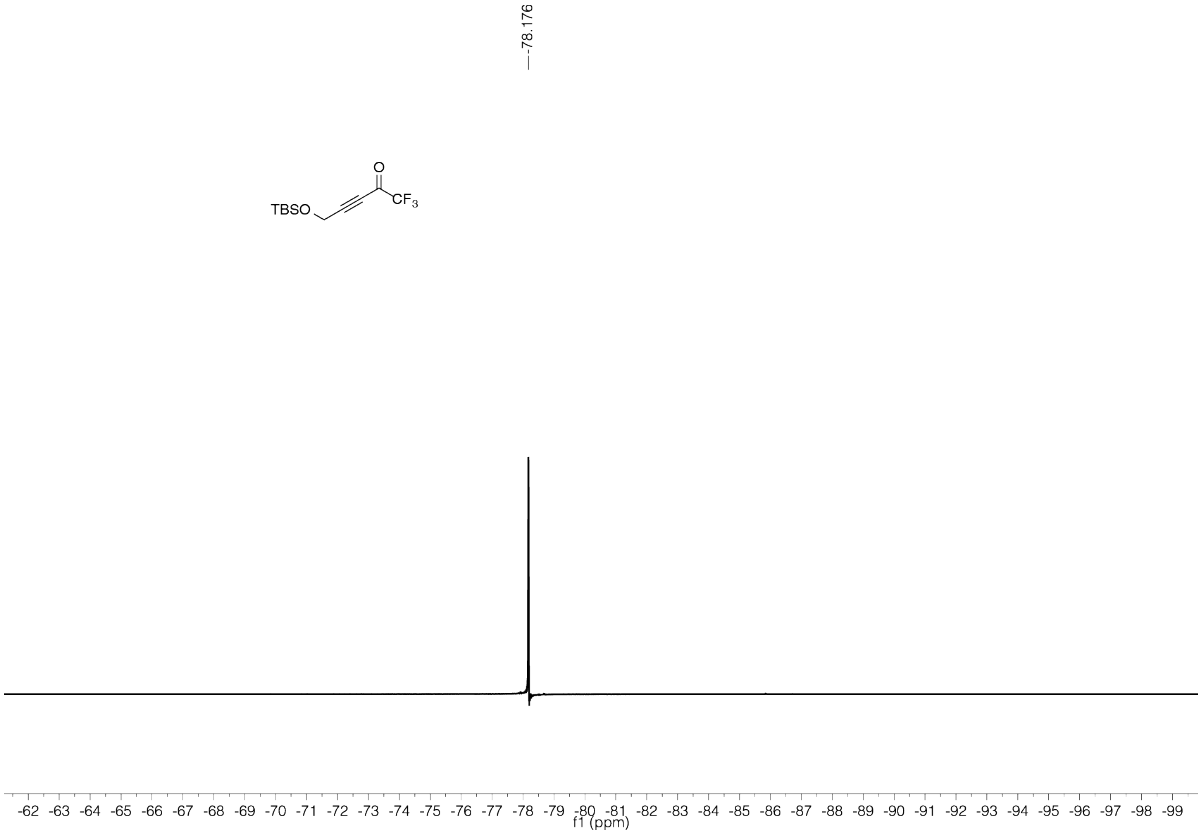

8-((*Tert*-Butyldiphenylsilyl)oxy)-1,1,1-trifluorooct-3-yn-2-one (4d):

^{1}H NMR (400MHz, $CDCl_3$)

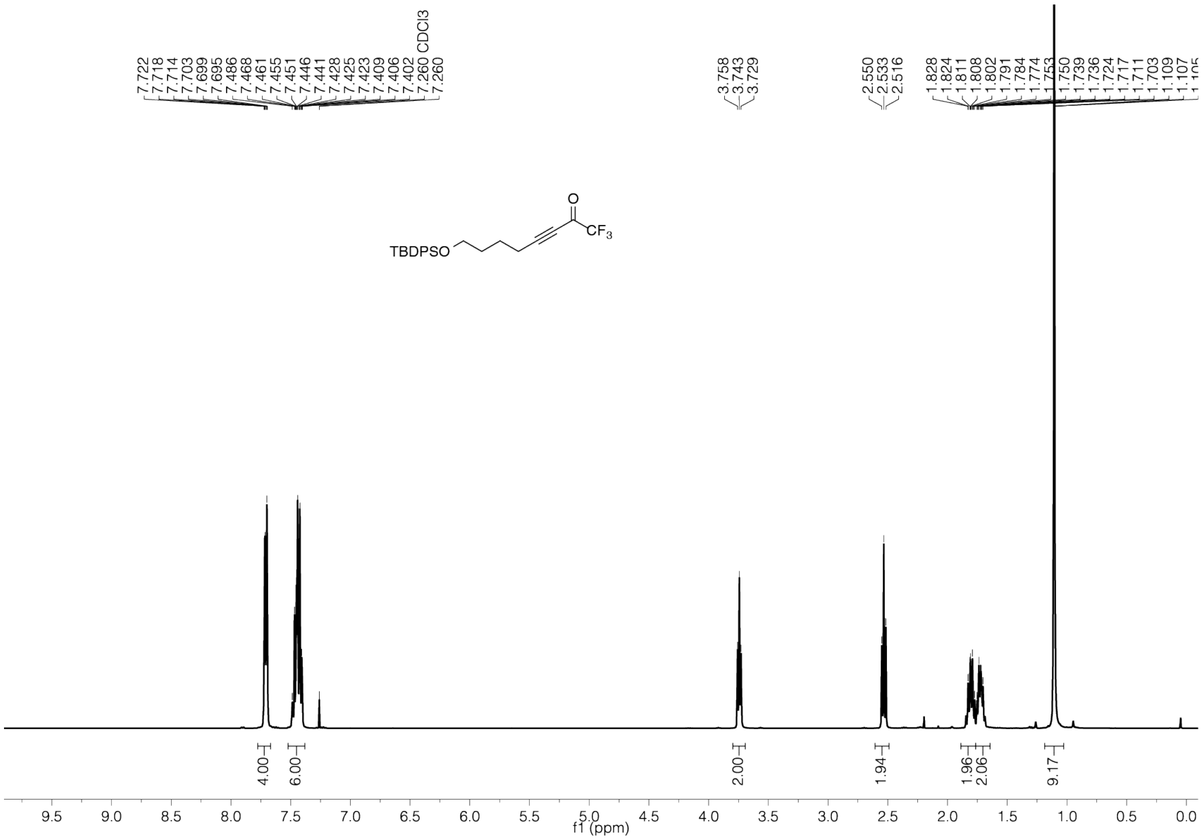

^{13}C NMR (100MHz, $CDCl_3$)

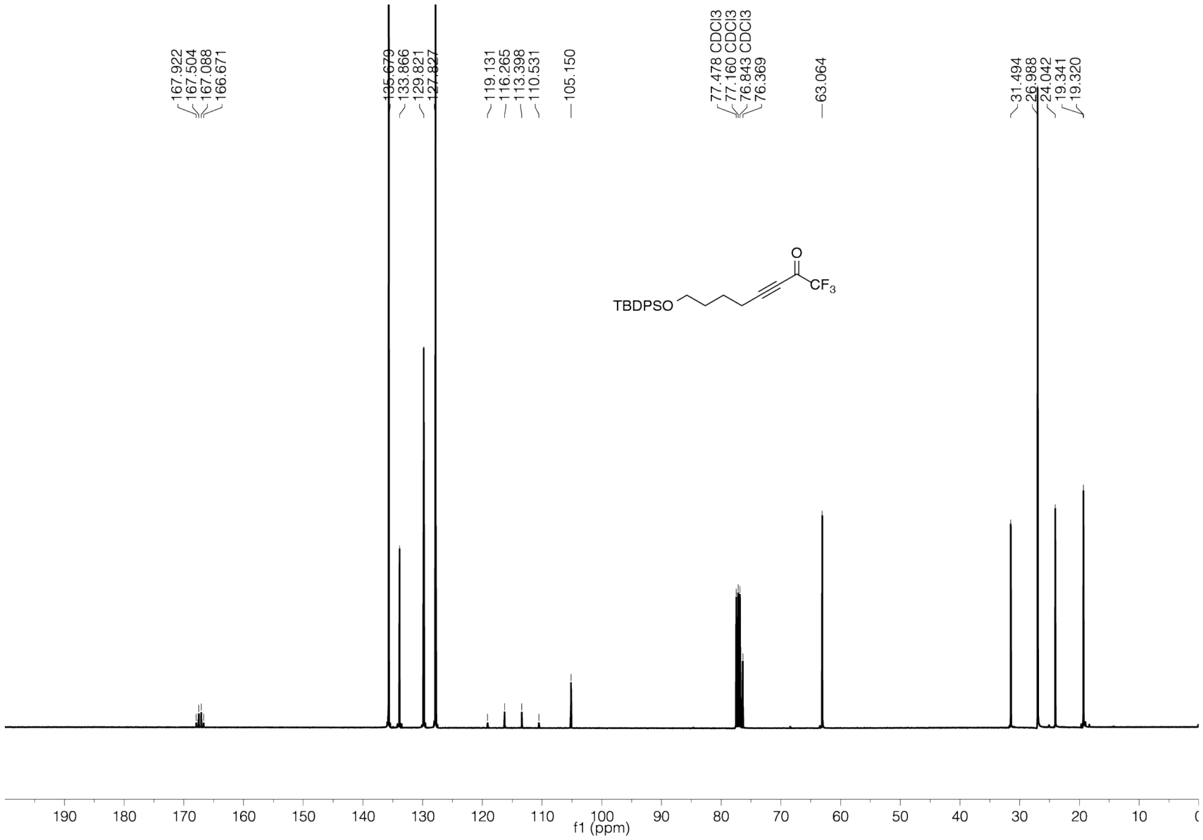

¹⁹F NMR (376MHz, $CDCl_3$)

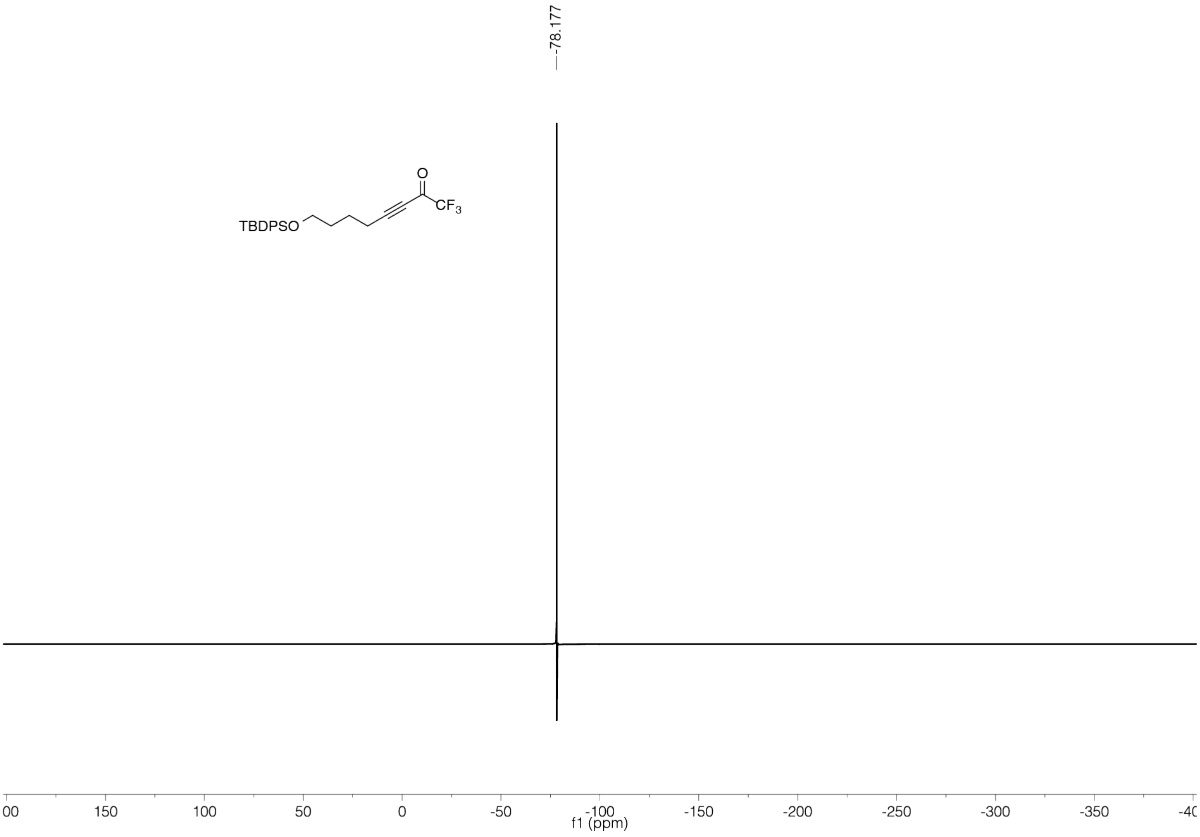

8-Chloro-1,1,1-trifluorooct-3-yn-2-one (4e):

^{1}H NMR (400MHz, $CDCl_3$)

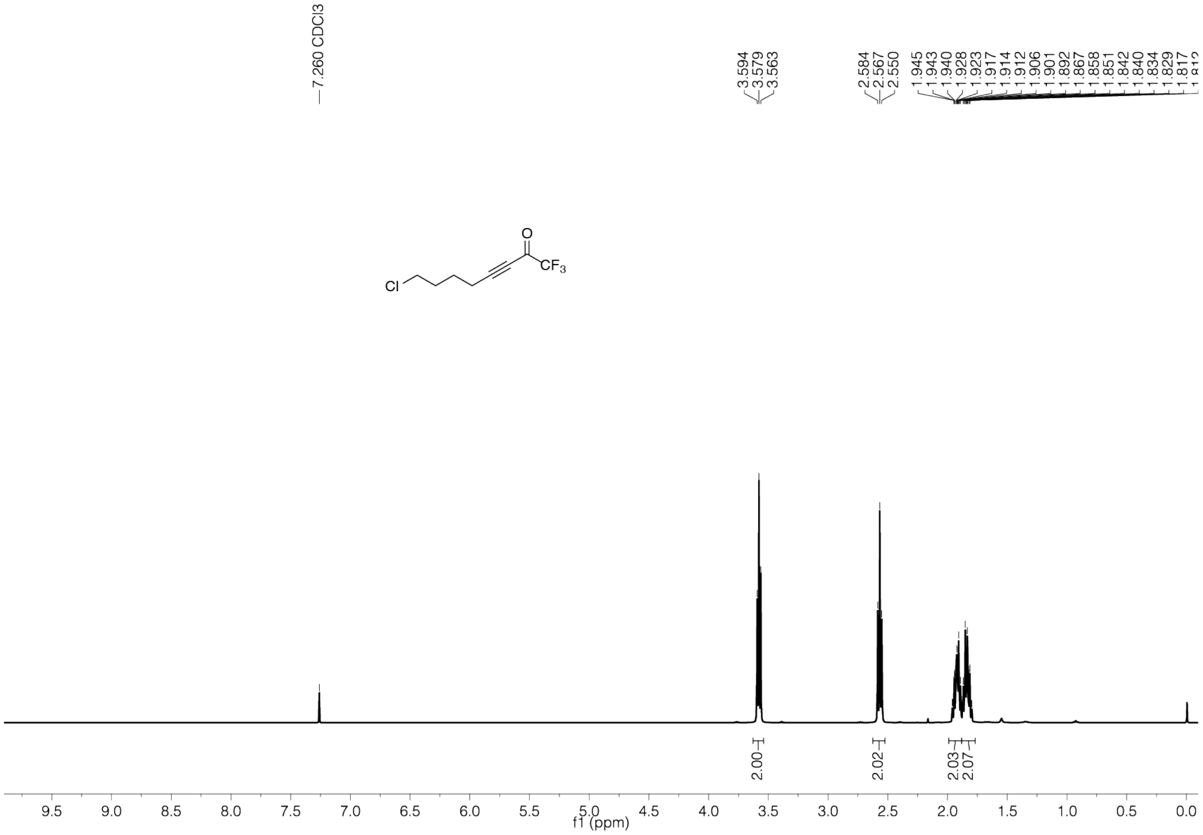

^{13}C NMR (100MHz, $CDCl_3$)

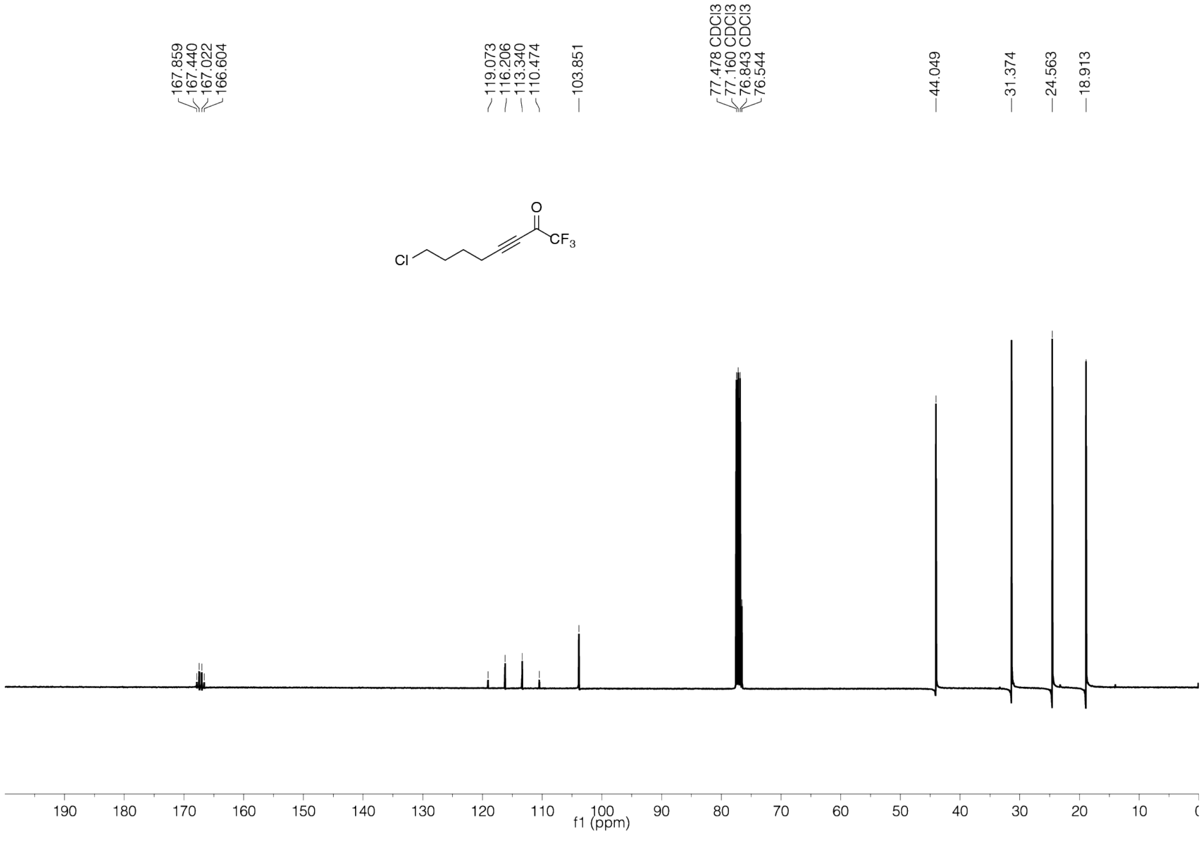

^{19}F NMR (376MHz, $CDCl_3$)

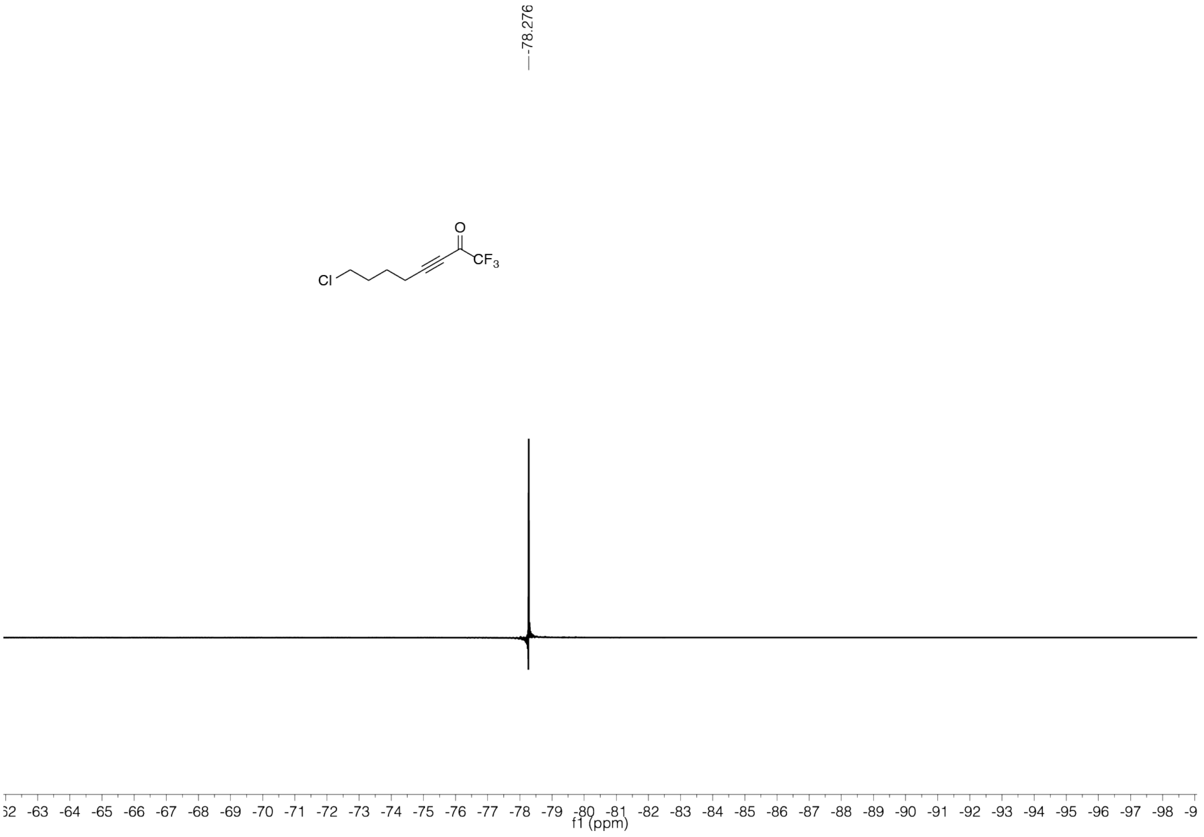

4-(4-(1,3-Dioxolan-2-yl)phenyl)-1,1,1-trifluorobut-3-yn-2-one (4h):

^{1}H NMR (400MHz, $CDCl_3$)

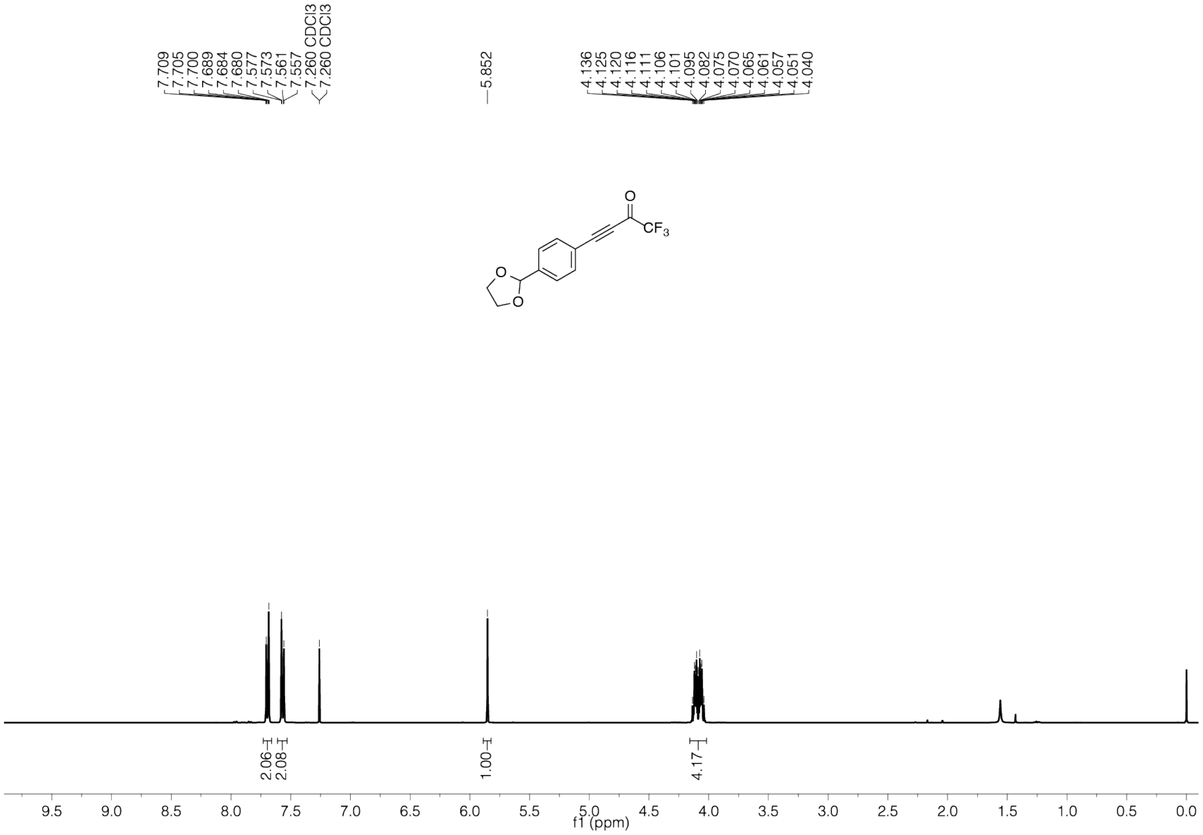

^{13}C NMR (100MHz, $CDCl_3$)

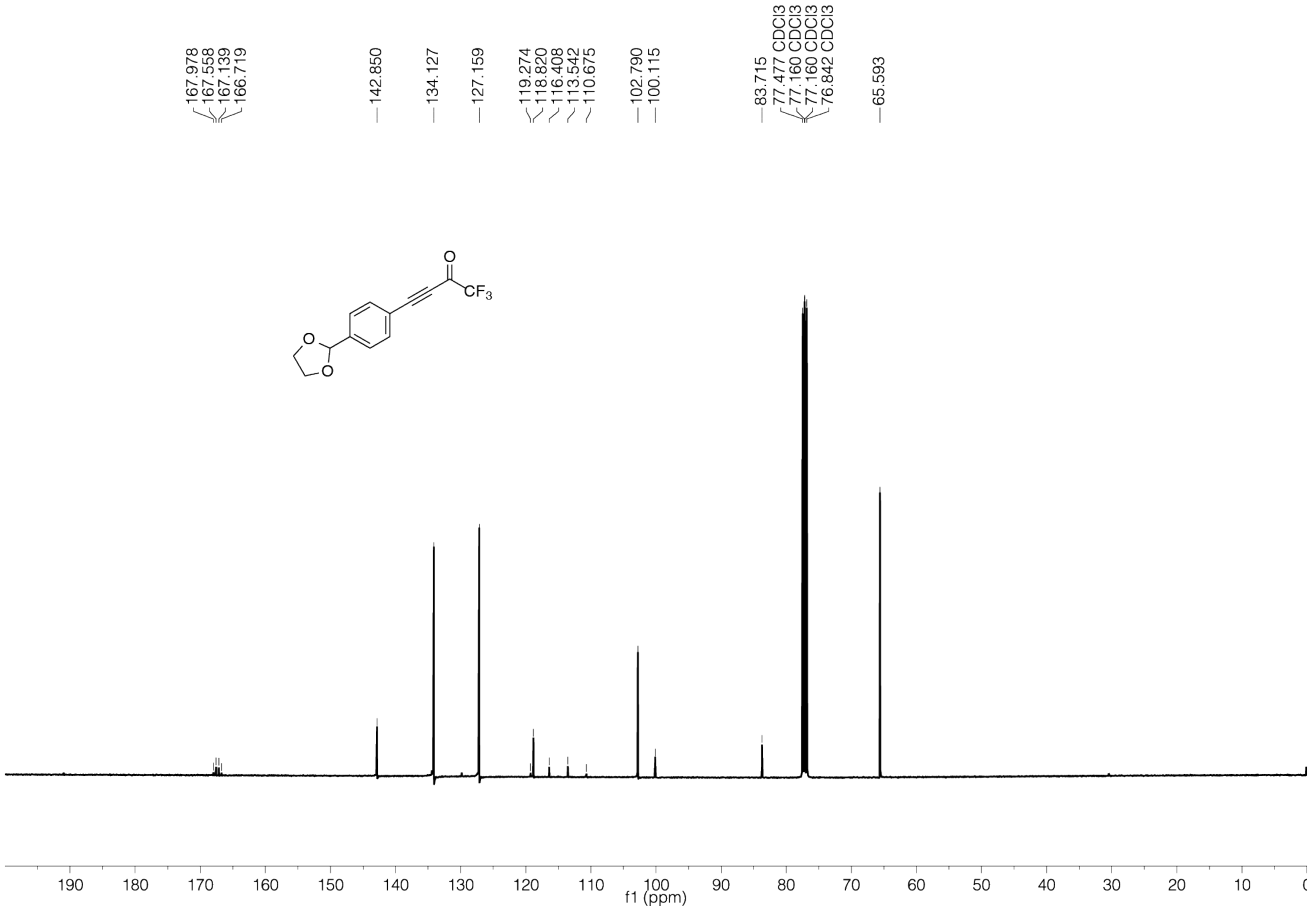

^{19}F NMR (376MHz, $CDCl_3$)

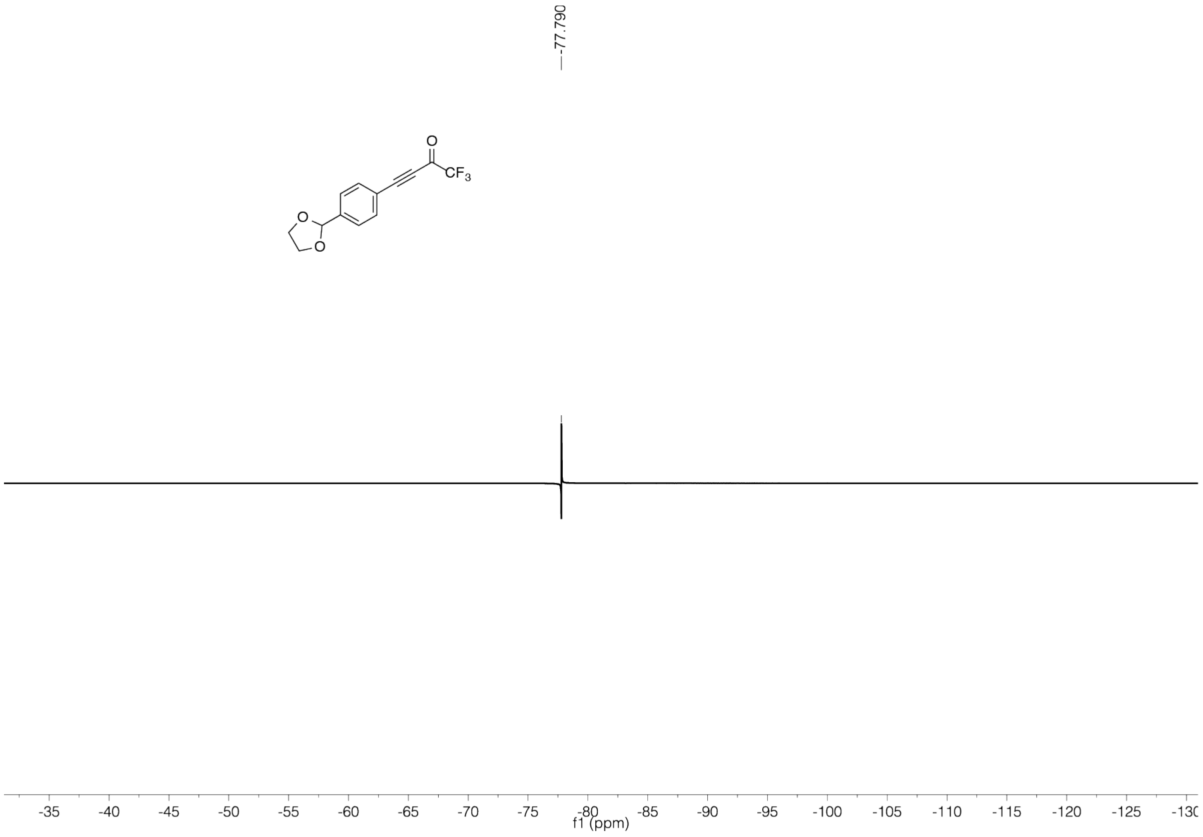

1,1,1-Trifluoro-4-(4-(morpholine-4-carbonyl)phenyl)but-3-yn-2-one hydrate (4i•H_2O):

^{1}H NMR (400MHz, DMSO-*d*6)

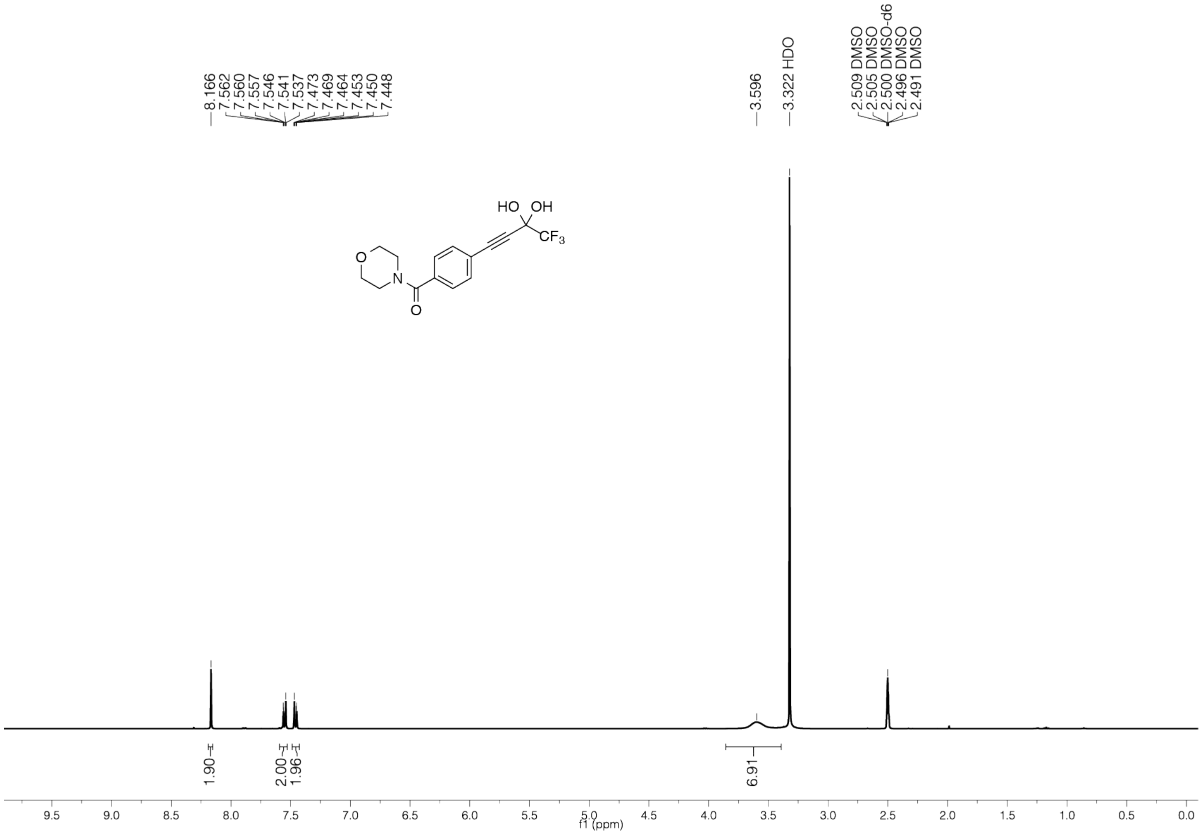

^{13}C NMR (100MHz, DMSO-*d*6)

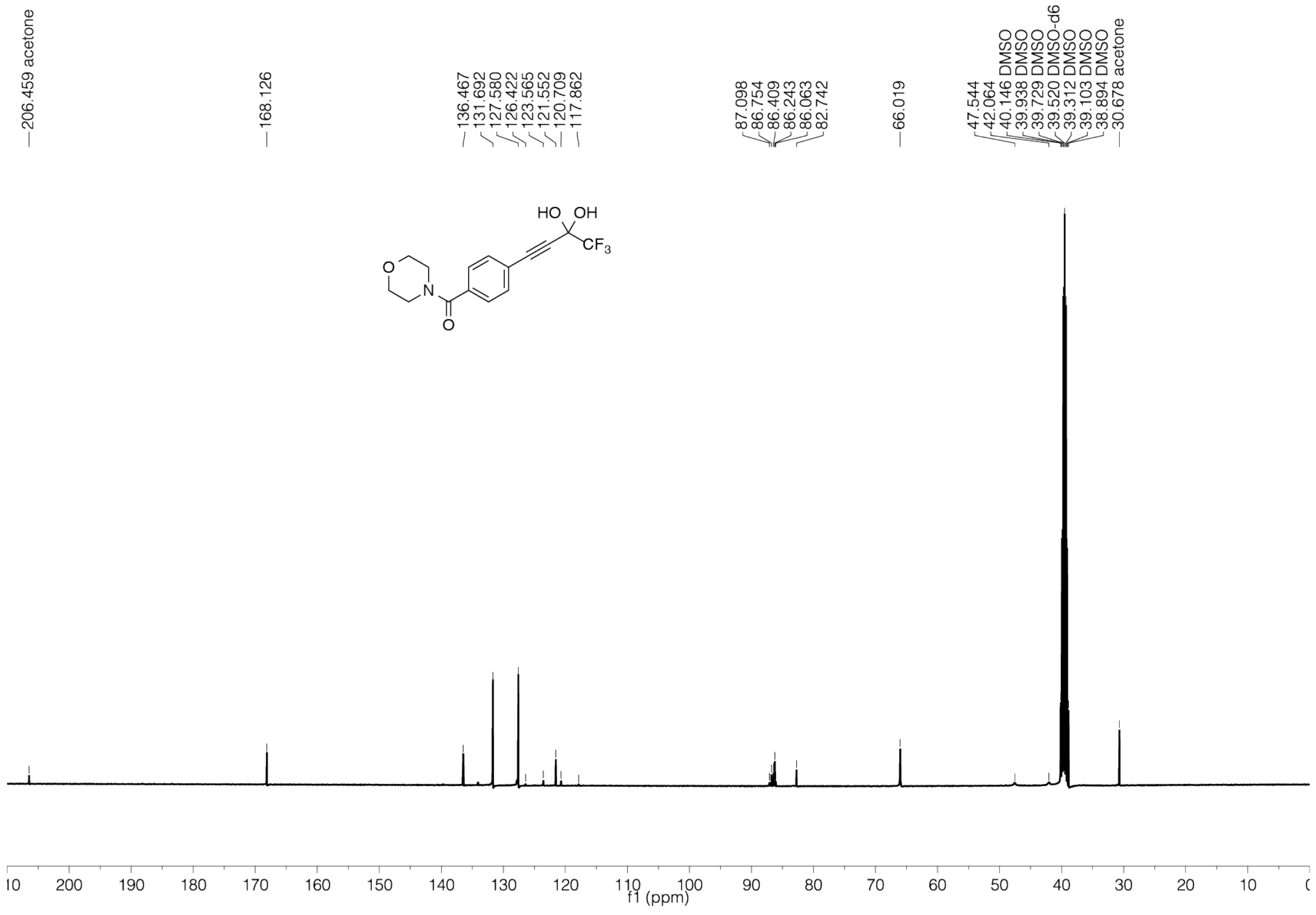

^{19}F NMR (376MHz, DMSO-d_6)

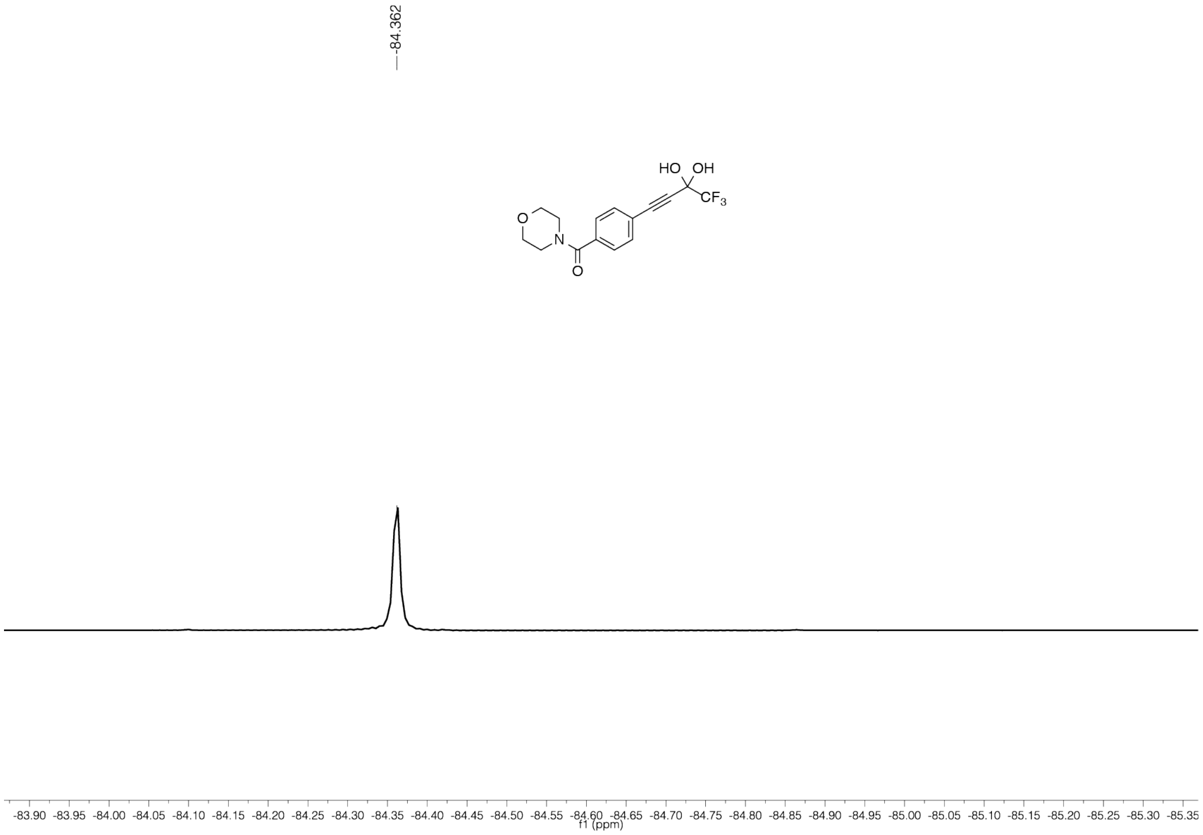

1,1,1-Trifluoro-4-(thiophen-3-yl)but-3-yn-2-one (4j):

^{1}H NMR (400MHz, $CDCl_3$)

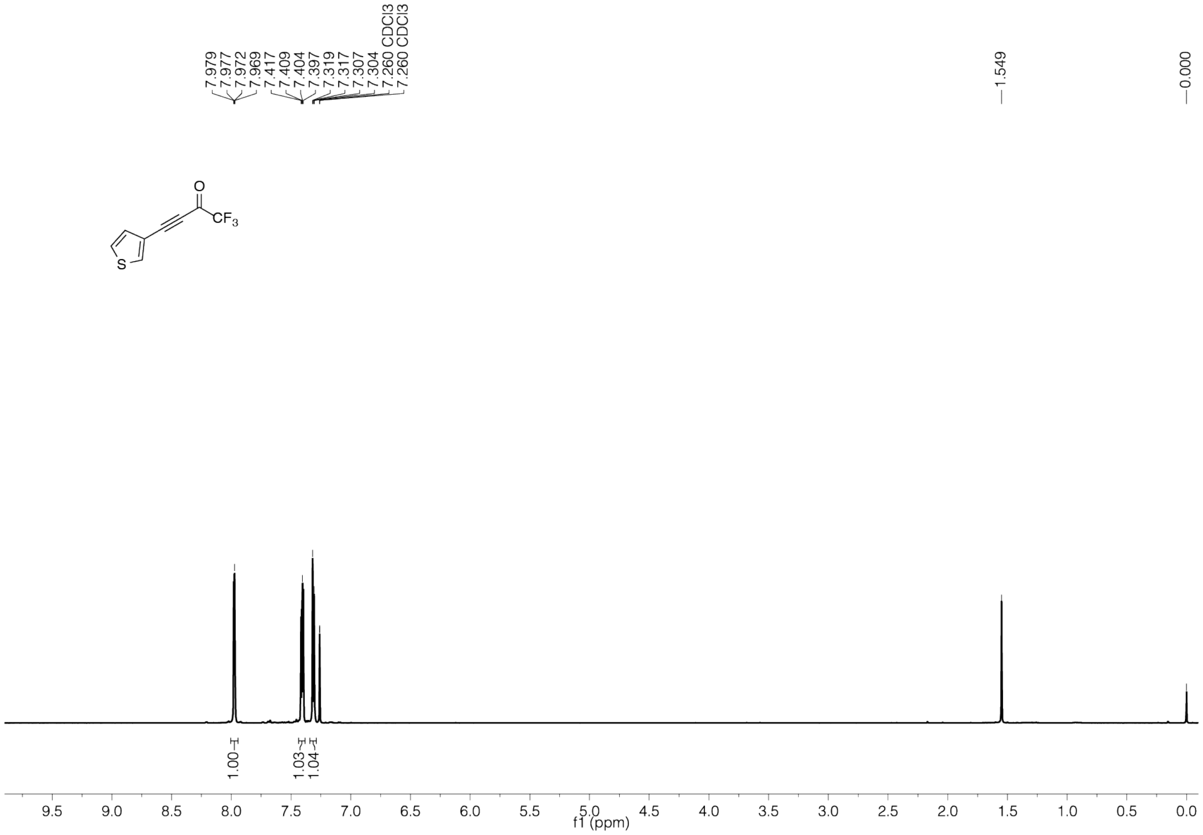

^{13}C NMR (100MHz, $CDCl_3$)

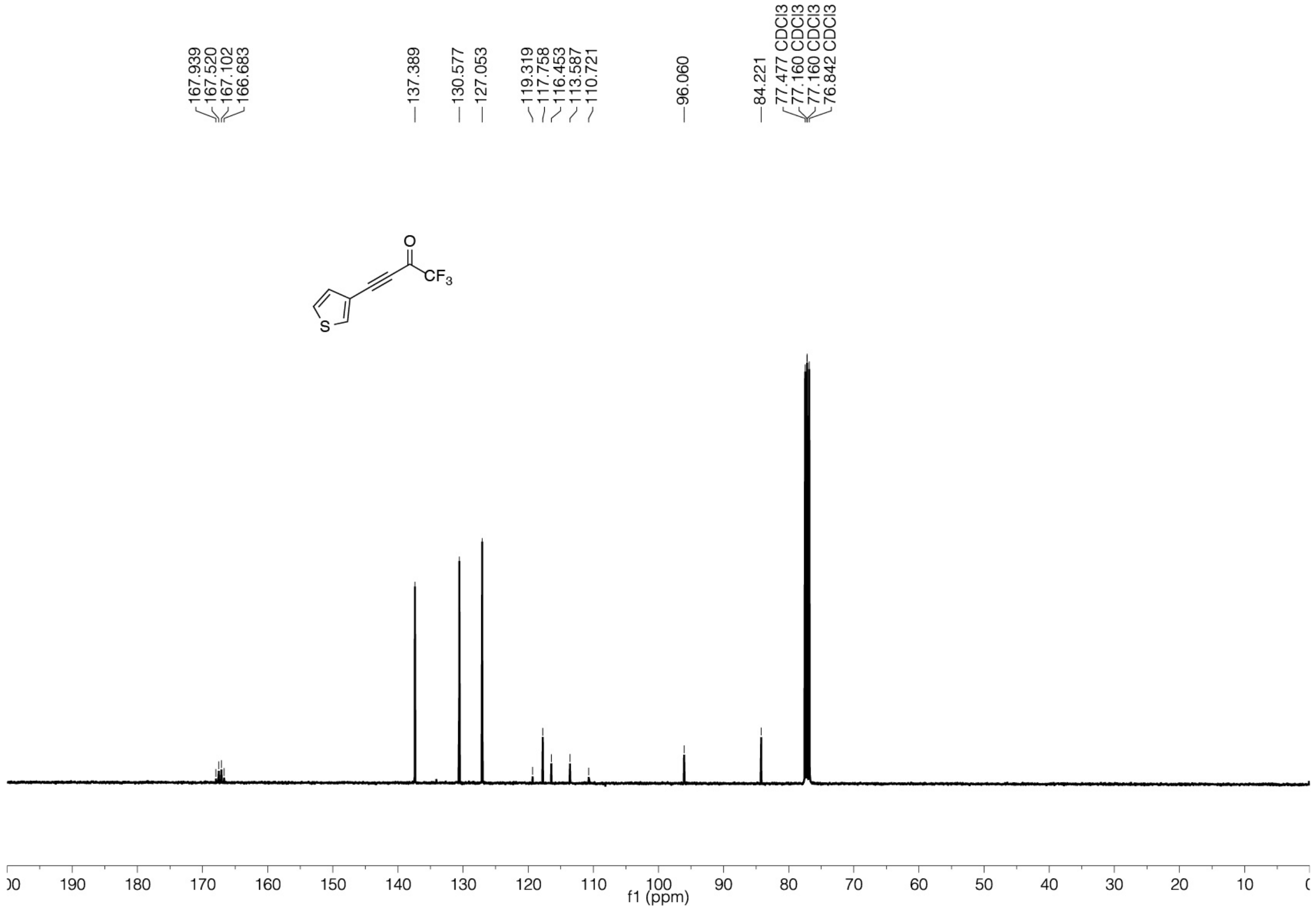

^{19}F NMR (376MHz, $CDCl_3$)

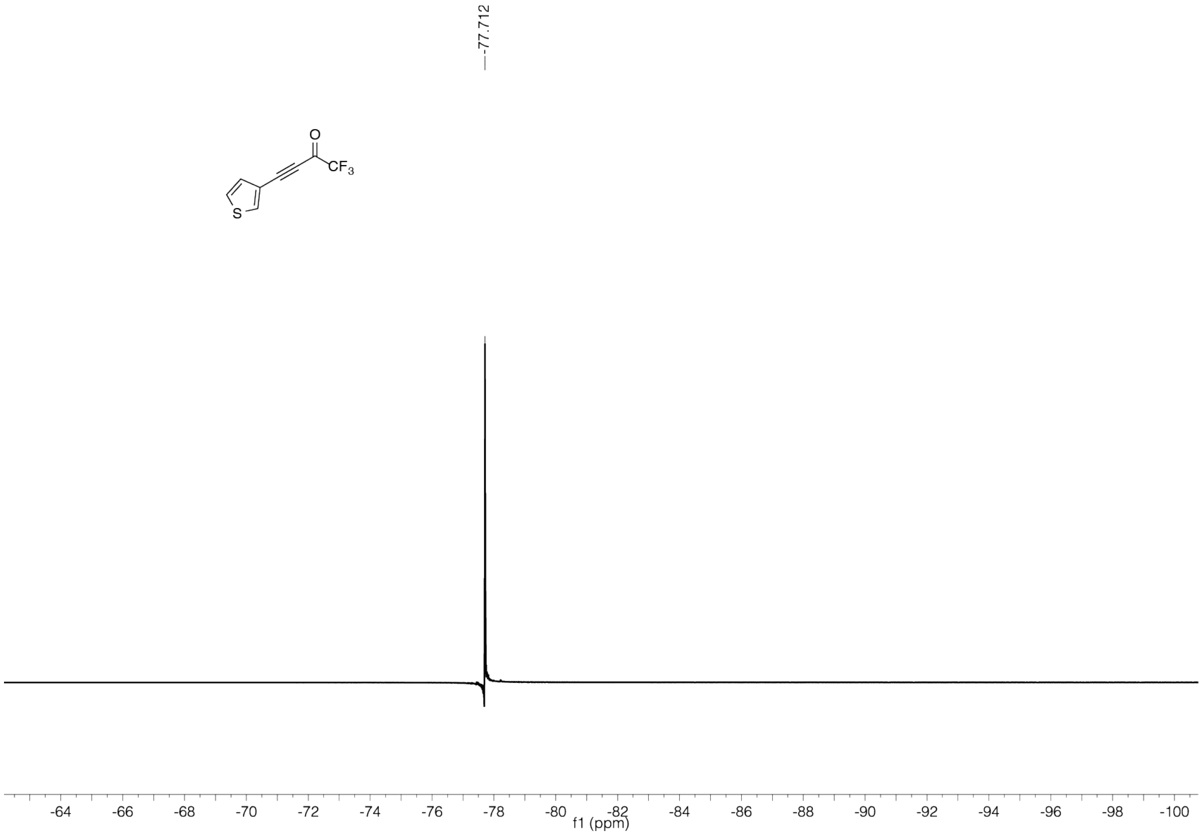

4-Cyclohexyl-1,1,1-trifluorobut-3-yn-2-one (4k):

^{1}H NMR (400MHz, $CDCl_3$)

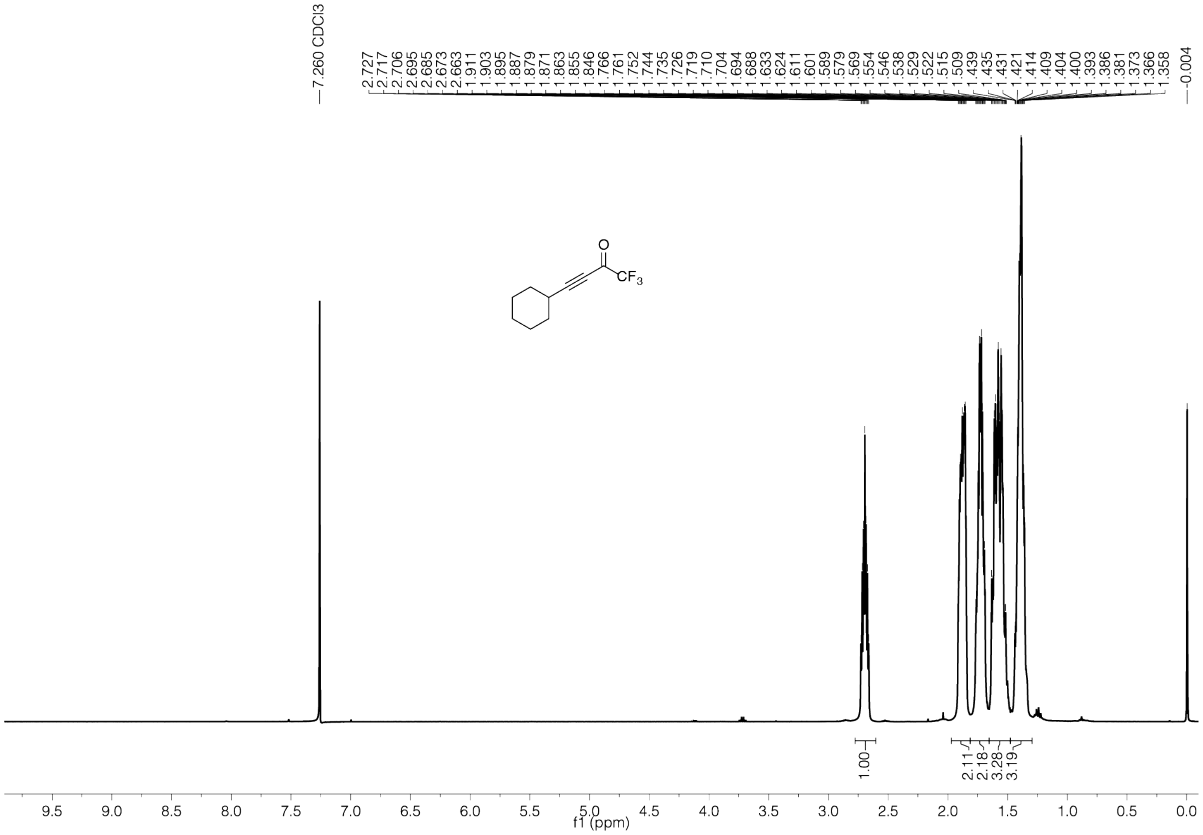

^{13}C NMR (100MHz, $CDCl_3$)

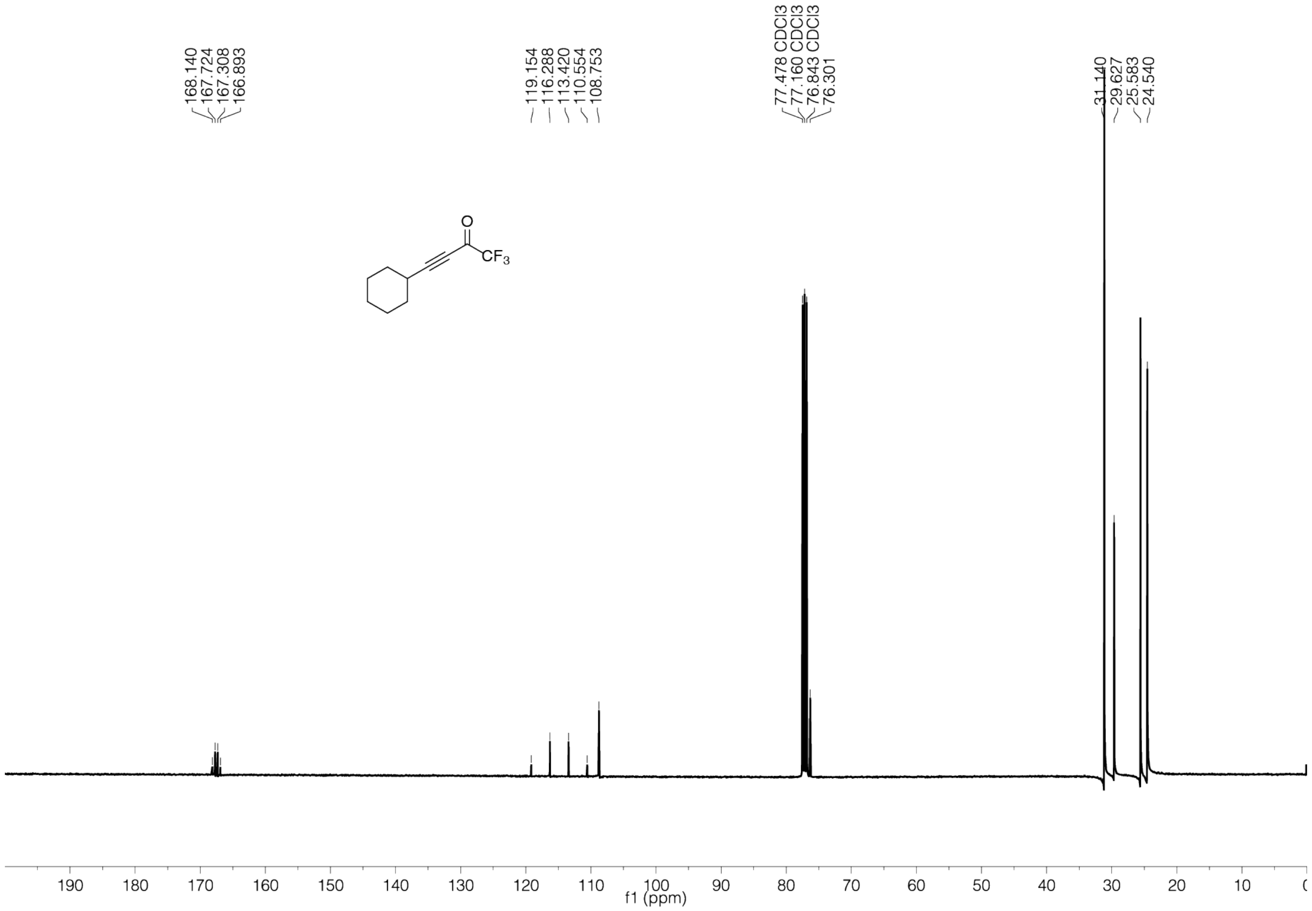

^{19}F NMR (376MHz, $CDCl_3$)

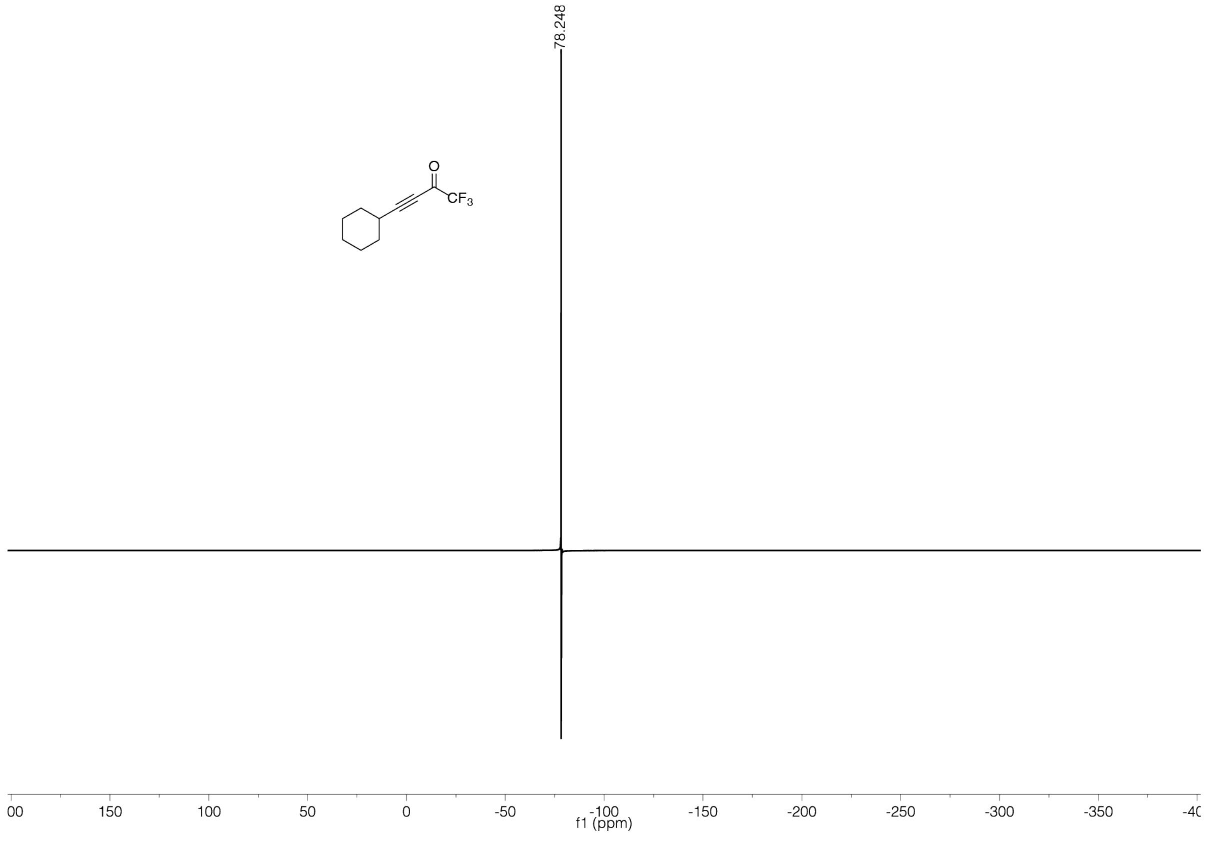

4-(Cyclohex-1-en-1-yl)-1,1,1-trifluorobut-3-yn-2-one (4l):

^{1}H NMR (400MHz, $CDCl_3$)

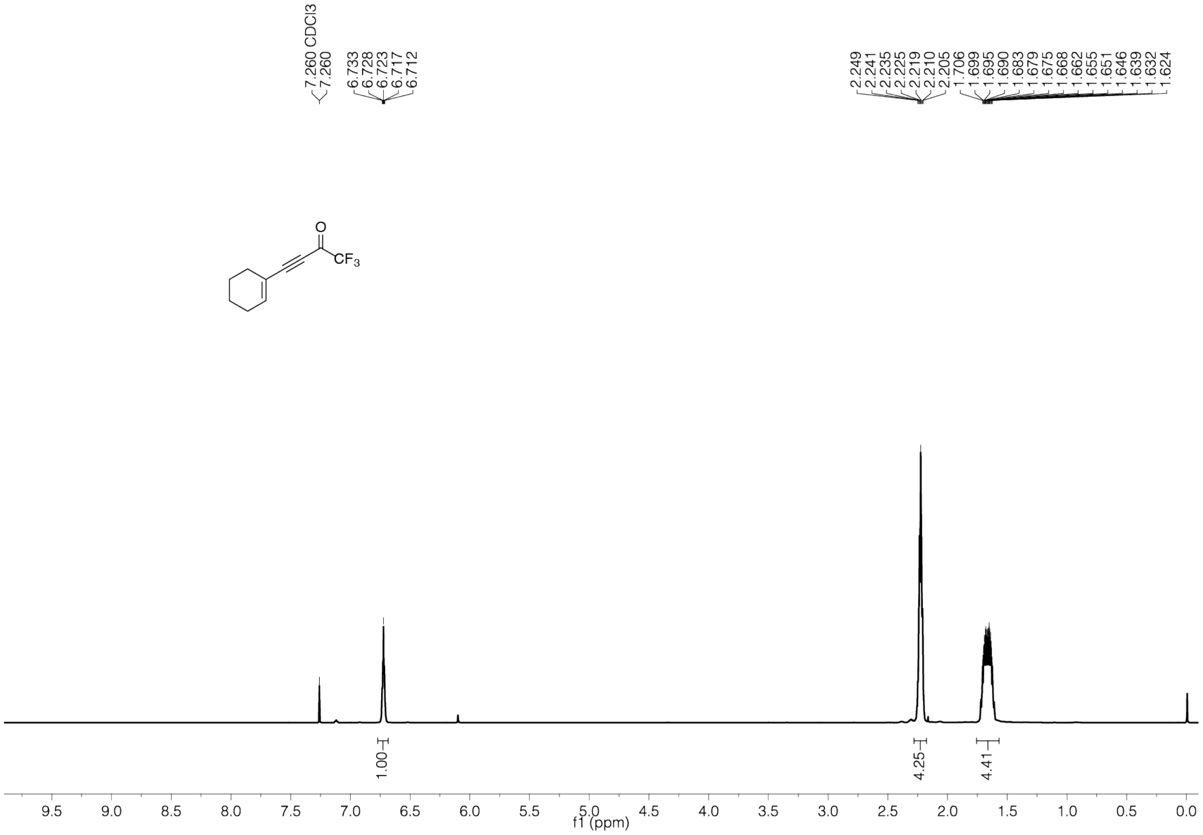

^{13}C NMR (100MHz, $CDCl_3$)

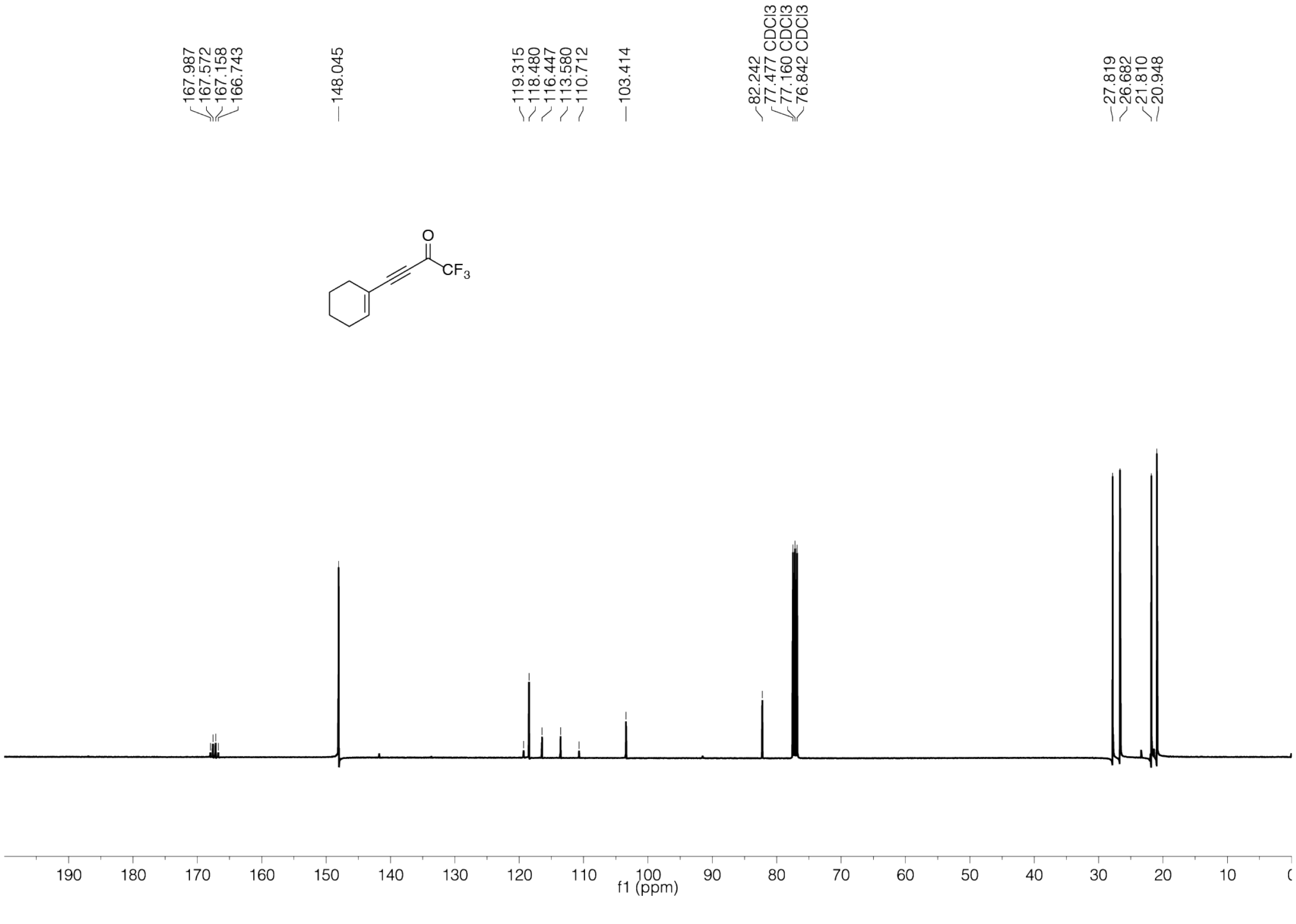

^{19}F NMR (376MHz, $CDCl_3$)

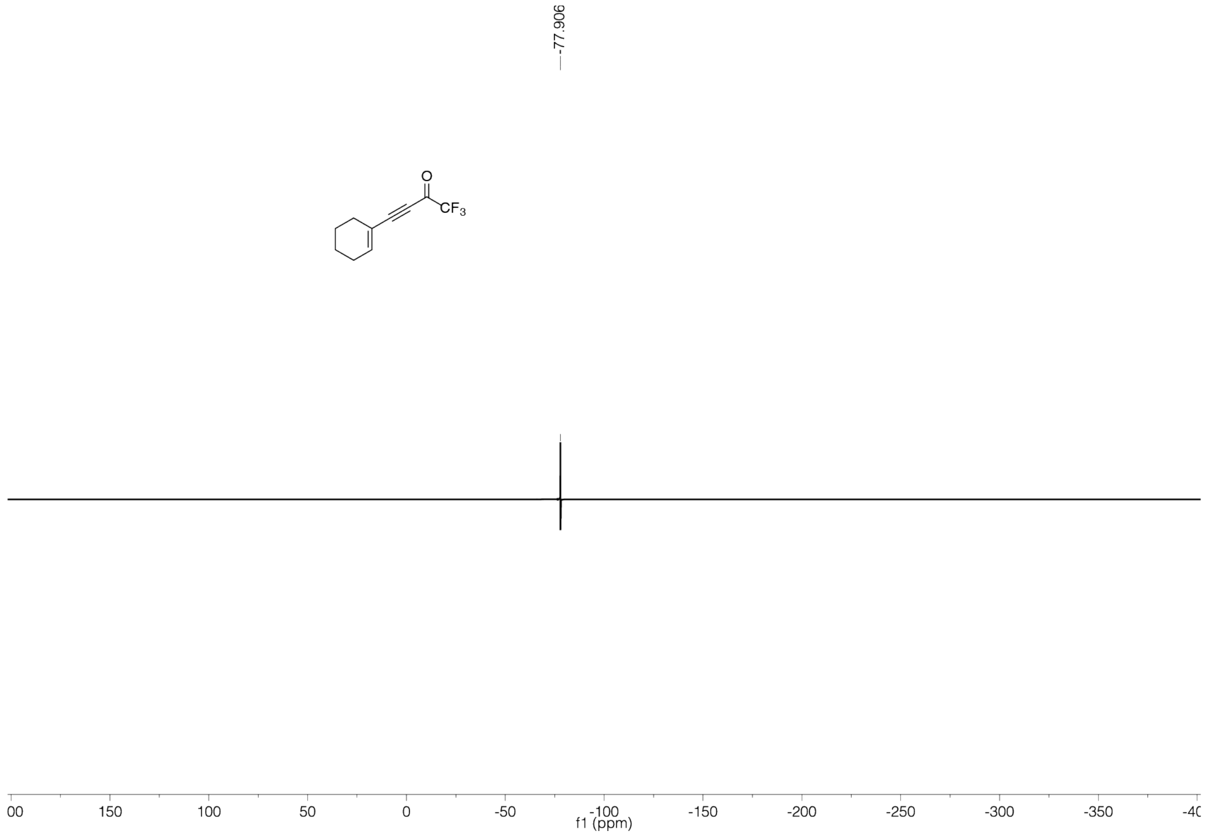

1,1-Difluorodec-3-yn-2-one (11a):

^{1}H NMR (400MHz, $CDCl_3$)

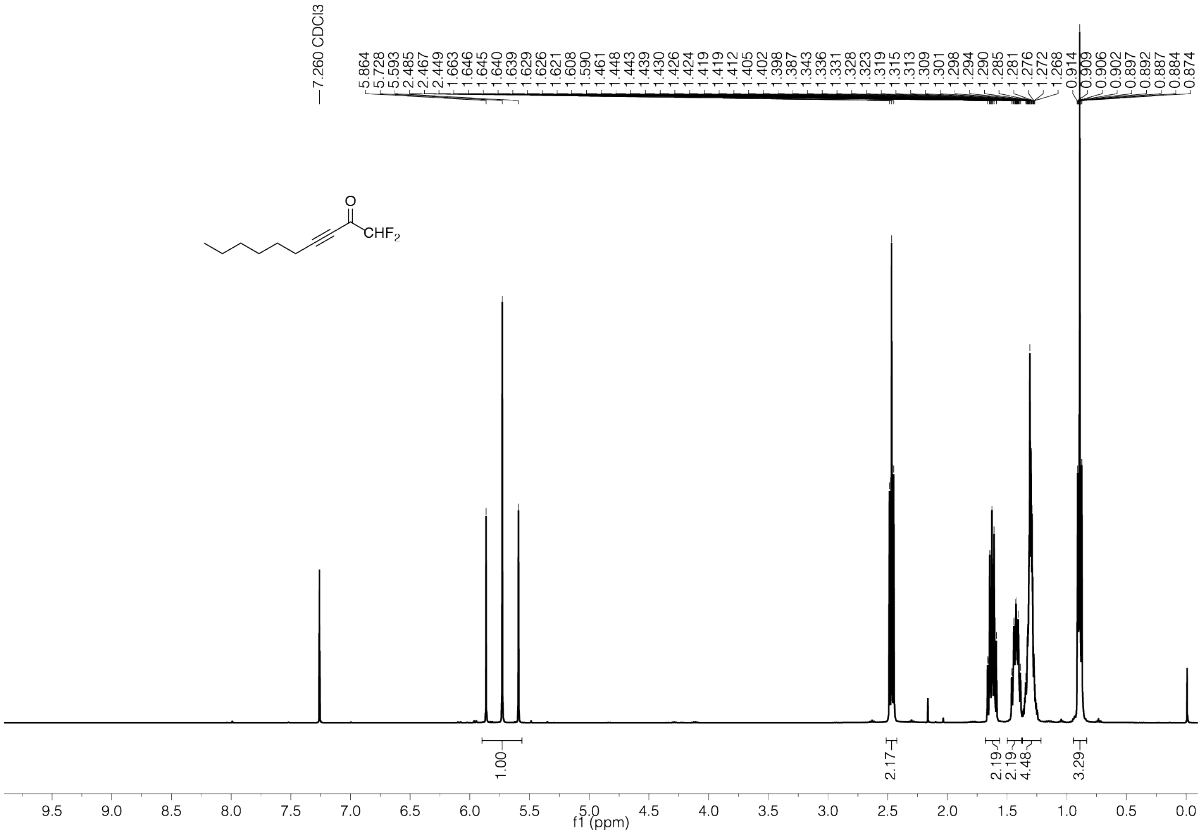

^{13}C NMR (100MHz, $CDCl_3$)

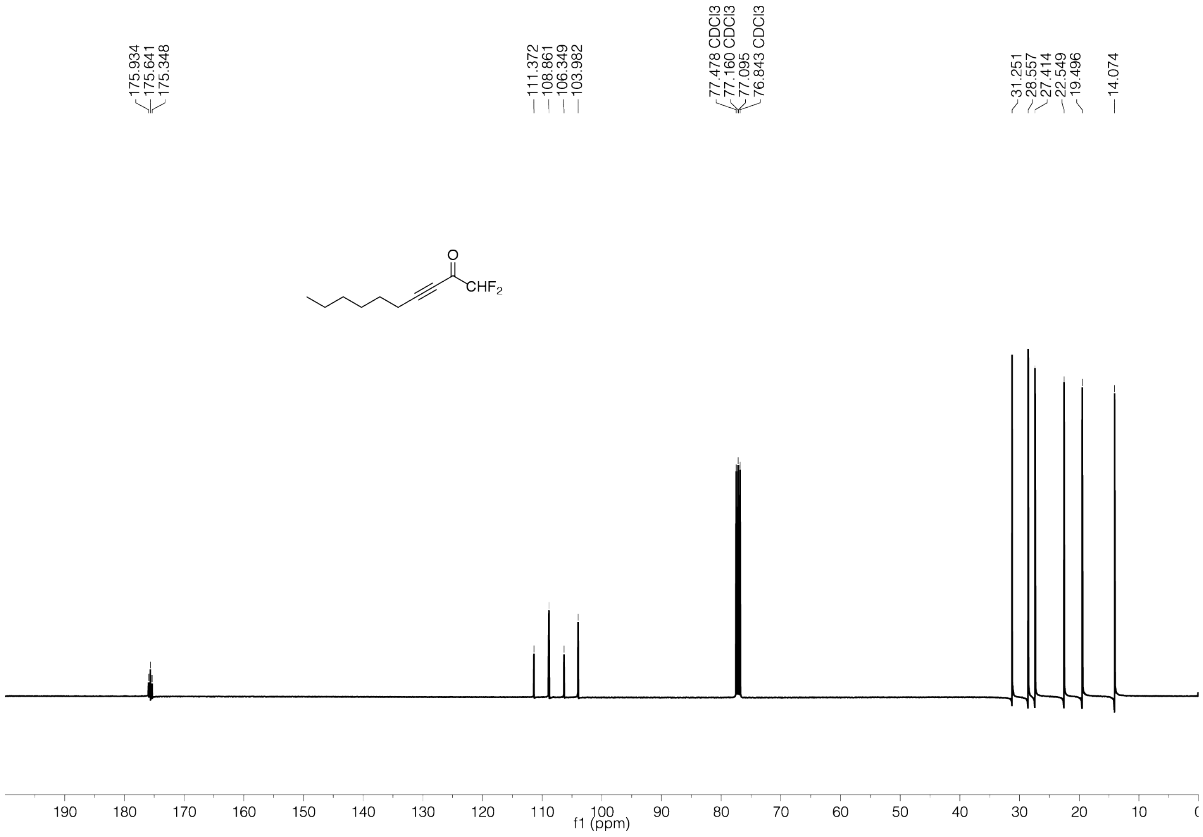

^{19}F NMR (376MHz, $CDCl_3$)

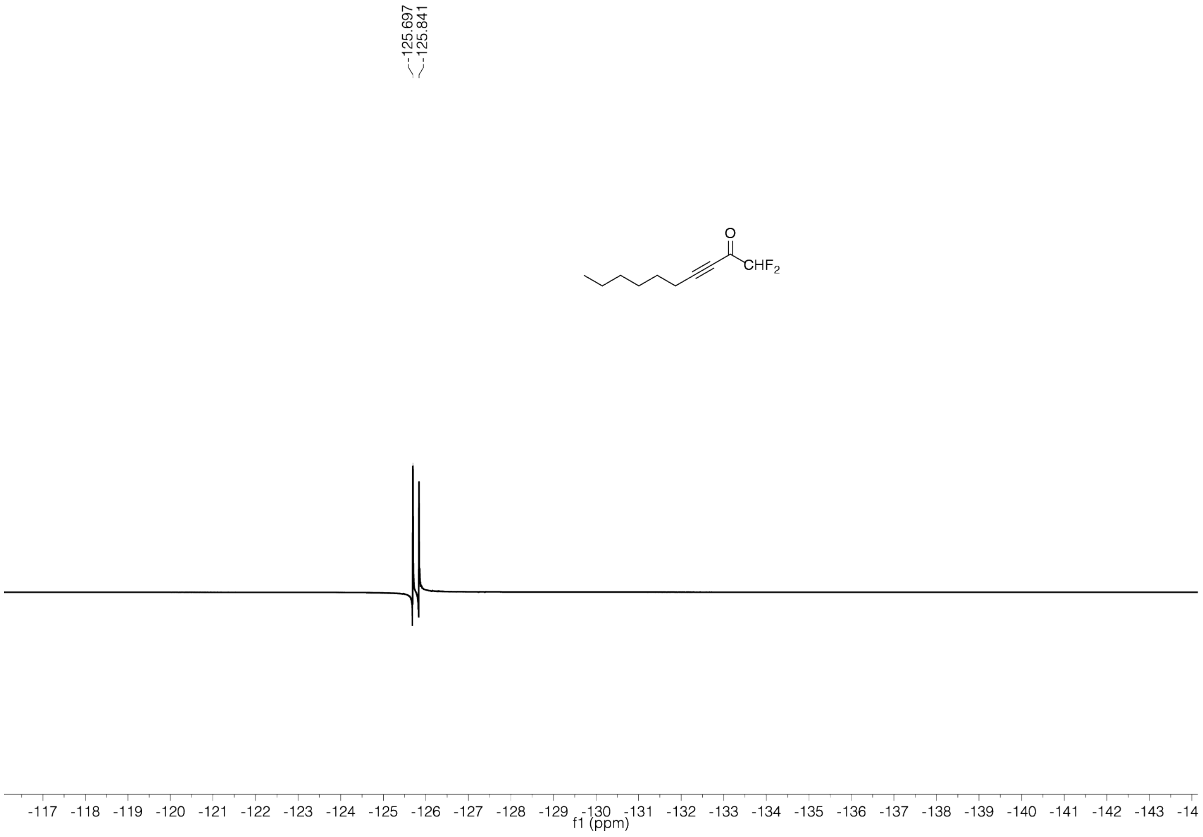

1-Bromo-1,1-difluorodec-3-yn-2-one (11c):

^{1}H NMR (400MHz, $CDCl_3$)

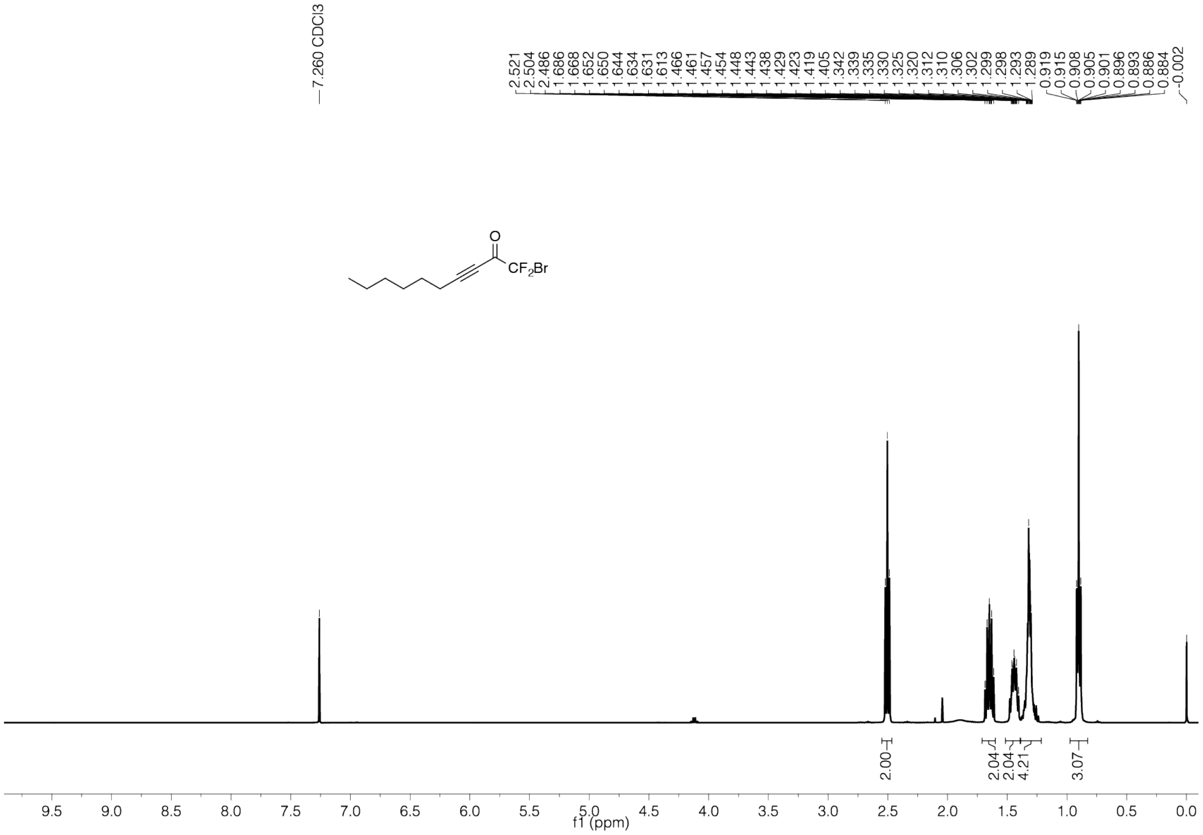

^{13}C NMR (100MHz, $CDCl_3$)

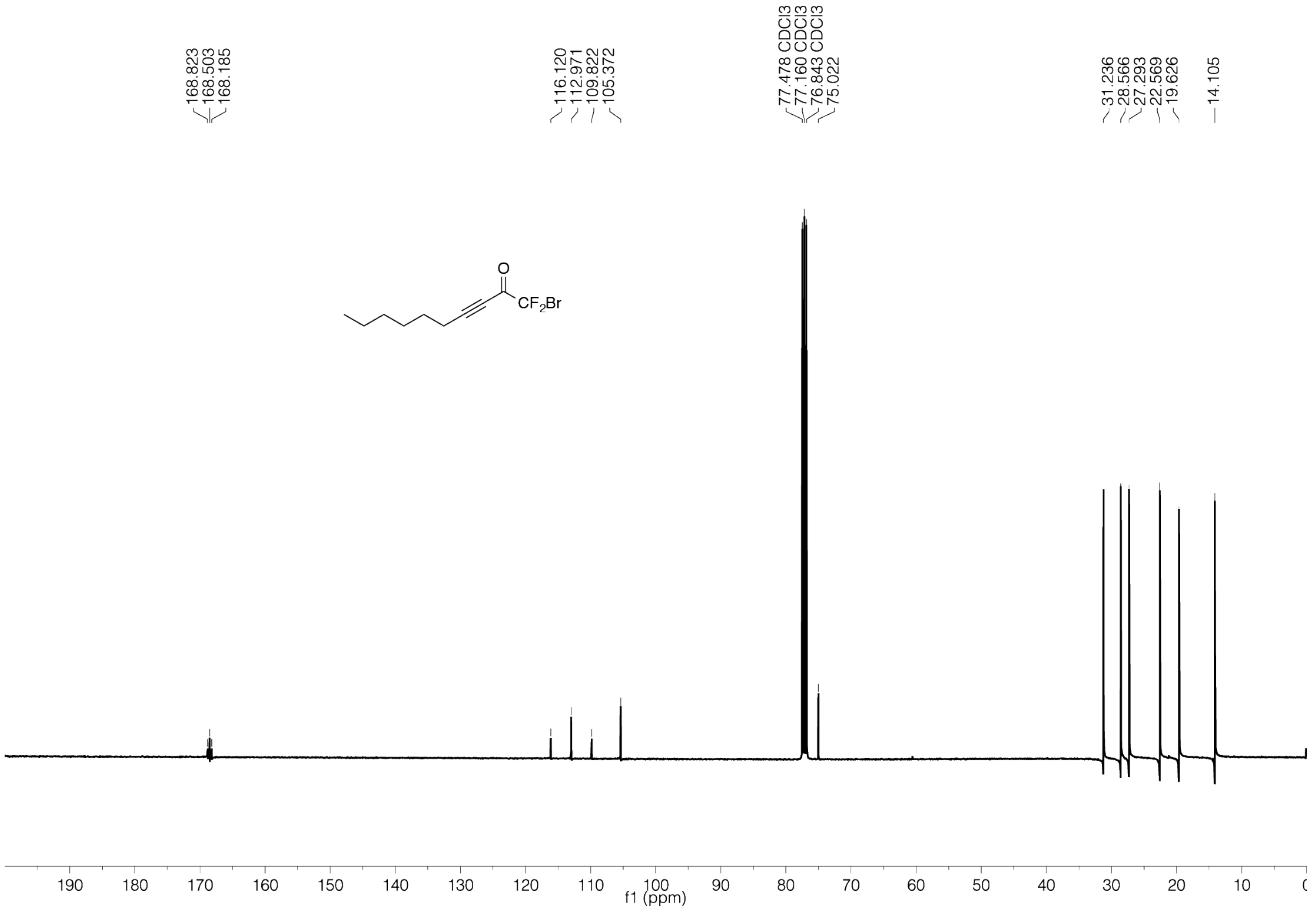

^{19}F NMR (376MHz, $CDCl_3$)

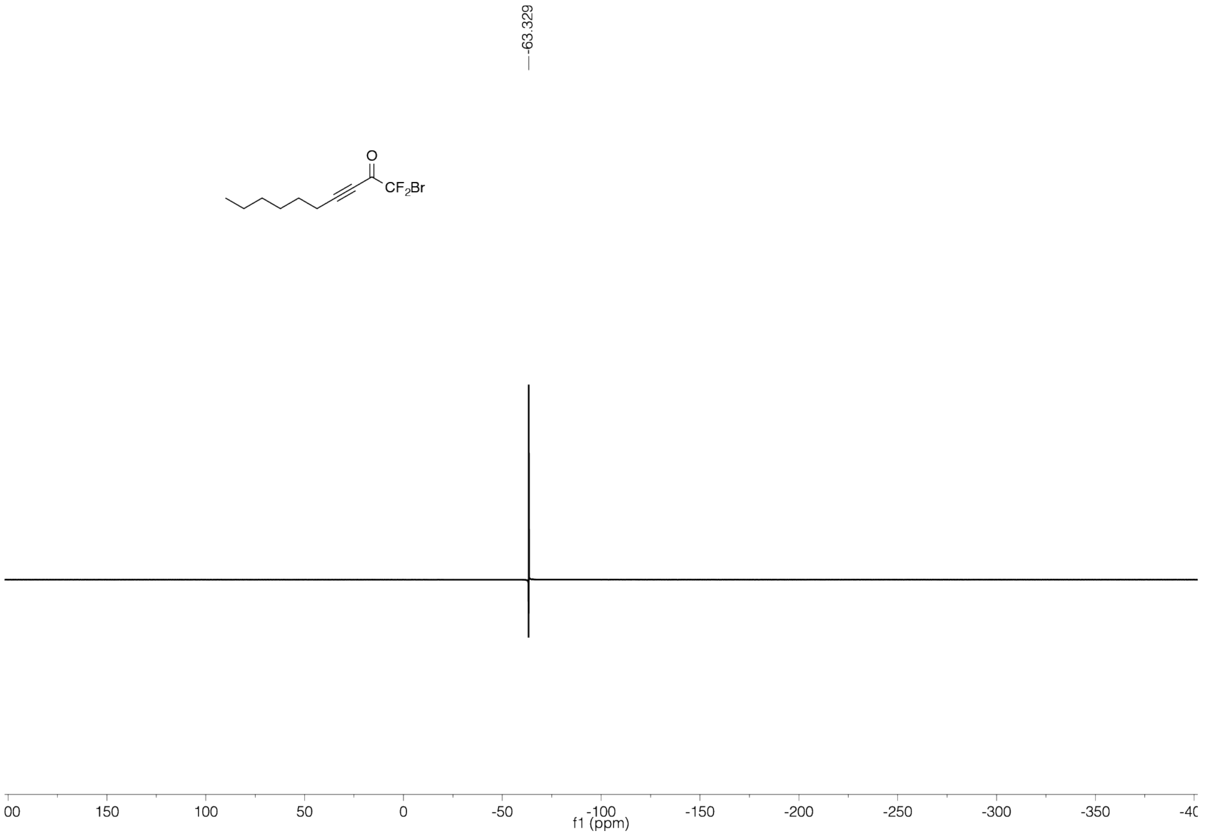

N,N'-((1R,2R)-cyclohexane-1,2-diyl)bis(N-methoxy-3,3,3-triphenylpropanamide) (10):

^{1}H NMR (400MHz, $CDCl_3$)

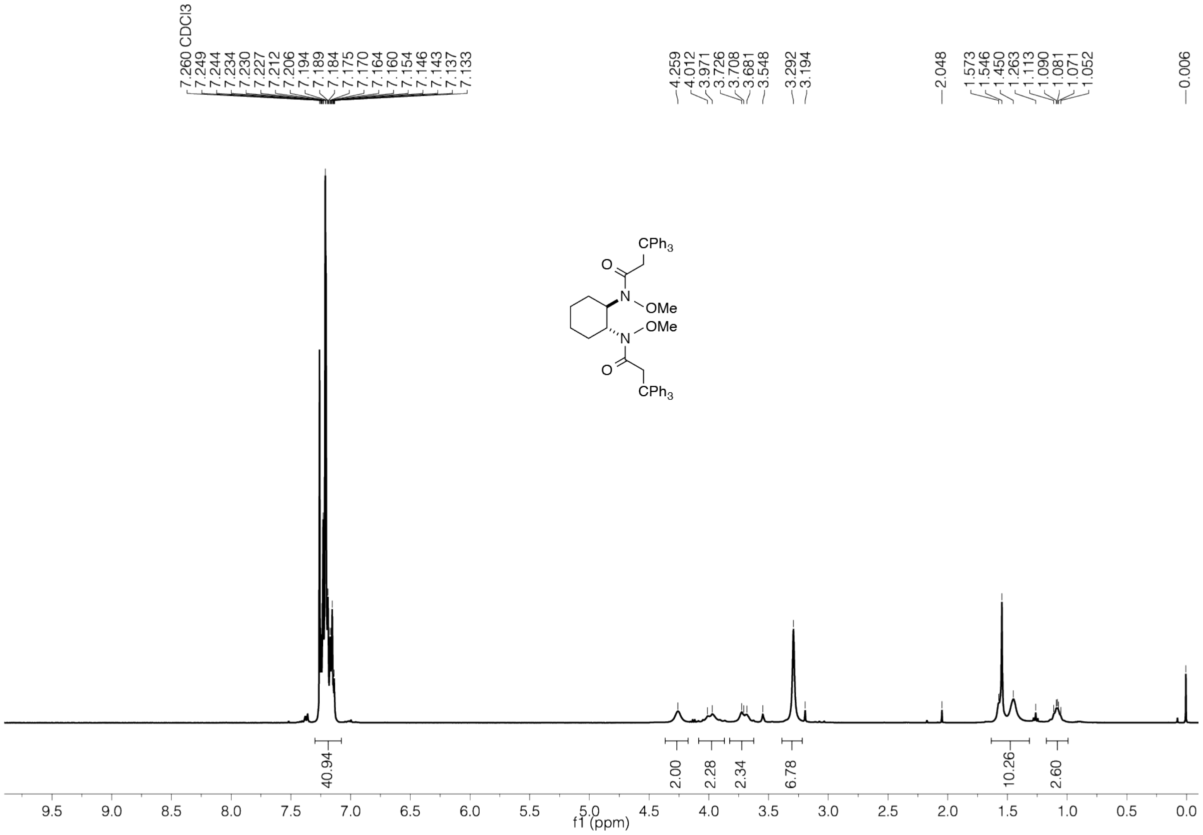

^{13}C NMR (100MHz, $CDCl_3$)

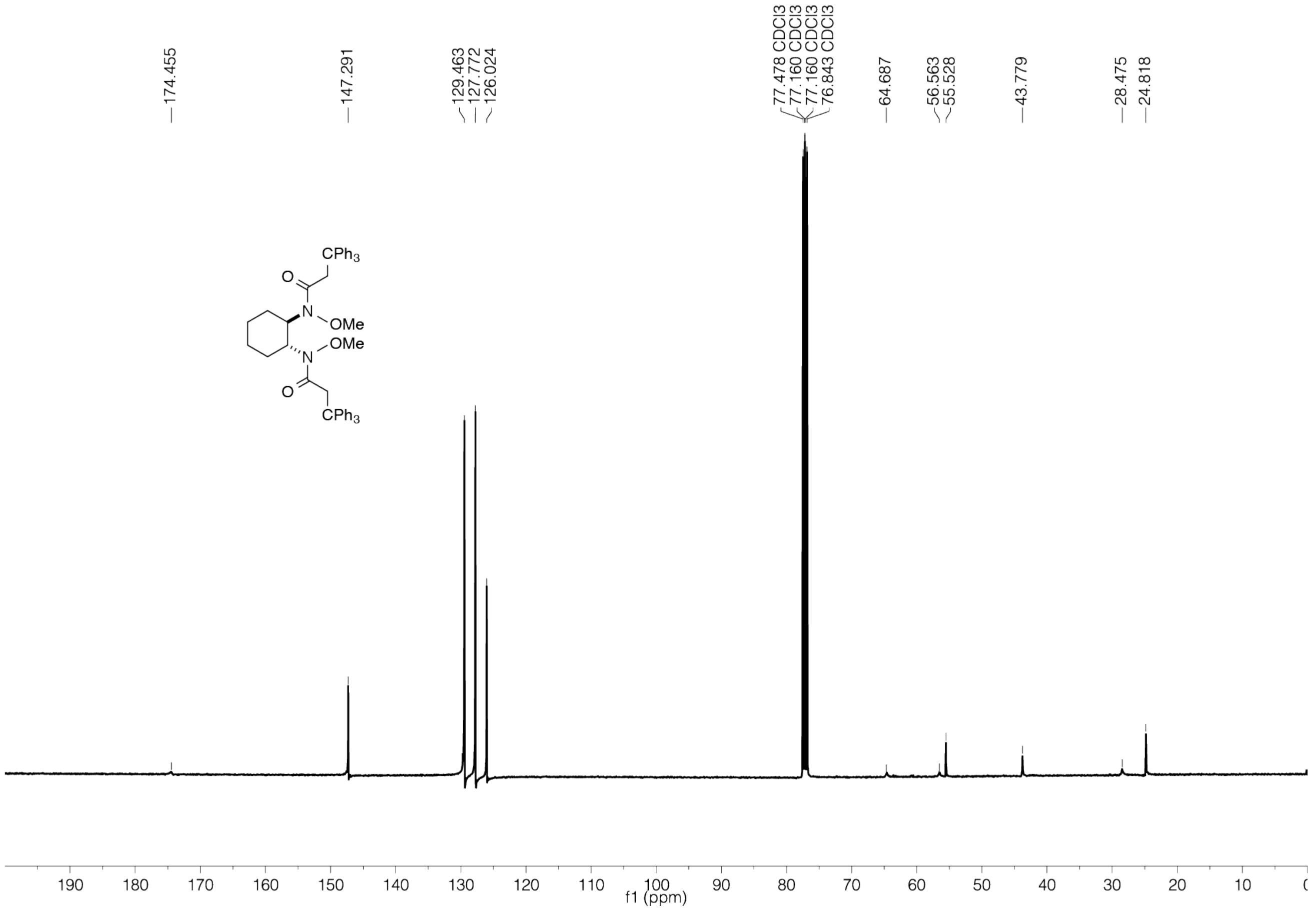

(2*R*,3*S*)-2-Azido-1-(2,3-dihydro-1*H*-pyrrolo[2,3-*b*]pyridin-1-yl)-3-hydroxy-3-(trifluoromethyl)-5-(triisopropylsilyl)pent-4-yn-1-one (5a):

^{1}H NMR (400MHz, $CDCl_3$)

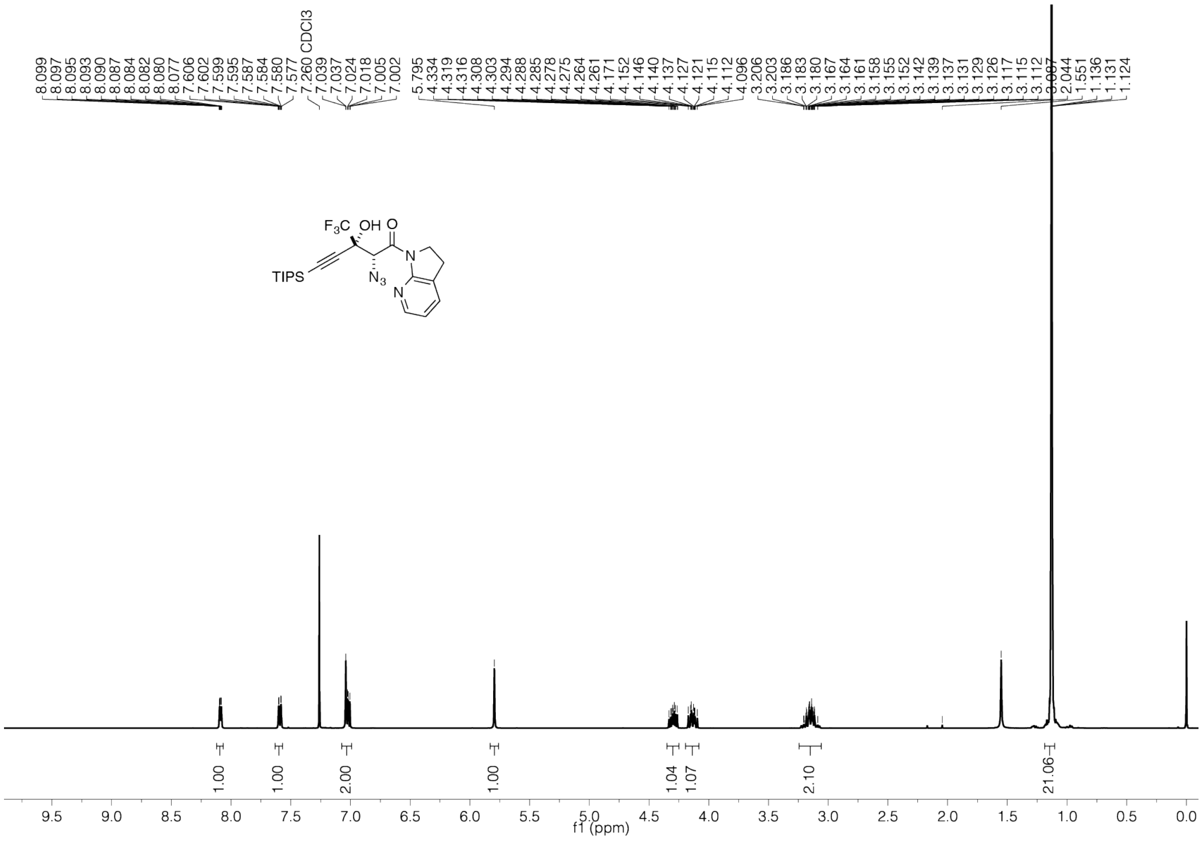

^{13}C NMR (100MHz, $CDCl_3$)

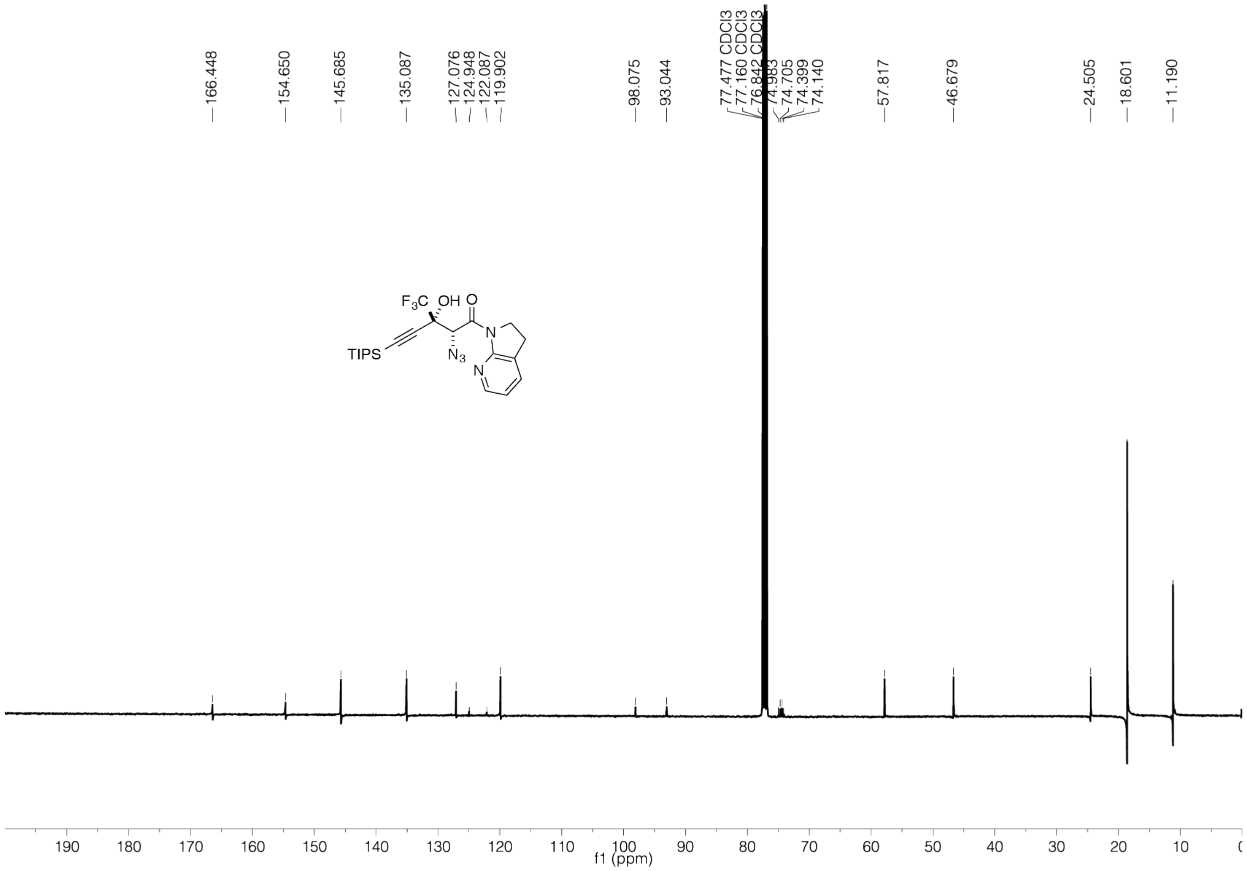

^{19}F NMR (376MHz, $CDCl_3$)

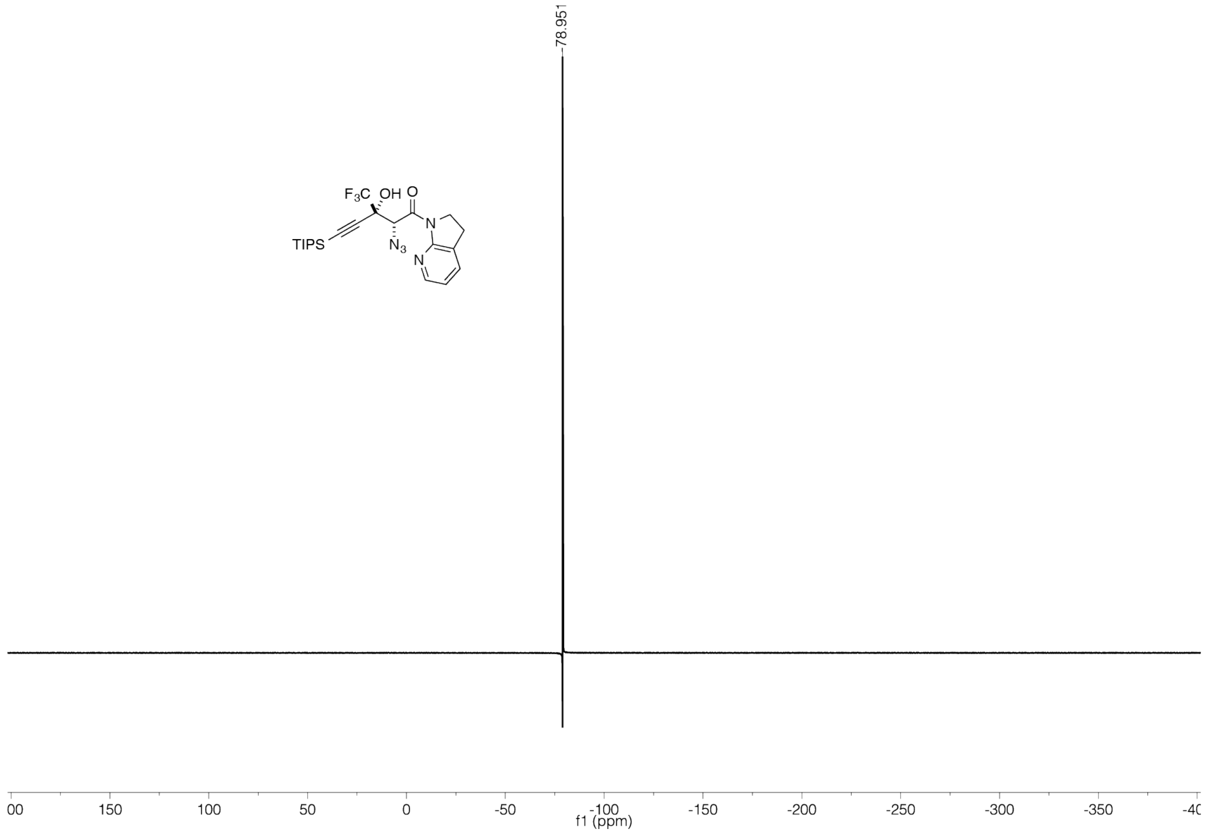

(2*R*,3*S*)-2-Azido-6-((*tert*-butyldimethylsilyl)oxy)-1-(2,3-dihydro-1*H*-pyrrolo[2,3-*b*]pyridin-1-yl)-3-hydroxy-3-(trifluoromethyl)hex-4-yn-1-one (5b):

^{1}H NMR (400MHz, $CDCl_3$)

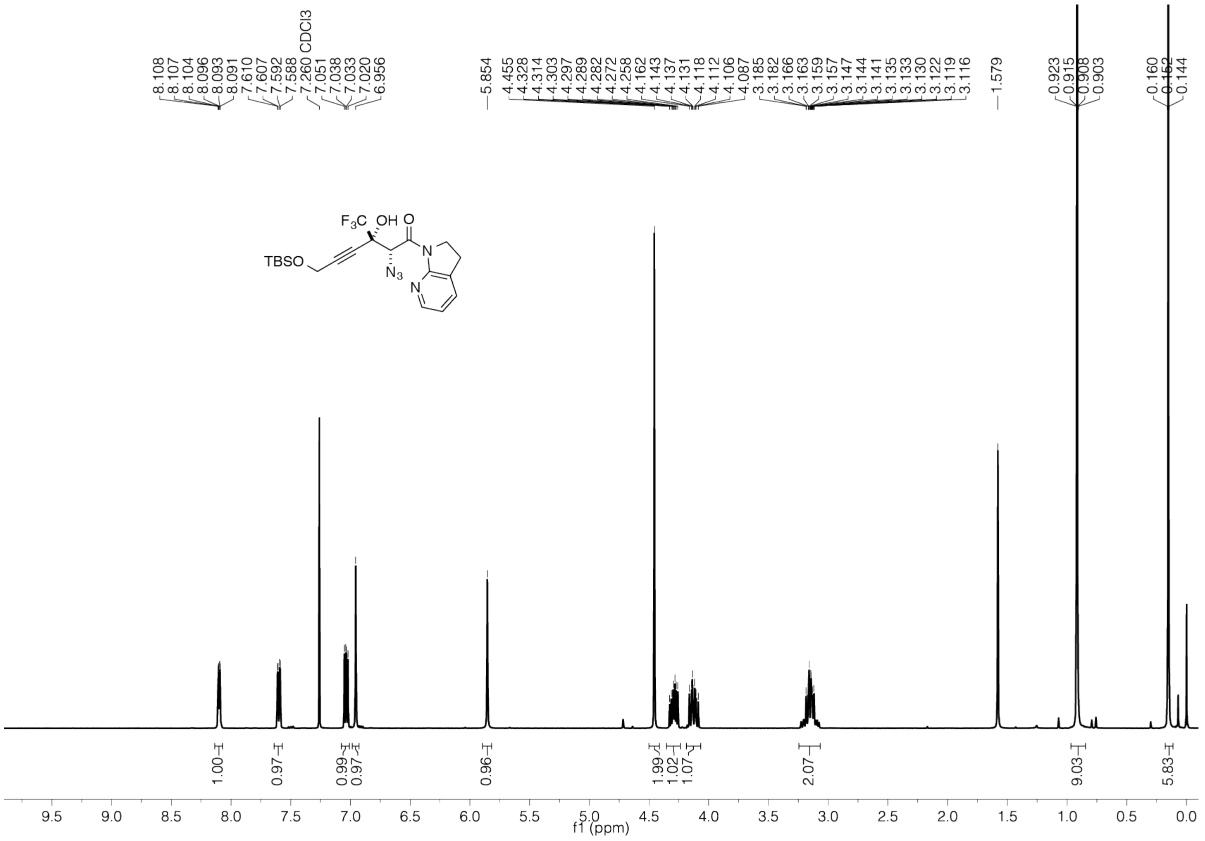

^{13}C NMR (100MHz, $CDCl_3$)

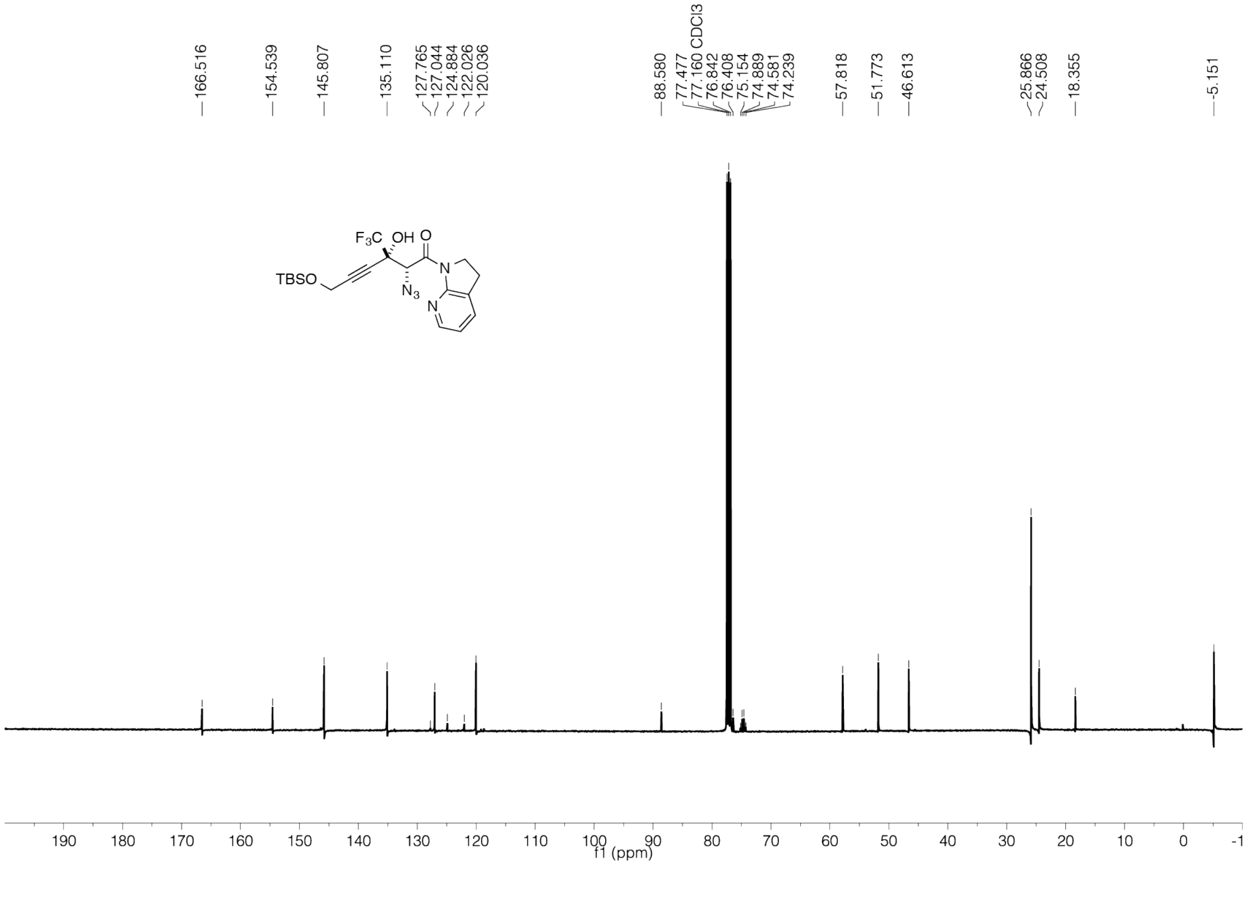

¹⁹F NMR (376MHz, $CDCl_3$)

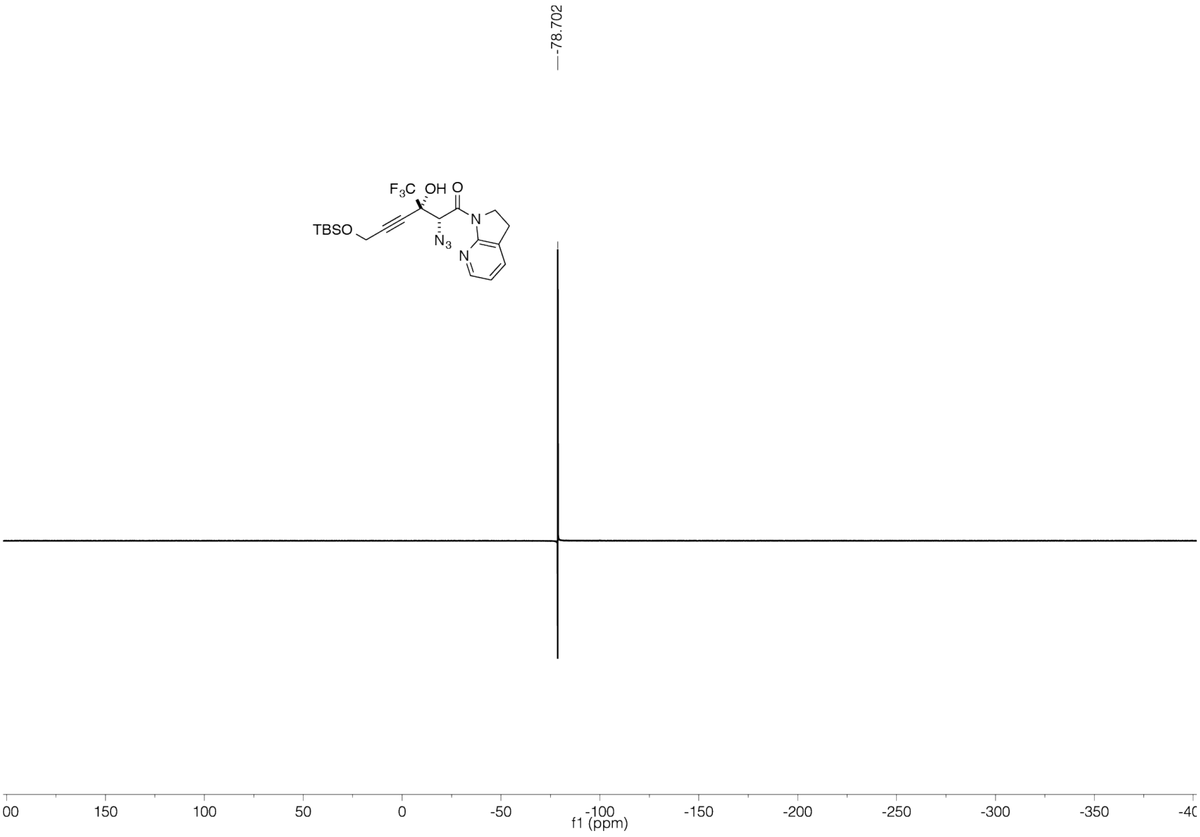

(2*R*,3*S*)-2-Azido-1-(2,3-dihydro-1*H*-pyrrolo[2,3-*b*]pyridin-1-yl)-3-hydroxy-3-(trifluoromethyl)undec-4-yn-1-one (5c):

^{1}H NMR (400MHz, $CDCl_3$)

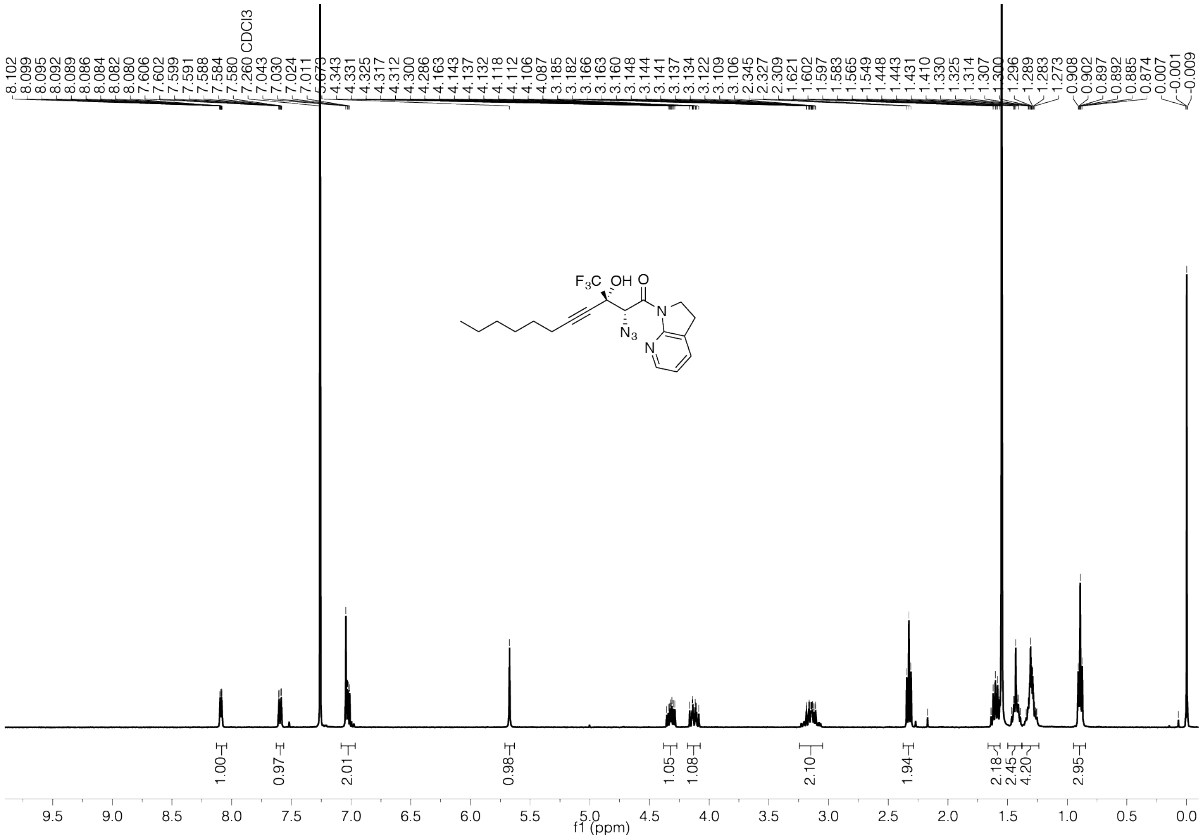

^{13}C NMR (100MHz, $CDCl_3$)

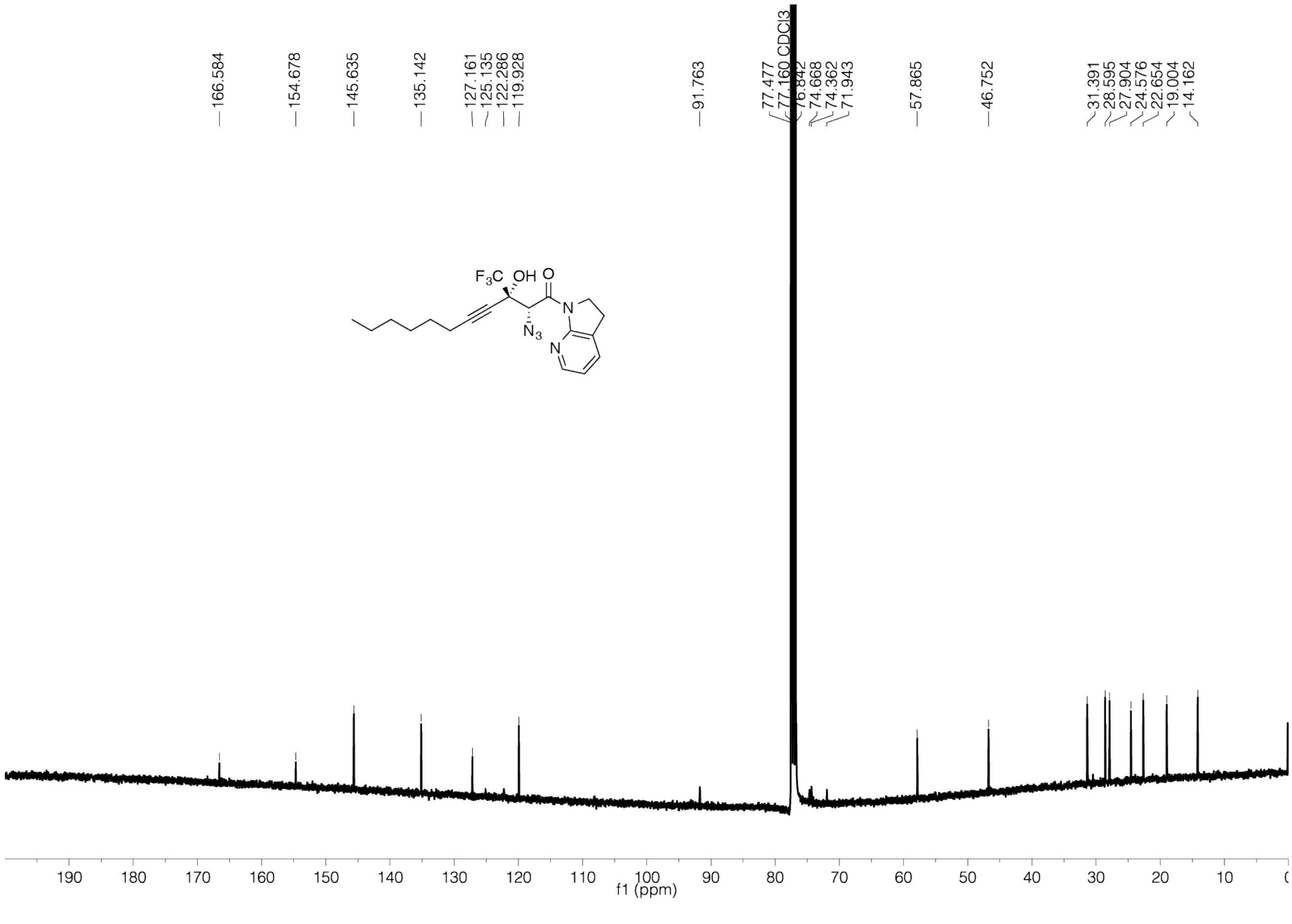

^{19}F NMR (376MHz, $CDCl_3$)

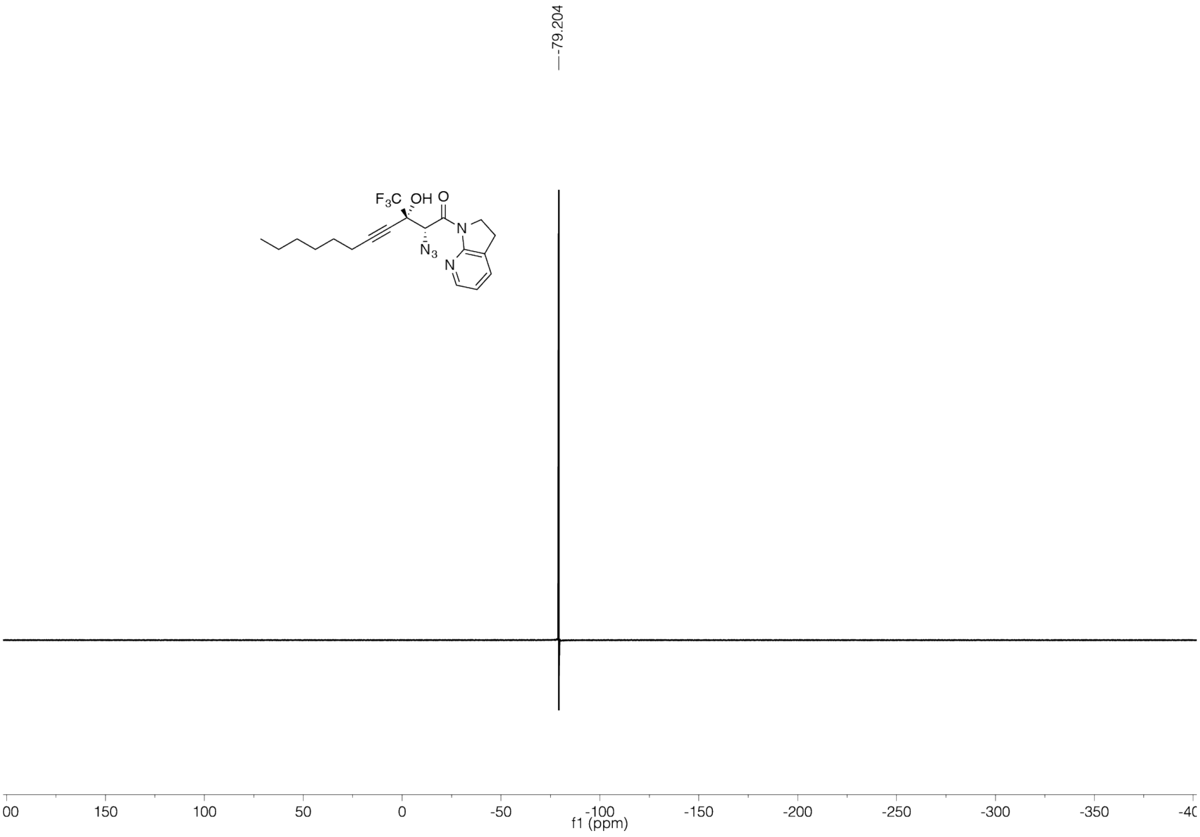

(2*R*,3*S*)-2-Azido-9-((*tert*-butyldiphenylsilyl)oxy)-1-(2,3-dihydro-1*H*-pyrrolo[2,3-*b*]pyridin-1-yl)-3-hydroxy-3-(trifluoromethyl)non-4-yn-1-one (5d):

^{1}H NMR (400MHz, $CDCl_3$)

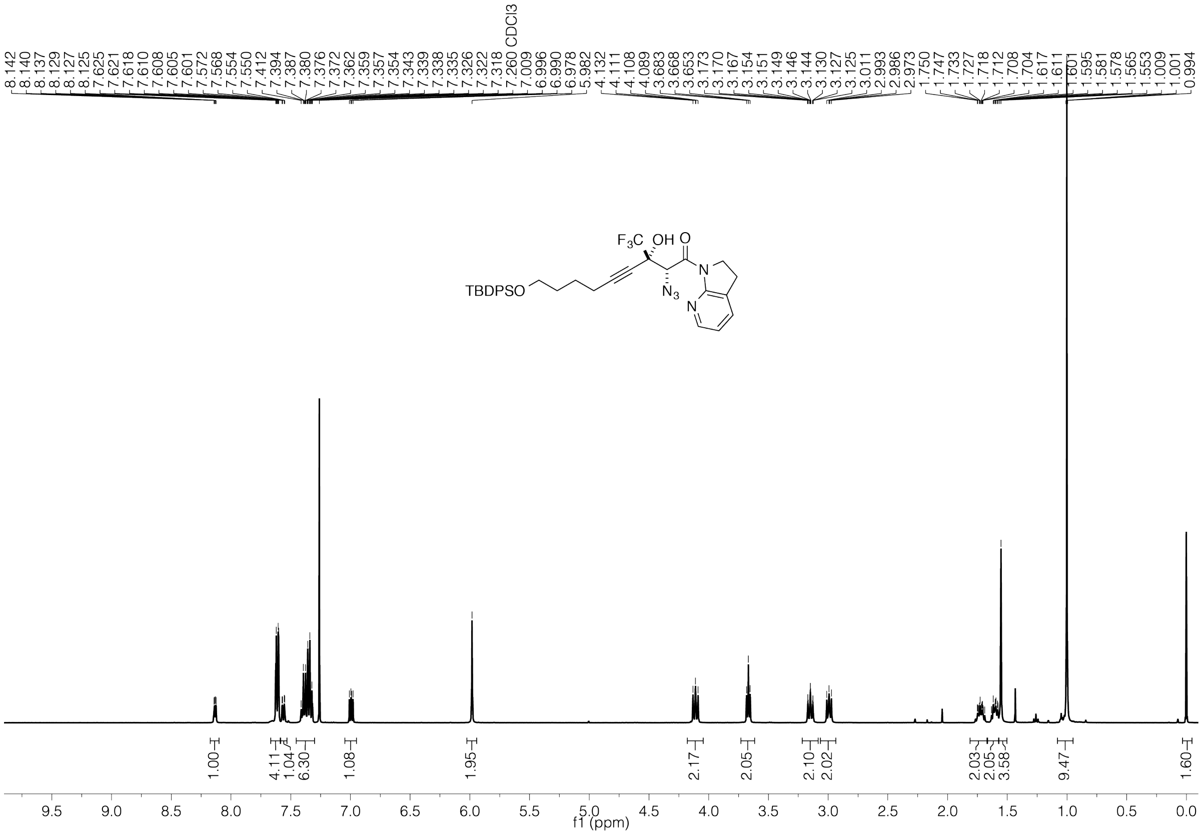

^{13}C NMR (100MHz, $CDCl_3$)

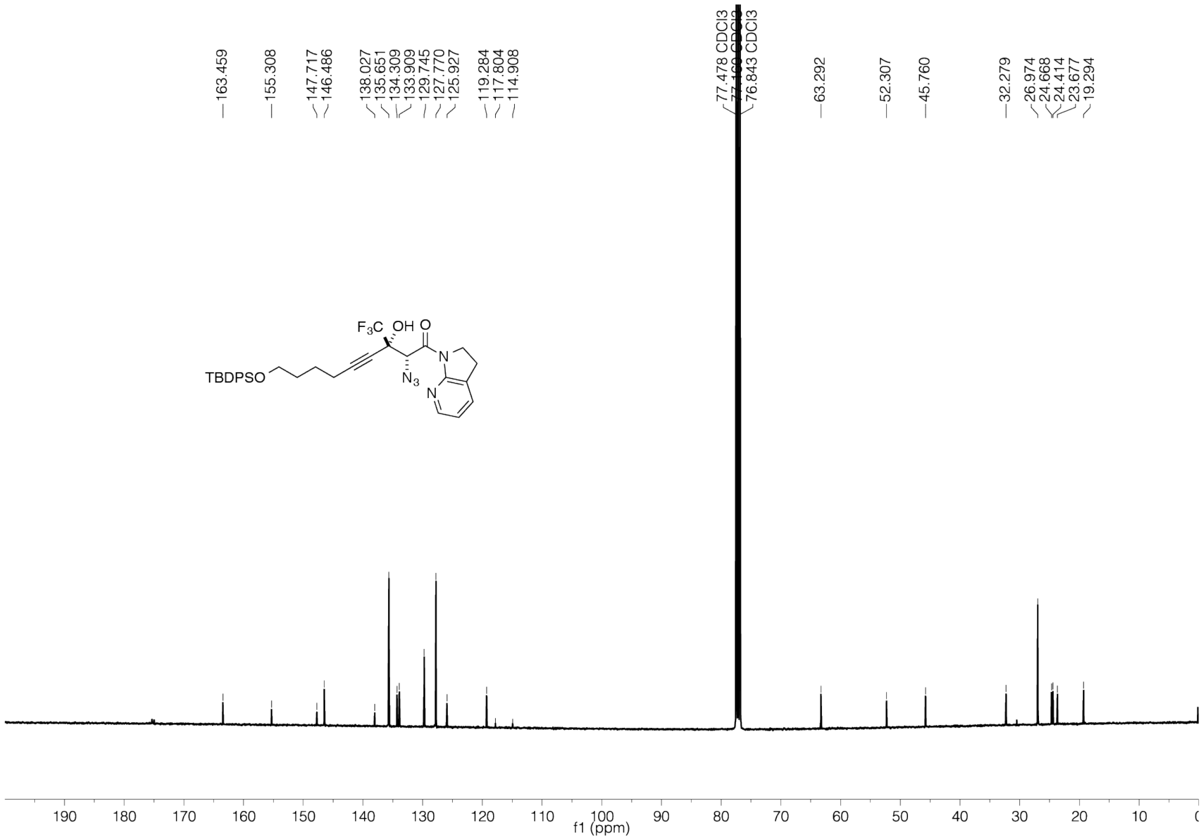

¹⁹F NMR (376MHz, $CDCl_3$)

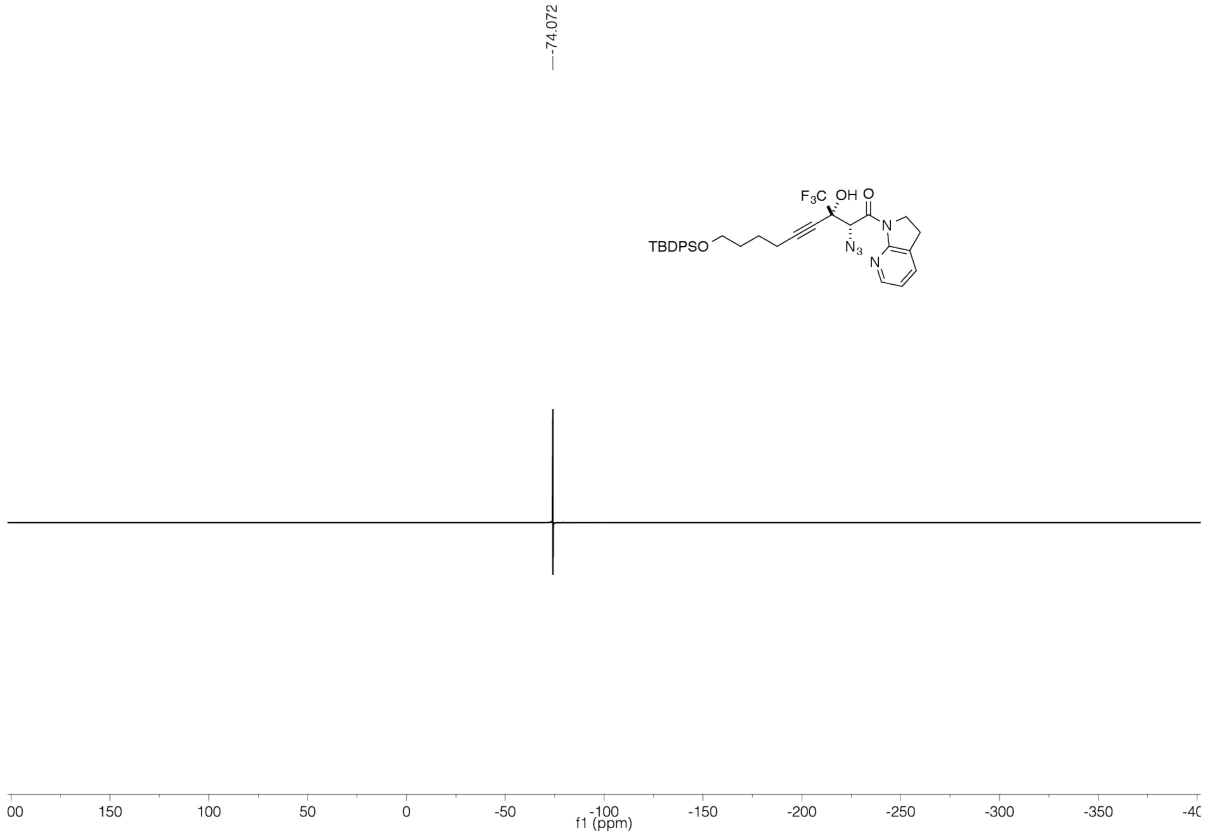

(2*R*,3*S*)-2-Azido-9-chloro-1-(2,3-dihydro-1*H*-pyrrolo[2,3-*b*]pyridin-1-yl)-3-hydroxy-3-(trifluoromethyl)non-4-yn-1-one (5e):

^{1}H NMR (400MHz, CDCl$_3$)

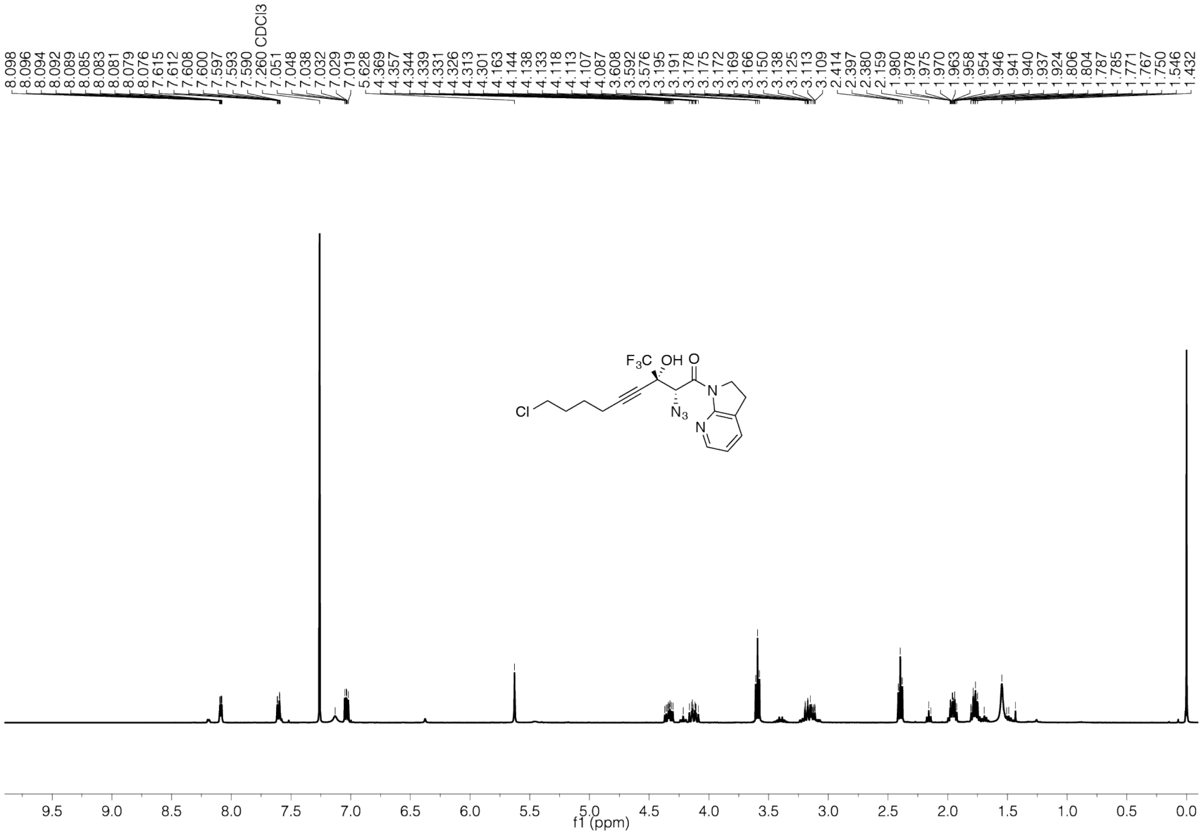

^{13}C NMR (100MHz, CDCl$_3$)

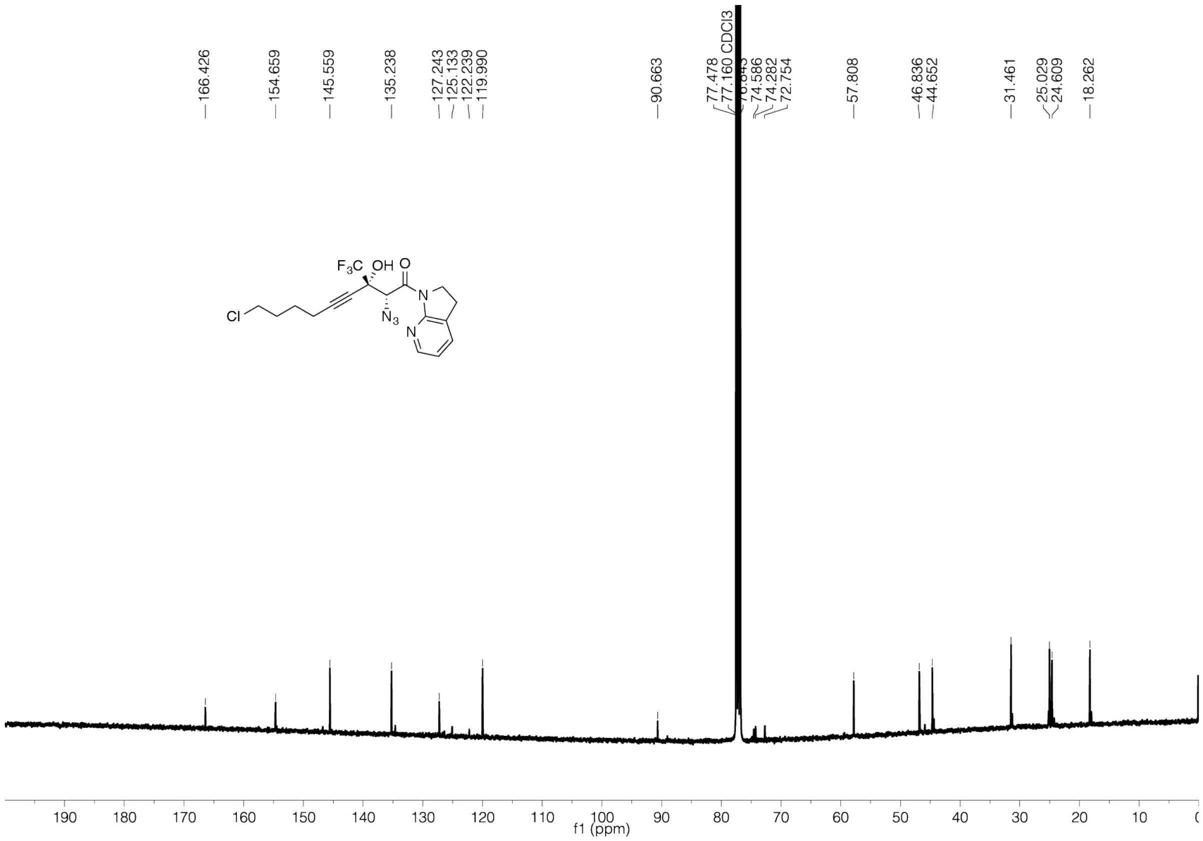

^{19}F NMR (376MHz, $CDCl_3$)

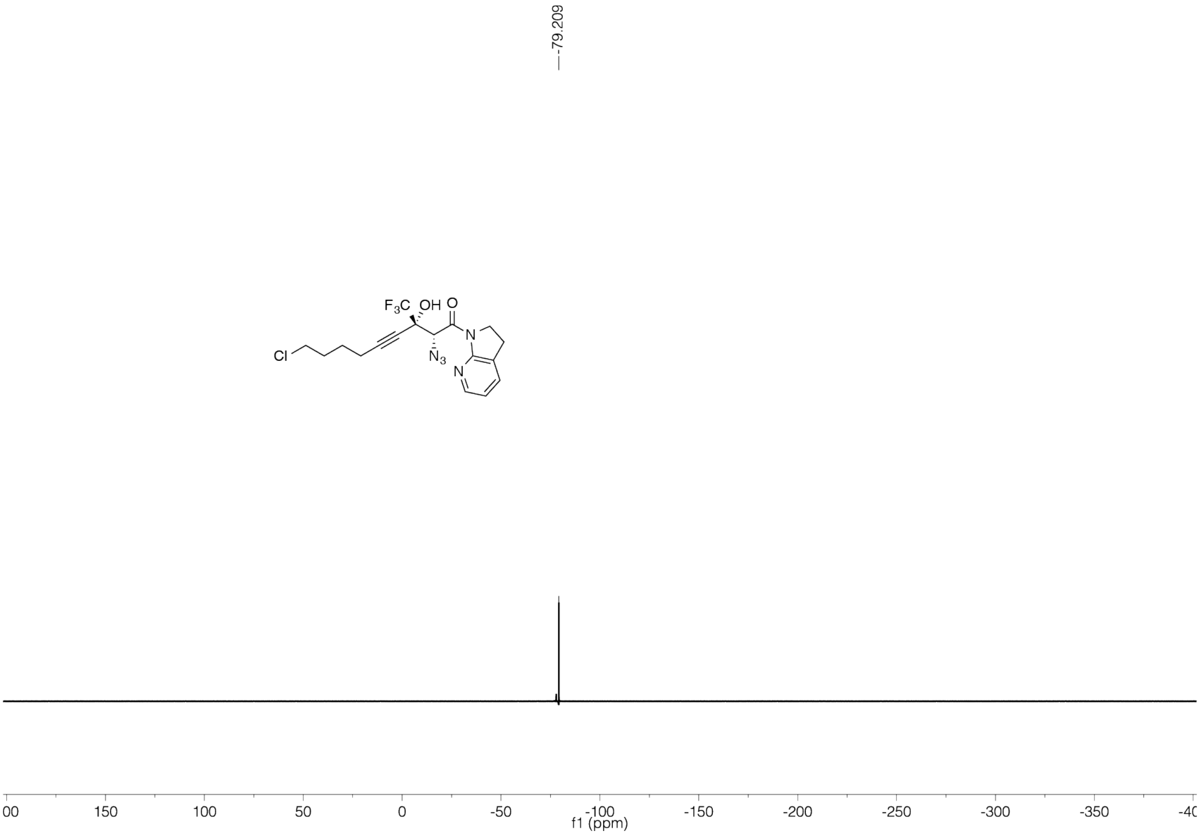

(2*R*,3*S*)-2-Azido-1-(2,3-dihydro-1*H*-pyrrolo[2,3-*b*]pyridin-1-yl)-3-hydroxy-5-(*p*-tolyl)-3-(trifluoromethyl)pent-4-yn-1-one (5f):

^{1}H NMR (400MHz, $CDCl_3$)

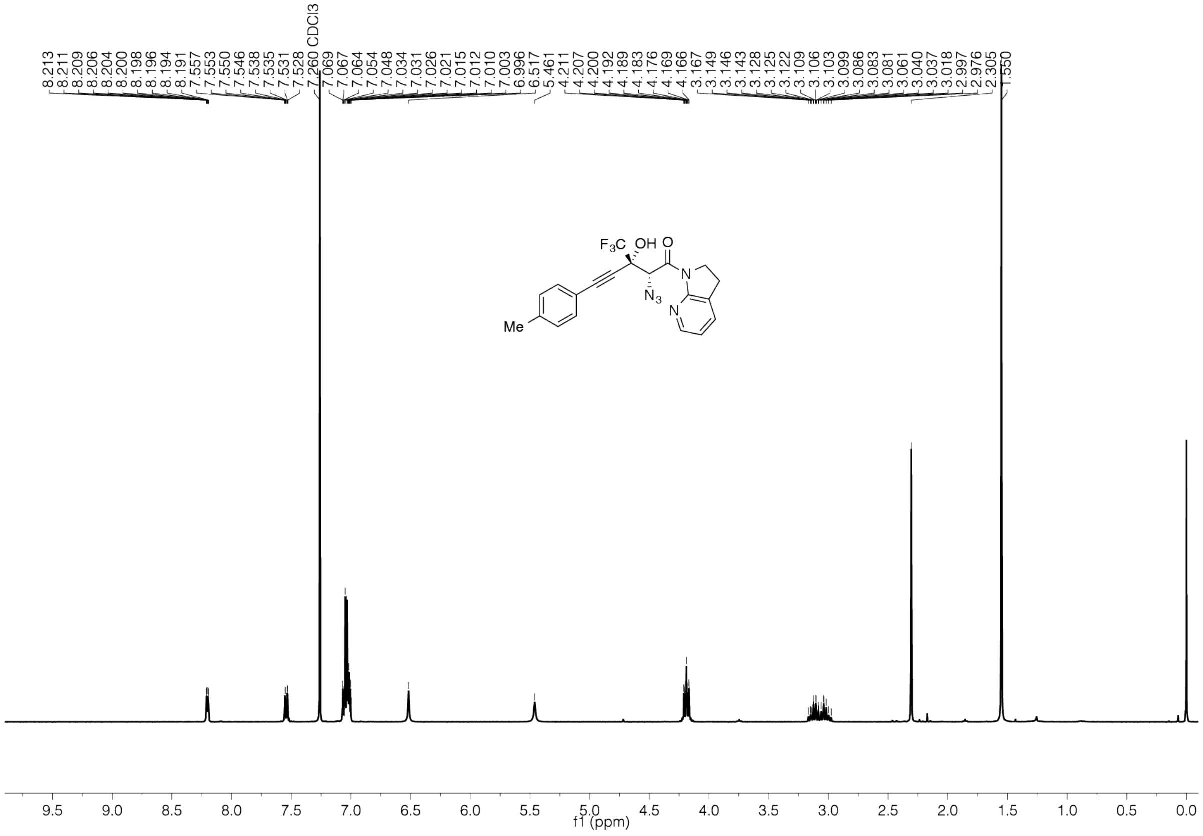

^{13}C NMR (100MHz, $CDCl_3$)

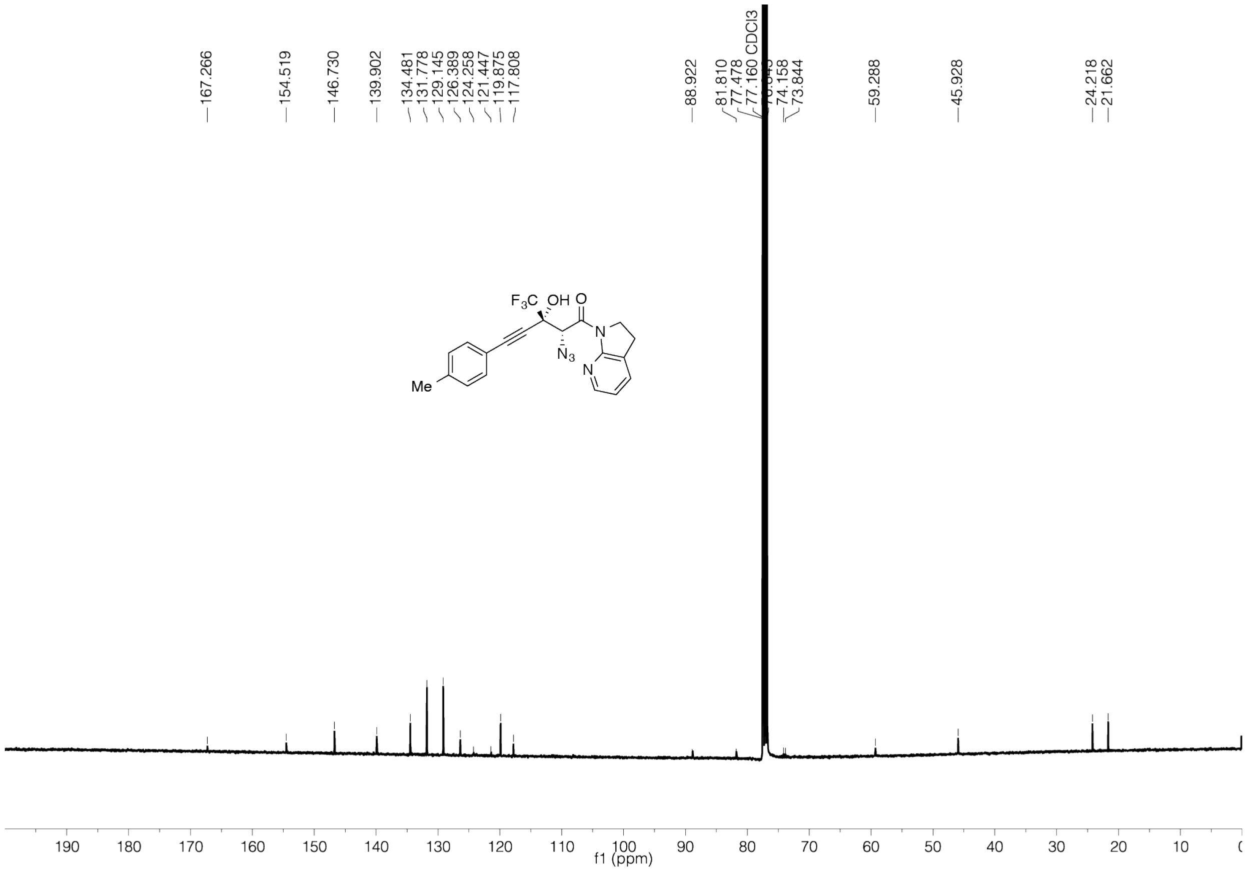

^{19}F NMR (376MHz, $CDCl_3$)

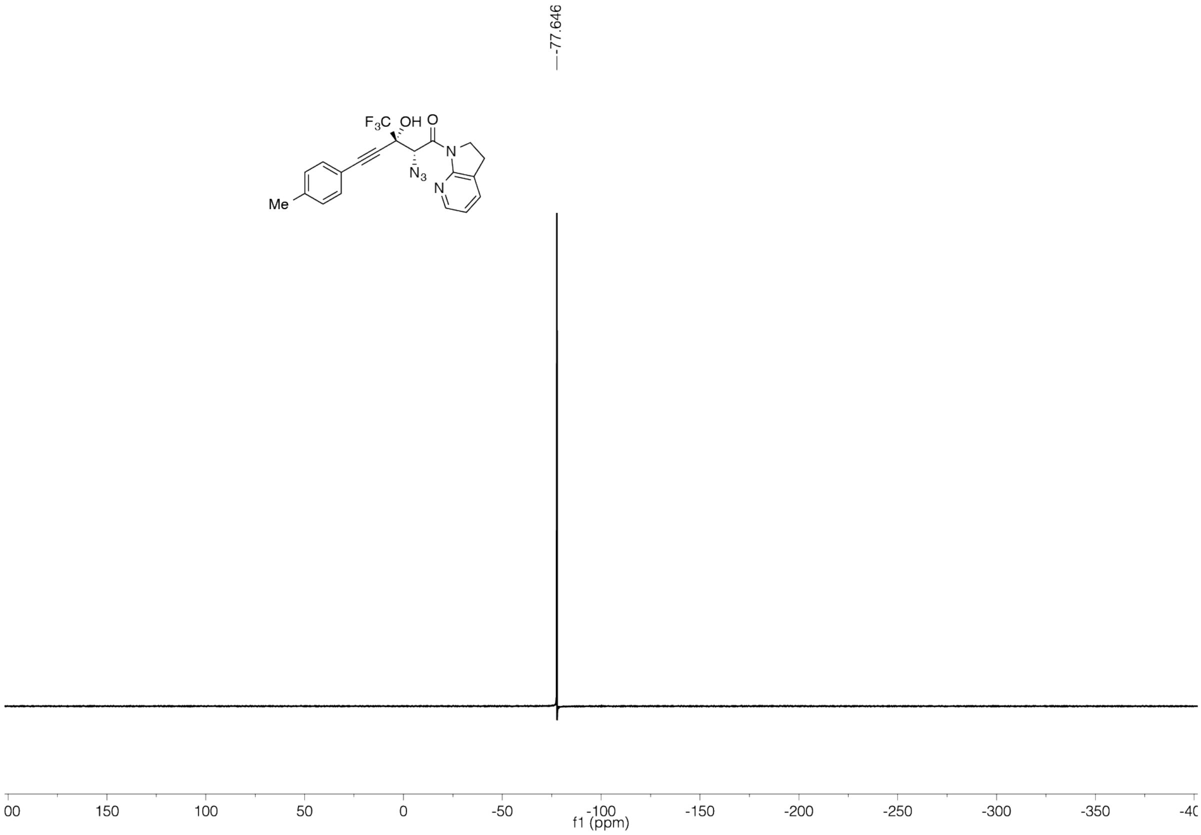

(2*R*,3*S*)-2-Azido-5-(4-chlorophenyl)-1-(2,3-dihydro-1*H*-pyrrolo[2,3-*b*]pyridin-1-yl)-3-hydroxy-3-(trifluoromethyl)pent-4-yn-1-one (5g):

^{1}H NMR (400MHz, CDCl$_3$)

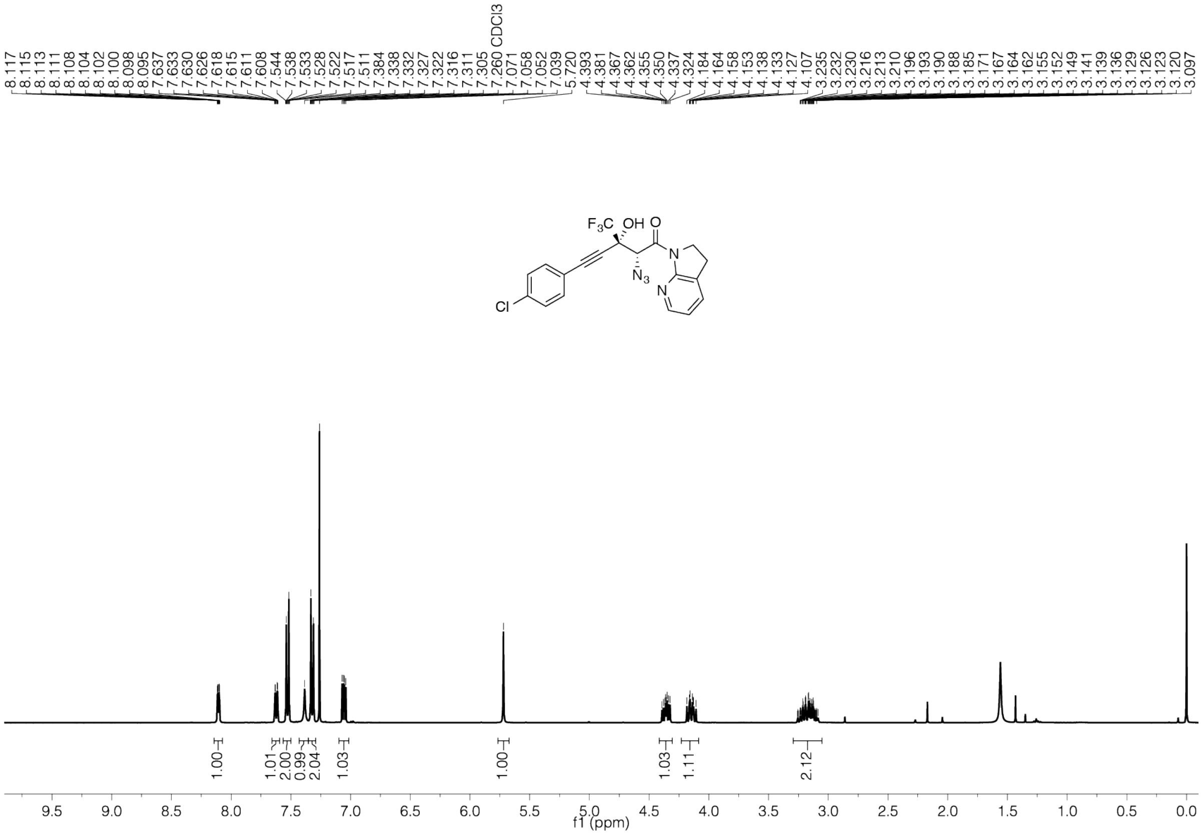

^{13}C NMR (100MHz, CDCl$_3$)

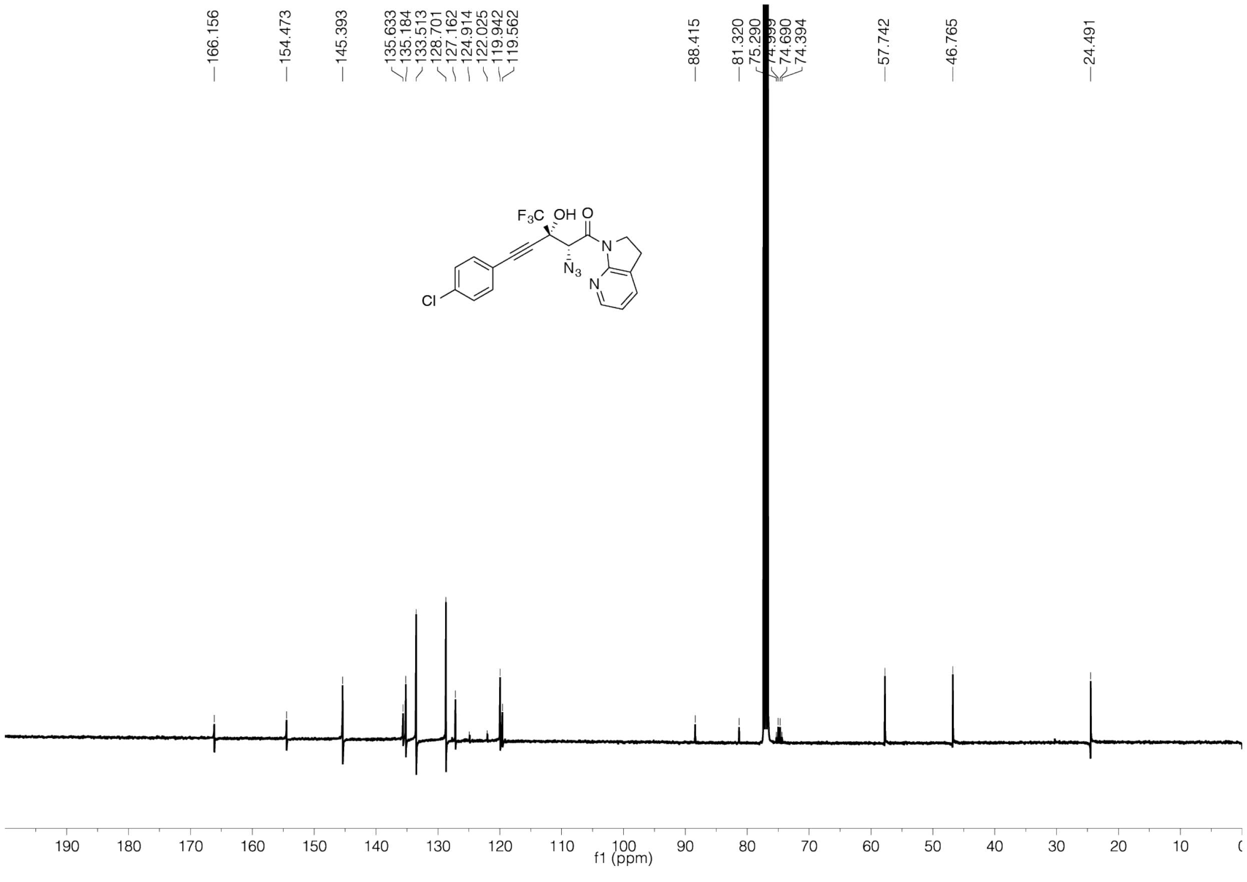

^{19}F NMR (376MHz, $CDCl_3$)

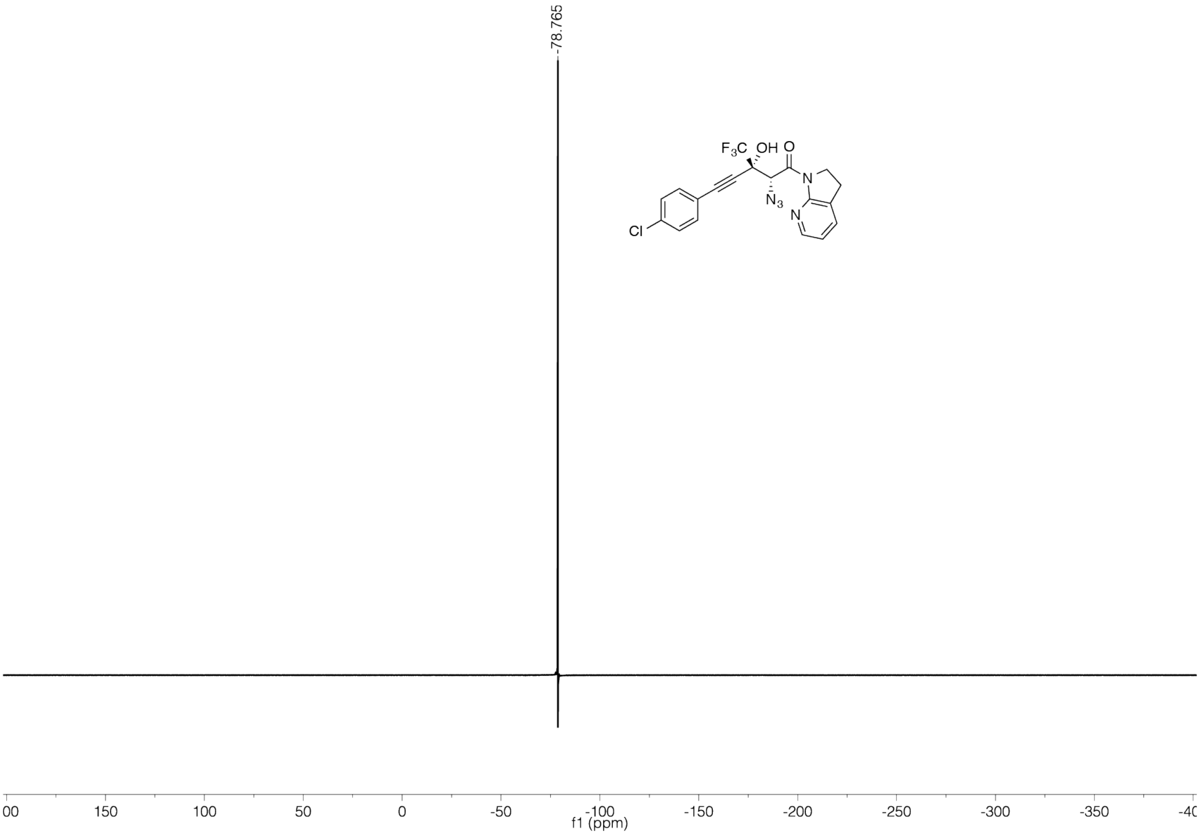

(2*R*,3*S*)-5-(4-(1,3-Dioxolan-2-yl)phenyl)-2-azido-1-(2,3-dihydro-1*H*-pyrrolo[2,3-*b*]pyridin-1-yl)-3-hydroxy-3-(trifluoro methyl)pent-4-yn-1-one (5h):

^{1}H NMR (400MHz, $CDCl_3$)

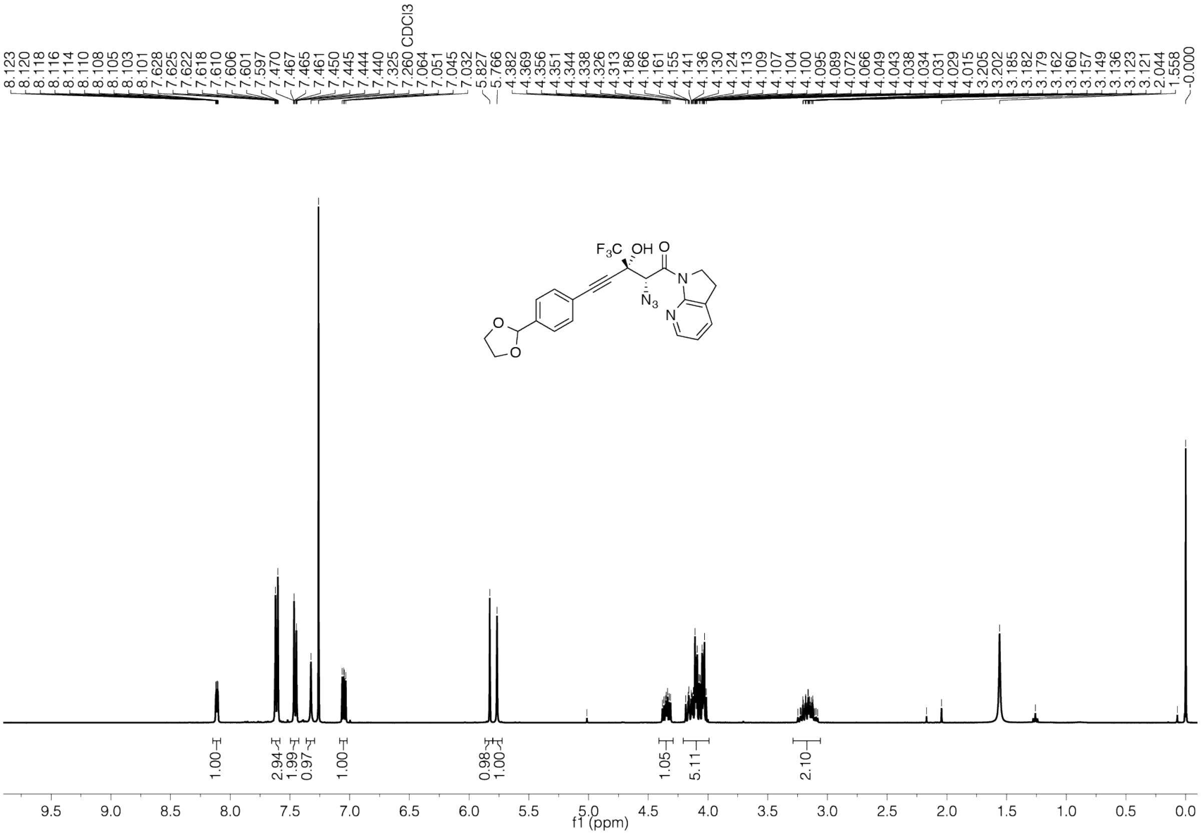

^{13}C NMR (100MHz, $CDCl_3$)

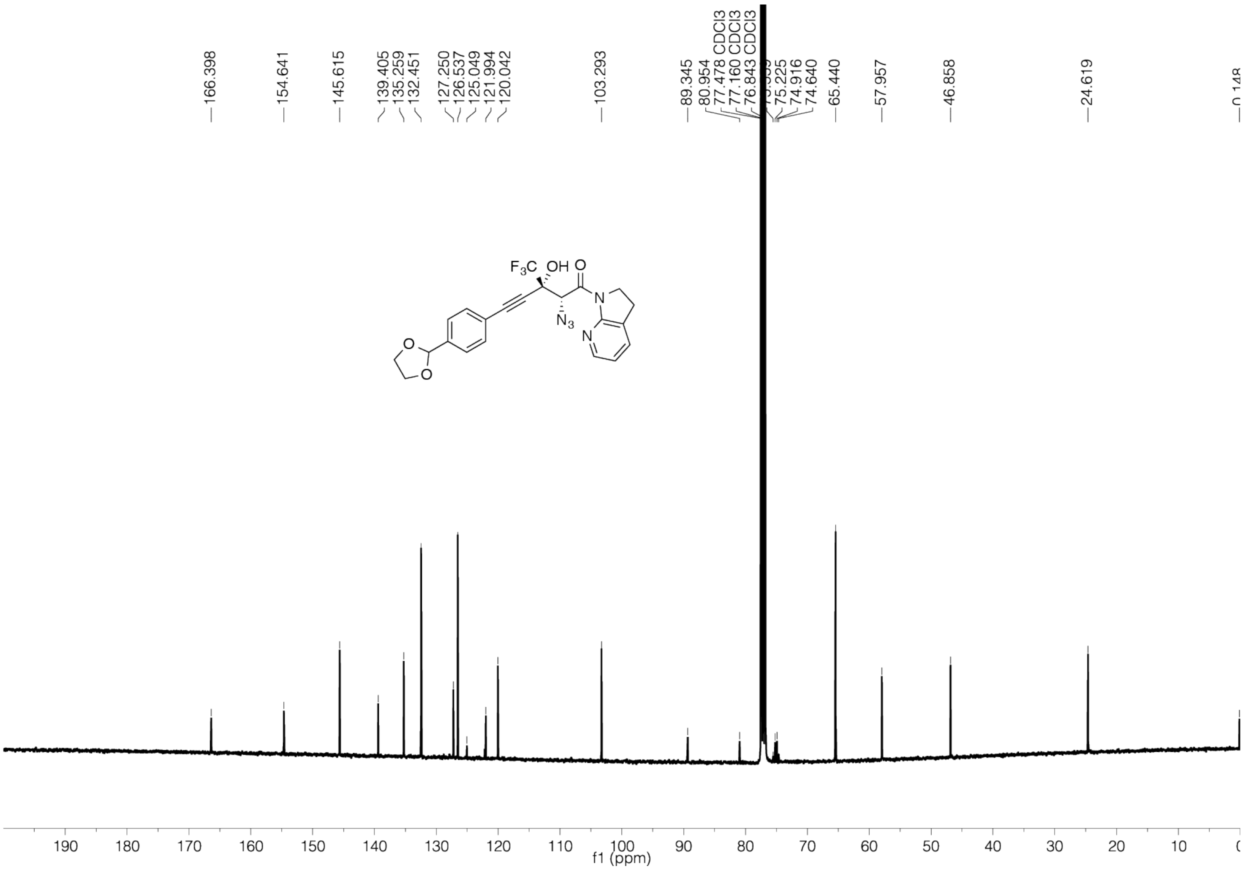

^{19}F NMR (376MHz, $CDCl_3$)

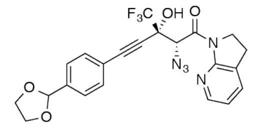

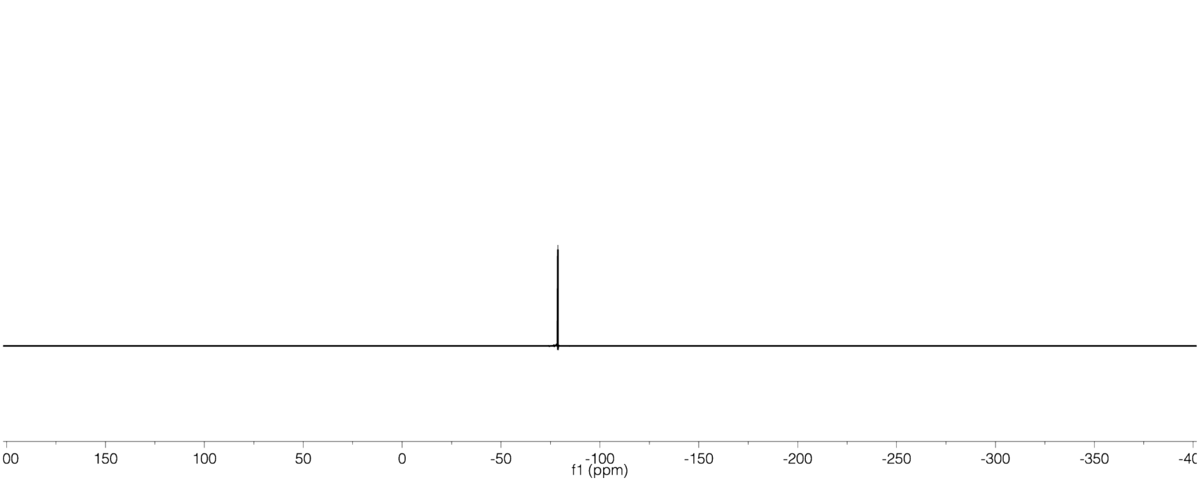

(2*R*,3*S*)-2-aAido-1-(2,3-dihydro-1*H*-pyrrolo[2,3-*b*]pyridin-1-yl)-3-hydroxy-5-(4-(morpholine-4-carbonyl)phenyl)-3-(trifluoromethyl)pent-4-yn-1-one (5i):

^{1}H NMR (400MHz, $CDCl_3$)

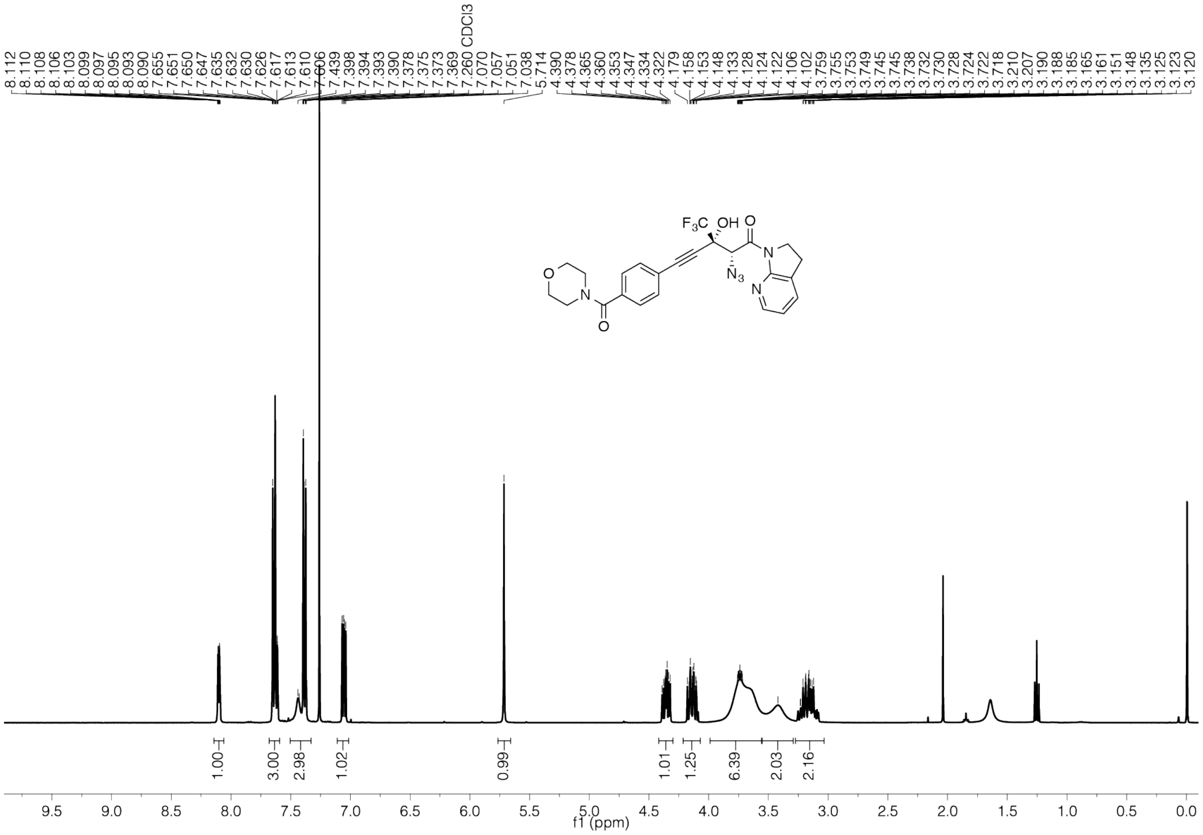

^{13}C NMR (100MHz, $CDCl_3$)

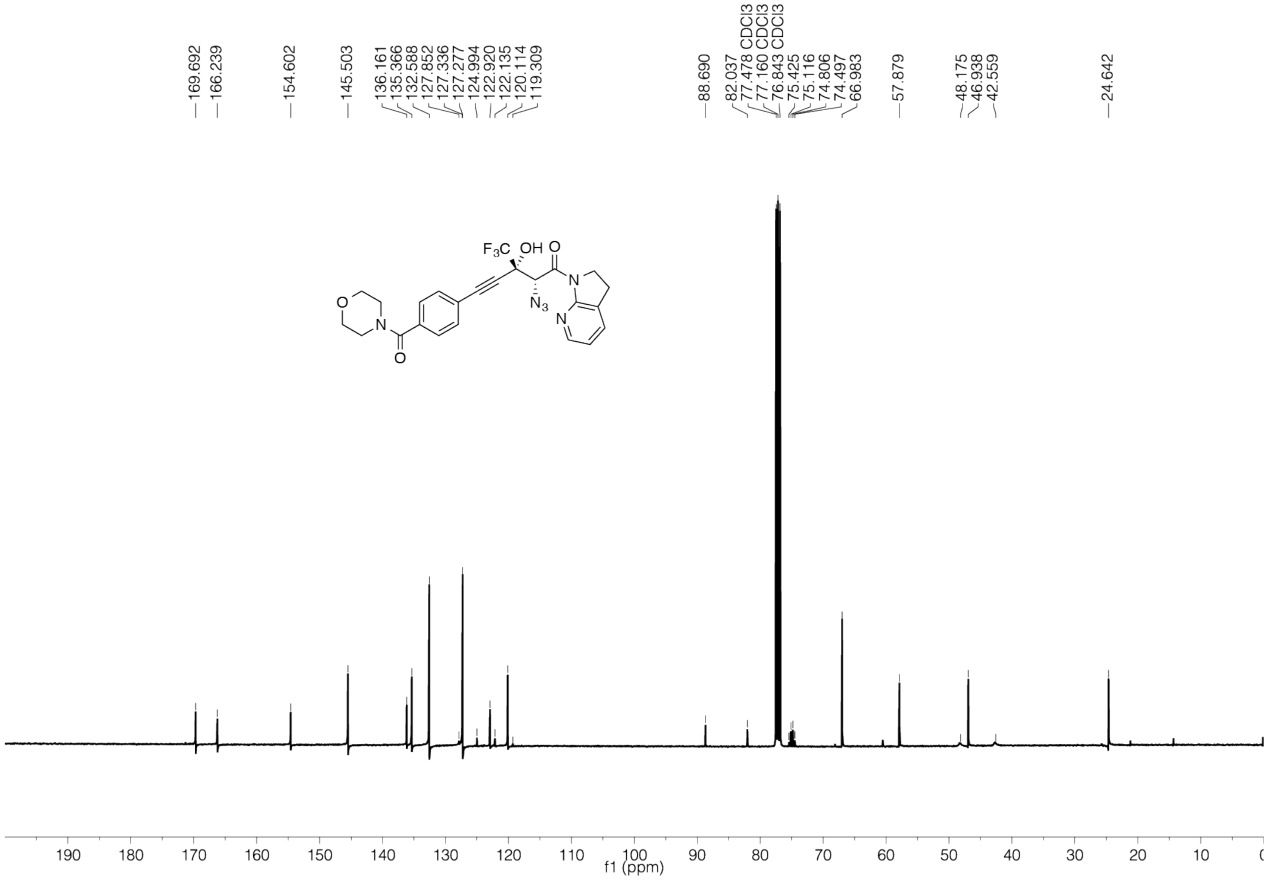

^{19}F NMR (376MHz, $CDCl_3$)

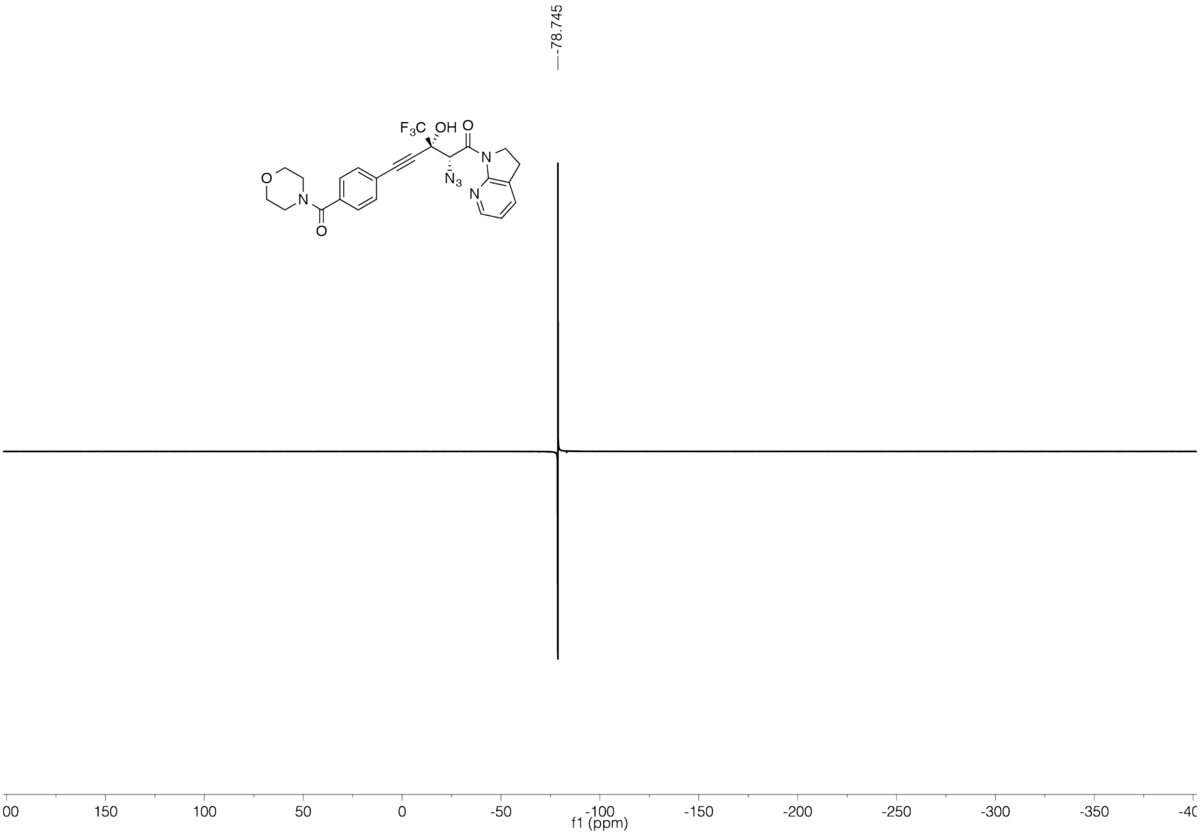

(2*R*,3*S*)-2-Azido-1-(2,3-dihydro-1*H*-pyrrolo[2,3-*b*]pyridin-1-yl)-3-hydroxy-5-(thiophen-3-yl)-3-(trifluoromethyl)pent-4-yn-1-one (5j):

^{1}H NMR (400MHz, $CDCl_3$)

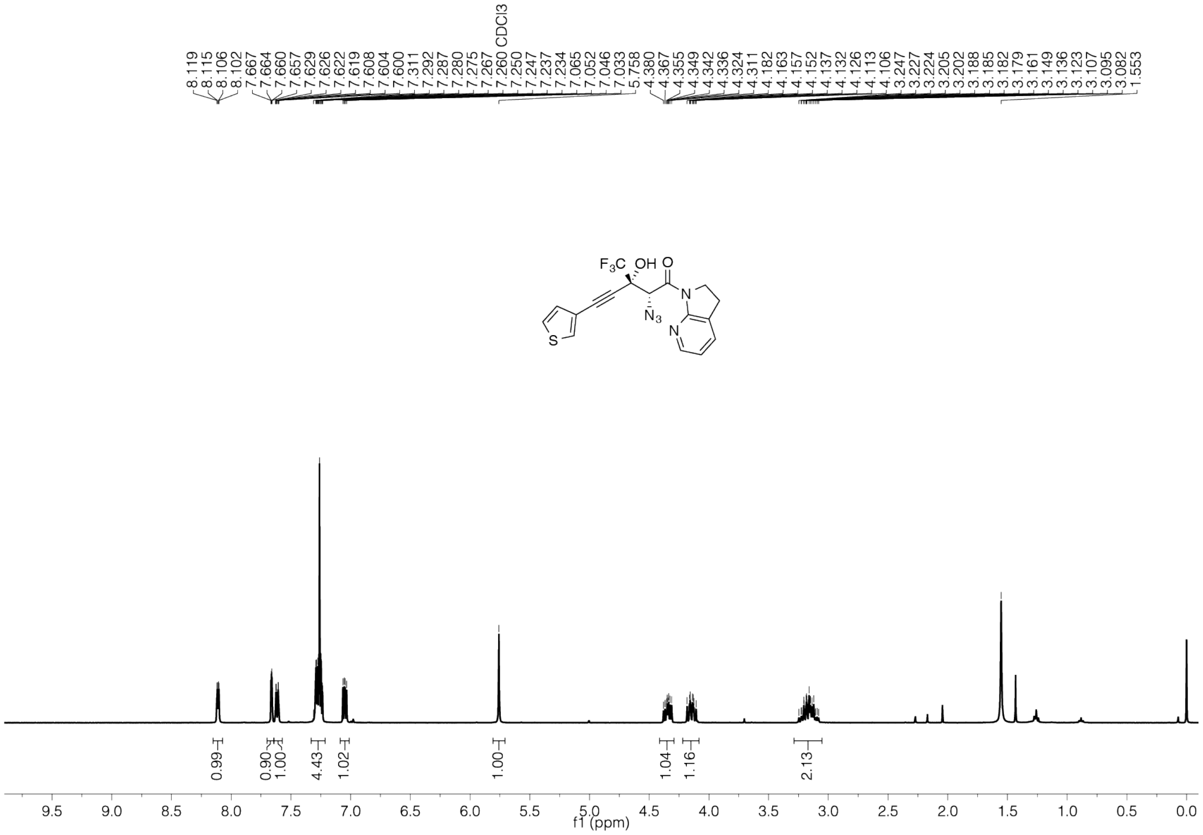

^{13}C NMR (100MHz, $CDCl_3$)

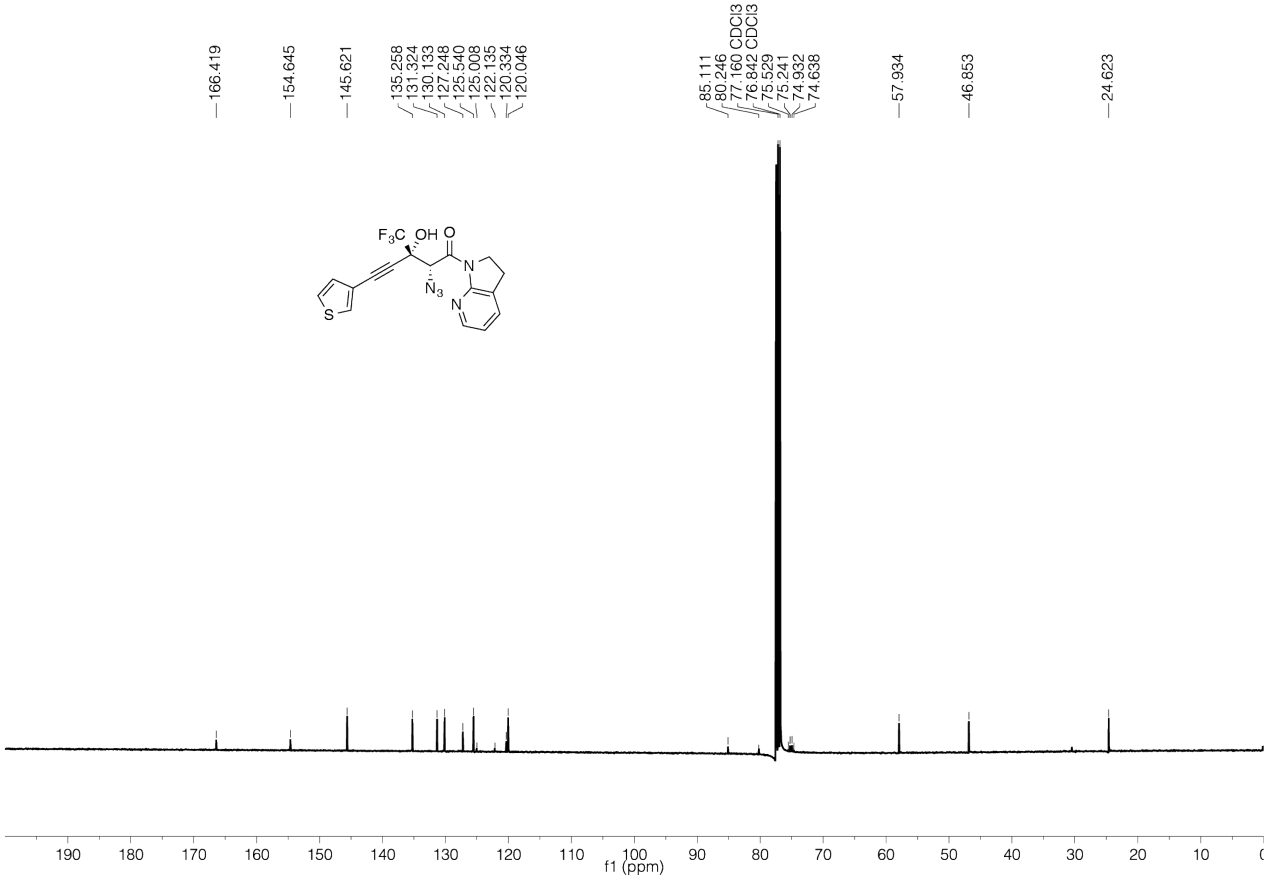

^{19}F NMR (376MHz, $CDCl_3$)

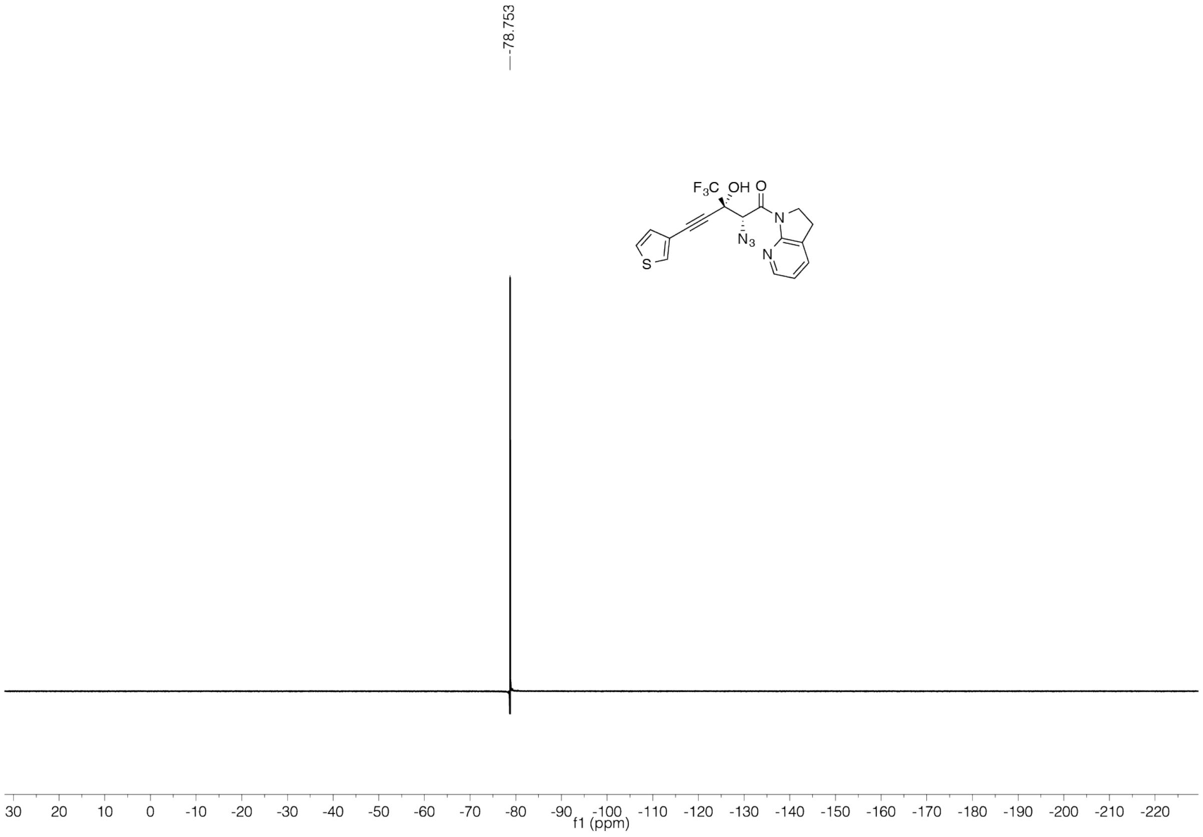

(2*R*,3*S*)-2-Azido-5-cyclohexyl-1-(2,3-dihydro-1*H*-pyrrolo[2,3-*b*]pyridin-1-yl)-3-hydroxy-3-(trifluoromethyl)pent-4-yn-1-one (5k):

^{1}H NMR (400MHz, $CDCl_3$)

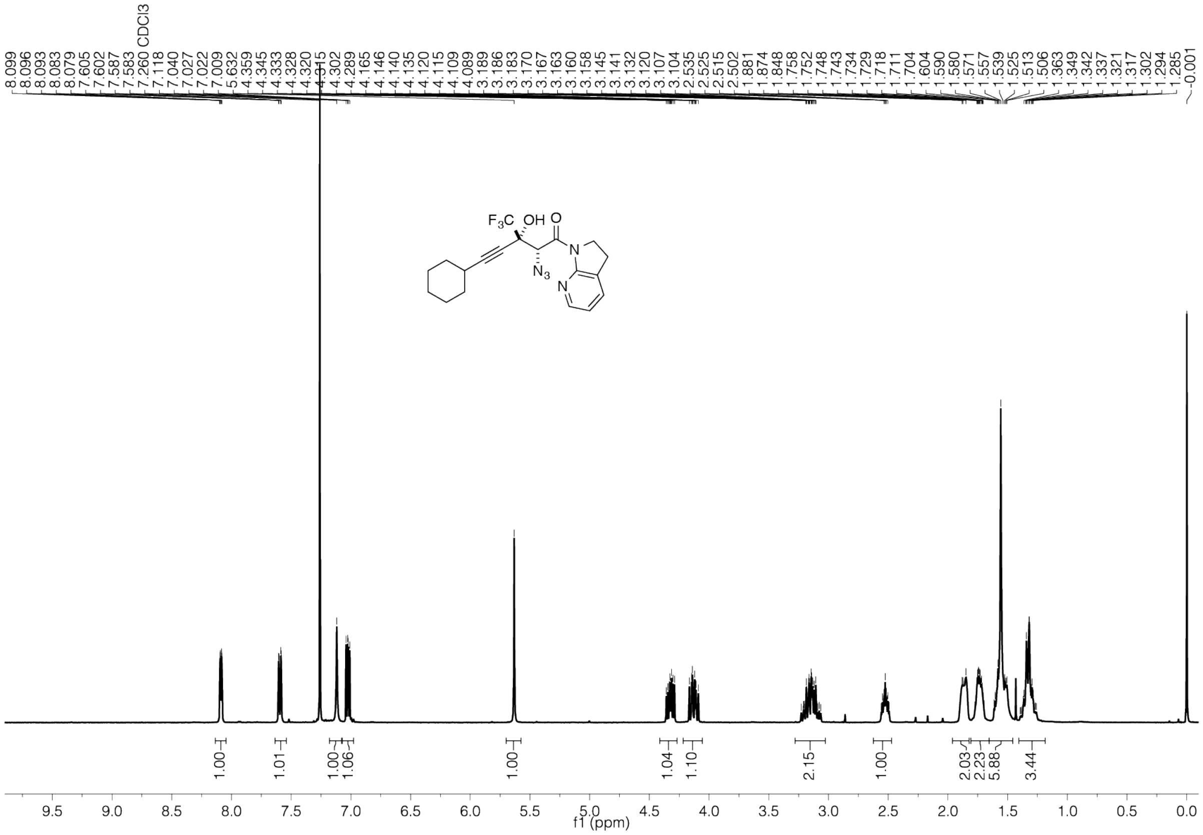

^{13}C NMR (100MHz, $CDCl_3$)

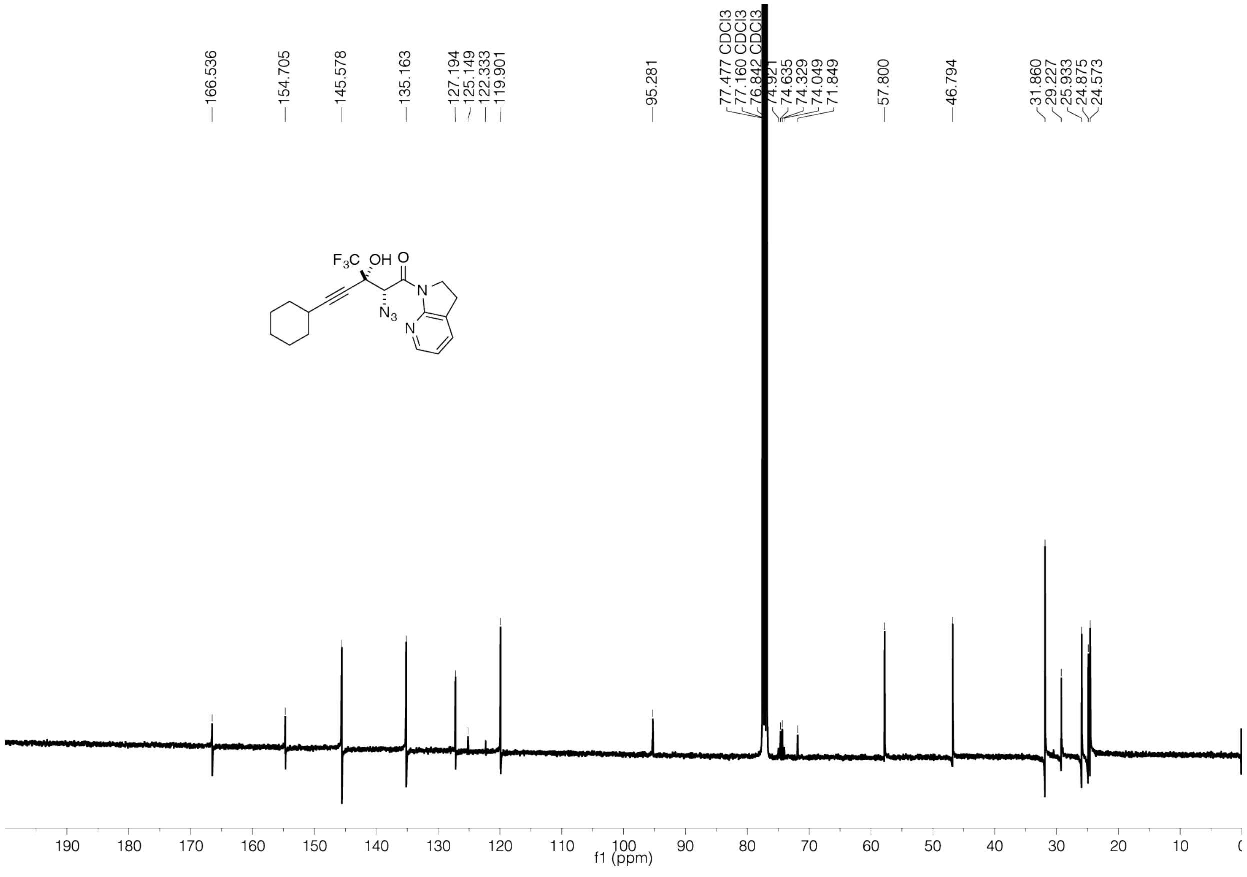

^{19}F NMR (376MHz, $CDCl_3$)

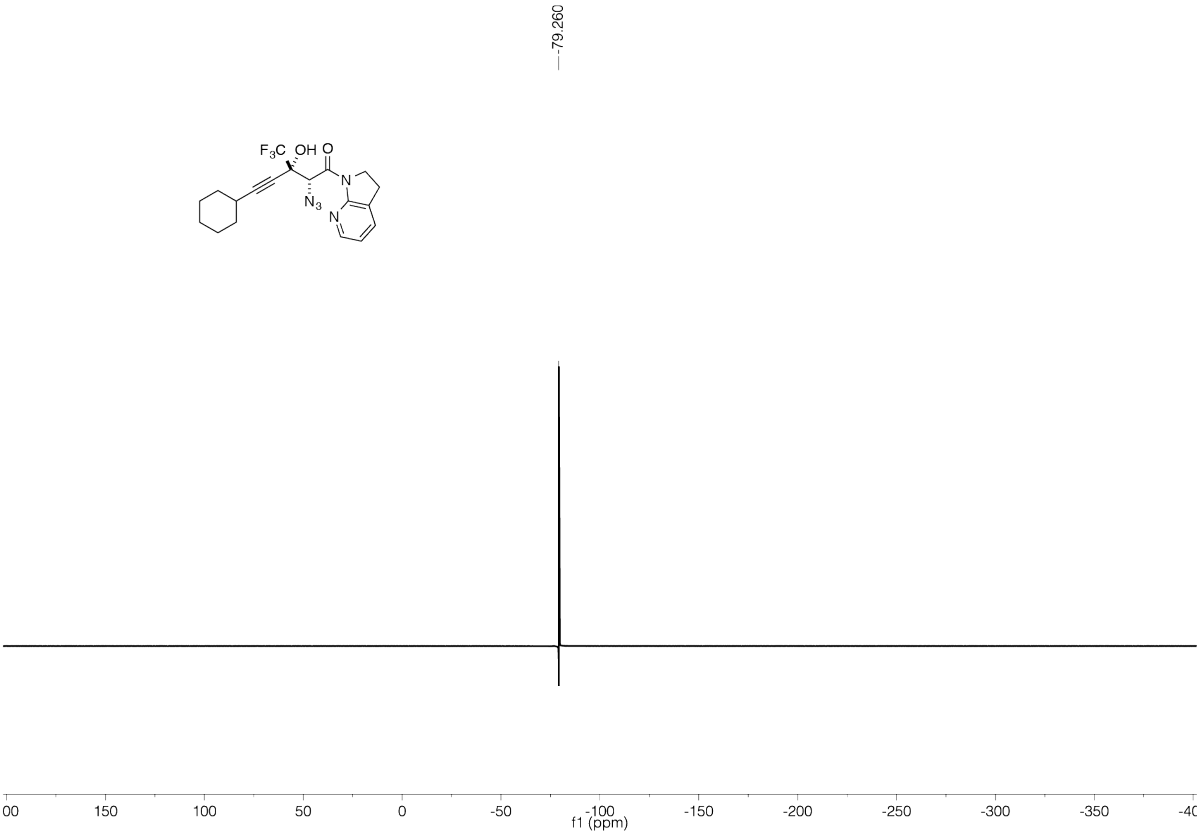

(2*R*,3*S*)-2-Azido-3-(difluoromethyl)-1-(2,3-dihydro-1*H*-pyrrolo[2,3-*b*]pyridin-1-yl)-3-hydroxyundec-4-yn-1-one (12a):

^{1}H NMR (400MHz, $CDCl_3$)

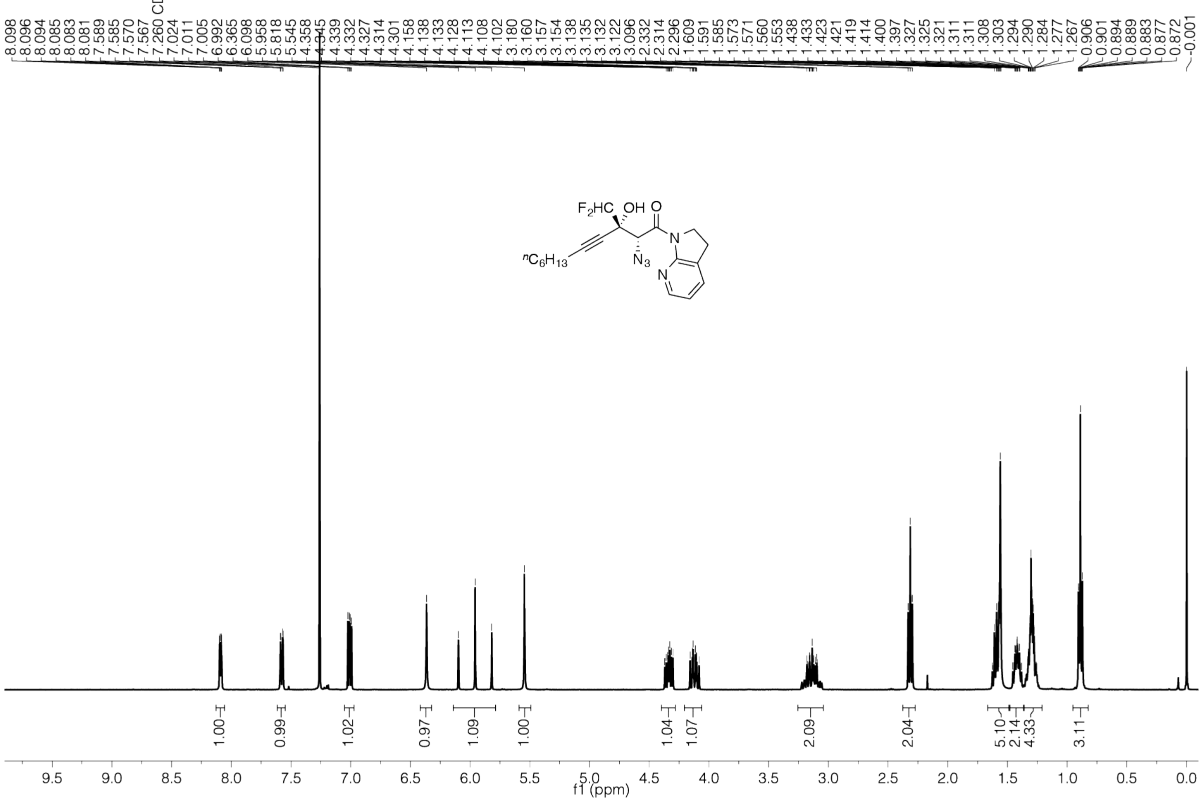

^{13}C NMR (100MHz, $CDCl_3$)

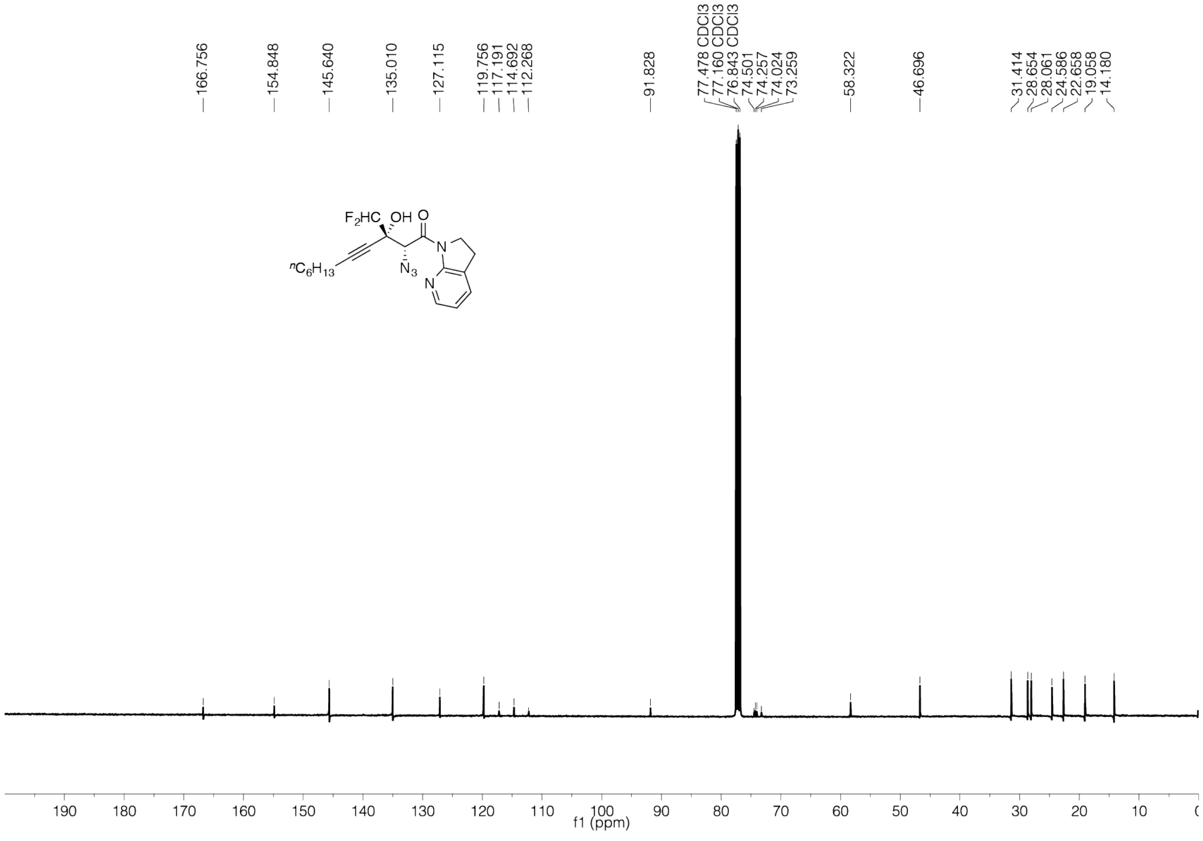

^{19}F NMR (376MHz, $CDCl_3$)

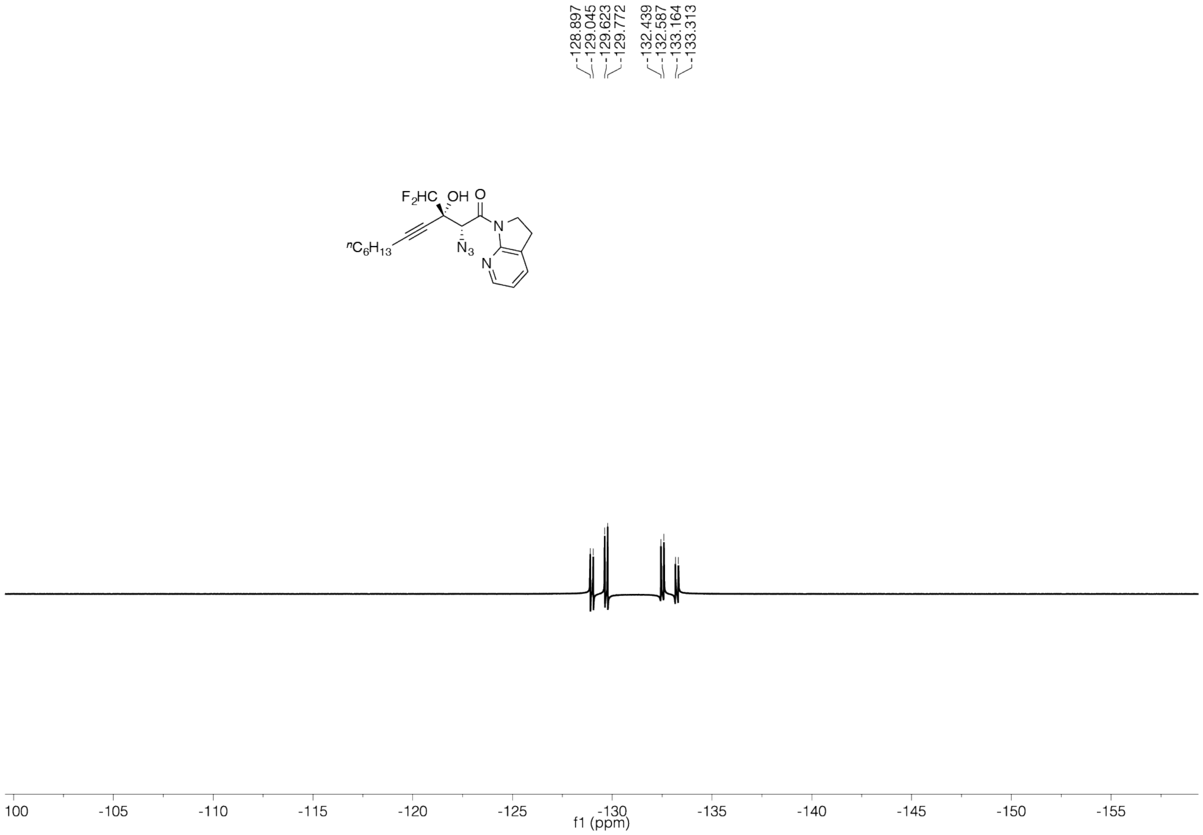

(2*R*,3*S*)-2-Azido-3-(chlorodifluoromethyl)-1-(2,3-dihydro-1*H*-pyrrolo[2,3-*b*]pyridin-1-yl)-3-hydroxyundec-4-yn-1-one (12b):

^{1}H NMR (400MHz, $CDCl_3$)

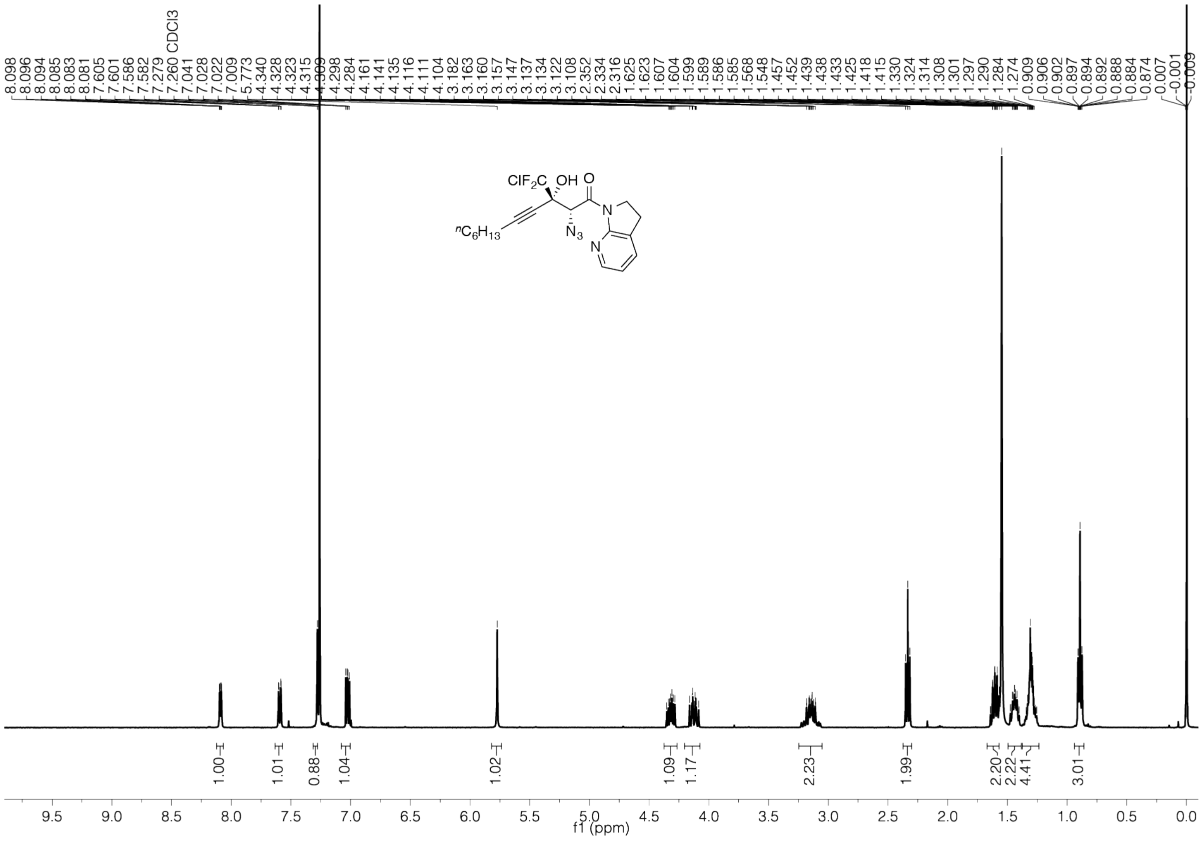

^{13}C NMR (100MHz, $CDCl_3$)

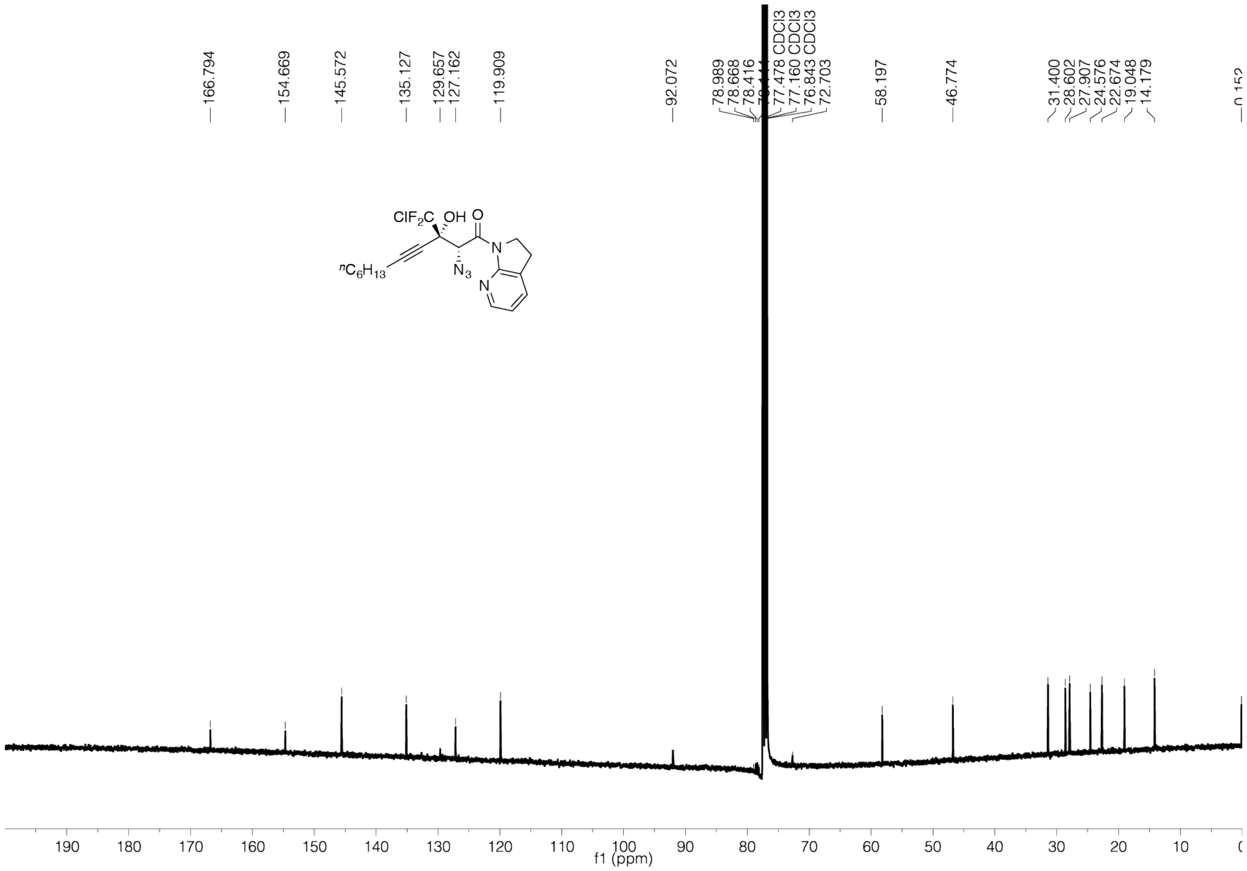

^{19}F NMR (376MHz, $CDCl_3$)

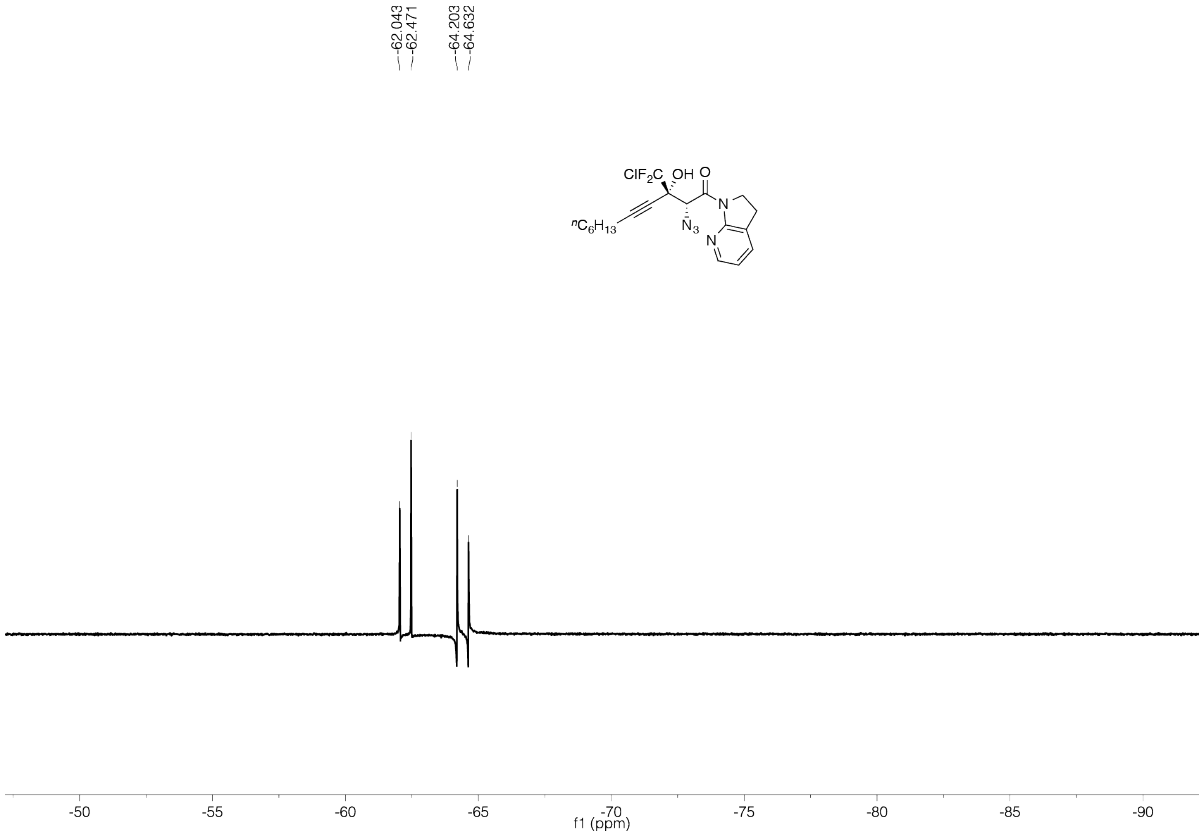

(2*R*,3*S*)-2-Azido-3-(bromodifluoromethyl)-1-(2,3-dihydro-1*H*-pyrrolo[2,3-*b*]pyridin-1-yl)-3-hydroxyundec-4-yn-1-one (12c):

^{1}H NMR (400MHz, $CDCl_3$)

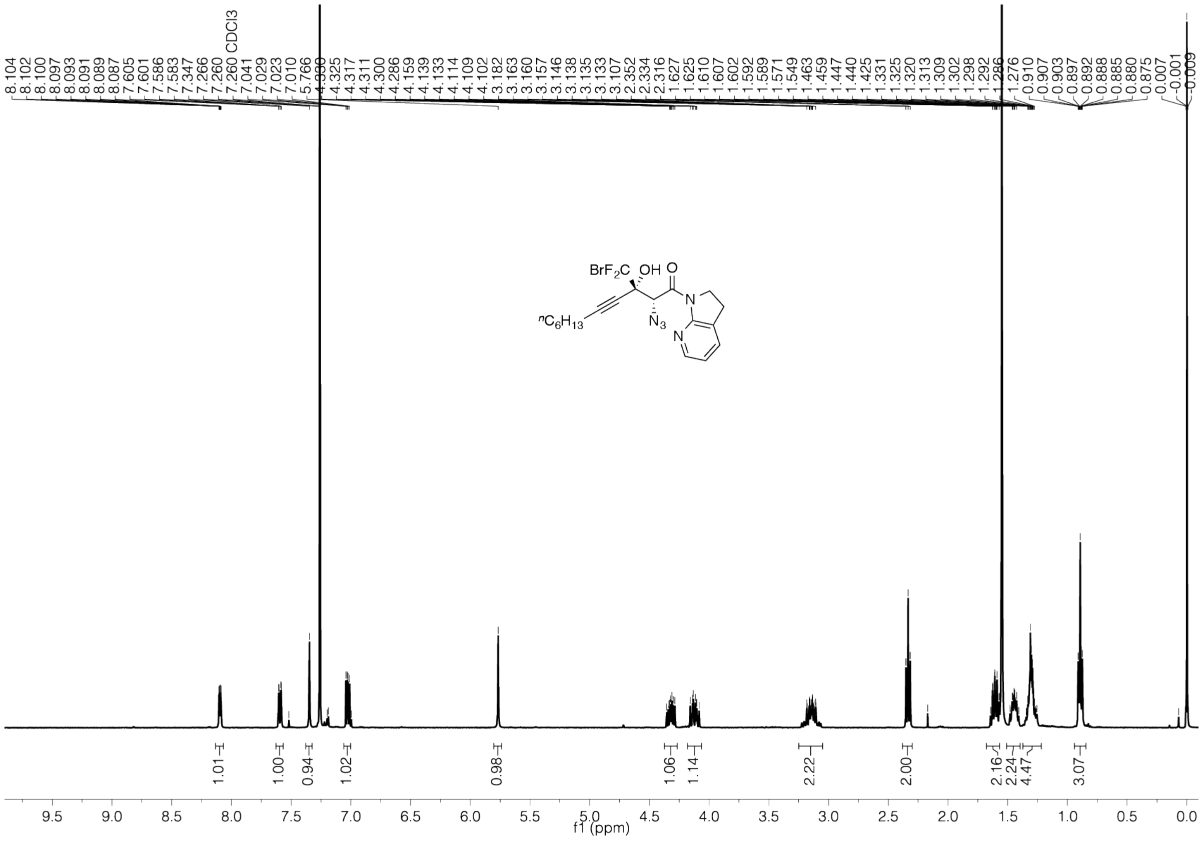

^{13}C NMR (100MHz, $CDCl_3$)

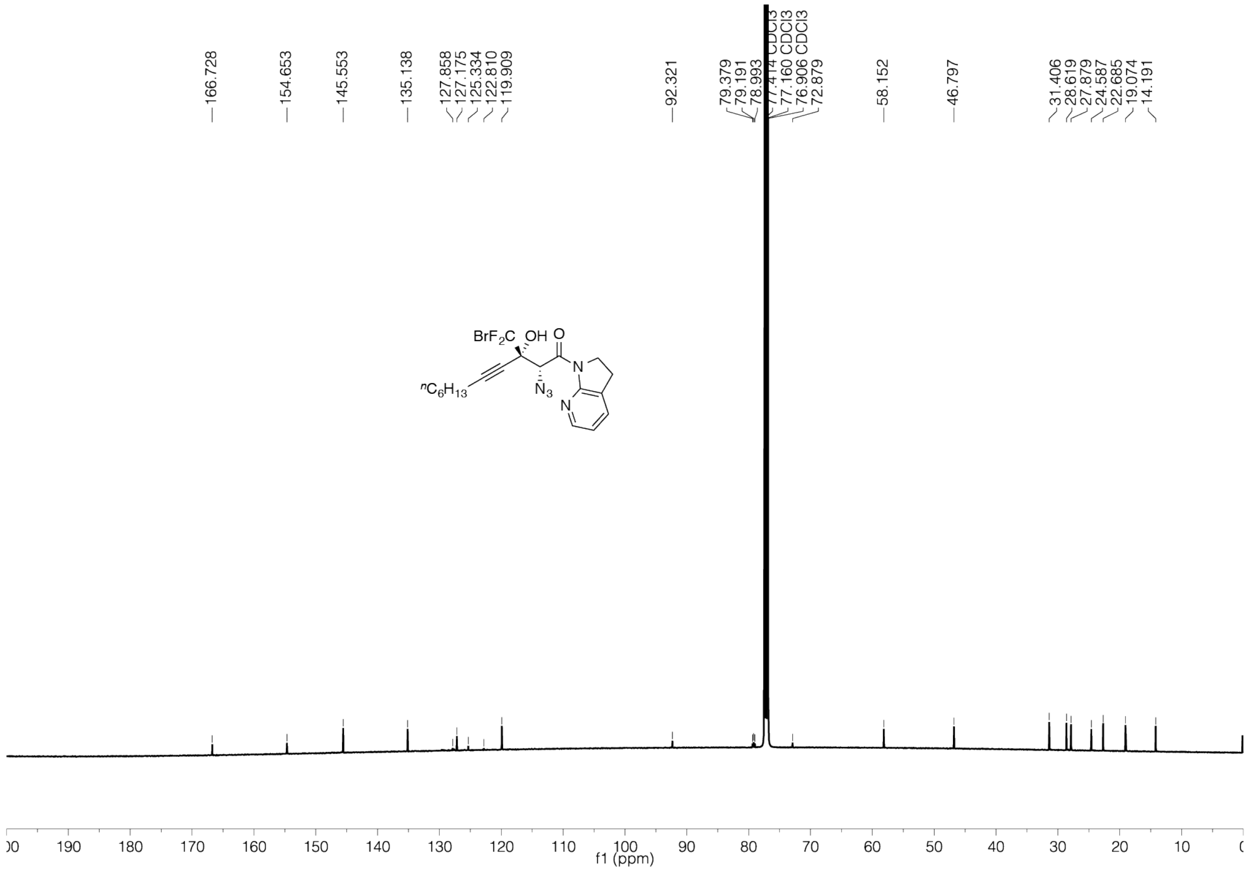

^{19}F NMR (376MHz, $CDCl_3$)

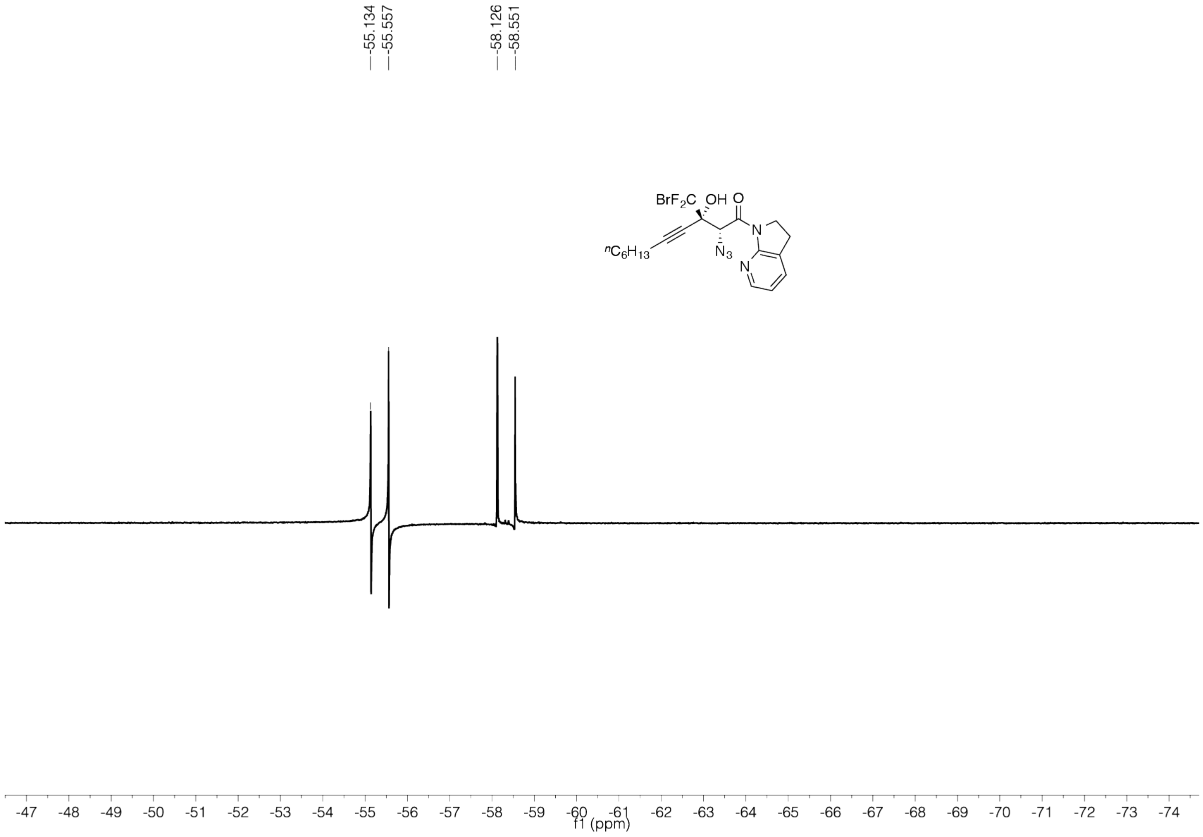

Methyl (2*R*,3*S*)-2-azido-3-hydroxy-3-(trifluoromethyl)-5-(triisopropylsilyl)pent-4-ynoate (13):

^{1}H NMR (500MHz, C_6D_6)

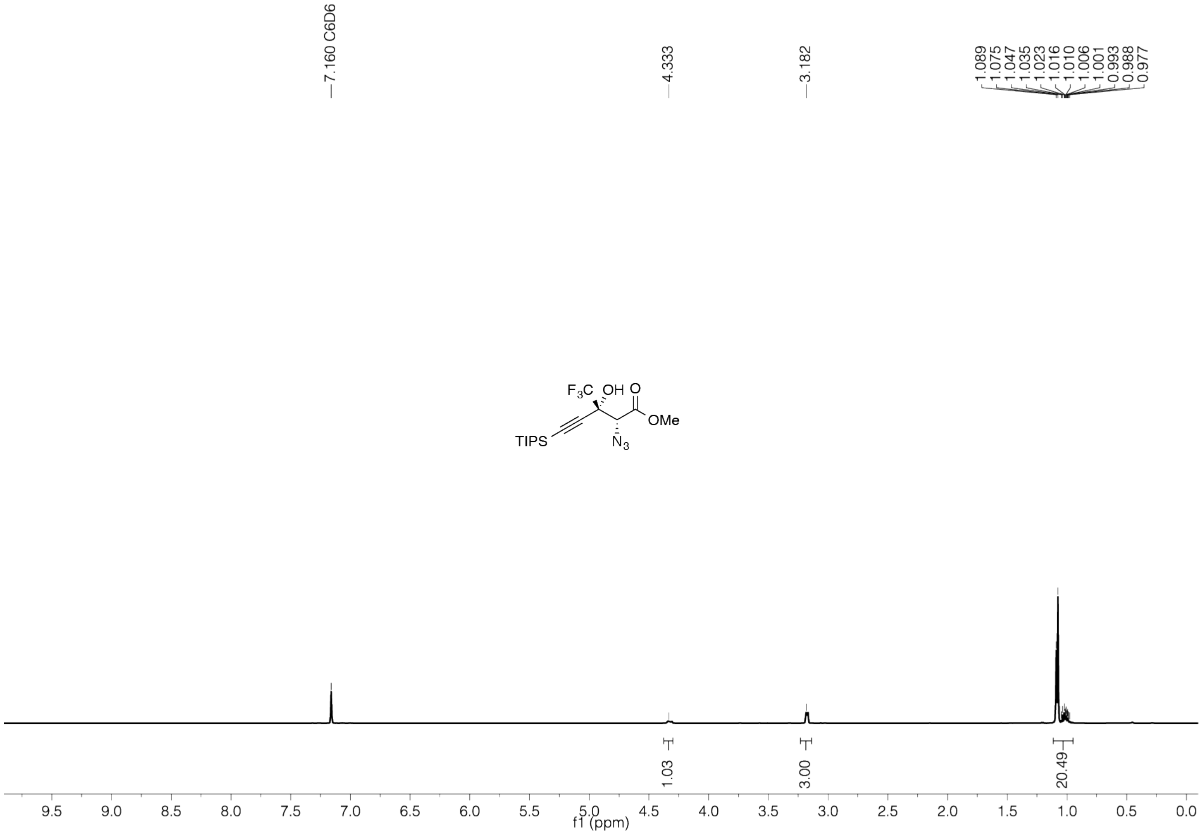

^{13}C NMR (125MHz, C_6D_6)

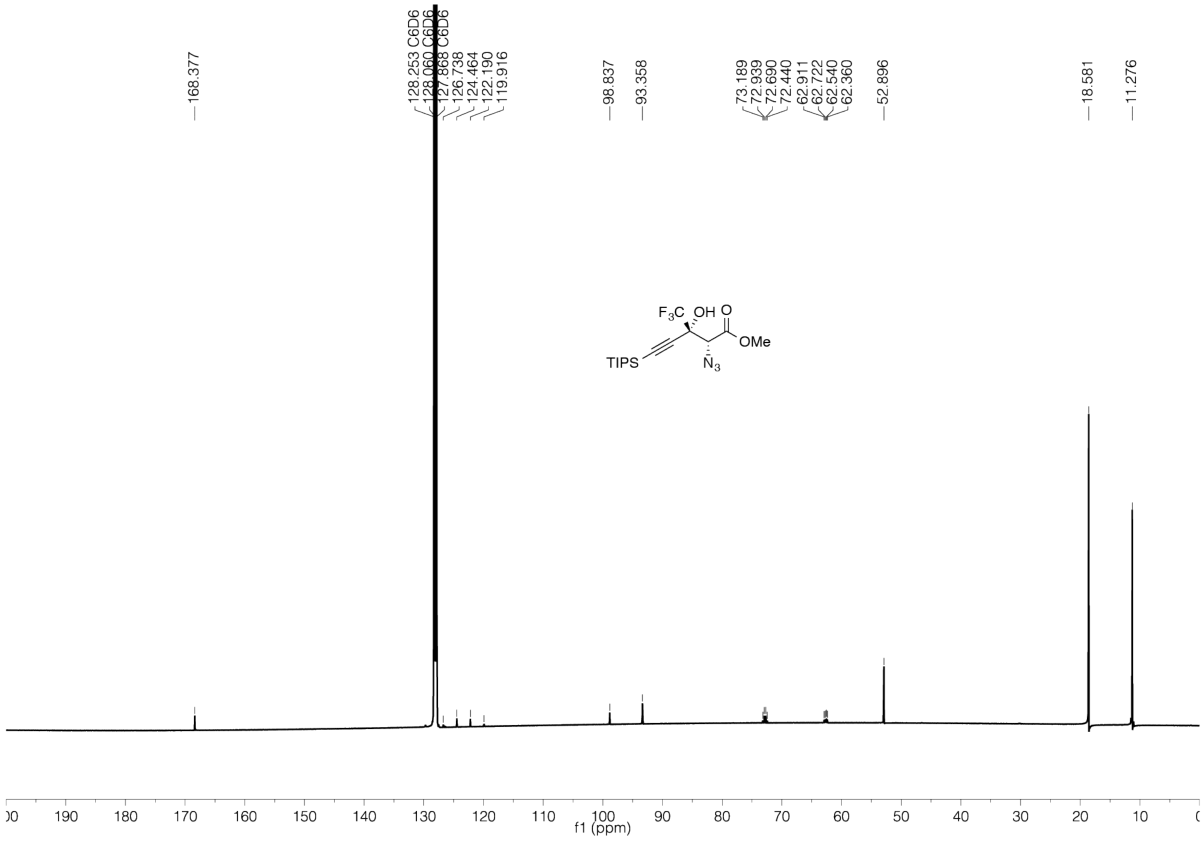

¹⁹F NMR (376MHz, $CDCl_3$)

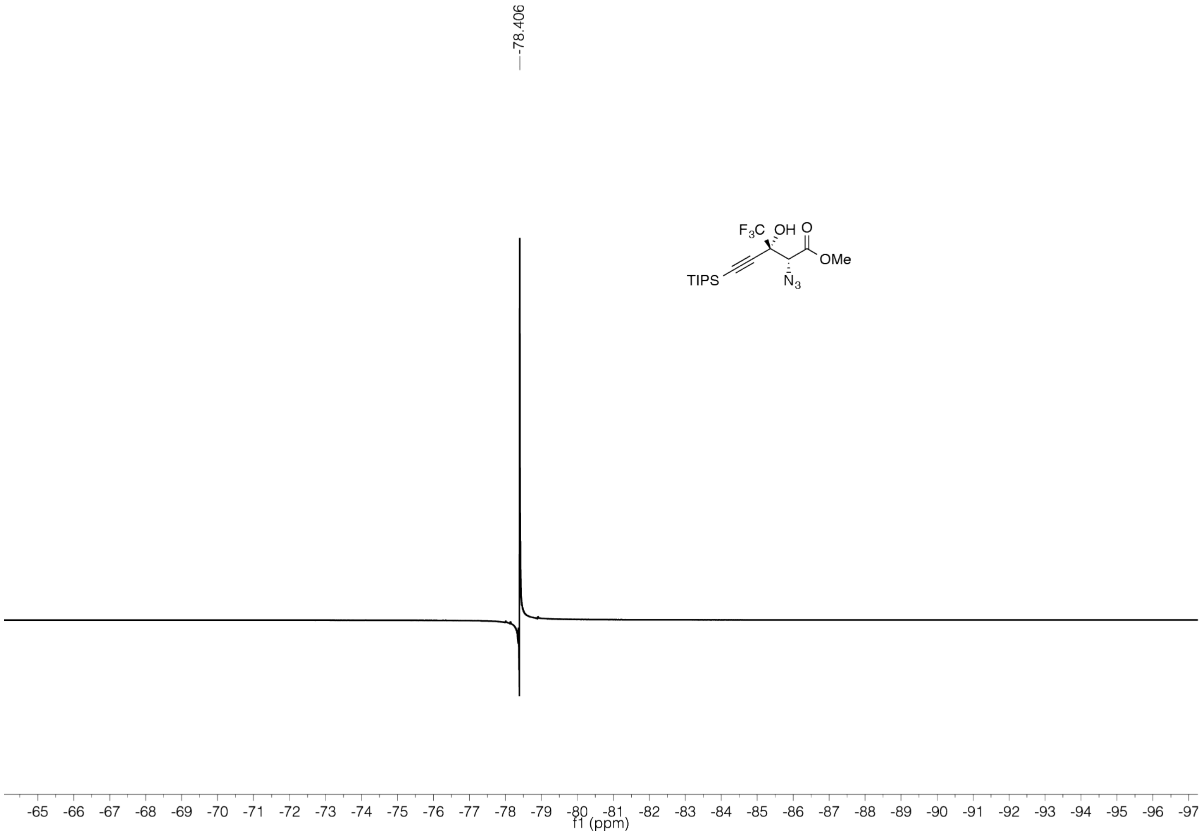

Methyl (2*R*,3*S*)-2-azido-3-hydroxy-3-(trifluoromethyl)pent-4-ynoate (14):

¹H NMR (400MHz, $CDCl_3$)

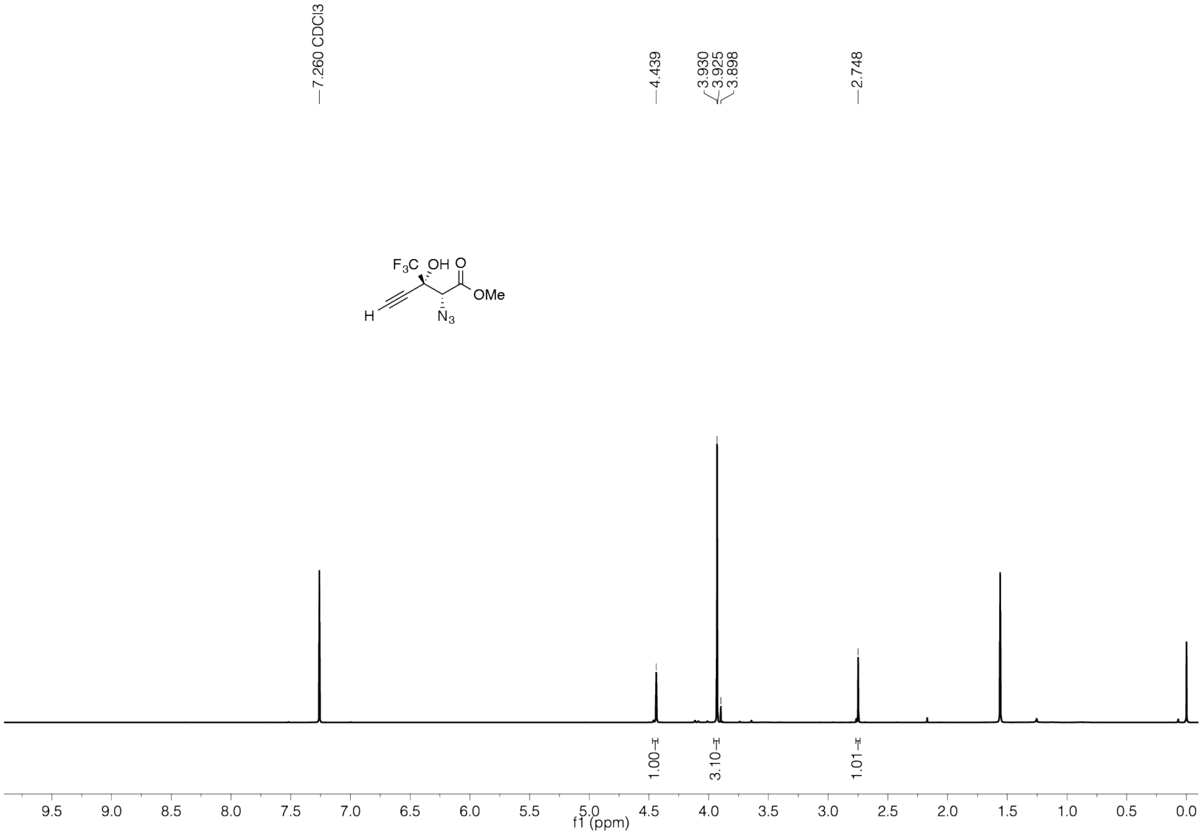

¹³C NMR (100MHz, $CDCl_3$)

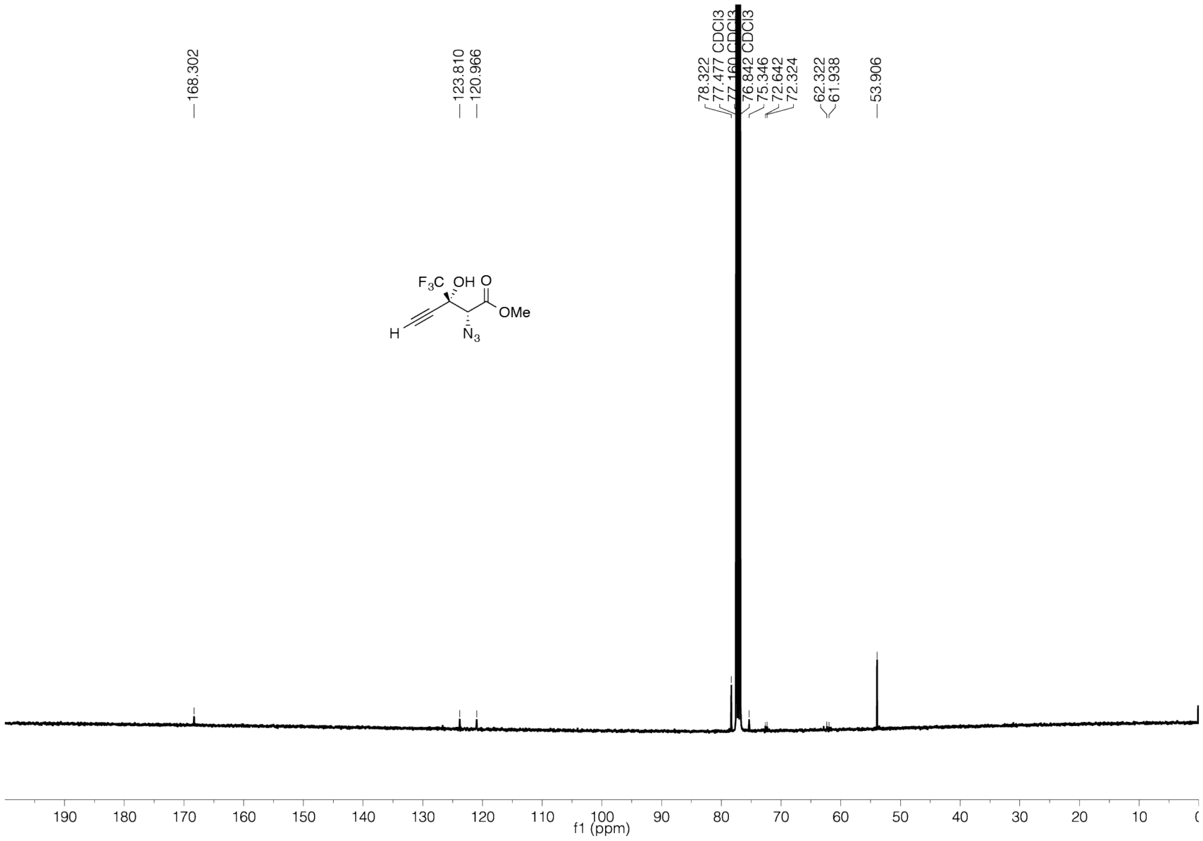

^{19}F NMR (376MHz, $CDCl_3$)

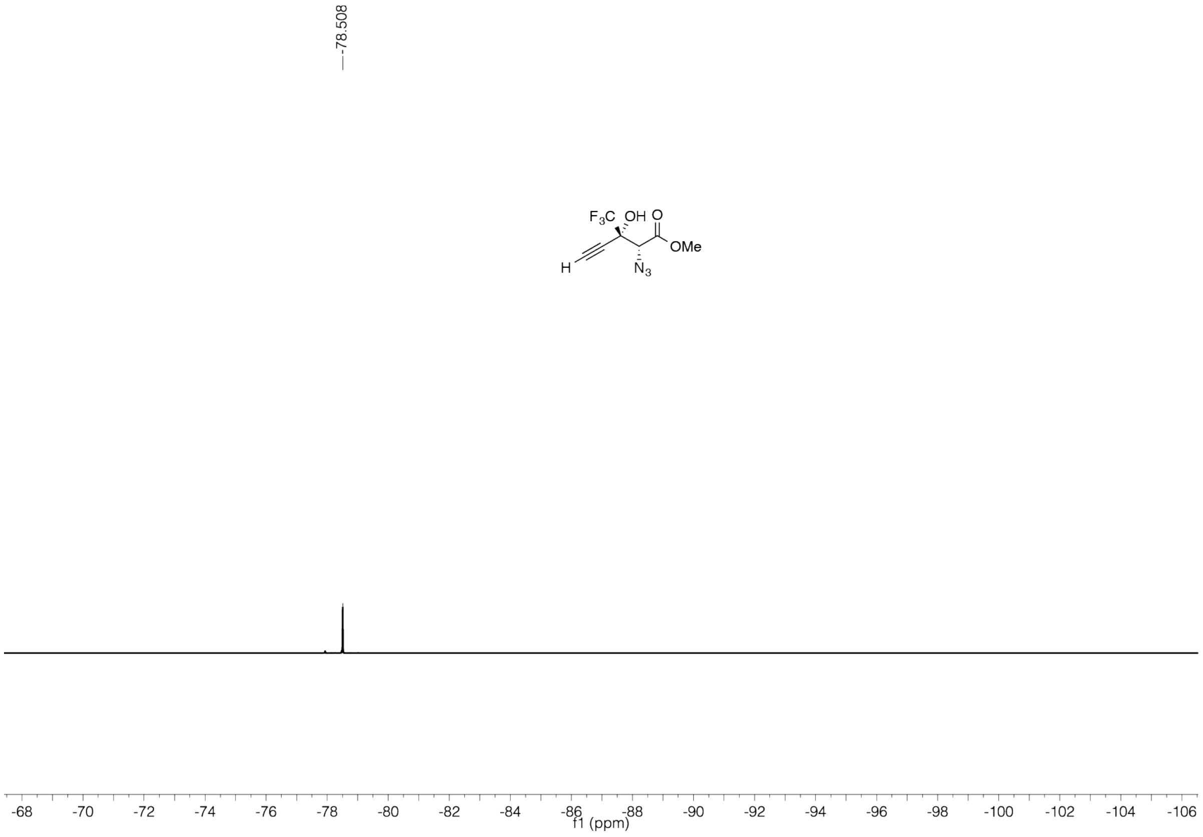

(9*H*-fluoren-9-yl)methyl ((2*R*,3*S*)-1-(2,3-dihydro-1*H*-pyrrolo[2,3-*b*]pyridin-1-yl)-3-hydroxy-1-oxo-5-(*p*-tolyl)-3-(trifluoromethyl)pentan-2-yl)carbamate (S1):

^{1}H NMR (400MHz, $CDCl_3$)

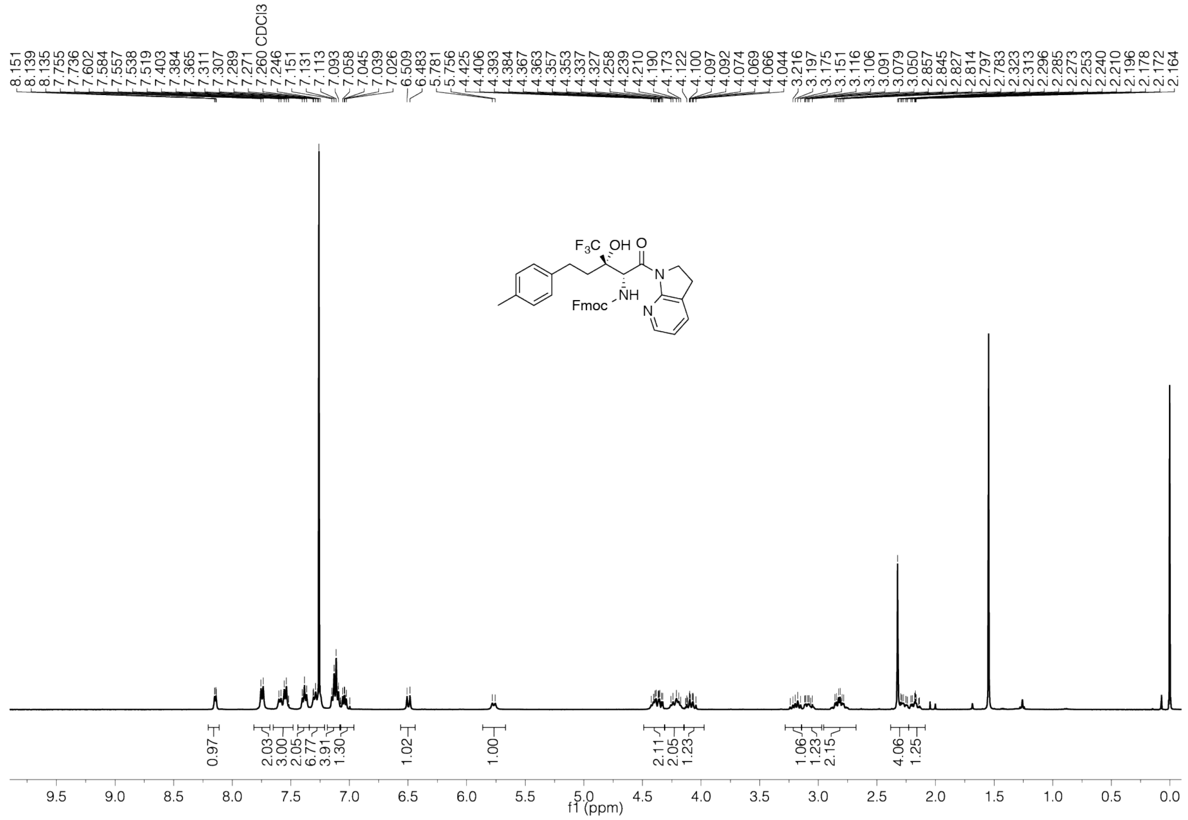

^{13}C NMR (125MHz, $CDCl_3$)

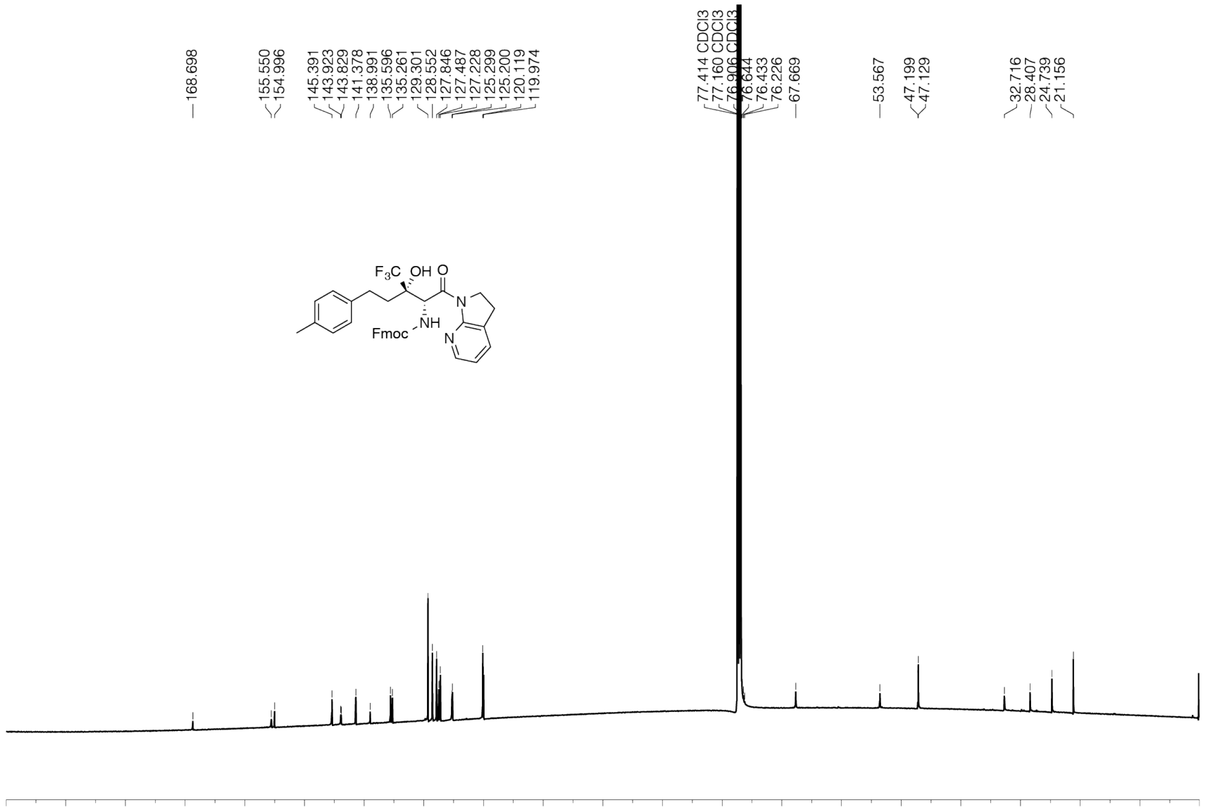

^{19}F NMR (376MHz, $CDCl_3$)

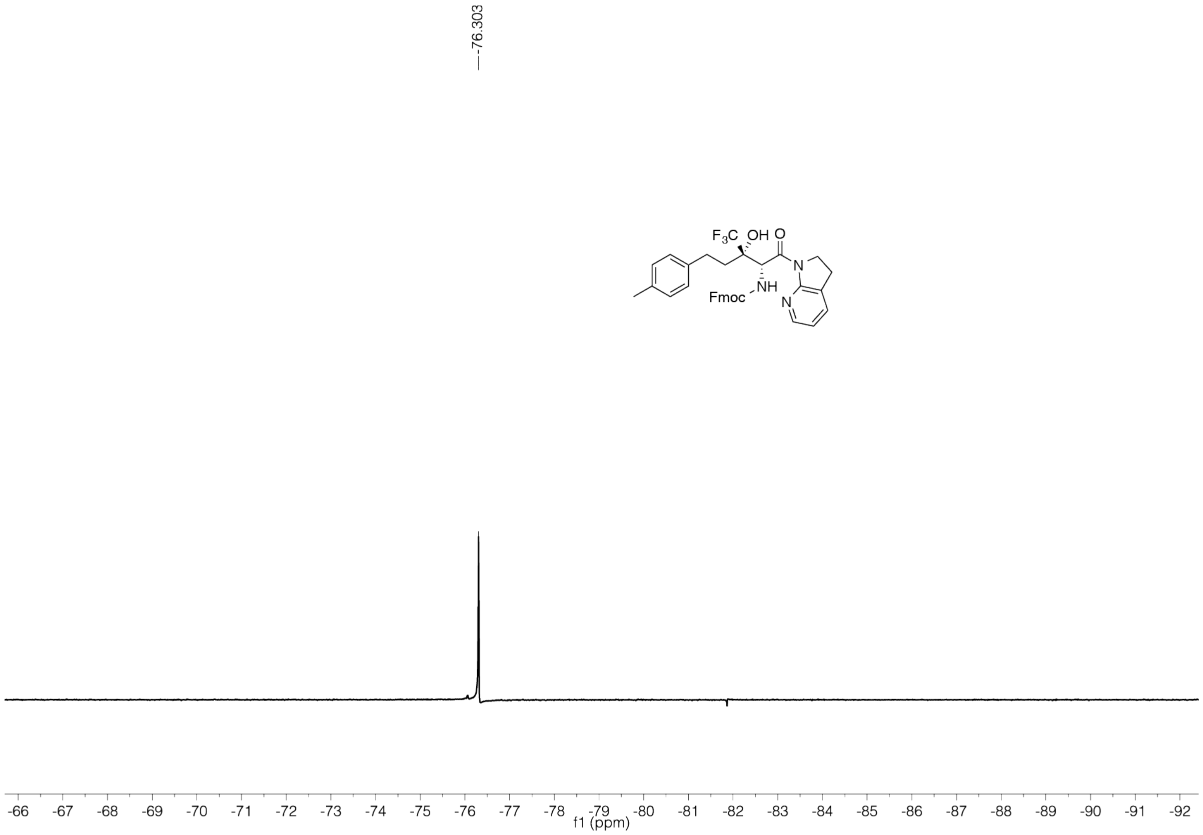

Methyl (2*R*,3*S*)-2-(((9*H*-fluoren-9-yl)methoxy)carbonyl)amino)-3-hydroxy-5-(*p*-tolyl)-3-(trifluoromethyl)pentanoate (15):

^{1}H NMR (500 MHz, $CDCl_3$, 273K)

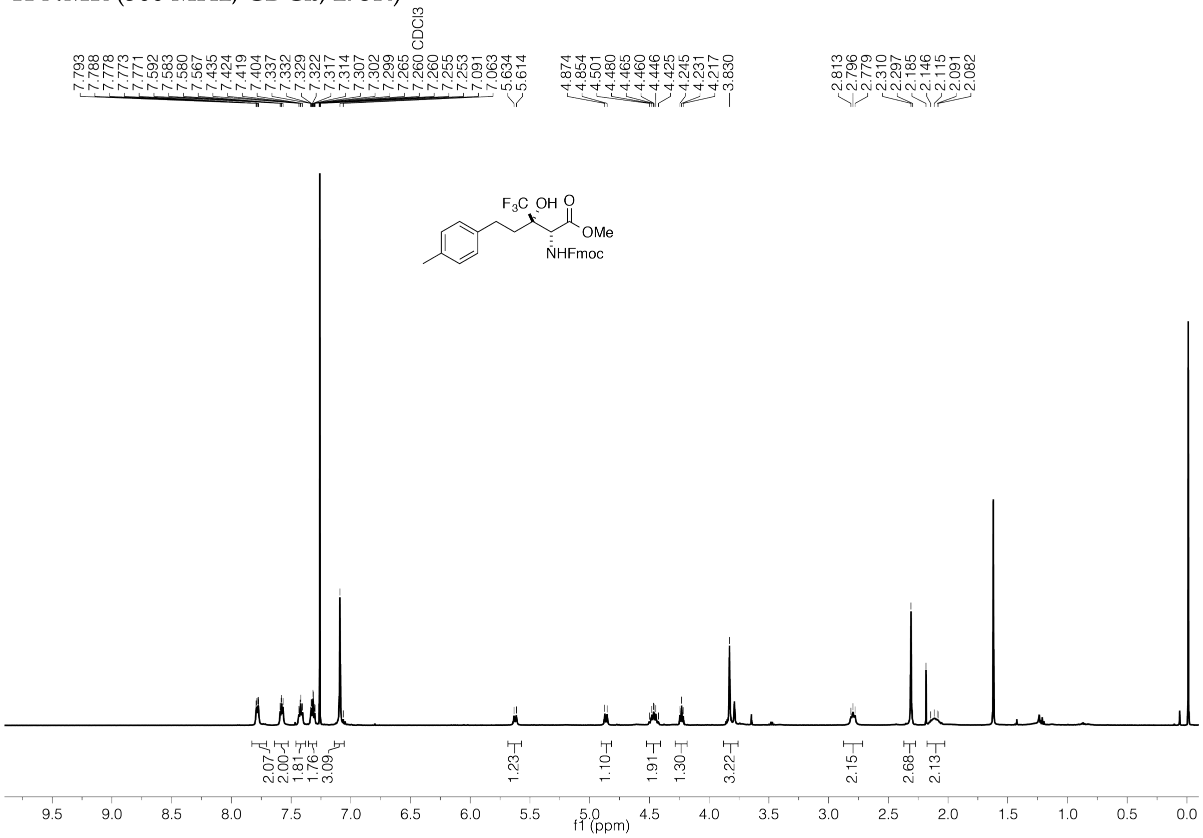

^{13}C NMR (125MHz, $CDCl_3$, 273K)

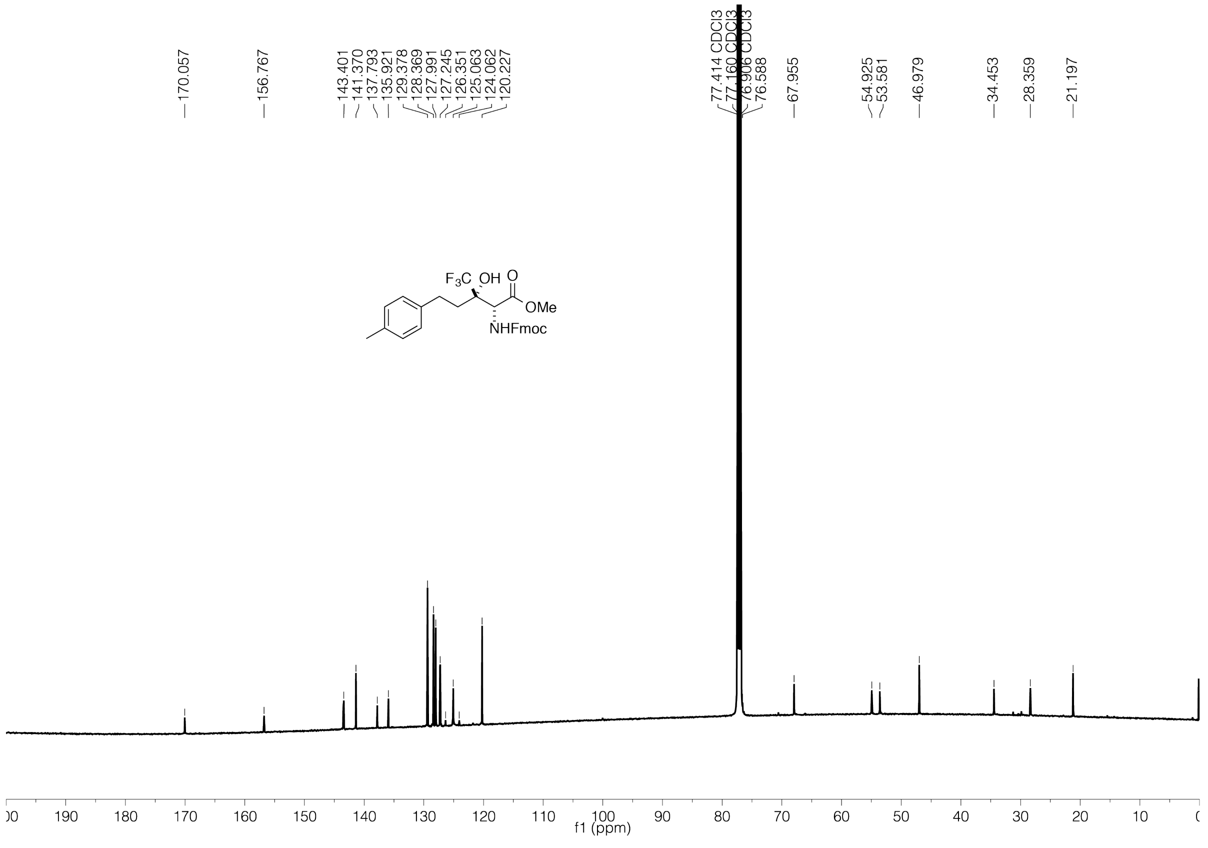

^{19}F NMR (376MHz, CDCl3)

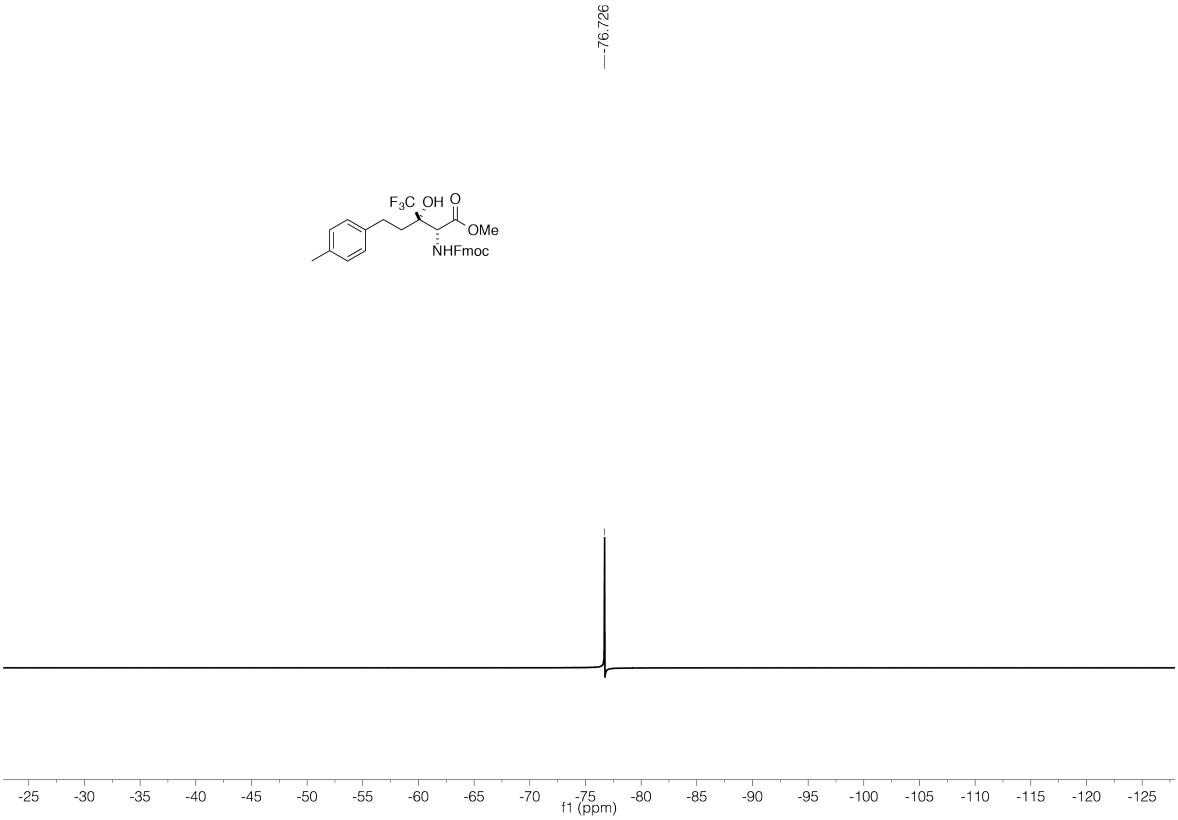

Supplement: Supplementary file 1 [file SC-008-C7SC00330G-s001.pdf]
